# Supplementary material for: The cohort trends of social connectedness in secondary school students in Finland between 2017 and 2021
Source: PLoS One. 2024 Oct 28;19(10):e0312579. doi: 10.1371/journal.pone.0312579 (PMC11516007; doi:10.1371/journal.pone.0312579)
Supplement: S1 File — (PDF) [file pone.0312579.s001.pdf]

S1 Online resource.

Stata outputs for the models

Table of Contents

1. Description on the online resource 1

2. Multivariable regressions with main effects only, unstandardized estimates and margins for study year 3

2.1. Number of close friends 3

2.2. Loneliness 3

2.3. Belonging at school 5

3. Multivariable regressions with interactions, unstandardized estimates and margins 7

3.1. Number of close friends 7

3.2. Loneliness 18

3.3. Belonging at school 28

4. Multivariable regressions with marginal effects 40

4.1. Close friends with Odds Ratios and marginal effects 40

4.2. Loneliness with exponentiated Beta and marginal effects 47

4.3. Belonging at school with exponentiated Beta and marginal effects 51

1. Description on the online resource

This file shows the original model results as Stata outputs.

* Section 2 shows the multivariable regressions with unstandardized estimates for three outcomes (number of close friends, loneliness and belonging at school). For each outcome, the main effect models with the predicted margins for study year are shown (2.1., 2.2. and 2.3). These models were used for the general illustration of the trends over time (”***General trends in social connectedness between 2017 and 2021***” in the results section of the main text and ***Supplementary Figures 1-5***).

* Section 3 shows the models with the interactions. First, the interaction year*gender*school level was added to the models (3.1., 3.2. and 3.3.) and the predicted margins were calculated for the significant interaction (or lower level interaction with year) in the model (“***The effects of year, gender and school level on social connectedness***” in the results section of the main text and ***Supplementary Figures 6-9***). Second, the interactions of parental education, urban-rural location of the school and immigration status of the student were added to the model with any relevant interactions with year, and gender or school level and the predicted margins were calculated for the significant interaction in the final model (“***The effects of socioeconomic factors on the trends of social connectedness***” in the results section of the main text and ***Supplementary Figures 10-17***).

* Section 4 shows the models with Odds rations or exponentiated Betas and marginal effects. This section is used for estimating the % changes and effect sizes. First, the main effect models are shown followed by the

final models for year*gender*school level interactions and the significant interactions with parental education, urban-rural location of the school and migrant status of the student added (as found in sections 2 and 3). For the interpretation of the effect sizes, Chen et al (2010) suggests the Odds Ratio cut-offs of 1.68/0.60, 3.47/0.29 and 6.71/0.15 for small, medium and large effect. For standardized regression coefficients, Nieminen (2022) suggests the ranges 1.10-0.29, 0.20-0.49 and 0.5 or more for small, medium and large effects. In the case of generalized linear models, the marginal effects (dy/dx in the Stata output) can be interpreted as a standardized coefficient. It is important to notice that all models in the current study required complex calculations (ordinal logit and generalized linear models with interactions). Therefore, the interpretation of the effect sizes should be done with care. In the observational studies with psychosocial constructs, effect sizes are generally small (Funder & Ozer, 2019).

References

Chen, H., Cohen, P., & Chen, S. (2010). How Big is a Big Odds Ratio? Interpreting the Magnitudes of Odds Ratios in Epidemiological Studies. Communications in Statistics—Simulation and Computation®, 39(4), 860–864. https://doi.org/10.1080/03610911003650383

Funder, D. C., & Ozer, D. J. (2019). Evaluating Effect Size in Psychological Research: Sense and Nonsense. *Advances in Methods and Practices in Psychological Science*, *2*(2), 156–168. https://doi.org/10.1177/2515245919847202

Nieminen, P. (2022). Application of Standardized Regression Coefficient in Meta-Analysis. BioMedInformatics 2022, Vol. 2, Pages 434-458, 2(3), 434–458. https://doi.org/10.3390/BIOMEDINFORMATICS2030028

2. Multivariable regressions with main effects only, unstandardized estimates and margins for study year

2.1. Number of close friends

```
. ologit closefriend i.year i.gender i.year i.schoollevel i.degree i.urbanrural i.immigrant, vce(robust) nolog

Ordered logistic regression                         Number of obs = 415,932
                                                    Wald chi2(11) = 4036.20
                                                    Prob > chi2   =  0.0000
Log pseudolikelihood = -486744.17                   Pseudo R2     =  0.0042

---------------------------------------------------------------------------------------------------------
                                       |               Robust
                           closefriend | Coefficient  std. err.      z    P>|z|     [95% conf. interval]
---------------------------------------+-----------------------------------------------------------------
                                  year |
                                  2019  |  -.0586656    .007513    -7.81   0.000    -.0733909   -.0439404
                                  2021  |  -.2248052   .0073028   -30.78   0.000    -.2391185    -.210492
                                        |
                                gender |
                                  girl  |  -.2080321   .0060658   -34.30   0.000     -.219921   -.1961433
                                        |
                           schoollevel |
                       uppersecondary  |   .0403563   .0067786     5.95   0.000     .0270705    .0536422
                           vocational  |   .0404798   .0086019     4.71   0.000     .0236205    .0573392
                                        |
                              1.degree  |   .1380811   .0061066    22.61   0.000     .1261124    .1500498
                                        |
                            urbanrural |
                            semiurban  |  -.0323388   .0079669    -4.06   0.000    -.0479537    -.016724
                                rural  |  -.0892156   .0092615    -9.63   0.000    -.1073677   -.0710634
                                        |
                             immigrant |
              one parent foreign-born  |  -.1462059   .0113425   -12.89   0.000    -.1684369    -.123975
born in Finland parents foreign-born   |  -.2424084   .0213443   -11.36   0.000    -.2842425   -.2005743
     student and parents born abroad   |  -.5141903   .0181466   -28.34   0.000    -.5497571   -.4786236
---------------------------------------+-----------------------------------------------------------------
                                 /cut1 |  -2.622885   .0099498                      -2.642386   -2.603384
                                 /cut2 |  -1.206868   .0086493                       -1.22382   -1.189916
                                 /cut3 |  -.2915825   .0083454                      -.3079392   -.2752259
---------------------------------------------------------------------------------------------------------

. margins year

Predictive margins                                  Number of obs = 415,932
Model VCE: Robust

1._predict: Pr(closefriend==1), predict(pr outcome(1))
2._predict: Pr(closefriend==2), predict(pr outcome(2))
3._predict: Pr(closefriend==3), predict(pr outcome(3))
4._predict: Pr(closefriend==4), predict(pr outcome(4))

--------------------------------------------------------------------------------
              |            Delta-method
              |     Margin   std. err.      z    P>|z|     [95% conf. interval]
--------------+-----------------------------------------------------------------
_predict#year |
      1 2017  |    .0726187    .000501   144.96   0.000     .0716368    .0736006
      1 2019  |    .0766612   .0005063   151.41   0.000     .0756688    .0776535
      1 2021  |    .0892484   .0005463   163.36   0.000     .0881776    .0903192
      2 2017  |     .170402   .0007817   217.99   0.000       .16887    .1719341
      2 2019  |    .1772545   .0007619   232.65   0.000     .1757612    .1787477
      2 2021  |    .1972145   .0007864   250.77   0.000     .1956731    .1987558
      3 2017  |     .200595   .0006751   297.12   0.000     .1992718    .2019183
      3 2019  |     .204127   .0006628   307.96   0.000     .2028279    .2054262
      3 2021  |    .2127395   .0006693   317.87   0.000     .2114277    .2140512
      4 2017  |    .5563842   .0013716   405.65   0.000     .5536959    .5590725
      4 2019  |    .5419573   .0012787   423.82   0.000      .539451    .5444636
      4 2021  |    .5007977   .0012196   410.63   0.000     .4984073     .503188
--------------------------------------------------------------------------------
```

2.2. Loneliness

```
. glm loneliness i.year i.gender i.schoollevel i.degree  i.immigrant i.urbanrural, family(gamma) link (log) vce(robust) nolog

Generalized linear models                      Number of obs   =    416,572
Optimization     : ML                          Residual df     =    416,560
                                               Scale parameter =   .1957444
Deviance         =  84570.54471                (1/df) Deviance =   .2030213
Pearson          =  81539.28191                (1/df) Pearson  =   .1957444

Variance function: V(u) = u^2                  [Gamma]
Link function    : g(u) = ln(u)                [Log]

                                               AIC             =    3.63757
Log pseudolikelihood = -757642.8251            BIC             =   -5305639

---------------------------------------------------------------------------------------------------------
                                       |               Robust
                            loneliness | Coefficient  std. err.      z    P>|z|     [95% conf. interval]
---------------------------------------+-----------------------------------------------------------------
                                  year |
                                  2019  |   .0239107   .0017595    13.59   0.000     .0204622    .0273592
```

```
                             2021  |   .1284306    .001715    74.89   0.000     .1250693    .1317919
                                   |
                           gender  |
                             girl  |   .2670785   .0013944   191.53   0.000     .2643455    .2698116
                                   |
                      schoollevel  |
                  uppersecondary   |    .063795   .0015226    41.90   0.000     .0608107    .0667793
                       vocational  |   .0257583   .0020755    12.41   0.000     .0216903    .0298262
                                   |
                         1.degree  |  -.0084148   .0014342    -5.87   0.000    -.0112257   -.0056039
                                   |
                        immigrant  |
        one parent foreign-born    |   .0467886   .0026629    17.57   0.000     .0415695    .0520077
born in Finland parents foreign-born  |  -.0300573   .0056517    -5.32   0.000    -.0411344   -.0189801
     student and parents born abroad  |   .0642911   .0048561    13.24   0.000     .0547732    .0738089
                                   |
                       urbanrural  |
                        semiurban  |  -.0117577   .0018729    -6.28   0.000    -.0154285   -.0080868
                            rural  |  -.0038989   .0021753    -1.79   0.073    -.0081625    .0003646
                                   |
                            _cons  |   .6023487   .0019771   304.66   0.000     .5984735    .6062238
-----------------------------------------------------------------------------------------------------

. margins year

Predictive margins                                       Number of obs = 416,572
Model VCE: Robust

Expression: Predicted mean loneliness, predict()

------------------------------------------------------------------------------
             |            Delta-method
             |     Margin   std. err.      z    P>|z|     [95% conf. interval]
-------------+----------------------------------------------------------------
        year |
       2017  |   2.167624   .0028151   770.00   0.000     2.162107    2.173142
       2019  |   2.220078   .0025969   854.89   0.000     2.214988    2.225168
       2021  |   2.464681   .0027019   912.21   0.000     2.459385    2.469976
------------------------------------------------------------------------------


. ologit loneliness i.year i.gender i.year i.schoollevel i.degree i.urbanrural i.immigrant, vce(robust) nolog

Ordered logistic regression                             Number of obs =  416,572
                                                        Wald chi2(11) = 47351.31
                                                        Prob > chi2   =   0.0000
Log pseudolikelihood = -558852.72                       Pseudo R2     =   0.0424

-----------------------------------------------------------------------------------------------------
                                   |               Robust
                        loneliness | Coefficient  std. err.      z    P>|z|     [95% conf. interval]
-----------------------------------+-----------------------------------------------------------------
                              year |
                             2019  |   .1038399   .0070423    14.75   0.000     .0900372    .1176426
                             2021  |   .5487565   .0071102    77.18   0.000     .5348208    .5626922
                                   |
                           gender  |
                             girl  |   1.148096   .0059573   192.72   0.000      1.13642    1.159772
                                   |
                      schoollevel  |
                  uppersecondary   |   .2814299   .0063567    44.27   0.000      .268971    .2938888
                       vocational  |   .1136658   .0085384    13.31   0.000     .0969307    .1304008
                                   |
                         1.degree  |   -.033569   .0059129    -5.68   0.000     -.045158     -.02198
                                   |
                       urbanrural  |
                        semiurban  |  -.0499734   .0076504    -6.53   0.000    -.0649679   -.0349788
                            rural  |  -.0151118   .0089418    -1.69   0.091    -.0326375    .0024139
                                   |
                        immigrant  |
        one parent foreign-born    |   .1760686   .0112222    15.69   0.000     .1540735    .1980638
born in Finland parents foreign-born  |  -.2155207   .0236075    -9.13   0.000    -.2617905   -.1692509
     student and parents born abroad  |    .105432    .020701     5.09   0.000     .0648587    .1460053
-----------------------------------+-----------------------------------------------------------------
                             /cut1 |  -.2244356   .0079574                         -.2400319   -.2088392
                             /cut2 |    1.40446   .0082743                          1.388243    1.420678
                             /cut3 |   3.033756   .0093738                          3.015383    3.052128
                             /cut4 |   4.518506   .0121754                          4.494642    4.542369
-----------------------------------------------------------------------------------------------------

. margins year

Predictive margins                                       Number of obs = 416,572
Model VCE: Robust

1._predict: Pr(loneliness==1), predict(pr outcome(1))
2._predict: Pr(loneliness==2), predict(pr outcome(2))
3._predict: Pr(loneliness==3), predict(pr outcome(3))
4._predict: Pr(loneliness==4), predict(pr outcome(4))
5._predict: Pr(loneliness==5), predict(pr outcome(5))

-------------------------------------------------------------------------------
              |            Delta-method
              |     Margin   std. err.      z    P>|z|     [95% conf. interval]
--------------+----------------------------------------------------------------
_predict#year |
      1 2017  |    .300567   .0010911   275.47   0.000     .2984285    .3027055
      1 2019  |   .2806327   .0009837   285.27   0.000     .2787046    .2825607
      1 2021  |   .2043359   .0008503   240.30   0.000     .2026693    .2060026
      2 2017  |     .35774   .0007595   470.99   0.000     .3562514    .3592287
      2 2019  |   .3557213   .0007592   468.56   0.000     .3542333    .3572092
      2 2021  |   .3330526   .0007569   440.02   0.000     .3315691    .3345361
      3 2017  |    .242455   .0008189   296.08   0.000     .2408501      .24406
      3 2019  |   .2550103   .0007911   322.36   0.000     .2534598    .2565607
      3 2021  |   .3048692    .000846   360.35   0.000      .303211    .3065274
      4 2017  |   .0743551   .0004647   160.02   0.000     .0734444    .0752659
      4 2019  |   .0811256   .0004789   169.40   0.000      .080187    .0820642
      4 2021  |   .1156401   .0006241   185.30   0.000     .1144169    .1168632
      5 2017  |   .0248828   .0002408   103.32   0.000     .0244108    .0253549
      5 2019  |   .0275102   .0002579   106.67   0.000     .0270048    .0280157
      5 2021  |   .0421023   .0003776   111.51   0.000     .0413622    .0428423
-------------------------------------------------------------------------------
```

2.3. Belonging at school

```
. glm belonging i.year i.gender i.schoollevel i.degree  i.immigrant i.urbanrural, family(gaussian) link (identity) vce(robust) nolog

Generalized linear models                         Number of obs   =    415,367
Optimization     : ML                             Residual df     =    415,355
                                                  Scale parameter =   .8968263
Deviance         =  372501.3057                   (1/df) Deviance =   .8968263
Pearson          =  372501.3057                   (1/df) Pearson  =   .8968263

Variance function: V(u) = 1                       [Gaussian]
Link function    : g(u) = u                       [Identity]

                                                  AIC             =   2.729013
Log pseudolikelihood = -566758.9554               BIC             =   -5000912

--------------------------------------------------------------------------------------------------
                                   |               Robust
                         belonging | Coefficient  std. err.      z    P>|z|     [95% conf. interval]
-----------------------------------+--------------------------------------------------------------
                              year |
                              2019  |  -.2135893   .0037169   -57.46   0.000    -.2208742   -.2063044
                              2021  |  -.2853264   .0037167   -76.77   0.000     -.292611   -.2780418
                                   |
                            gender |
                              girl  |  -.3444783   .0029673  -116.09   0.000    -.3502941   -.3386624
                                   |
                       schoollevel |
                   uppersecondary  |   .0295346   .0033845     8.73   0.000     .0229012    .0361681
                       vocational  |   .1818368   .0041806    43.50   0.000      .173643    .1900306
                                   |
                          1.degree |   .0887873   .0030714    28.91   0.000     .0827675    .0948071
                                   |
                         immigrant |
          one parent foreign-born  |  -.0569491   .0059226    -9.62   0.000    -.0685571    -.045341
born in Finland parents foreign-born  |   .0345385    .011182     3.09   0.002     .0126222    .0564548
     student and parents born abroad  |  -.0776721   .0100215    -7.75   0.000    -.0973138   -.0580304
                                   |
                        urbanrural |
                        semiurban  |   .0279224   .0039767     7.02   0.000     .0201283    .0357166
                            rural  |   .0638874   .0047192    13.54   0.000     .0546378    .0731369
                                   |
                             _cons |   3.791712   .0041059   923.49   0.000     3.783665    3.799759
--------------------------------------------------------------------------------------------------

. margins year

Predictive margins                                     Number of obs = 415,367
Model VCE: Robust

Expression: Predicted mean belonging, predict()

------------------------------------------------------------------------------
             |            Delta-method
             |     Margin   std. err.      z    P>|z|     [95% conf. interval]
-------------+----------------------------------------------------------------
        year |
       2017  |    3.70178   .0027962  1323.88   0.000       3.6963    3.707261
       2019  |   3.488191   .0024495  1424.04   0.000      3.48339    3.492992
       2021  |   3.416454   .0024392  1400.62   0.000     3.411673    3.421235
------------------------------------------------------------------------------

.
. ologit belongmeanr5 i.year i.gender i.year i.schoollevel i.degree i.urbanrural i.immigrant, vce(robust) nolog

Ordered logistic regression                          Number of obs =  415,367
                                                     Wald chi2(11) = 25036.24
                                                     Prob > chi2   =   0.0000
Log pseudolikelihood = -564258.51                    Pseudo R2     =   0.0230

--------------------------------------------------------------------------------------------------
                                   |               Robust
                      belongmeanr5 | Coefficient  std. err.      z    P>|z|     [95% conf. interval]
-----------------------------------+--------------------------------------------------------------
                              year |
                              2019  |   -.486816   .0073944   -65.84   0.000    -.5013088   -.4723232
                              2021  |  -.6318351   .0074106   -85.26   0.000    -.6463596   -.6173106
                                   |
                            gender |
                              girl  |  -.7024651   .0058368  -120.35   0.000     -.713905   -.6910253
                                   |
                       schoollevel |
                   uppersecondary  |   .0885226   .0065325    13.55   0.000     .0757192     .101326
                       vocational  |   .3761295   .0082743    45.46   0.000     .3599122    .3923469
                                   |
                          1.degree |   .1479307   .0059507    24.86   0.000     .1362675    .1595939
                                   |
                        urbanrural |
                        semiurban  |   .0748312   .0076975     9.72   0.000     .0597444     .089918
                            rural  |   .1480747   .0091408    16.20   0.000     .1301591    .1659904
                                   |
                         immigrant |
          one parent foreign-born  |   -.106212   .0113892    -9.33   0.000    -.1285344   -.0838895
born in Finland parents foreign-born  |   .0811309   .0218455     3.71   0.000     .0383144    .1239473
     student and parents born abroad  |  -.0706175   .0195938    -3.60   0.000    -.1090206   -.0322143
-----------------------------------+--------------------------------------------------------------
                             /cut1 |   -3.68503   .0110685                     -3.706723   -3.663336
                             /cut2 |  -2.366012   .0091687                     -2.383982   -2.348042
                             /cut3 |  -.4509971   .0083639                       -.46739   -.4346042
                             /cut4 |   1.306647   .0088265                      1.289348    1.323947
--------------------------------------------------------------------------------------------------

. margins year
```

Predictive margins   Number of obs = 415,367
Model VCE: Robust

1._predict: Pr(belongmeanr5==1), predict(pr outcome(1))
2._predict: Pr(belongmeanr5==2), predict(pr outcome(2))
3._predict: Pr(belongmeanr5==3), predict(pr outcome(3))
4._predict: Pr(belongmeanr5==4), predict(pr outcome(4))
5._predict: Pr(belongmeanr5==5), predict(pr outcome(5))

_predict#year	Margin	Delta-method std. err.	z	P>|z|	[95% conf.	interval]
1 2017	.0312804	.0002743	114.03	0.000	.0307427	.031818
1 2019	.0497981	.000386	129.01	0.000	.0490416	.0505547
1 2021	.057072	.0004325	131.96	0.000	.0562244	.0579197
2 2017	.0754711	.0004873	154.89	0.000	.0745161	.0764261
2 2019	.1119519	.000588	190.38	0.000	.1107993	.1131044
2 2021	.1248899	.0006327	197.39	0.000	.1236498	.12613
3 2017	.3293201	.000984	334.69	0.000	.3273915	.3312486
3 2019	.3910629	.0008603	454.59	0.000	.3893768	.392749
3 2021	.4051008	.0008569	472.76	0.000	.4034213	.4067802
4 2017	.372923	.0009038	412.61	0.000	.3711515	.3746945
4 2019	.3193859	.0008636	369.82	0.000	.3176932	.3210785
4 2021	.300215	.0008466	354.59	0.000	.2985556	.3018744
5 2017	.1910055	.0009955	191.88	0.000	.1890544	.1929566
5 2019	.1278013	.0006316	202.33	0.000	.1265633	.1290393
5 2021	.1127223	.0005783	194.90	0.000	.1115887	.1138558

3. Multivariable regressions with interactions, unstandardized estimates and margins

3.1. Number of close friends

```
. ologit closefriend i.year##i.gender##i.schoollevel i.degree i.urbanrural i.immigrant, vce(robust) nolog

Ordered logistic regression                         Number of obs = 415,932
                                                    Wald chi2(23) = 4882.06
                                                    Prob > chi2   =  0.0000
Log pseudolikelihood = -486421.66                   Pseudo R2     =  0.0049

-----------------------------------------------------------------------------------------------------
                                      |               Robust
                          closefriend | Coefficient  std. err.      z    P>|z|     [95% conf. interval]
--------------------------------------+--------------------------------------------------------------
                                 year |
                                2019  |  -.0113531   .0161277    -0.70   0.481    -.0429627    .0202566
                                2021  |  -.0789721    .015754    -5.01   0.000    -.1098493   -.0480949
                                      |
                               gender |
                                girl  |  -.1231099   .0152898    -8.05   0.000    -.1530774   -.0931425
                                      |
                          year#gender |
                            2019#girl |  -.0399431   .0207575    -1.92   0.054    -.0806271     .000741
                            2021#girl |   -.225964   .0201198   -11.23   0.000    -.2653981     -.18653
                                      |
                          schoollevel |
                      uppersecondary  |  -.0165845   .0211987    -0.78   0.434    -.0581331    .0249642
                          vocational  |   .2101389   .0216445     9.71   0.000     .1677166    .2525613
                                      |
                     year#schoollevel |
                 2019#uppersecondary  |  -.0539498   .0284551    -1.90   0.058    -.1097207    .0018212
                     2019#vocational  |   -.113126   .0307083    -3.68   0.000     -.173313   -.0529389
                 2021#uppersecondary  |  -.0665962   .0278637    -2.39   0.017     -.121208   -.0119845
                     2021#vocational  |  -.0977706   .0306376    -3.19   0.001    -.1578191   -.0377221
                                      |
                   gender#schoollevel |
                 girl#uppersecondary  |    .127211   .0266902     4.77   0.000     .0748991    .1795228
                     girl#vocational  |  -.2474954   .0291776    -8.48   0.000    -.3046824   -.1903083
                                      |
              year#gender#schoollevel |
            2019#girl#uppersecondary  |   .0212318   .0358217     0.59   0.553    -.0489774    .0914409
                2019#girl#vocational  |   .0725614   .0415712     1.75   0.081    -.0089166    .1540395
            2021#girl#uppersecondary  |    .078139   .0348088     2.24   0.025     .0099149     .146363
                2021#girl#vocational  |   .0284179   .0410986     0.69   0.489    -.0521339    .1089696
                                      |
                             1.degree |    .139646    .006113    22.84   0.000     .1276648    .1516273
                                      |
                           urbanrural |
                           semiurban  |  -.0334961     .00797    -4.20   0.000    -.0491171   -.0178752
                               rural  |  -.0884081   .0092667    -9.54   0.000    -.1065704   -.0702458
                                      |
                            immigrant |
              one foreign-born parent  |   -.144607   .0113521   -12.74   0.000    -.1668567   -.1223574
born in Finland foreign-born parents  |  -.2423189   .0213373   -11.36   0.000    -.2841392   -.2004986
     student and parents born abroad  |  -.5147035    .018156   -28.35   0.000    -.5502885   -.4791184
--------------------------------------+--------------------------------------------------------------
                                /cut1 |  -2.560676    .013709                      -2.587545   -2.533807
                                /cut2 |  -1.143717   .0128095                      -1.168824   -1.118611
                                /cut3 |  -.2271271   .0126156                      -.2518532    -.202401
-----------------------------------------------------------------------------------------------------

.
. testparm  year#gender#schoollevel

 ( 1)  [closefriend]2019.year#2.gender#20.schoollevel = 0
 ( 2)  [closefriend]2019.year#2.gender#30.schoollevel = 0
 ( 3)  [closefriend]2021.year#2.gender#20.schoollevel = 0
 ( 4)  [closefriend]2021.year#2.gender#30.schoollevel = 0

           chi2(  4) =    9.25
         Prob > chi2 =    0.0551

.
. ologit closefriend i.year##i.gender i.year##i.schoollevel i.degree i.urbanrural i.immigrant, vce(robust) nolog

Ordered logistic regression                         Number of obs = 415,932
                                                    Wald chi2(17) = 4427.81
                                                    Prob > chi2   =  0.0000
Log pseudolikelihood = -486632.63                   Pseudo R2     =  0.0045

-----------------------------------------------------------------------------------------------------
                                      |               Robust
                          closefriend | Coefficient  std. err.      z    P>|z|     [95% conf. interval]
--------------------------------------+--------------------------------------------------------------
                                 year |
                                2019  |  -.0283939   .0137621    -2.06   0.039    -.0553671   -.0014207
                                2021  |  -.1036531   .0134339    -7.72   0.000    -.1299831   -.0773231
                                      |
                               gender |
                                girl  |  -.1373008   .0112371   -12.22   0.000    -.1593252   -.1152764
                                      |
                           ear#gender |
                            2019#girl |  -.0088963    .015333    -0.58   0.562    -.0389484    .0211557
                            2021#girl |  -.1815634   .0149296   -12.16   0.000    -.2108248    -.152302
                                      |
                          schoollevel |
                      uppersecondary  |    .061818   .0129869     4.76   0.000     .0363641    .0872719
                          vocational  |   .0956443   .0147316     6.49   0.000     .0667708    .1245177
```

```
                                         |
                        year#schoolevel |
                  2019#uppersecondary  |  -.0418511   .0173606    -2.41   0.016    -.0758773    -.007825
                       2019#vocational  |  -.0770158   .0208759    -3.69   0.000    -.1179318   -.0360998
                  2021#uppersecondary  |  -.0173424   .0167904    -1.03   0.302    -.0502509    .0155662
                       2021#vocational  |  -.0844622   .0205879    -4.10   0.000    -.1248138   -.0441106
                                         |
                               1.degree |    .138266    .006107    22.64   0.000     .1262966    .1502354
                                         |
                             urbanrural |
                             semiurban  |  -.0318348   .0079674    -4.00   0.000    -.0474506   -.0162191
                                 rural  |  -.0886349   .0092651    -9.57   0.000    -.1067942   -.0704755
                                         |
                              immigrant |
                 one foreign-born parent  |  -.1455395   .0113468   -12.83   0.000    -.1677788   -.1233003
born in Finland foreign-born parents  |  -.2429328   .0213498   -11.38   0.000    -.2847777   -.2010879
     student and parents born abroad  |  -.5138975   .0181497   -28.31   0.000    -.5494703   -.4783247
-----------------------------------------+----------------------------------------------------------------
                                  /cut1 |  -2.567881   .0122264                     -2.591845   -2.543918
                                  /cut2 |  -1.151461   .0112175                     -1.173447   -1.129475
                                  /cut3 |  -.2356877   .0109943                     -.2572361   -.2141392
----------------------------------------------------------------------------------------------------------

.
. testparm year#gender

 ( 1)  [closefriend]2019.year#2.gender = 0
 ( 2)  [closefriend]2021.year#2.gender = 0

           chi2(  2) =  201.59
         Prob > chi2 =    0.0000

. testparm year#schoollevel

 ( 1)  [closefriend]2019.year#20.schoollevel = 0
 ( 2)  [closefriend]2019.year#30.schoollevel = 0
 ( 3)  [closefriend]2021.year#20.schoollevel = 0
 ( 4)  [closefriend]2021.year#30.schoollevel = 0

           chi2(  4) =   23.63
         Prob > chi2 =    0.0001

.
. margins year#gender

Predictive margins                                     Number of obs = 415,932
Model VCE: Robust

1._predict: Pr(closefriend==1), predict(pr outcome(1))
2._predict: Pr(closefriend==2), predict(pr outcome(2))
3._predict: Pr(closefriend==3), predict(pr outcome(3))
4._predict: Pr(closefriend==4), predict(pr outcome(4))

-----------------------------------------------------------------------------------
                  |            Delta-method
                  |     Margin   std. err.      z    P>|z|     [95% conf. interval]
------------------+----------------------------------------------------------------
_predict#year#gender |
      1#2017#boy  |   .0677726   .0006498   104.30   0.000      .066499    .0690461
     1#2017#girl  |    .076965   .0006014   127.98   0.000     .0757863    .0781437
      1#2019#boy  |   .0711438   .0006461   110.11   0.000     .0698775    .0724102
     1#2019#girl  |   .0814163   .0006005   135.59   0.000     .0802394    .0825932
      1#2021#boy  |   .0758672   .0006601   114.94   0.000     .0745735    .0771608
     1#2021#girl  |   .1014215   .0006745   150.36   0.000     .1000995    .1027435
      2#2017#boy  |   .1622233   .0010751   150.89   0.000     .1601161    .1643305
     2#2017#girl  |   .1781486   .0009661   184.40   0.000      .176255    .1800422
      2#2019#boy  |   .1682482   .0010293   163.45   0.000     .1662307    .1702657
     2#2019#girl  |    .185503    .000935   198.39   0.000     .1836704    .1873357
      2#2021#boy  |   .1763375    .001023   172.37   0.000     .1743324    .1783426
     2#2021#girl  |   .2152316   .0009574   224.80   0.000      .213355    .2171081
      3#2017#boy  |   .1964852   .0007995   245.76   0.000     .1949182    .1980522
     3#2017#girl  |   .2050917   .0007399   277.19   0.000     .2036416    .2065419
      3#2019#boy  |   .1999954   .0007599   263.20   0.000     .1985061    .2014847
     3#2019#girl  |   .2085928   .0007163   291.22   0.000     .2071889    .2099967
      3#2021#boy  |   .2042312   .0007418   275.33   0.000     .2027773     .205685
     3#2021#girl  |   .2190029   .0006949   315.16   0.000      .217641    .2203649
      4#2017#boy  |   .5735189    .002126   269.77   0.000     .5693521    .5776857
     4#2017#girl  |   .5397947   .0017778   303.63   0.000     .5363102    .5432791
      4#2019#boy  |   .5606126   .0019859   282.30   0.000     .5567203    .5645048
     4#2019#girl  |   .5244879   .0016559   316.73   0.000     .5212424    .5277335
      4#2021#boy  |   .5435642    .001925   282.37   0.000     .5397912    .5473371
     4#2021#girl  |    .464344   .0015324   303.02   0.000     .4613406    .4673475
-----------------------------------------------------------------------------------

. margins year#schoollevel

Predictive margins                                     Number of obs = 415,932
Model VCE: Robust

1._predict: Pr(closefriend==1), predict(pr outcome(1))
2._predict: Pr(closefriend==2), predict(pr outcome(2))
3._predict: Pr(closefriend==3), predict(pr outcome(3))
4._predict: Pr(closefriend==4), predict(pr outcome(4))

------------------------------------------------------------------------------------------
                         |            Delta-method
                         |     Margin   std. err.      z    P>|z|     [95% conf. interval]
-------------------------+----------------------------------------------------------------
_predict#year#schoollevel |
   1#2017#lowersecondary  |   .0748509   .0006258   119.61   0.000     .0736244    .0760775
   1#2017#uppersecondary  |   .0706877   .0007715    91.62   0.000     .0691755    .0721998
       1#2017#vocational  |    .068501    .000862    79.47   0.000     .0668115    .0701904
   1#2019#lowersecondary  |     .07719   .0006087   126.82   0.000      .075997     .078383
   1#2019#uppersecondary  |   .0757821   .0007437   101.90   0.000     .0743245    .0772398
       1#2019#vocational  |   .0758758   .0009869    76.88   0.000     .0739415      .07781
   1#2021#lowersecondary  |   .0905563   .0006532   138.64   0.000     .0892761    .0918365
   1#2021#uppersecondary  |    .086972   .0007827   111.12   0.000      .085438    .0885061
       1#2021#vocational  |   .0896429   .0011237    79.77   0.000     .0874404    .0918453
   2#2017#lowersecondary  |   .1745039   .0009971   175.00   0.000     .1725495    .1764583
   2#2017#uppersecondary  |    .167322   .0012976   128.94   0.000     .1647786    .1698653
       2#2017#vocational  |   .1634523    .001502   108.82   0.000     .1605084    .1663963
   2#2019#lowersecondary  |    .178414    .000944   188.99   0.000     .1765638    .1802643
```

```
   2#2019#uppersecondary  |   .1760574   .0011951   147.32   0.000     .1737151    .1783998
       2#2019#vocational  |    .176215   .0016253   108.42   0.000     .1730296    .1794004
   2#2021#lowersecondary  |   .1987139   .0009429   210.74   0.000     .1968658     .200562
   2#2021#uppersecondary  |   .1933165   .0011721   164.93   0.000     .1910192    .1956138
       2#2021#vocational  |   .1973533   .0016708   118.12   0.000     .1940785     .200628
   3#2017#lowersecondary  |   .2031419   .0007478   271.64   0.000     .2016761    .2046076
   3#2017#uppersecondary  |   .1992981   .0008937   223.00   0.000     .1975464    .2010498
       3#2017#vocational  |   .1970907    .001012   194.76   0.000     .1951073    .1990742
   3#2019#lowersecondary  |   .2050699   .0007191   285.16   0.000     .2036604    .2064794
   3#2019#uppersecondary  |   .2039032   .0008119   251.13   0.000     .2023118    .2054946
       3#2019#vocational  |   .2039823   .0009811   207.92   0.000     .2020594    .2059052
   3#2021#lowersecondary  |   .2127545   .0006968   305.31   0.000     .2113887    .2141203
   3#2021#uppersecondary  |   .2107295   .0007551   279.08   0.000     .2092496    .2122095
       3#2021#vocational  |    .212261   .0008589   247.13   0.000     .2105776    .2139444
   4#2017#lowersecondary  |   .5475033   .0018768   291.73   0.000     .5438249    .5511817
   4#2017#uppersecondary  |   .5626923   .0026089   215.68   0.000     .5575789    .5678057
       4#2017#vocational  |    .570956   .0030827   185.21   0.000      .564914     .576998
   4#2019#lowersecondary  |   .5393261   .0017262   312.44   0.000     .5359428    .5427093
   4#2019#uppersecondary  |   .5442572   .0023217   234.42   0.000     .5397067    .5488077
       4#2019#vocational  |   .5439269   .0032682   166.43   0.000     .5375213    .5503326
   4#2021#lowersecondary  |   .4979754   .0016137   308.59   0.000     .4948125    .5011382
   4#2021#uppersecondary  |   .5089819   .0021753   233.98   0.000     .5047183    .5132455
       4#2021#vocational  |   .5007429   .0032325   154.91   0.000     .4944073    .5070784
-----------------------------------------------------------------------------------------

.
. ****

. ologit closefriend i.year##i.schoollevel##i.gender##i.degree i.urbanrural i.immigrant, vce(robust) nolog

Ordered logistic regression                            Number of obs = 415,932
                                                       Wald chi2(40) = 5013.24
                                                       Prob > chi2   =  0.0000
Log pseudolikelihood = -486372.89                      Pseudo R2     =  0.0050

-----------------------------------------------------------------------------------------------------
                                    |               Robust
                        closefriend | Coefficient  std. err.      z    P>|z|     [95% conf. interval]
------------------------------------+----------------------------------------------------------------
                               year |
                               2019 |   .0053531   .0230551     0.23   0.816    -.0398341    .0505403
                               2021 |  -.0592869   .0226658    -2.62   0.009    -.1037111   -.0148627
                                    |
                        schoollevel |
                     uppersecondary |  -.0831491   .0343761    -2.42   0.016     -.150525   -.0157732
                         vocational |   .1646876   .0275404     5.98   0.000     .1107094    .2186658
                                    |
                   year#schoollevel |
                2019#uppersecondary |  -.0126769   .0476073    -0.27   0.790    -.1059855    .0806316
                    2019#vocational |  -.0673363   .0397145    -1.70   0.090    -.1451753    .0105026
                2021#uppersecondary |  -.0994152   .0474762    -2.09   0.036    -.1924668   -.0063636
                    2021#vocational |  -.0795297   .0398664    -1.99   0.046    -.1576663   -.0013931
                                    |
                             gender |
                               girl |  -.1591008   .0210287    -7.57   0.000    -.2003164   -.1178853
                                    |
                        year#gender |
                          2019#girl |  -.0660968    .029341    -2.25   0.024    -.1236041   -.0085895
                          2021#girl |  -.2179338   .0286247    -7.61   0.000    -.2740371   -.1618305
                                    |
                 schoollevel#gender |
                uppersecondary#girl |   .1712017   .0419405     4.08   0.000     .0889999    .2534035
                    vocational#girl |  -.1941238   .0365406    -5.31   0.000    -.2657421   -.1225055
                                    |
            year#schoollevel#gender |
           2019#uppersecondary#girl |  -.0311902   .0578822    -0.54   0.590    -.1446371    .0822567
               2019#vocational#girl |   .0446209   .0527765     0.85   0.398    -.0588192     .148061
           2021#uppersecondary#girl |   .0716649    .057217     1.25   0.210    -.0404784    .1838082
               2021#vocational#girl |   .0137925   .0525469     0.26   0.793    -.0891976    .1167826
                                    |
                           1.degree |   .0922729   .0237865     3.88   0.000     .0456523    .1388936
                                    |
                        year#degree |
                             2019 1 |  -.0269134   .0322763    -0.83   0.404    -.0901737    .0363469
                             2021 1 |  -.0302471    .031558    -0.96   0.338    -.0920996    .0316054
                                    |
                 schoollevel#degree |
                  uppersecondary#1 |   .1138994   .0439954     2.59   0.010       .02767    .2001288
                      vocational#1 |    .116496    .045306     2.57   0.010     .0276979    .2052941
                                    |
            year#schoollevel#degree |
             2019#uppersecondary#1 |  -.0598264   .0598739    -1.00   0.318    -.1771772    .0575244
                 2019#vocational#1 |  -.1495921   .0637687    -2.35   0.019    -.2745763   -.0246078
             2021#uppersecondary#1 |   .0412647   .0591878     0.70   0.486    -.0747412    .1572707
                 2021#vocational#1 |  -.0793886   .0634441    -1.25   0.211    -.2037367    .0449595
                                    |
                      gender#degree |
                             girl#1 |   .0756022    .030635     2.47   0.014     .0155587    .1356457
                                    |
                 year#gender#degree |
                        2019#girl#1 |   .0416802   .0415926     1.00   0.316    -.0398399    .1232003
                        2021#girl#1 |  -.0248032   .0403523    -0.61   0.539    -.1038921    .0542858
                                    |
          schoollevel#gender#degree |
             uppersecondary#girl#1 |  -.0815824     .05492    -1.49   0.137    -.1892235    .0260587
                 vocational#girl#1 |  -.1271137   .0620176    -2.05   0.040     -.248666   -.0055615
                                    |
     year#schoollevel#gender#degree |
        2019#uppersecondary#girl#1 |   .0736419   .0743999     0.99   0.322    -.0721792     .219463
            2019#vocational#girl#1 |   .1068642    .087632     1.22   0.223    -.0648913    .2786197
        2021#uppersecondary#girl#1 |   .0241351    .072872     0.33   0.740    -.1186915    .1669617
            2021#vocational#girl#1 |   .0394002    .086167     0.46   0.647     -.129484    .2082844
                                    |
                         urbanrural |
                          semiurban |  -.0334509   .0079722    -4.20   0.000     -.049076   -.0178258
                              rural |  -.0882167   .0092712    -9.52   0.000    -.1063879   -.0700454
                                    |
                          immigrant |
             one foreign-born parent |  -.1441574   .0113535   -12.70   0.000    -.1664099    -.121905
born in Finland foreign-born parents |  -.2406555   .0213509   -11.27   0.000    -.2825024   -.1988086
     student and parents born abroad |  -.5142355   .0181635   -28.31   0.000    -.5498354   -.4786356
------------------------------------+----------------------------------------------------------------
```

```
                              /cut1 |    -2.5837   .0177385                     -2.618466   -2.548933
                              /cut2 |  -1.166635   .0170368                     -1.200026   -1.133243
                              /cut3 |  -.2498565   .0168858                      -.282952   -.2167609
---------------------------------------------------------------------------------------------------

. testparm year#schoollevel#gender#degree

 ( 1)  [closefriend]2019.year#20.schoollevel#2.gender#1.degree = 0
 ( 2)  [closefriend]2019.year#30.schoollevel#2.gender#1.degree = 0
 ( 3)  [closefriend]2021.year#20.schoollevel#2.gender#1.degree = 0
 ( 4)  [closefriend]2021.year#30.schoollevel#2.gender#1.degree = 0

           chi2(  4) =    2.05
         Prob > chi2 =    0.7260

.
. ologit closefriend i.year##i.schoollevel##i.gender##i.urbanrural i.degree  i.immigrant, vce(robust) nolog

Ordered logistic regression                           Number of obs = 415,932
                                                      Wald chi2(57) = 5036.90
                                                      Prob > chi2   =  0.0000
Log pseudolikelihood = -486350.71                     Pseudo R2     =  0.0051

---------------------------------------------------------------------------------------------------
                                      |               Robust
                          closefriend | Coefficient  std. err.      z    P>|z|     [95% conf. interval]
--------------------------------------+------------------------------------------------------------
                                 year |
                                 2019 |  -.0284094   .0198785    -1.43   0.153    -.0673705    .0105517
                                 2021 |  -.0941486    .019356    -4.86   0.000    -.1320857   -.0562114
                                      |
                          schoollevel |
                       uppersecondary |   -.004517   .0254214    -0.18   0.859    -.0543421     .045308
                           vocational |   .1931016   .0251891     7.67   0.000     .1437318    .2424713
                                      |
                     year#schoollevel |
                  2019#uppersecondary |  -.0352312   .0338689    -1.04   0.298     -.101613    .0311505
                      2019#vocational |  -.0886193   .0356504    -2.49   0.013    -.1584928   -.0187458
                  2021#uppersecondary |  -.0593797   .0331294    -1.79   0.073    -.1243121    .0055527
                      2021#vocational |  -.0816336   .0355088    -2.30   0.022    -.1512296   -.0120377
                                      |
                               gender |
                                 girl |  -.1020884     .01906    -5.36   0.000    -.1394453   -.0647314
                                      |
                          year#gender |
                            2019#girl |  -.0256049    .025649    -1.00   0.318    -.0758759    .0246661
                            2021#girl |  -.2418407   .0248001    -9.75   0.000     -.290448   -.1932334
                                      |
                   schoollevel#gender |
                  uppersecondary#girl |    .135632   .0321672     4.22   0.000     .0725854    .1986787
                      vocational#girl |  -.2220469    .033783    -6.57   0.000    -.2882604   -.1558334
                                      |
              year#schoollevel#gender |
             2019#uppersecondary#girl |   .0022827   .0428338     0.05   0.957      -.08167    .0862354
                 2019#vocational#girl |   .0437575   .0479956     0.91   0.362    -.0503122    .1378272
             2021#uppersecondary#girl |   .0890481   .0415357     2.14   0.032     .0076396    .1704566
                 2021#vocational#girl |   .0313797   .0474075     0.66   0.508    -.0615373    .1242966
                                      |
                           urbanrural |
                            semiurban |  -.0281709   .0309355    -0.91   0.362    -.0888035    .0324617
                                rural |  -.0455706   .0330712    -1.38   0.168    -.1103889    .0192477
                                      |
                      year#urbanrural |
                       2019#semiurban |   .0813376   .0421023     1.93   0.053    -.0011813    .1638566
                           2019#rural |   .0205868    .045824     0.45   0.653    -.0692267    .1104003
                       2021#semiurban |   .0683732   .0413776     1.65   0.098    -.0127254    .1494719
                           2021#rural |   .0276023   .0448777     0.62   0.539    -.0603562    .1155609
                                      |
               schoollevel#urbanrural |
             uppersecondary#semiurban |  -.0291545   .0567032    -0.51   0.607    -.1402908    .0819818
                 uppersecondary#rural |  -.0433116   .0648956    -0.67   0.505    -.1705046    .0838814
                 vocational#semiurban |   .1275173   .0572586     2.23   0.026     .0152924    .2397421
                     vocational#rural |  -.0015458   .0921895    -0.02   0.987     -.182234    .1791424
                                      |
          year#schoollevel#urbanrural |
        2019#uppersecondary#semiurban |  -.0842578    .077192    -1.09   0.275    -.2355514    .0670358
            2019#uppersecondary#rural |  -.0348184   .0891542    -0.39   0.696    -.2095576    .1399207
            2019#vocational#semiurban |  -.1622524   .0814805    -1.99   0.046    -.3219513   -.0025535
                2019#vocational#rural |   .1046314   .1297958     0.81   0.420    -.1497637    .3590265
        2021#uppersecondary#semiurban |   .0180023   .0757568     0.24   0.812    -.1304784    .1664829
            2021#uppersecondary#rural |  -.0848263   .0872955    -0.97   0.331    -.2559224    .0862697
            2021#vocational#semiurban |  -.0842531   .0813752    -1.04   0.300    -.2437456    .0752394
                2021#vocational#rural |  -.0069106   .1299594    -0.05   0.958    -.2616263    .2478051
                                      |
                    gender#urbanrural |
                       girl#semiurban |  -.0447525   .0396819    -1.13   0.259    -.1225277    .0330227
                           girl#rural |  -.0757695   .0422977    -1.79   0.073    -.1586716    .0071325
                                      |
               year#gender#urbanrural |
                  2019#girl#semiurban |  -.0879912   .0540421    -1.63   0.103    -.1939117    .0179294
                      2019#girl#rural |   .0007362   .0588308     0.01   0.990    -.1145701    .1160425
                  2021#girl#semiurban |   .0060003   .0526373     0.11   0.909    -.0971669    .1091674
                      2021#girl#rural |   .0882851   .0571514     1.54   0.122    -.0237295    .2002997
                                      |
        schoollevel#gender#urbanrural |
        uppersecondary#girl#semiurban |   .0259801   .0715955     0.36   0.717    -.1143446    .1663047
            uppersecondary#girl#rural |  -.1379044   .0802957    -1.72   0.086     -.295281    .0194723
            vocational#girl#semiurban |   -.194932   .0802996    -2.43   0.015    -.3523164   -.0375476
                vocational#girl#rural |  -.0938562   .1196464    -0.78   0.433    -.3283589    .1406465
                                      |
   year#schoollevel#gender#urbanrural |
   2019#uppersecondary#girl#semiurban |   .0350877   .0969869     0.36   0.718     -.155003    .2251785
       2019#uppersecondary#girl#rural |   .0858529   .1102257     0.78   0.436    -.1301854    .3018913
       2019#vocational#girl#semiurban |   .1933507   .1139821     1.70   0.090     -.03005     .4167515
           2019#vocational#girl#rural |  -.0450434   .1692899    -0.27   0.790    -.3768456    .2867588
   2021#uppersecondary#girl#semiurban |  -.1709151   .0945699    -1.81   0.071    -.3562686    .0144384
       2021#uppersecondary#girl#rural |   .1550814   .1077029     1.44   0.150    -.0560124    .3661752
       2021#vocational#girl#semiurban |   .1037587    .112136     0.93   0.355    -.1160239    .3235413
           2021#vocational#girl#rural |  -.0989937   .1694425    -0.58   0.559    -.4310948    .2331074
                                      |
                             1.degree |   .1397517   .0061154    22.85   0.000     .1277657    .1517377
                                      |
```

```
                          immigrant |
             one foreign-born parent  |  -.1446228   .0113536   -12.74   0.000    -.1668755   -.1223701
born in Finland foreign-born parents  |   -.242122   .0213556   -11.34   0.000    -.2839781   -.2002659
     student and parents born abroad  |  -.5143817   .0181606   -28.32   0.000    -.5499759   -.4787875
--------------------------------------+----------------------------------------------------------------
                              /cut1 |  -2.552865   .0161181                      -2.584456   -2.521274
                              /cut2 |  -1.135733   .0153595                      -1.165838   -1.105629
                              /cut3 |  -.2188623    .015203                      -.2486596    -.189065
-------------------------------------------------------------------------------------------------------

. testparm year#schoollevel#gender#urbanrural

 ( 1)  [closefriend]2019.year#20.schoollevel#2.gender#2.urbanrural = 0
 ( 2)  [closefriend]2019.year#20.schoollevel#2.gender#3.urbanrural = 0
 ( 3)  [closefriend]2019.year#30.schoollevel#2.gender#2.urbanrural = 0
 ( 4)  [closefriend]2019.year#30.schoollevel#2.gender#3.urbanrural = 0
 ( 5)  [closefriend]2021.year#20.schoollevel#2.gender#2.urbanrural = 0
 ( 6)  [closefriend]2021.year#20.schoollevel#2.gender#3.urbanrural = 0
 ( 7)  [closefriend]2021.year#30.schoollevel#2.gender#2.urbanrural = 0
 ( 8)  [closefriend]2021.year#30.schoollevel#2.gender#3.urbanrural = 0

           chi2(  8) =   13.42
         Prob > chi2 =    0.0981

.
. ologit closefriend i.year##i.schoollevel##i.gender##i.immigrant i.degree i.urbanrural, vce(robust) nolog

Ordered logistic regression                           Number of obs = 415,932
                                                      Wald chi2(74) = 4955.71
                                                      Prob > chi2   =  0.0000
Log pseudolikelihood = -486357                        Pseudo R2     =  0.0050

-----------------------------------------------------------------------------------------------------------------------------
                                                    |               Robust
                                        closefriend | Coefficient  std. err.      z    P>|z|     [95% conf. interval]
----------------------------------------------------+------------------------------------------------------------------------
                                               year |
                                              2019  |  -.0106656   .0172044    -0.62   0.535    -.0443855    .0230544
                                              2021  |  -.0675682   .0168233    -4.02   0.000    -.1005414   -.0345951
                                                    |
                                        schoollevel |
                                    uppersecondary  |  -.0238149   .0224337    -1.06   0.288    -.0677842    .0201544
                                        vocational  |   .2087067   .0228214     9.15   0.000     .1639776    .2534358
                                                    |
                                   year#schoollevel |
                               2019#uppersecondary  |  -.0602646    .030221    -1.99   0.046    -.1194967   -.0010325
                                   2019#vocational  |  -.1140121   .0324732    -3.51   0.000    -.1776583   -.0503658
                               2021#uppersecondary  |  -.0836422   .0296303    -2.82   0.005    -.1417165   -.0255678
                                   2021#vocational  |  -.1050052   .0324309    -3.24   0.001    -.1685687   -.0414417
                                                    |
                                             gender |
                                              girl  |  -.1340054   .0162879    -8.23   0.000    -.1659291   -.1020817
                                                    |
                                        year#gender |
                                         2019#girl  |  -.0415326   .0221362    -1.88   0.061    -.0849187    .0018535
                                         2021#girl  |  -.2404812   .0214863   -11.19   0.000    -.2825935   -.1983688
                                                    |
                                 schoollevel#gender |
                               uppersecondary#girl  |   .1326799   .0282384     4.70   0.000     .0773336    .1880263
                                   vocational#girl  |  -.2414967   .0308558    -7.83   0.000     -.301973   -.1810204
                                                    |
                            year#schoollevel#gender |
                          2019#uppersecondary#girl  |   .0241838   .0380344     0.64   0.525    -.0503624    .0987299
                              2019#vocational#girl  |   .0637836   .0440251     1.45   0.147     -.022504    .1500712
                          2021#uppersecondary#girl  |   .1031856    .037012     2.79   0.005     .0306435    .1757277
                              2021#vocational#girl  |   .0237835   .0435334     0.55   0.585    -.0615404    .1091075
                                                    |
                                          immigrant |
                           one foreign-born parent  |  -.1572161   .0460725    -3.41   0.001    -.2475166   -.0669157
              born in Finland foreign-born parents  |  -.2490027   .0916099    -2.72   0.007    -.4285548   -.0694505
                   student and parents born abroad  |  -.6749757   .0686757    -9.83   0.000    -.8095776   -.5403738
                                                    |
                                     year#immigrant |
                      2019#one foreign-born parent  |  -.0506075    .062516    -0.81   0.418    -.1731366    .0719216
         2019#born in Finland foreign-born parents  |   .0821773   .1210355     0.68   0.497    -.1550478    .3194025
              2019#student and parents born abroad  |   .0294907   .0930731     0.32   0.751    -.1529292    .2119106
                      2021#one foreign-born parent  |  -.1428881    .061294    -2.33   0.020    -.2630221    -.022754
         2021#born in Finland foreign-born parents  |   .0254646   .1150866     0.22   0.825     -.200101    .2510303
              2021#student and parents born abroad  |  -.0337334   .0899281    -0.38   0.708    -.2099892    .1425224
                                                    |
                              schoollevel#immigrant |
            uppersecondary#one foreign-born parent  |  -.0073826   .0838194    -0.09   0.930    -.1716656    .1569004
uppersecondary#born in Finland foreign-born parents  |  -.0396777    .168158    -0.24   0.813    -.3692614     .289906
    uppersecondary#student and parents born abroad  |   .2553222   .1454506     1.76   0.079    -.0297557    .5404002
                vocational#one foreign-born parent  |  -.0376299   .0929752    -0.40   0.686    -.2198579    .1445981
   vocational#born in Finland foreign-born parents  |   .3509565   .1879294     1.87   0.062    -.0173784    .7192913
        vocational#student and parents born abroad  |  -.0842713   .1316491    -0.64   0.522    -.3422988    .1737561
                                                    |
                         year#schoollevel#immigrant |
       2019#uppersecondary#one foreign-born parent  |   .1556873   .1114757     1.40   0.163     -.062801    .3741756
2019#uppersecondary#born in Finland foreign-born parents  |  -.0630148    .214334    -0.29   0.769    -.4831018    .3570722
2019#uppersecondary#student and parents born abroad  |  -.1309042   .1861754    -0.70   0.482    -.4958013    .2339929
           2019#vocational#one foreign-born parent  |    .113635   .1293062     0.88   0.380    -.1398004    .3670704
2019#vocational#born in Finland foreign-born parents  |  -.3291005   .2591304    -1.27   0.204    -.8369866    .1787857
   2019#vocational#student and parents born abroad  |  -.0123521   .1832413    -0.07   0.946    -.3714985    .3467942
       2021#uppersecondary#one foreign-born parent  |   .2306076   .1088868     2.12   0.034     .0171933    .4440219
2021#uppersecondary#born in Finland foreign-born parents  |   .0285199   .2061033     0.14   0.890    -.3754353     .432475
2021#uppersecondary#student and parents born abroad  |  -.0494174   .1807664    -0.27   0.785    -.4037131    .3048783
           2021#vocational#one foreign-born parent  |   .0693202   .1284541     0.54   0.589    -.1824452    .3210857
2021#vocational#born in Finland foreign-born parents  |  -.5242798   .2570605    -2.04   0.041    -1.028109   -.0204504
   2021#vocational#student and parents born abroad  |   .2764441   .1802568     1.53   0.125    -.0768527    .6297409
                                                    |
                                   gender#immigrant |
                      girl#one foreign-born parent  |   .0007663   .0588154     0.01   0.990    -.1145098    .1160423
         girl#born in Finland foreign-born parents  |   .0386748   .1151772     0.34   0.737    -.1870684    .2644179
              girl#student and parents born abroad  |   .2999018   .0928881     3.23   0.001     .1178446     .481959
                                                    |
                              year#gender#immigrant |
                 2019#girl#one foreign-born parent  |   .0976382   .0794857     1.23   0.219     -.058151    .2534273
    2019#girl#born in Finland foreign-born parents  |  -.1284095   .1537092    -0.84   0.403     -.429674    .1728549
         2019#girl#student and parents born abroad  |  -.1008256   .1254706    -0.80   0.422    -.3467434    .1450923
                 2021#girl#one foreign-born parent  |    .190487   .0772626     2.47   0.014     .0390551    .3419188
```

```
             2021#girl#born in Finland foreign-born parents  |  -.0266284   .1440206    -0.18   0.853    -.3089037    .2556468
                  2021#girl#student and parents born abroad  |  -.0069047   .1198662    -0.06   0.954    -.2418381    .2280286
                                                             |
                                schoollevel#gender#immigrant |
                uppersecondary#girl#one foreign-born parent  |   .0475581   .1061359     0.45   0.654    -.1604644    .2555805
   uppersecondary#girl#born in Finland foreign-born parents  |   .0715897    .210935     0.34   0.734    -.3418353    .4850146
        uppersecondary#girl#student and parents born abroad  |  -.2792659   .1817333    -1.54   0.124    -.6354567    .0769249
                    vocational#girl#one foreign-born parent  |   .1639952   .1224242     1.34   0.180    -.0759518    .4039422
       vocational#girl#born in Finland foreign-born parents  |  -.6143569   .2403497    -2.56   0.011    -1.085434   -.1432801
            vocational#girl#student and parents born abroad  |  -.1856099    .177429    -1.05   0.296    -.5333643    .1621445
                                                             |
                           year#schoollevel#gender#immigrant |
           2019#uppersecondary#girl#one foreign-born parent  |   -.166852   .1401614    -1.19   0.234    -.4415632    .1078593
2019#uppersecondary#girl#born in Finland foreign-born parents  |   .0020159   .2700913     0.01   0.994    -.5273535    .5313852
     2019#uppersecondary#girl#student and parents born abroad  |   .2958484   .2344287     1.26   0.207    -.1636235    .7553203
               2019#vocational#girl#one foreign-born parent  |  -.2477314     .17095    -1.45   0.147    -.5827872    .0873244
  2019#vocational#girl#born in Finland foreign-born parents  |   .4175677   .3447061     1.21   0.226    -.2580439    1.093179
       2019#vocational#girl#student and parents born abroad  |   .5468249   .2490656     2.20   0.028     .0586654    1.034984
           2021#uppersecondary#girl#one foreign-born parent  |  -.3102375    .135827    -2.28   0.022    -.5764536   -.0440215
2021#uppersecondary#girl#born in Finland foreign-born parents  |  -.2090237   .2580392    -0.81   0.418    -.7147712    .2967239
     2021#uppersecondary#girl#student and parents born abroad  |   .0860564   .2254167     0.38   0.703    -.3557522    .5278651
               2021#vocational#girl#one foreign-born parent  |   -.126028   .1688843    -0.75   0.456    -.4570351    .2049791
  2021#vocational#girl#born in Finland foreign-born parents  |   .7295326   .3454367     2.11   0.035     .0524892    1.406576
       2021#vocational#girl#student and parents born abroad  |   .1236673    .245222     0.50   0.614    -.3569589    .6042936
                                                             |
                                                    1.degree |    .139827   .0061158    22.86   0.000     .1278403    .1518138
                                                             |
                                                  urbanrural |
                                                  semiurban  |  -.0332131   .0079728    -4.17   0.000    -.0488396   -.0175866
                                                      rural  |  -.0882856    .009269    -9.52   0.000    -.1064526   -.0701187
-------------------------------------------------------------+----------------------------------------------------------------
                                                       /cut1 |  -2.568186   .0143484                      -2.596309   -2.540064
                                                       /cut2 |  -1.150946   .0134971                        -1.1774   -1.124492
                                                       /cut3 |  -.2341976   .0133124                      -.2602893   -.2081059
------------------------------------------------------------------------------------------------------------------------------

. testparm year#schoollevel#gender#immigrant

 ( 1)  [closefriend]2019.year#20.schoollevel#2.gender#2.immigrant = 0
 ( 2)  [closefriend]2019.year#20.schoollevel#2.gender#3.immigrant = 0
 ( 3)  [closefriend]2019.year#20.schoollevel#2.gender#4.immigrant = 0
 ( 4)  [closefriend]2019.year#30.schoollevel#2.gender#2.immigrant = 0
 ( 5)  [closefriend]2019.year#30.schoollevel#2.gender#3.immigrant = 0
 ( 6)  [closefriend]2019.year#30.schoollevel#2.gender#4.immigrant = 0
 ( 7)  [closefriend]2021.year#20.schoollevel#2.gender#2.immigrant = 0
 ( 8)  [closefriend]2021.year#20.schoollevel#2.gender#3.immigrant = 0
 ( 9)  [closefriend]2021.year#20.schoollevel#2.gender#4.immigrant = 0
 (10)  [closefriend]2021.year#30.schoollevel#2.gender#2.immigrant = 0
 (11)  [closefriend]2021.year#30.schoollevel#2.gender#3.immigrant = 0
 (12)  [closefriend]2021.year#30.schoollevel#2.gender#4.immigrant = 0

           chi2( 12) =   19.80
         Prob > chi2 =    0.0710

.
. ologit closefriend i.year##i.schoollevel##i.degree i.year##i.gender##i.degree i.urbanrural i.immigrant, vce(robust) nolog

Ordered logistic regression                             Number of obs = 415,932
                                                        Wald chi2(28) = 4631.86
                                                        Prob > chi2   =  0.0000
Log pseudolikelihood = -486565.56                       Pseudo R2     =  0.0046

------------------------------------------------------------------------------------------
                                      |               Robust
                          closefriend | Coefficient  std. err.      z    P>|z|     [95% conf. interval]
--------------------------------------+---------------------------------------------------
                                 year |
                                 2019 |  -.0024094   .0197261    -0.12   0.903    -.0410719    .0362531
                                 2021 |   -.075619    .019424    -3.89   0.000    -.1136893   -.0375488
                                      |
                          schoollevel |
                       uppersecondary |   .0298011   .0198059     1.50   0.132    -.0090179      .06862
                           vocational |    .071763   .0183351     3.91   0.000     .0358268    .1076992
                                      |
                     year#schoollevel |
                  2019#uppersecondary |  -.0312509   .0272174    -1.15   0.251     -.084596    .0220942
                      2019#vocational |  -.0467492   .0263679    -1.77   0.076    -.0984293    .0049308
                  2021#uppersecondary |  -.0483968   .0266905    -1.81   0.070    -.1007092    .0039156
                      2021#vocational |  -.0743345   .0262031    -2.84   0.005    -.1256917   -.0229774
                                      |
                             1.degree |   .0867594   .0202899     4.28   0.000     .0469919     .126527
                                      |
                          year#degree |
                               2019 1 |  -.0412269   .0275741    -1.50   0.135    -.0952711    .0128173
                               2021 1 |  -.0434279   .0269544    -1.61   0.107    -.0962575    .0094018
                                      |
                   schoollevel#degree |
                     uppersecondary#1 |    .053346   .0263777     2.02   0.043     .0016467    .1050453
                         vocational#1 |    .070953   .0311968     2.27   0.023     .0098083    .1320976
                                      |
              year#schoollevel#degree |
                2019#uppersecondary#1 |  -.0176184   .0355944    -0.49   0.621    -.0873821    .0521453
                    2019#vocational#1 |   -.096954    .044057    -2.20   0.028    -.1833043   -.0106038
                2021#uppersecondary#1 |   .0484171   .0346227     1.40   0.162    -.0194421    .1162764
                    2021#vocational#1 |  -.0595911   .0432188    -1.38   0.168    -.1442984    .0251161
                                      |
                               gender |
                                 girl |  -.1770892   .0156025   -11.35   0.000    -.2076694   -.1465089
                                      |
                          year#gender |
                            2019#girl |  -.0523284    .021954    -2.38   0.017    -.0953574   -.0092994
                            2021#girl |  -.1888702   .0216253    -8.73   0.000    -.2312549   -.1464854
                                      |
                        gender#degree |
                               girl#1 |   .0850391   .0225053     3.78   0.000     .0409295    .1291487
                                      |
                   year#gender#degree |
                          2019#girl#1 |   .0685302   .0307693     2.23   0.026     .0082234    .1288369
                          2021#girl#1 |  -.0008679   .0300287    -0.03   0.977    -.0597232    .0579873
                                      |
                           urbanrural |
                            semiurban |   -.032161   .0079696    -4.04   0.000    -.0477811   -.0165408
```

```
                              rural  |  -.0889275   .0092698    -9.59   0.000    -.1070959    -.070759
                                     |
                          immigrant  |
              one foreign-born parent  |  -.1450952   .0113485   -12.79   0.000    -.1673378   -.1228526
born in Finland foreign-born parents  |  -.2415433    .021362   -11.31   0.000    -.2834121   -.1996745
     student and parents born abroad  |  -.5140037   .0181593   -28.31   0.000    -.5495952   -.4784122
-------------------------------------+----------------------------------------------------------------
                               /cut1 |  -2.592832   .0154502                     -2.623114    -2.56255
                               /cut2 |  -1.176253   .0146518                      -1.20497   -1.147536
                               /cut3 |  -.2601979   .0144751                     -.2885685   -.2318273
------------------------------------------------------------------------------------------------------

. testparm  year#gender#degree

 ( 1)  [closefriend]2019.year#2.gender#1.degree = 0
 ( 2)  [closefriend]2021.year#2.gender#1.degree = 0

           chi2(  2) =    7.19
         Prob > chi2 =    0.0274

. testparm  year#schoollevel#degree

 ( 1)  [closefriend]2019.year#20.schoollevel#1.degree = 0
 ( 2)  [closefriend]2019.year#30.schoollevel#1.degree = 0
 ( 3)  [closefriend]2021.year#20.schoollevel#1.degree = 0
 ( 4)  [closefriend]2021.year#30.schoollevel#1.degree = 0

           chi2(  4) =    9.64
         Prob > chi2 =    0.0469

.
. ologit closefriend i.year##i.schoollevel##i.urbanrural i.year##i.gender##i.urbanrural i.degree i.immigrant, vce(robust) nolog

Ordered logistic regression                           Number of obs = 415,932
                                                      Wald chi2(39) = 4556.51
                                                      Prob > chi2   =  0.0000
Log pseudolikelihood = -486574.45                     Pseudo R2     =  0.0046

------------------------------------------------------------------------------------------------------
                                     |               Robust
                         closefriend | Coefficient  std. err.      z    P>|z|     [95% conf. interval]
-------------------------------------+----------------------------------------------------------------
                                year |
                               2019  |  -.0417375   .0167349    -2.49   0.013    -.0745372   -.0089377
                               2021  |  -.1235088   .0162812    -7.59   0.000    -.1554194   -.0915982
                                     |
                         schoollevel |
                     uppersecondary  |    .078604    .015666     5.02   0.000     .0478993    .1093087
                         vocational  |   .0882788   .0169696     5.20   0.000     .0550189    .1215386
                                     |
                    year#schoollevel |
                2019#uppersecondary  |  -.0343579   .0208098    -1.65   0.099    -.0751442    .0064285
                    2019#vocational  |  -.0656881   .0240558    -2.73   0.006    -.1128366   -.0185396
                2021#uppersecondary  |  -.0030653    .020077    -0.15   0.879    -.0424156    .0362849
                    2021#vocational  |  -.0651943   .0237025    -2.75   0.006    -.1116504   -.0187382
                                     |
                          urbanrural |
                          semiurban  |  -.0193696   .0267063    -0.73   0.468    -.0717129    .0329737
                              rural  |  -.0391203   .0293488    -1.33   0.183    -.0966428    .0184022
                                     |
                     year#urbanrural |
                     2019#semiurban  |   .0633955   .0363515     1.74   0.081    -.0078522    .1346432
                         2019#rural  |    .020842   .0406208     0.51   0.608    -.0587733    .1004574
                     2021#semiurban  |    .091057   .0357039     2.55   0.011     .0210785    .1610354
                         2021#rural  |   .0239436   .0397581     0.60   0.547    -.0539809     .101868
                                     |
              schoollevel#urbanrural |
           uppersecondary#semiurban  |  -.0134103   .0347852    -0.39   0.700    -.0815881    .0547675
               uppersecondary#rural  |  -.1258222   .0384328    -3.27   0.001    -.2011492   -.0504953
               vocational#semiurban  |   .0641886   .0405204     1.58   0.113      -.01523    .1436072
                   vocational#rural  |  -.0553673    .059671    -0.93   0.353    -.1723203    .0615856
                                     |
         year#schoollevel#urbanrural |
      2019#uppersecondary#semiurban  |  -.0638036    .046986    -1.36   0.174    -.1558943    .0282872
          2019#uppersecondary#rural  |   .0183768   .0527181     0.35   0.727    -.0849489    .1217024
          2019#vocational#semiurban  |  -.0843379   .0575231    -1.47   0.143    -.1970811    .0284053
              2019#vocational#rural  |   .0780683   .0842484     0.93   0.354    -.0870554     .243192
      2021#uppersecondary#semiurban  |  -.0871334   .0456124    -1.91   0.056    -.1765321    .0022653
          2021#uppersecondary#rural  |   .0090005   .0514757     0.17   0.861    -.0918901    .1098911
          2021#vocational#semiurban  |  -.0549859   .0565106    -0.97   0.331    -.1657447     .055773
              2021#vocational#rural  |  -.0650244   .0840804    -0.77   0.439     -.229819    .0997702
                                     |
                              gender |
                               girl  |  -.1143929   .0135038    -8.47   0.000    -.1408599   -.0879259
                                     |
                         year#gender |
                          2019#girl  |  -.0012927   .0183366    -0.07   0.944    -.0372318    .0346463
                          2021#girl  |  -.1890906    .017828   -10.61   0.000    -.2240328   -.1541485
                                     |
                   gender#urbanrural |
                     girl#semiurban  |  -.0609897   .0299682    -2.04   0.042    -.1197263    -.002253
                         girl#rural  |  -.0880997   .0338748    -2.60   0.009    -.1544932   -.0217063
                                     |
              year#gender#urbanrural |
                2019#girl#semiurban  |  -.0557925   .0410345    -1.36   0.174    -.1362186    .0246337
                    2019#girl#rural  |   .0003624   .0470847     0.01   0.994    -.0919219    .0926466
                2021#girl#semiurban  |  -.0338323   .0400506    -0.84   0.398      -.11233    .0446654
                    2021#girl#rural  |   .0953895   .0459767     2.07   0.038     .0052769    .1855021
                                     |
                            1.degree |   .1383307   .0061092    22.64   0.000      .126357    .1503044
                                     |
                           immigrant |
             one foreign-born parent  |  -.1456226   .0113479   -12.83   0.000     -.167864   -.1233811
born in Finland foreign-born parents  |  -.2427401   .0213657   -11.36   0.000     -.284616   -.2008641
     student and parents born abroad  |  -.5137997   .0181532   -28.30   0.000    -.5493793   -.4782201
-------------------------------------+----------------------------------------------------------------
                               /cut1 |  -2.559362   .0139734                      -2.58675   -2.531975
                               /cut2 |  -1.142808   .0131029                     -1.168489   -1.117127
                               /cut3 |  -.2268004   .0129172                     -.2521177   -.2014831
------------------------------------------------------------------------------------------------------

. testparm  year#gender#urbanrural
```

```
 ( 1)  [closefriend]2019.year#2.gender#2.urbanrural = 0
 ( 2)  [closefriend]2019.year#2.gender#3.urbanrural = 0
 ( 3)  [closefriend]2021.year#2.gender#2.urbanrural = 0
 ( 4)  [closefriend]2021.year#2.gender#3.urbanrural = 0

           chi2(  4) =    8.20
         Prob > chi2 =    0.0845

. testparm  year#schoollevel#urbanrural

 ( 1)  [closefriend]2019.year#20.schoollevel#2.urbanrural = 0
 ( 2)  [closefriend]2019.year#20.schoollevel#3.urbanrural = 0
 ( 3)  [closefriend]2019.year#30.schoollevel#2.urbanrural = 0
 ( 4)  [closefriend]2019.year#30.schoollevel#3.urbanrural = 0
 ( 5)  [closefriend]2021.year#20.schoollevel#2.urbanrural = 0
 ( 6)  [closefriend]2021.year#20.schoollevel#3.urbanrural = 0
 ( 7)  [closefriend]2021.year#30.schoollevel#2.urbanrural = 0
 ( 8)  [closefriend]2021.year#30.schoollevel#3.urbanrural = 0

           chi2(  8) =    8.74
         Prob > chi2 =    0.3647

.
. ologit closefriend i.year##i.schoollevel##i.immigrant i.year##i.gender##i.immigrant i.degree i.urbanrural, vce(robust) nolog

Ordered logistic regression                           Number of obs = 415,932
                                                      Wald chi2(50) = 4464.76
                                                      Prob > chi2   =  0.0000
Log pseudolikelihood = -486586.96                     Pseudo R2     =  0.0046

-------------------------------------------------------------------------------------------------------------------------
                                                         |               Robust
                                             closefriend | Coefficient  std. err.      z    P>|z|     [95% conf. interval]
---------------------------------------------------------+---------------------------------------------------------------
                                                    year |
                                                    2019 |  -.0273423   .0146526    -1.87   0.062    -.0560608    .0013762
                                                    2021 |  -.0958405   .0143137    -6.70   0.000    -.1238949   -.0677861
                                                         |
                                             schoollevel |
                                         uppersecondary  |   .0579672   .0137346     4.22   0.000     .0310478    .0848866
                                             vocational  |   .0978572   .0155716     6.28   0.000     .0673375     .128377
                                                         |
                                        year#schoollevel |
                                    2019#uppersecondary  |   -.046356   .0184297    -2.52   0.012    -.0824775   -.0102345
                                        2019#vocational  |  -.0828103   .0221014    -3.75   0.000    -.1261283   -.0394923
                                    2021#uppersecondary  |  -.0190325   .0178544    -1.07   0.286    -.0540263    .0159614
                                        2021#vocational  |  -.0938392   .0217992    -4.30   0.000    -.1365648   -.0511136
                                                         |
                                               immigrant |
                                 one foreign-born parent |  -.1823165   .0395848    -4.61   0.000    -.2599013   -.1047318
                    born in Finland foreign-born parents |  -.2013207   .0792028    -2.54   0.011    -.3565553   -.0460862
                         student and parents born abroad |  -.6277275    .060697   -10.34   0.000    -.7466915   -.5087635
                                                         |
                                          year#immigrant |
                            2019#one foreign-born parent |  -.0010781    .053628    -0.02   0.984     -.106187    .1040308
               2019#born in Finland foreign-born parents |   .0277433   .1043039     0.27   0.790    -.1766886    .2321753
                    2019#student and parents born abroad |   -.042209   .0818062    -0.52   0.606    -.2025462    .1181282
                            2021#one foreign-born parent |  -.0782278   .0524444    -1.49   0.136     -.181017    .0245614
               2021#born in Finland foreign-born parents |  -.0119201   .0994713    -0.12   0.905    -.2068803    .1830401
                    2021#student and parents born abroad |  -.0457335   .0790821    -0.58   0.563    -.2007315    .1092645
                                                         |
                                   schoollevel#immigrant |
                  uppersecondary#one foreign-born parent |    .017265   .0516008     0.33   0.738    -.0838707    .1184007
     uppersecondary#born in Finland foreign-born parents |   .0069848   .1019362     0.07   0.945    -.1928064    .2067761
          uppersecondary#student and parents born abroad |   .1017793   .0881366     1.15   0.248    -.0709652    .2745238
                      vocational#one foreign-born parent |    .036467   .0609766     0.60   0.550     -.083045    .1559789
         vocational#born in Finland foreign-born parents |   .0098708   .1183863     0.08   0.934    -.2221622    .2419037
              vocational#student and parents born abroad |  -.1635248   .0905743    -1.81   0.071    -.3410472    .0139976
                                                         |
                              year#schoollevel#immigrant |
             2019#uppersecondary#one foreign-born parent |   .0595317   .0678003     0.88   0.380    -.0733544    .1924177
2019#uppersecondary#born in Finland foreign-born parents |  -.0664574   .1308967    -0.51   0.612    -.3230102    .1900955
     2019#uppersecondary#student and parents born abroad |   .0225762    .114049     0.20   0.843    -.2009558    .2461082
                 2019#vocational#one foreign-born parent |  -.0066644    .085435    -0.08   0.938    -.1741138     .160785
    2019#vocational#born in Finland foreign-born parents |  -.0637499   .1718078    -0.37   0.711    -.4004869    .2729872
         2019#vocational#student and parents born abroad |   .2284668   .1266482     1.80   0.071    -.0197592    .4766927
             2021#uppersecondary#one foreign-born parent |   .0442152   .0652869     0.68   0.498    -.0837448    .1721753
2021#uppersecondary#born in Finland foreign-born parents |  -.1051407   .1245486    -0.84   0.399    -.3492515    .1389701
     2021#uppersecondary#student and parents born abroad |  -.0128161   .1091522    -0.12   0.907    -.2267506    .2011183
                 2021#vocational#one foreign-born parent |   .0025395   .0840938     0.03   0.976    -.1622813    .1673603
    2021#vocational#born in Finland foreign-born parents |  -.1259062   .1715914    -0.73   0.463    -.4622192    .2104068
         2021#vocational#student and parents born abroad |   .3282157   .1244794     2.64   0.008     .0842405     .572191
                                                         |
                                                  gender |
                                                    girl |  -.1457115   .0119165   -12.23   0.000    -.1690674   -.1223555
                                                         |
                                             year#gender |
                                               2019#girl |  -.0111829   .0162986    -0.69   0.493    -.0431277    .0207618
                                               2021#girl |  -.1898435   .0158924   -11.95   0.000    -.2209921   -.1586949
                                                         |
                                        gender#immigrant |
                            girl#one foreign-born parent |   .0458504   .0445928     1.03   0.304    -.0415498    .1332505
               girl#born in Finland foreign-born parents |  -.0426082    .088076    -0.48   0.629    -.2152339    .1300175
                    girl#student and parents born abroad |   .1960313   .0707092     2.77   0.006     .0574439    .3346187
                                                         |
                                   year#gender#immigrant |
                       2019#girl#one foreign-born parent |   .0098544   .0600811     0.16   0.870    -.1079024    .1276111
          2019#girl#born in Finland foreign-born parents |  -.0355353    .116728    -0.30   0.761    -.2643181    .1932474
               2019#girl#student and parents born abroad |   .0576924    .094874     0.61   0.543    -.1282572     .243642
                       2021#girl#one foreign-born parent |   .0767652   .0584212     1.31   0.189    -.0377382    .1912685
          2021#girl#born in Finland foreign-born parents |   .0360014    .110885     0.32   0.745    -.1813292    .2533321
               2021#girl#student and parents born abroad |   .0314647   .0912808     0.34   0.730    -.1474424    .2103718
                                                         |
                                                1.degree |   .1383943   .0061093    22.65   0.000     .1264202    .1503684
                                                         |
                                              urbanrural |
                                               semiurban |  -.0315739   .0079695    -3.96   0.000    -.0471938    -.015954
                                                   rural |  -.0884272   .0092678    -9.54   0.000    -.1065917   -.0702628
---------------------------------------------------------+---------------------------------------------------------------
                                                   /cut1 |  -2.573872   .0127108                      -2.598785    -2.54896
                                                   /cut2 |  -1.157238   .0117524                      -1.180272   -1.134203
```

```
                                          /cut3 |  -.2413666   .0115393                    -.2639832     -.21875
------------------------------------------------------------------------------------------------------------------------

. testparm  year#gender#immigrant

 ( 1)  [closefriend]2019.year#2.gender#2.immigrant = 0
 ( 2)  [closefriend]2019.year#2.gender#3.immigrant = 0
 ( 3)  [closefriend]2019.year#2.gender#4.immigrant = 0
 ( 4)  [closefriend]2021.year#2.gender#2.immigrant = 0
 ( 5)  [closefriend]2021.year#2.gender#3.immigrant = 0
 ( 6)  [closefriend]2021.year#2.gender#4.immigrant = 0

           chi2(  6) =    3.03
         Prob > chi2 =    0.8053

. testparm  year#schoollevel#immigrant

 ( 1)  [closefriend]2019.year#20.schoollevel#2.immigrant = 0
 ( 2)  [closefriend]2019.year#20.schoollevel#3.immigrant = 0
 ( 3)  [closefriend]2019.year#20.schoollevel#4.immigrant = 0
 ( 4)  [closefriend]2019.year#30.schoollevel#2.immigrant = 0
 ( 5)  [closefriend]2019.year#30.schoollevel#3.immigrant = 0
 ( 6)  [closefriend]2019.year#30.schoollevel#4.immigrant = 0
 ( 7)  [closefriend]2021.year#20.schoollevel#2.immigrant = 0
 ( 8)  [closefriend]2021.year#20.schoollevel#3.immigrant = 0
 ( 9)  [closefriend]2021.year#20.schoollevel#4.immigrant = 0
 (10)  [closefriend]2021.year#30.schoollevel#2.immigrant = 0
 (11)  [closefriend]2021.year#30.schoollevel#3.immigrant = 0
 (12)  [closefriend]2021.year#30.schoollevel#4.immigrant = 0

           chi2( 12) =   10.08
         Prob > chi2 =    0.6090

.
. ologit closefriend i.year##i.urbanrural i.year##i.immigrant i.degree i.schoollevel i.gender, vce(robust) nolog

Ordered logistic regression                         Number of obs = 415,932
                                                    Wald chi2(21) = 4055.45
                                                    Prob > chi2   =  0.0000
Log pseudolikelihood = -486733.61                   Pseudo R2     =  0.0043

---------------------------------------------------------------------------------------------------------
                                        |               Robust
                            closefriend | Coefficient  std. err.      z    P>|z|     [95% conf. interval]
----------------------------------------+----------------------------------------------------------------
                                   year |
                                  2019  |  -.0685377   .0095274    -7.19   0.000     -.087211   -.0498644
                                  2021  |  -.2424813   .0092455   -26.23   0.000    -.2606021   -.2243606
                                        |
                             urbanrural |
                             semiurban  |  -.0502624   .0147051    -3.42   0.001    -.0790839    -.021441
                                 rural  |  -.1323705   .0166341    -7.96   0.000    -.1649728   -.0997682
                                        |
                        year#urbanrural |
                        2019#semiurban  |   .0068498   .0200879     0.34   0.733    -.0325217    .0462213
                            2019#rural  |      .0398   .0229686     1.73   0.083    -.0052176    .0848175
                        2021#semiurban  |    .041818   .0195265     2.14   0.032     .0035469    .0800892
                            2021#rural  |   .0814358    .022372     3.64   0.000     .0375876     .125284
                                        |
                              immigrant |
               one foreign-born parent  |  -.1501186   .0218428    -6.87   0.000    -.1929296   -.1073075
     born in Finland foreign-born parents  |  -.2295442   .0427293    -5.37   0.000    -.3132921   -.1457963
          student and parents born abroad  |  -.5544779   .0353728   -15.68   0.000    -.6238072   -.4851485
                                        |
                         year#immigrant |
          2019#one foreign-born parent  |   .0270968   .0293059     0.92   0.355    -.0303417    .0845353
2019#born in Finland foreign-born parents  |  -.0137503   .0568519    -0.24   0.809    -.1251779    .0976774
     2019#student and parents born abroad  |   .0536005   .0473821     1.13   0.258    -.0392666    .1464677
          2021#one foreign-born parent  |  -.0137884   .0283451    -0.49   0.627    -.0693439     .041767
2021#born in Finland foreign-born parents  |  -.0194796   .0537515    -0.36   0.717    -.1248307    .0858714
     2021#student and parents born abroad  |   .0562826   .0454138     1.24   0.215    -.0327269    .1452921
                                        |
                               1.degree |   .1380851   .0061072    22.61   0.000     .1261151     .150055
                                        |
                            schoollevel |
                        uppersecondary  |   .0403719   .0067794     5.96   0.000     .0270846    .0536592
                            vocational  |   .0396896   .0086052     4.61   0.000     .0228238    .0565554
                                        |
                                 gender |
                                  girl  |  -.2080877   .0060661   -34.30   0.000    -.2199771   -.1961984
----------------------------------------+----------------------------------------------------------------
                                  /cut1 |  -2.633119   .0106167                     -2.653927   -2.612311
                                  /cut2 |  -1.217089   .0094084                     -1.235529   -1.198649
                                  /cut3 |  -.3017671   .0091296                     -.3196609   -.2838733
---------------------------------------------------------------------------------------------------------

. testparm  year#urbanrural

 ( 1)  [closefriend]2019.year#2.urbanrural = 0
 ( 2)  [closefriend]2019.year#3.urbanrural = 0
 ( 3)  [closefriend]2021.year#2.urbanrural = 0
 ( 4)  [closefriend]2021.year#3.urbanrural = 0

           chi2(  4) =   16.58
         Prob > chi2 =    0.0023

. testparm  year#immigrant

 ( 1)  [closefriend]2019.year#2.immigrant = 0
 ( 2)  [closefriend]2019.year#3.immigrant = 0
 ( 3)  [closefriend]2019.year#4.immigrant = 0
 ( 4)  [closefriend]2021.year#2.immigrant = 0
 ( 5)  [closefriend]2021.year#3.immigrant = 0
 ( 6)  [closefriend]2021.year#4.immigrant = 0

           chi2(  6) =    4.34
         Prob > chi2 =    0.6308

.
. ****final model*****

. ologit closefriend i.year##i.schoollevel##i.degree i.year##i.gender##i.degree i.year##i.urbanrural i.immigrant, vce(robust) nolog
```

```
Ordered logistic regression                          Number of obs = 415,932
                                                     Wald chi2(32) = 4641.80
                                                     Prob > chi2   =  0.0000
Log pseudolikelihood = -486559.68                    Pseudo R2     =  0.0046

------------------------------------------------------------------------------------------------------
                                      |               Robust
                          closefriend | Coefficient  std. err.      z    P>|z|     [95% conf. interval]
--------------------------------------+---------------------------------------------------------------
                                 year |
                                 2019  |  -.0075677   .0210131    -0.36   0.719    -.0487526    .0336172
                                 2021  |   -.096368   .0206123    -4.68   0.000    -.1367674   -.0559687
                                      |
                          schoollevel |
                      uppersecondary  |   .0283845   .0198117     1.43   0.152    -.0104457    .0672147
                          vocational  |   .0660981   .0184927     3.57   0.000     .0298531    .1023431
                                      |
                     year#schoollevel |
                 2019#uppersecondary  |  -.0303997   .0272321    -1.12   0.264    -.0837737    .0229744
                     2019#vocational  |  -.0426343   .0266115    -1.60   0.109     -.094792    .0095234
                 2021#uppersecondary  |  -.0453403   .0267034    -1.70   0.090     -.097678    .0069973
                     2021#vocational  |  -.0637704   .0264262    -2.41   0.016    -.1155649    -.011976
                                      |
                             1.degree |   .0830313   .0203653     4.08   0.000     .0431161    .1229465
                                      |
                          year#degree |
                              2019 1  |  -.0390669   .0277174    -1.41   0.159     -.093392    .0152583
                              2021 1  |  -.0355742   .0270746    -1.31   0.189    -.0886394    .0174909
                                      |
                   schoollevel#degree |
                    uppersecondary#1  |   .0535405   .0263775     2.03   0.042     .0018415    .1052394
                        vocational#1  |   .0736907   .0312245     2.36   0.018     .0124918    .1348896
                                      |
              year#schoollevel#degree |
               2019#uppersecondary#1  |  -.0177577   .0355944    -0.50   0.618    -.0875215    .0520061
                   2019#vocational#1  |  -.0990492   .0440971    -2.25   0.025     -.185478   -.0126205
               2021#uppersecondary#1  |   .0480867   .0346222     1.39   0.165    -.0197716    .1159451
                   2021#vocational#1  |  -.0645351   .0432533    -1.49   0.136      -.14931    .0202397
                                      |
                               gender |
                                girl  |  -.1774469   .0156063   -11.37   0.000    -.2080347    -.146859
                                      |
                          year#gender |
                           2019#girl  |  -.0521759   .0219588    -2.38   0.017    -.0952143   -.0091374
                           2021#girl  |   -.188199   .0216272    -8.70   0.000    -.2305875   -.1458105
                                      |
                        gender#degree |
                              girl#1  |    .085323   .0225083     3.79   0.000     .0412075    .1294385
                                      |
                   year#gender#degree |
                         2019#girl#1  |   .0683691    .030773     2.22   0.026     .0080551    .1286831
                         2021#girl#1  |  -.0013818   .0300301    -0.05   0.963    -.0602397    .0574761
                                      |
                           urbanrural |
                           semiurban  |   -.043774    .014742    -2.97   0.003    -.0726678   -.0148802
                               rural  |  -.1225971   .0168289    -7.28   0.000    -.1555811   -.0896131
                                      |
                      year#urbanrural |
                      2019#semiurban  |  -.0014675    .020168    -0.07   0.942    -.0409961    .0380611
                          2019#rural  |   .0256763   .0232911     1.10   0.270    -.0199735    .0713261
                      2021#semiurban  |   .0324364   .0195899     1.66   0.098    -.0059591    .0708319
                          2021#rural  |   .0685099   .0226757     3.02   0.003     .0240663    .1129535
                                      |
                            immigrant |
             one foreign-born parent  |  -.1450413   .0113482   -12.78   0.000    -.1672834   -.1227993
born in Finland foreign-born parents  |  -.2411996   .0213641   -11.29   0.000    -.2830725   -.1993267
     student and parents born abroad  |  -.5139689   .0181593   -28.30   0.000    -.5495604   -.4783774
--------------------------------------+---------------------------------------------------------------
                                /cut1 |  -2.602482   .0161065                      -2.63405   -2.570914
                                /cut2 |  -1.185894   .0153389                     -1.215958   -1.155831
                                /cut3 |  -.2698157    .015169                     -.2995464    -.240085
------------------------------------------------------------------------------------------------------

. testparm  year#schoollevel#degree

 ( 1)  [closefriend]2019.year#20.schoollevel#1.degree = 0
 ( 2)  [closefriend]2019.year#30.schoollevel#1.degree = 0
 ( 3)  [closefriend]2021.year#20.schoollevel#1.degree = 0
 ( 4)  [closefriend]2021.year#30.schoollevel#1.degree = 0

           chi2(  4) =    9.99
         Prob > chi2 =    0.0405

. testparm  year#gender#degree

 ( 1)  [closefriend]2019.year#2.gender#1.degree = 0
 ( 2)  [closefriend]2021.year#2.gender#1.degree = 0

           chi2(  2) =    7.22
         Prob > chi2 =    0.0270

. testparm  year#urbanrural

 ( 1)  [closefriend]2019.year#2.urbanrural = 0
 ( 2)  [closefriend]2019.year#3.urbanrural = 0
 ( 3)  [closefriend]2021.year#2.urbanrural = 0
 ( 4)  [closefriend]2021.year#3.urbanrural = 0

           chi2(  4) =   11.92
         Prob > chi2 =    0.0179

.
. margins year#schoollevel#degree

Predictive margins                                   Number of obs = 415,932
Model VCE: Robust

1._predict: Pr(closefriend==1), predict(pr outcome(1))
2._predict: Pr(closefriend==2), predict(pr outcome(2))
3._predict: Pr(closefriend==3), predict(pr outcome(3))
4._predict: Pr(closefriend==4), predict(pr outcome(4))
```

_predict#year#schoollevel#degree	Margin	Delta-method std. err.	z	P>|z|	[95% conf.	interval]
1#2017#lowersecondary#0	.0796087	.0008412	94.63	0.000	.07796	.0812575
1#2017#lowersecondary#1	.0705658	.0007971	88.53	0.000	.0690035	.0721282
1#2017#uppersecondary#0	.0775568	.0012685	61.14	0.000	.0750706	.0800431
1#2017#uppersecondary#1	.0653833	.0008863	73.77	0.000	.0636461	.0671205
1#2017#vocational#0	.0749054	.0010939	68.47	0.000	.0727613	.0770494
1#2017#vocational#1	.0619383	.0013519	45.81	0.000	.0592886	.064588
1#2019#lowersecondary#0	.0821547	.0008462	97.09	0.000	.0804962	.0838132
1#2019#lowersecondary#1	.072878	.0007323	99.51	0.000	.0714426	.0743133
1#2019#uppersecondary#0	.0823065	.0012493	65.88	0.000	.0798578	.0847551
1#2019#uppersecondary#1	.0706313	.0008312	84.97	0.000	.0690021	.0722605
1#2019#vocational#0	.0804061	.0012421	64.73	0.000	.0779715	.0828406
1#2019#vocational#1	.073006	.0015731	46.41	0.000	.0699229	.0760891
1#2021#lowersecondary#0	.0948209	.000904	104.89	0.000	.0930491	.0965927
1#2021#lowersecondary#1	.0868186	.0007835	110.81	0.000	.085283	.0883542
1#2021#uppersecondary#0	.0962805	.0013807	69.73	0.000	.0935744	.0989867
1#2021#uppersecondary#1	.0803518	.0008516	94.36	0.000	.0786828	.0820208
1#2021#vocational#0	.094622	.0014357	65.91	0.000	.0918082	.0974359
1#2021#vocational#1	.0859148	.001736	49.49	0.000	.0825123	.0893173
2#2017#lowersecondary#0	.1825281	.0013397	136.25	0.000	.1799024	.1851539
2#2017#lowersecondary#1	.167369	.0013459	124.36	0.000	.1647311	.1700069
2#2017#uppersecondary#0	.1791478	.0020838	85.97	0.000	.1750636	.183232
2#2017#uppersecondary#1	.158054	.0015744	100.39	0.000	.1549683	.1611397
2#2017#vocational#0	.1746962	.00183	95.46	0.000	.1711094	.178283
2#2017#vocational#1	.1516442	.0025233	60.10	0.000	.1466988	.1565897
2#2019#lowersecondary#0	.1864769	.001318	141.48	0.000	.1838936	.1890601
2#2019#lowersecondary#1	.1714371	.0011996	142.91	0.000	.1690858	.1737883
2#2019#uppersecondary#0	.1867193	.0019753	94.53	0.000	.1828478	.1905907
2#2019#uppersecondary#1	.1675184	.0014064	119.11	0.000	.1647619	.170275
2#2019#vocational#0	.1836625	.0019889	92.35	0.000	.1797644	.1875606
2#2019#vocational#1	.1716582	.0026908	63.79	0.000	.1663843	.1769321
2#2021#lowersecondary#0	.2049037	.0012958	158.14	0.000	.202364	.2074433
2#2021#lowersecondary#1	.1935604	.0011702	165.41	0.000	.1912669	.1958539
2#2021#uppersecondary#0	.2069824	.0019697	105.08	0.000	.2031218	.210843
2#2021#uppersecondary#1	.1833638	.0013454	136.29	0.000	.1807268	.1860008
2#2021#vocational#0	.2046186	.002061	99.28	0.000	.2005791	.2086581
2#2021#vocational#1	.1921672	.0026697	71.98	0.000	.1869346	.1973998
3#2017#lowersecondary#0	.207205	.000848	244.33	0.000	.2055429	.2088672
3#2017#lowersecondary#1	.1997122	.0009165	217.91	0.000	.1979159	.2015085
3#2017#uppersecondary#0	.2056067	.0011596	177.31	0.000	.203334	.2078794
3#2017#uppersecondary#1	.1942245	.0010939	177.55	0.000	.1920804	.1963685
3#2017#vocational#0	.2033921	.0010811	188.14	0.000	.2012732	.205511
3#2017#vocational#1	.1901206	.0017301	109.89	0.000	.1867297	.1935116
3#2019#lowersecondary#0	.2087664	.0008209	254.31	0.000	.2071574	.2103753
3#2019#lowersecondary#1	.2019833	.0008323	242.68	0.000	.200352	.2036146
3#2019#uppersecondary#0	.2088721	.0010482	199.27	0.000	.2068176	.2109265
3#2019#uppersecondary#1	.1998443	.0009414	212.28	0.000	.1979992	.2016894
3#2019#vocational#0	.2075123	.0010676	194.37	0.000	.2054199	.2096047
3#2019#vocational#1	.2021011	.0015252	132.51	0.000	.1991117	.2050904
3#2021#lowersecondary#0	.2148321	.0007609	282.35	0.000	.2133408	.2163234
3#2021#lowersecondary#1	.2114239	.0007576	279.08	0.000	.2099391	.2129087
3#2021#uppersecondary#0	.2154835	.0008924	241.48	0.000	.2137345	.2172325
3#2021#uppersecondary#1	.2071048	.0008444	245.28	0.000	.2054498	.2087597
3#2021#vocational#0	.2147406	.0009113	235.65	0.000	.2129545	.2165267
3#2021#vocational#1	.2108719	.0012206	172.76	0.000	.2084795	.2132643
4#2017#lowersecondary#0	.5306581	.0026093	203.37	0.000	.5255439	.5357723
4#2017#lowersecondary#1	.562353	.0027158	207.07	0.000	.5570302	.5676758
4#2017#uppersecondary#0	.5376886	.0042438	126.70	0.000	.5293709	.5460063
4#2017#uppersecondary#1	.5823383	.0032952	176.72	0.000	.5758797	.5887968
4#2017#vocational#0	.5470063	.0037187	147.10	0.000	.5397179	.5542947
4#2017#vocational#1	.5962968	.0054562	109.29	0.000	.5856028	.6069909
4#2019#lowersecondary#0	.5226021	.0025353	206.13	0.000	.5176331	.5275711
4#2019#lowersecondary#1	.5537016	.0023602	234.60	0.000	.5490757	.5583276
4#2019#uppersecondary#0	.5221022	.0039596	131.86	0.000	.5143416	.5298628
4#2019#uppersecondary#1	.562006	.0028482	197.32	0.000	.5564236	.5675883
4#2019#vocational#0	.5284191	.0039976	132.19	0.000	.520584	.5362542
4#2019#vocational#1	.5532347	.0056001	98.79	0.000	.5422587	.5642108
4#2021#lowersecondary#0	.4854434	.0023917	202.97	0.000	.4807558	.490131
4#2021#lowersecondary#1	.5081971	.0021709	234.10	0.000	.5039423	.512452
4#2021#uppersecondary#0	.4812535	.0038263	125.77	0.000	.4737541	.488753
4#2021#uppersecondary#1	.5291796	.0026186	202.08	0.000	.5240472	.534312
4#2021#vocational#0	.4860187	.0040115	121.16	0.000	.4781563	.4938812
4#2021#vocational#1	.5110462	.0053674	95.21	0.000	.5005262	.5215661

. margins year#gender#degree

Predictive margins Number of obs = 415,932
Model VCE: Robust

1._predict: Pr(closefriend==1), predict(pr outcome(1))
2._predict: Pr(closefriend==2), predict(pr outcome(2))
3._predict: Pr(closefriend==3), predict(pr outcome(3))
4._predict: Pr(closefriend==4), predict(pr outcome(4))

_predict#year#gender#degree	Margin	Delta-method std. err.	z	P>|z|	[95% conf.	interval]
1#2017#boy#0	.0715585	.0009136	78.33	0.000	.0697679	.0733491
1#2017#boy#1	.0646381	.0008168	79.14	0.000	.0630372	.066239
1#2017#girl#0	.0842665	.000828	101.77	0.000	.0826436	.0858895
1#2017#girl#1	.0704295	.0007616	92.48	0.000	.0689368	.0719221
1#2019#boy#0	.0728916	.0009136	79.78	0.000	.0711009	.0746823
1#2019#boy#1	.069558	.0007938	87.63	0.000	.0680022	.0711138
1#2019#girl#0	.0899935	.0008612	104.50	0.000	.0883056	.0916814
1#2019#girl#1	.0746307	.0007238	103.11	0.000	.0732121	.0760493
1#2021#boy#0	.0788669	.0009644	81.78	0.000	.0769766	.0807571
1#2021#boy#1	.0733732	.0007906	92.81	0.000	.0718237	.0749227
1#2021#girl#0	.1098173	.0009631	114.03	0.000	.1079297	.1117048
1#2021#girl#1	.0949523	.0008156	116.42	0.000	.0933538	.0965508
2#2017#boy#0	.1691405	.0015317	110.42	0.000	.1661383	.1721426
2#2017#boy#1	.1566347	.0014325	109.34	0.000	.153827	.1594423
2#2017#girl#0	.1902436	.0012944	146.98	0.000	.1877067	.1927805
2#2017#girl#1	.1670754	.0013002	128.50	0.000	.1645272	.1696237
2#2019#boy#0	.1714966	.0015121	113.42	0.000	.1685329	.1744603

```
          2#2019#boy#1  |   .1656485   .0013242   125.10   0.000     .1630531    .1682438
         2#2019#girl#0  |    .199121   .0012928   154.03   0.000     .1965872    .2016547
         2#2019#girl#1  |   .1744753   .0011933   146.22   0.000     .1721365     .176814
          2#2021#boy#0  |   .1816045   .0015313   118.59   0.000     .1786031    .1846059
          2#2021#boy#1  |    .172294   .0012838   134.20   0.000     .1697777    .1748103
         2#2021#girl#0  |   .2267284   .0012878   176.06   0.000     .2242043    .2292524
         2#2021#girl#1  |   .2063977   .0011815   174.69   0.000     .2040821    .2087134
          3#2017#boy#0  |   .2007396   .0009817   204.48   0.000     .1988154    .2026637
          3#2017#boy#1  |   .1932703   .0010117   191.03   0.000     .1912874    .1952532
         3#2017#girl#0  |    .210895   .0008071   261.30   0.000     .2093131    .2124768
         3#2017#girl#1  |   .1994762   .0009059   220.19   0.000     .1977006    .2012517
          3#2019#boy#0  |   .2020668    .000957   211.14   0.000      .200191    .2039425
          3#2019#boy#1  |   .1988307    .000902   220.43   0.000     .1970628    .2005986
         3#2019#girl#0  |   .2143909   .0007741   276.95   0.000     .2128736    .2159081
         3#2019#girl#1  |   .2036179   .0008253   246.73   0.000     .2020005    .2052354
          3#2021#boy#0  |   .2071615   .0009062   228.60   0.000     .2053853    .2089377
          3#2021#boy#1  |    .202448   .0008557   236.57   0.000     .2007707    .2041252
         3#2021#girl#0  |   .2219673     .00071   312.64   0.000     .2205758    .2233589
         3#2021#girl#1  |   .2167975   .0007337   295.47   0.000     .2153595    .2182356
          4#2017#boy#0  |   .5585615   .0031128   179.44   0.000     .5524606    .5646624
          4#2017#boy#1  |   .5854568   .0029799   196.47   0.000     .5796164    .5912973
         4#2017#girl#0  |   .5145949   .0024533   209.76   0.000     .5097866    .5194032
         4#2017#girl#1  |    .563019   .0026141   215.38   0.000     .5578955    .5681424
          4#2019#boy#0  |    .553545   .0030542   181.24   0.000     .5475589    .5595312
          4#2019#boy#1  |   .5659628   .0026766   211.45   0.000     .5607168    .5712088
         4#2019#girl#0  |   .4964946    .002396   207.22   0.000     .4917986    .5011907
         4#2019#girl#1  |   .5472761   .0023203   235.86   0.000     .5427284    .5518238
          4#2021#boy#0  |   .5323671   .0030297   175.72   0.000     .5264291    .5383052
          4#2021#boy#1  |   .5518848   .0025437   216.96   0.000     .5468993    .5568703
         4#2021#girl#0  |    .441487   .0022329   197.72   0.000     .4371107    .4458634
         4#2021#girl#1  |   .4818524    .002109   228.47   0.000     .4777188     .485986
------------------------------------------------------------------------------------------

. margins  year#urbanrural

Predictive margins                                      Number of obs = 415,932
Model VCE: Robust

1._predict: Pr(closefriend==1), predict(pr outcome(1))
2._predict: Pr(closefriend==2), predict(pr outcome(2))
3._predict: Pr(closefriend==3), predict(pr outcome(3))
4._predict: Pr(closefriend==4), predict(pr outcome(4))

------------------------------------------------------------------------------------------
                         |            Delta-method
                         |     Margin   std. err.      z    P>|z|     [95% conf. interval]
-------------------------+----------------------------------------------------------------
_predict#year#urbanrural |
            1#2017#urban  |   .0710422    .000553   128.47   0.000     .0699584    .0721261
        1#2017#semiurban  |   .0739805   .0009593    77.12   0.000     .0721003    .0758608
            1#2017#rural  |   .0795542    .001177    67.59   0.000     .0772473    .0818612
            1#2019#urban  |   .0751741   .0005519   136.22   0.000     .0740925    .0762557
        1#2019#semiurban  |   .0783745   .0009573    81.87   0.000     .0764983    .0802507
            1#2019#rural  |   .0821822   .0011757    69.90   0.000     .0798778    .0844865
            1#2021#urban  |   .0886021   .0005965   148.53   0.000     .0874329    .0897712
        1#2021#semiurban  |   .0895185   .0010165    88.06   0.000     .0875262    .0915109
            1#2021#rural  |   .0930513   .0012458    74.69   0.000     .0906096     .095493
            2#2017#urban  |   .1678977   .0008896   188.73   0.000      .166154    .1696413
        2#2017#semiurban  |   .1729738   .0016024   107.94   0.000      .169833    .1761145
            2#2017#rural  |    .182279   .0018968    96.10   0.000     .1785613    .1859966
            2#2019#urban  |   .1750209   .0008531   205.15   0.000     .1733488    .1766931
        2#2019#semiurban  |   .1803645   .0015396   117.15   0.000      .177347    .1833821
            2#2019#rural  |   .1865453   .0018559   100.52   0.000     .1829078    .1901827
            2#2021#urban  |   .1958048   .0008687   225.41   0.000     .1941023    .1975074
        2#2021#semiurban  |   .1971817    .001503   131.19   0.000     .1942358    .2001275
            2#2021#rural  |    .202394    .001807   112.00   0.000     .1988523    .2059357
            3#2017#urban  |    .199595   .0007203   277.09   0.000     .1981831    .2010068
        3#2017#semiurban  |   .2023262   .0009984   202.65   0.000     .2003694    .2042831
            3#2017#rural  |   .2069101   .0010519   196.69   0.000     .2048483    .2089718
            3#2019#urban  |   .2034188   .0006947   292.81   0.000     .2020572    .2047804
        3#2019#semiurban  |     .20605   .0009228   223.29   0.000     .2042414    .2078587
            3#2019#rural  |   .2088699   .0009976   209.37   0.000     .2069147    .2108252
            3#2021#urban  |   .2117629   .0006859   308.73   0.000     .2104185    .2131072
        3#2021#semiurban  |   .2122757   .0008216   258.36   0.000     .2106653    .2138861
            3#2021#rural  |    .214111   .0008674   246.85   0.000      .212411     .215811
            4#2017#urban  |   .5614652   .0016561   339.03   0.000     .5582193    .5647111
        4#2017#semiurban  |   .5507195   .0032453   169.70   0.000     .5443589    .5570801
            4#2017#rural  |   .5312567   .0038201   139.07   0.000     .5237695     .538744
            4#2019#urban  |   .5463861   .0015244   358.43   0.000     .5433984    .5493738
        4#2019#semiurban  |    .535211   .0030583   175.00   0.000     .5292168    .5412051
            4#2019#rural  |   .5224027   .0036977   141.28   0.000     .5151553      .52965
            4#2021#urban  |   .5038303   .0014437   348.98   0.000     .5010006    .5066599
        4#2021#semiurban  |   .5010241   .0028838   173.74   0.000     .4953719    .5066762
            4#2021#rural  |   .4904437   .0034998   140.13   0.000     .4835842    .4973033
------------------------------------------------------------------------------------------
```

3.2. Loneliness

```
. glm loneliness i.year##i.gender##i.schoollevel i.degree  i.immigrant i.urbanrural, family(gamma) link (log) vce(robust) nolog

Generalized linear models                         Number of obs   =    416,572
Optimization     : ML                             Residual df     =    416,548
                                                  Scale parameter =   .1955379
Deviance         =  84402.40005                   (1/df) Deviance =   .2026235
Pearson          =  81450.92023                   (1/df) Pearson  =   .1955379

Variance function: V(u) = u^2                     [Gamma]
Link function    : g(u) = ln(u)                   [Log]

                                                  AIC             =   3.637224
Log pseudolikelihood = -757558.7528               BIC             =   -5305651

------------------------------------------------------------------------------------------------------
                                     |               Robust
                          loneliness | Coefficient  std. err.      z    P>|z|     [95% conf. interval]
```

```
--------------------------------------+----------------------------------------------------------------
                                 year |
                                 2019  |   .003188   .0038701     0.82   0.410    -.0043972    .0107732
                                 2021  |  .1077817   .0038107    28.28   0.000     .1003129    .1152505
                                       |
                               gender |
                                 girl  |  .2580445    .003678    70.16   0.000     .2508357    .2652534
                                       |
                          year#gender |
                            2019#girl  |  .0342266   .0049349     6.94   0.000     .0245544    .0438989
                            2021#girl  |  .0352163   .0048118     7.32   0.000     .0257853    .0446472
                                       |
                          schoollevel |
                       uppersecondary  |  .0919895    .004904    18.76   0.000     .0823778    .1016013
                           vocational  |  .0017647   .0052009     0.34   0.734    -.0084289    .0119582
                                       |
                     year#schoollevel |
                  2019#uppersecondary  |  .0223625   .0064962     3.44   0.001     .0096301    .0350948
                      2019#vocational  |  .0046615   .0074037     0.63   0.529    -.0098494    .0191725
                  2021#uppersecondary  |  .0155224   .0063681     2.44   0.015     .0030412    .0280037
                      2021#vocational  |  .0265715   .0073497     3.62   0.000     .0121662    .0409767
                                       |
                   gender#schoollevel |
                  girl#uppersecondary  | -.0535064   .0060892    -8.79   0.000    -.0654411   -.0415718
                      girl#vocational  |   .044958   .0069843     6.44   0.000     .0312691    .0586469
                                       |
              year#gender#schoollevel |
             2019#girl#uppersecondary  | -.0254509   .0080657    -3.16   0.002    -.0412594   -.0096423
                 2019#girl#vocational  | -.0042738   .0099489    -0.43   0.668    -.0237732    .0152257
             2021#girl#uppersecondary  | -.0273825   .0078502    -3.49   0.000    -.0427687   -.0119964
                 2021#girl#vocational  |  -.025311   .0097459    -2.60   0.009    -.0444126   -.0062094
                                       |
                             1.degree | -.0089792   .0014341    -6.26   0.000      -.01179   -.0061685
                                       |
                            immigrant |
                 one foreign-born parent  |  .0464942   .0026633    17.46   0.000     .0412742    .0517142
born in Finland foreign-born parents  | -.0304367   .0056478    -5.39   0.000    -.0415063   -.0193672
     student and parents born abroad  |  .0652759   .0048647    13.42   0.000     .0557412    .0748106
                                       |
                           urbanrural |
                            semiurban  |  -.011622   .0018729    -6.21   0.000    -.0152927   -.0079513
                                rural  | -.0040757   .0021735    -1.88   0.061    -.0083357    .0001843
                                       |
                                _cons |  .6090909   .0030501   199.70   0.000     .6031129    .6150689
-------------------------------------------------------------------------------------------------------

. testparm  year#gender#schoollevel

 ( 1)  [loneliness]2019.year#2.gender#20.schoollevel = 0
 ( 2)  [loneliness]2019.year#2.gender#30.schoollevel = 0
 ( 3)  [loneliness]2021.year#2.gender#20.schoollevel = 0
 ( 4)  [loneliness]2021.year#2.gender#30.schoollevel = 0

           chi2(  4) =   19.13
         Prob > chi2 =    0.0007

. margins year#gender#schoollevel

Predictive margins                                     Number of obs = 416,572
Model VCE: Robust

Expression: Predicted mean loneliness, predict()

-------------------------------------------------------------------------------------------
                          |            Delta-method
                          |     Margin   std. err.      z    P>|z|     [95% conf. interval]
--------------------------+----------------------------------------------------------------
     year#gender#schoollevel |
 2017#boy#lowersecondary  |   1.834168   .0052904   346.70   0.000     1.823799    1.844537
 2017#boy#uppersecondary  |   2.010896    .007972   252.24   0.000     1.995271    2.026521
     2017#boy#vocational  |   1.837407   .0079514   231.08   0.000     1.821823    1.852992
2017#girl#lowersecondary  |    2.37414   .0054345   436.87   0.000     2.363489    2.384792
2017#girl#uppersecondary  |   2.467286   .0069274   356.16   0.000     2.453708    2.480863
    2017#girl#vocational  |   2.487699   .0102024   243.83   0.000     2.467702    2.507695
 2019#boy#lowersecondary  |   1.840025   .0047466   387.65   0.000     1.830721    1.849328
 2019#boy#uppersecondary  |   2.062937   .0070341   293.28   0.000     2.049151    2.076724
     2019#boy#vocational  |   1.851887   .0085551   216.46   0.000     1.835119    1.868655
2019#girl#lowersecondary  |   2.464651   .0050294   490.05   0.000     2.454793    2.474508
2019#girl#uppersecondary  |   2.553448   .0060608   421.31   0.000      2.54157    2.565327
    2019#girl#vocational  |    2.58354   .0110466   233.88   0.000     2.561889    2.605191
 2021#boy#lowersecondary  |   2.042905   .0050819   402.00   0.000     2.032944    2.052865
 2021#boy#uppersecondary  |   2.274783   .0073566   309.22   0.000     2.260364    2.289201
     2021#boy#vocational  |   2.101621   .0096287   218.27   0.000     2.082749    2.120493
2021#girl#lowersecondary  |   2.739111   .0050727   539.97   0.000     2.729169    2.749053
2021#girl#uppersecondary  |   2.813014   .0060334   466.24   0.000     2.801189    2.824839
    2021#girl#vocational  |   2.873746   .0114222   251.59   0.000     2.851359    2.896133
-------------------------------------------------------------------------------------------

.
. glm loneliness i.year##i.schoollevel##i.gender##i.degree i.urbanrural i.immigrant, family(gamma) link (log) vce(robust) nolog

Generalized linear models                         Number of obs   =    416,572
Optimization     : ML                             Residual df     =    416,531
                                                  Scale parameter =   .1955003
Deviance         =  84384.63318                   (1/df) Deviance =   .2025891
Pearson          =  81431.95292                   (1/df) Pearson  =   .1955003

Variance function: V(u) = u^2                     [Gamma]
Link function    : g(u) = ln(u)                   [Log]

                                                  AIC             =   3.637263
Log pseudolikelihood = -757549.8694               BIC             =   -5305449

-------------------------------------------------------------------------------------------------------
                                      |               Robust
                           loneliness | Coefficient  std. err.      z    P>|z|     [95% conf. interval]
--------------------------------------+----------------------------------------------------------------
                                 year |
                                 2019  |  .0010307   .0055854     0.18   0.854    -.0099164    .0119778
                                 2021  |  .1062887   .0055686    19.09   0.000     .0953745    .1172029
                                       |
                          schoollevel |
```

```
                     uppersecondary  |   .1116765   .0080377    13.89   0.000     .0959229    .1274301
                         vocational  |   .0064699   .0066876     0.97   0.333    -.0066376    .0195774
                                     |
                   year#schoollevel  |
                2019#uppersecondary  |   .0227536   .0110127     2.07   0.039     .0011691     .044338
                    2019#vocational  |  -.0002329   .0096421    -0.02   0.981     -.019131    .0186652
                2021#uppersecondary  |   .0203129   .0109615     1.85   0.064    -.0011712     .041797
                    2021#vocational  |   .0215219   .0096607     2.23   0.026     .0025874    .0404565
                                     |
                             gender  |
                               girl  |   .2633594   .0051695    50.95   0.000     .2532274    .2734914
                                     |
                        year#gender  |
                          2019#girl  |   .0435984   .0071067     6.13   0.000     .0296697    .0575272
                          2021#girl  |   .0398647   .0069961     5.70   0.000     .0261526    .0535767
                                     |
                 schoollevel#gender  |
                uppersecondary#girl  |  -.0653306   .0097209    -6.72   0.000    -.0843833   -.0462779
                    vocational#girl  |   .0425063   .0088612     4.80   0.000     .0251386     .059874
                                     |
            year#schoollevel#gender  |
           2019#uppersecondary#girl  |  -.0304547    .013277    -2.29   0.022    -.0564771   -.0044323
               2019#vocational#girl  |   -.011456   .0127654    -0.90   0.369    -.0364757    .0135637
           2021#uppersecondary#girl  |  -.0325075   .0131145    -2.48   0.013    -.0582113   -.0068036
               2021#vocational#girl  |  -.0297074   .0126029    -2.36   0.018    -.0544086   -.0050063
                                     |
                           1.degree  |    .003909   .0057735     0.68   0.498    -.0074069    .0152248
                                     |
                        year#degree  |
                             2019 1  |   .0028002   .0077524     0.36   0.718    -.0123942    .0179947
                             2021 1  |   .0011983     .00765     0.16   0.876    -.0137953     .016192
                                     |
                 schoollevel#degree  |
                  uppersecondary#1   |  -.0330222   .0102236    -3.23   0.001    -.0530601   -.0129843
                      vocational#1   |  -.0081643   .0107953    -0.76   0.449    -.0293227     .012994
                                     |
            year#schoollevel#degree  |
             2019#uppersecondary#1   |   .0011315   .0137593     0.08   0.934    -.0258361    .0280992
                 2019#vocational#1   |   .0150766   .0153049     0.99   0.325    -.0149204    .0450737
             2021#uppersecondary#1   |  -.0036421   .0135919    -0.27   0.789    -.0302817    .0229976
                 2021#vocational#1   |   .0151642   .0151524     1.00   0.317     -.014534    .0448624
                                     |
                      gender#degree  |
                            girl#1   |  -.0107237   .0073587    -1.46   0.145    -.0251466    .0036991
                                     |
                 year#gender#degree  |
                       2019#girl#1   |  -.0165668   .0098869    -1.68   0.094    -.0359449    .0028112
                       2021#girl#1   |  -.0071822   .0096596    -0.74   0.457    -.0261147    .0117503
                                     |
          schoollevel#gender#degree  |
             uppersecondary#girl#1   |    .019477   .0125786     1.55   0.122    -.0051766    .0441307
                 vocational#girl#1   |   .0018456   .0147224     0.13   0.900    -.0270098    .0307009
                                     |
     year#schoollevel#gender#degree  |
        2019#uppersecondary#girl#1   |   .0093443   .0168636     0.55   0.580    -.0237076    .0423963
            2019#vocational#girl#1   |   .0141679   .0208271     0.68   0.496    -.0266526    .0549883
        2021#uppersecondary#girl#1   |   .0067282   .0165296     0.41   0.684    -.0256692    .0391256
            2021#vocational#girl#1   |   .0096551   .0203322     0.47   0.635    -.0301953    .0495055
                                     |
                         urbanrural  |
                          semiurban  |  -.0117016    .001873    -6.25   0.000    -.0153727   -.0080305
                              rural  |  -.0043033    .002175    -1.98   0.048    -.0085663   -.0000403
                                     |
                          immigrant  |
              one foreign-born parent  |    .046375   .0026633    17.41   0.000      .041155    .0515949
 born in Finland foreign-born parents  |  -.0310909   .0056488    -5.50   0.000    -.0421624   -.0200194
      student and parents born abroad  |   .0650631   .0048673    13.37   0.000     .0555235    .0746028
                                     |
                              _cons  |   .6028187    .004132   145.89   0.000     .5947201    .6109173
------------------------------------------------------------------------------------------------------

. testparm year#schoollevel#gender#degree

 ( 1)  [loneliness]2019.year#20.schoollevel#2.gender#1.degree = 0
 ( 2)  [loneliness]2019.year#30.schoollevel#2.gender#1.degree = 0
 ( 3)  [loneliness]2021.year#20.schoollevel#2.gender#1.degree = 0
 ( 4)  [loneliness]2021.year#30.schoollevel#2.gender#1.degree = 0

           chi2(  4) =    0.63
         Prob > chi2 =    0.9593

.
. glm loneliness i.year##i.schoollevel##i.gender##i.urbanrural i.degree i.immigrant, family(gamma) link (log) vce(robust) nolog

Generalized linear models                          Number of obs   =    416,572
Optimization     : ML                              Residual df     =    416,514
                                                   Scale parameter =     .19551
Deviance         =  84383.45969                    (1/df) Deviance =   .2025945
Pearson          =  81432.65632                    (1/df) Pearson  =     .19551

Variance function: V(u) = u^2                      [Gamma]
Link function    : g(u) = ln(u)                    [Log]

                                                   AIC             =   3.637341
Log pseudolikelihood = -757549.2826                BIC             =   -5305230

------------------------------------------------------------------------------------------------------
                                     |               Robust
                         loneliness  | Coefficient  std. err.      z    P>|z|     [95% conf. interval]
-------------------------------------+----------------------------------------------------------------
                               year  |
                               2019  |   .0044617   .0047781     0.93   0.350    -.0049033    .0138267
                               2021  |   .1076154   .0046898    22.95   0.000     .0984236    .1168072
                                     |
                        schoollevel  |
                     uppersecondary  |   .0920245   .0058857    15.64   0.000     .0804888    .1035602
                         vocational  |    .006109   .0060866     1.00   0.316    -.0058205    .0180385
                                     |
                   year#schoollevel  |
                2019#uppersecondary  |   .0264234   .0077353     3.42   0.001     .0112625    .0415844
                    2019#vocational  |   .0050385   .0086225     0.58   0.559    -.0118613    .0219383
                2021#uppersecondary  |   .0172885   .0075839     2.28   0.023     .0024244    .0321526
```

```
                2021#vocational  |   .0356448   .0085559     4.17   0.000     .0188756    .0524141
                                 |
                          gender |
                            girl  |   .2568683   .0045854    56.02   0.000     .2478812    .2658555
                                 |
                     year#gender |
                       2019#girl  |   .0304899   .0060923     5.00   0.000     .0185492    .0424307
                       2021#girl  |   .0352478   .0059297     5.94   0.000     .0236258    .0468697
                                 |
              schoollevel#gender |
             uppersecondary#girl  |  -.0539762   .0073161    -7.38   0.000    -.0683154    -.039637
                 vocational#girl  |   .0344212   .0080996     4.25   0.000     .0185462    .0502962
                                 |
         year#schoollevel#gender |
        2019#uppersecondary#girl  |  -.0238111   .0096113    -2.48   0.013     -.042649   -.0049732
            2019#vocational#girl  |   .0050413   .0114939     0.44   0.661    -.0174864     .027569
        2021#uppersecondary#girl  |  -.0292992   .0093568    -3.13   0.002    -.0476381   -.0109602
            2021#vocational#girl  |  -.0296344    .011267    -2.63   0.009    -.0517173   -.0075516
                                 |
                      urbanrural |
                       semiurban  |  -.0048329   .0075037    -0.64   0.520    -.0195398    .0098741
                           rural  |  -.0106085   .0079642    -1.33   0.183    -.0262181    .0050011
                                 |
                 year#urbanrural |
                  2019#semiurban  |  -.0095435   .0101312    -0.94   0.346    -.0294003    .0103134
                      2019#rural  |    .002803   .0109124     0.26   0.797    -.0185849    .0241909
                  2021#semiurban  |  -.0001228    .010012    -0.01   0.990    -.0197459    .0195004
                      2021#rural  |   .0009967   .0108082     0.09   0.927     -.020187    .0221804
                                 |
          schoollevel#urbanrural |
        uppersecondary#semiurban  |  -.0134118   .0133018    -1.01   0.313    -.0394827    .0126592
            uppersecondary#rural  |    .017441   .0147369     1.18   0.237    -.0114428    .0463249
            vocational#semiurban  |  -.0265829   .0135815    -1.96   0.050    -.0532023    .0000364
                vocational#rural  |  -.0066426   .0218255    -0.30   0.761    -.0494198    .0361346
                                 |
     year#schoollevel#urbanrural |
   2019#uppersecondary#semiurban  |  -.0021266   .0176723    -0.12   0.904    -.0367637    .0325104
       2019#uppersecondary#rural  |  -.0370393   .0203839    -1.82   0.069     -.076991    .0029123
       2019#vocational#semiurban  |   .0091473   .0194619     0.47   0.638    -.0289974    .0472921
           2019#vocational#rural  |  -.0335809    .031147    -1.08   0.281    -.0946278    .0274661
   2021#uppersecondary#semiurban  |  -.0040949   .0173299    -0.24   0.813    -.0380608     .029871
       2021#uppersecondary#rural  |  -.0089099   .0198819    -0.45   0.654    -.0478778    .0300579
       2021#vocational#semiurban  |  -.0309908   .0192342    -1.61   0.107    -.0686892    .0067076
           2021#vocational#rural  |  -.0680949   .0310398    -2.19   0.028    -.1289318    -.007258
                                 |
               gender#urbanrural |
                  girl#semiurban  |  -.0023684   .0095456    -0.25   0.804    -.0210775    .0163407
                      girl#rural  |   .0101662   .0101938     1.00   0.319    -.0098132    .0301456
                                 |
          year#gender#urbanrural |
             2019#girl#semiurban  |   .0168082   .0128987     1.30   0.193    -.0084728    .0420892
                 2019#girl#rural  |   .0059662   .0139512     0.43   0.669    -.0213777      .03331
             2021#girl#semiurban  |  -.0012767    .012615    -0.10   0.919    -.0260017    .0234483
                 2021#girl#rural  |   .0024697   .0136468     0.18   0.856    -.0242774    .0292169
                                 |
   schoollevel#gender#urbanrural |
   uppersecondary#girl#semiurban  |   .0068258   .0165382     0.41   0.680    -.0255885    .0392402
       uppersecondary#girl#rural  |  -.0026039   .0183001    -0.14   0.887    -.0384715    .0332636
       vocational#girl#semiurban  |   .0650434   .0188979     3.44   0.001     .0280042    .1020826
           vocational#girl#rural  |   .0380564   .0294719     1.29   0.197    -.0197074    .0958201
                                 |
year#schoollevel#gender#urbanrural |
2019#uppersecondary#girl#semiurban  |  -.0094418   .0220165    -0.43   0.668    -.0525933    .0337097
    2019#uppersecondary#girl#rural  |   .0055462   .0250878     0.22   0.825     -.043625    .0547174
    2019#vocational#girl#semiurban  |   -.064605   .0270991    -2.38   0.017    -.1177182   -.0114918
        2019#vocational#girl#rural  |   .0073621    .040919     0.18   0.857    -.0728377     .087562
2021#uppersecondary#girl#semiurban  |   .0207949   .0214248     0.97   0.332     -.021197    .0627868
    2021#uppersecondary#girl#rural  |  -.0106687   .0243348    -0.44   0.661     -.058364    .0370266
    2021#vocational#girl#semiurban  |    .003372   .0263185     0.13   0.898    -.0482114    .0549554
        2021#vocational#girl#rural  |   .0451431   .0403908     1.12   0.264    -.0340215    .1243076
                                 |
                        1.degree |  -.0089291   .0014345    -6.22   0.000    -.0117406   -.0061176
                                 |
                       immigrant |
          one foreign-born parent  |   .0465449   .0026633    17.48   0.000     .0413249     .051765
born in Finland foreign-born parents  |  -.0301483    .005646    -5.34   0.000    -.0412142   -.0190824
     student and parents born abroad  |   .0653372   .0048638    13.43   0.000     .0558043    .0748701
                                 |
                           _cons |   .6088153   .0036942   164.80   0.000     .6015748    .6160557
------------------------------------------------------------------------------------------------------

. testparm year#schoollevel#gender#urbanrural

 ( 1)  [loneliness]2019.year#20.schoollevel#2.gender#2.urbanrural = 0
 ( 2)  [loneliness]2019.year#20.schoollevel#2.gender#3.urbanrural = 0
 ( 3)  [loneliness]2019.year#30.schoollevel#2.gender#2.urbanrural = 0
 ( 4)  [loneliness]2019.year#30.schoollevel#2.gender#3.urbanrural = 0
 ( 5)  [loneliness]2021.year#20.schoollevel#2.gender#2.urbanrural = 0
 ( 6)  [loneliness]2021.year#20.schoollevel#2.gender#3.urbanrural = 0
 ( 7)  [loneliness]2021.year#30.schoollevel#2.gender#2.urbanrural = 0
 ( 8)  [loneliness]2021.year#30.schoollevel#2.gender#3.urbanrural = 0

           chi2(  8) =   11.84
         Prob > chi2 =    0.1583

.
. glm loneliness i.year##i.schoollevel##i.gender##i.immigrant i.degree i.urbanrural, family(gamma) link (log) vce(robust) nolog

Generalized linear models                         Number of obs   =    416,572
Optimization     : ML                             Residual df     =    416,497
                                                  Scale parameter =    .195182
Deviance         =  84309.40086                   (1/df) Deviance =    .202425
Pearson          =  81292.72352                   (1/df) Pearson  =    .195182

Variance function: V(u) = u^2                     [Gamma]
Link function    : g(u) = ln(u)                   [Log]

                                                  AIC             =   3.637245
Log pseudolikelihood = -757512.2532               BIC             =   -5305085

------------------------------------------------------------------------------------------------------------------------------
                                                              |               Robust
```

loneliness	Coefficient	std. err.	z	P>|z|	[95% conf. interval]	
**year**						
2019	.0032171	.004072	0.79	0.430	-.004764	.0111981
2021	.1068165	.0040151	26.60	0.000	.098947	.1146861
**schoollevel**						
uppersecondary	.0970737	.0051442	18.87	0.000	.0869911	.1071562
vocational	.0039669	.0054301	0.73	0.465	-.006676	.0146097
**year#schoollevel**						
2019#uppersecondary	.0198039	.0068373	2.90	0.004	.0064031	.0332048
2019#vocational	.0051993	.0077521	0.67	0.502	-.0099947	.0203932
2021#uppersecondary	.0138903	.0067064	2.07	0.038	.0007459	.0270347
2021#vocational	.0251598	.0076994	3.27	0.001	.0100693	.0402503
**gender**						
girl	.265978	.003872	68.69	0.000	.2583889	.273567
**year#gender**						
2019#girl	.03399	.0052029	6.53	0.000	.0237924	.0441875
2021#girl	.0363529	.0050803	7.16	0.000	.0263957	.04631
**schoollevel#gender**						
uppersecondary#girl	-.0585061	.0063892	-9.16	0.000	-.0710288	-.0459834
vocational#girl	.0468405	.0073031	6.41	0.000	.0325268	.0611543
**year#schoollevel#gender**						
2019#uppersecondary#girl	-.0219232	.0084917	-2.58	0.010	-.0385667	-.0052797
2019#vocational#girl	-.0027613	.0104211	-0.26	0.791	-.0231863	.0176638
2021#uppersecondary#girl	-.0258008	.0082726	-3.12	0.002	-.0420147	-.0095868
2021#vocational#girl	-.0224662	.0102217	-2.20	0.028	-.0425004	-.002432
**immigrant**						
one foreign-born parent	.0550383	.0113294	4.86	0.000	.032833	.0772436
born in Finland foreign-born parents	.0186208	.0239852	0.78	0.438	-.0283894	.065631
student and parents born abroad	.1547082	.0184981	8.36	0.000	.1184526	.1909638
**year#immigrant**						
2019#one foreign-born parent	.0061093	.0151437	0.40	0.687	-.0235718	.0357903
2019#born in Finland foreign-born parents	.013218	.0320739	0.41	0.680	-.0496457	.0760818
2019#student and parents born abroad	-.0174848	.0246403	-0.71	0.478	-.065779	.0308094
2021#one foreign-born parent	.0260509	.0149798	1.74	0.082	-.0033089	.0554108
2021#born in Finland foreign-born parents	-.0318054	.0304364	-1.04	0.296	-.0914597	.0278489
2021#student and parents born abroad	-.0129175	.0237033	-0.54	0.586	-.0593752	.0335402
**schoollevel#immigrant**						
uppersecondary#one foreign-born parent	-.0208964	.0198541	-1.05	0.293	-.0598097	.018017
uppersecondary#born in Finland foreign-born parents	-.0318395	.041789	-0.76	0.446	-.1137444	.0500655
uppersecondary#student and parents born abroad	-.0662818	.0367676	-1.80	0.071	-.138345	.0057814
vocational#one foreign-born parent	.0121429	.0228645	0.53	0.595	-.0326706	.0569565
vocational#born in Finland foreign-born parents	-.044803	.0465788	-0.96	0.336	-.1360957	.0464898
vocational#student and parents born abroad	-.032943	.0347254	-0.95	0.343	-.1010035	.0351175
**year#schoollevel#immigrant**						
2019#uppersecondary#one foreign-born parent	.0058604	.025739	0.23	0.820	-.0445871	.0563078
2019#uppersecondary#born in Finland foreign-born parents	.0328361	.0537057	0.61	0.541	-.0724251	.1380974
2019#uppersecondary#student and parents born abroad	.0406654	.0468277	0.87	0.385	-.0511151	.1324459
2019#vocational#one foreign-born parent	-.0135926	.0314842	-0.43	0.666	-.0753004	.0481153
2019#vocational#born in Finland foreign-born parents	-.0342263	.0657428	-0.52	0.603	-.1630799	.0946273
2019#vocational#student and parents born abroad	.0123652	.0491508	0.25	0.801	-.0839686	.108699
2021#uppersecondary#one foreign-born parent	.0004328	.0253049	0.02	0.986	-.0491638	.0500294
2021#uppersecondary#born in Finland foreign-born parents	.0695107	.0523014	1.33	0.184	-.0329982	.1720196
2021#uppersecondary#student and parents born abroad	.001108	.0449994	0.02	0.980	-.0870891	.0893051
2021#vocational#one foreign-born parent	.0177862	.0314738	0.57	0.572	-.0439014	.0794738
2021#vocational#born in Finland foreign-born parents	.1141993	.0665	1.72	0.086	-.0161384	.2445369
2021#vocational#student and parents born abroad	-.0423256	.0475967	-0.89	0.374	-.1356133	.0509621
**gender#immigrant**						
girl#one foreign-born parent	-.0184359	.0143668	-1.28	0.199	-.0465944	.0097226
girl#born in Finland foreign-born parents	-.0809467	.0307662	-2.63	0.009	-.1412472	-.0206461
girl#student and parents born abroad	-.1464865	.0248181	-5.90	0.000	-.1951291	-.0978439
**year#gender#immigrant**						
2019#girl#one foreign-born parent	.005075	.0190725	0.27	0.790	-.0323065	.0424565
2019#girl#born in Finland foreign-born parents	-.0340903	.0409436	-0.83	0.405	-.1143383	.0461576
2019#girl#student and parents born abroad	.01322	.0330098	0.40	0.689	-.051478	.0779181
2021#girl#one foreign-born parent	-.0326038	.018674	-1.75	0.081	-.0692042	.0039965
2021#girl#born in Finland foreign-born parents	.0547614	.0384793	1.42	0.155	-.0206567	.1301795
2021#girl#student and parents born abroad	.0179564	.0313926	0.57	0.567	-.043572	.0794848
**schoollevel#gender#immigrant**						
uppersecondary#girl#one foreign-born parent	.0228598	.0246264	0.93	0.353	-.0254071	.0711268
uppersecondary#girl#born in Finland foreign-born parents	-.0370229	.0526657	-0.70	0.482	-.1402458	.0662001
uppersecondary#girl#student and parents born abroad	.0810652	.0464603	1.74	0.081	-.0099953	.1721257
vocational#girl#one foreign-born parent	-.0429085	.0298825	-1.44	0.151	-.101477	.0156601
vocational#girl#born in Finland foreign-born parents	-.0104325	.0645554	-0.16	0.872	-.1369588	.1160938
vocational#girl#student and parents born abroad	-.0263657	.0480553	-0.55	0.583	-.1205523	.067821
**year#schoollevel#gender#immigrant**						
2019#uppersecondary#girl#one foreign-born parent	-.032823	.0318627	-1.03	0.303	-.0952727	.0296268
2019#uppersecondary#girl#born in Finland foreign-born parents	.0786284	.0676454	1.16	0.245	-.0539542	.2112109
2019#uppersecondary#girl#student and parents born abroad	-.0664863	.0591044	-1.12	0.261	-.1823287	.0493561
2019#vocational#girl#one foreign-born parent	-.0044162	.0413379	-0.11	0.915	-.0854371	.0766047
2019#vocational#girl#born in Finland foreign-born parents	.110188	.090739	1.21	0.225	-.0676572	.2880332
2019#vocational#girl#student and parents born abroad	-.087994	.0691694	-1.27	0.203	-.2235635	.0475755
2021#uppersecondary#girl#one foreign-born parent	-.0013348	.0310128	-0.04	0.966	-.0621187	.0594492
2021#uppersecondary#girl#born in Finland foreign-born parents	-.0124219	.0651113	-0.19	0.849	-.1400377	.1151939
2021#uppersecondary#girl#student and parents born abroad	-.0188837	.0565585	-0.33	0.738	-.1297363	.0919688
2021#vocational#girl#one foreign-born parent	-.005571	.0405208	-0.14	0.891	-.0849903	.0738482
2021#vocational#girl#born in Finland foreign-born parents	-.1182822	.0905027	-1.31	0.191	-.2956642	.0590999
2021#vocational#girl#student and parents born abroad	-.0292126	.0650952	-0.45	0.654	-.156797	.0983717
1.degree	-.0089591	.0014327	-6.25	0.000	-.0117671	-.006151
**urbanrural**						
semiurban	-.0117668	.0018711	-6.29	0.000	-.015434	-.0080996
rural	-.0041454	.0021718	-1.91	0.056	-.0084021	.0001114
_cons	.6040432	.0031843	189.70	0.000	.5978021	.6102843

```
. testparm year#schoollevel#gender#immigrant

 ( 1)  [loneliness]2019.year#20.schoollevel#2.gender#2.immigrant = 0
 ( 2)  [loneliness]2019.year#20.schoollevel#2.gender#3.immigrant = 0
 ( 3)  [loneliness]2019.year#20.schoollevel#2.gender#4.immigrant = 0
 ( 4)  [loneliness]2019.year#30.schoollevel#2.gender#2.immigrant = 0
 ( 5)  [loneliness]2019.year#30.schoollevel#2.gender#3.immigrant = 0
 ( 6)  [loneliness]2019.year#30.schoollevel#2.gender#4.immigrant = 0
 ( 7)  [loneliness]2021.year#20.schoollevel#2.gender#2.immigrant = 0
 ( 8)  [loneliness]2021.year#20.schoollevel#2.gender#3.immigrant = 0
 ( 9)  [loneliness]2021.year#20.schoollevel#2.gender#4.immigrant = 0
 (10)  [loneliness]2021.year#30.schoollevel#2.gender#2.immigrant = 0
 (11)  [loneliness]2021.year#30.schoollevel#2.gender#3.immigrant = 0
 (12)  [loneliness]2021.year#30.schoollevel#2.gender#4.immigrant = 0

           chi2( 12) =   12.12
         Prob > chi2 =    0.4359

.
.
.
. glm loneliness i.year##i.schoollevel##degree i.year##i.gender##i.degree i.urbanrural i.immigrant, family(gamma) link (log) vce(robust)
nolog

Generalized linear models                         Number of obs   =    416,572
Optimization     : ML                             Residual df     =    416,543
                                                  Scale parameter =   .1956817
Deviance         =  84530.55417                   (1/df) Deviance =   .2029336
Pearson          =  81509.83586                   (1/df) Pearson  =   .1956817

Variance function: V(u) = u^2                     [Gamma]
Link function    : g(u) = ln(u)                   [Log]

                                                  AIC             =   3.637555
Log pseudolikelihood = -757622.8298               BIC             =   -5305459

----------------------------------------------------------------------------------------------------
                                      |               Robust
                           loneliness | Coefficient  std. err.      z    P>|z|     [95% conf. interval]
--------------------------------------+-------------------------------------------------------------
                                 year |
                                 2019 |   .0070937   .0047563     1.49   0.136    -.0022286     .016416
                                 2021 |   .1144835   .0047363    24.17   0.000     .1052005    .1237664
                                      |
                          schoollevel |
                       uppersecondary |   .0711579   .0046128    15.43   0.000      .062117    .0801988
                           vocational |   .0248827   .0044906     5.54   0.000     .0160813    .0336841
                                      |
                     year#schoollevel |
                  2019#uppersecondary |   .0036138   .0062792     0.58   0.565    -.0086931    .0159208
                      2019#vocational |  -.0058119   .0064456    -0.90   0.367     -.018445    .0068212
                  2021#uppersecondary |   .0001492   .0061698     0.02   0.981    -.0119434    .0122417
                      2021#vocational |   .0074904   .0063771     1.17   0.240    -.0050085    .0199893
                                      |
                             1.degree |   .0085213   .0048721     1.75   0.080    -.0010278    .0180704
                                      |
                          year#degree |
                               2019 1 |   .0018997   .0065638     0.29   0.772    -.0109651    .0147646
                               2021 1 |    .000545   .0064664     0.08   0.933    -.0121289     .013219
                                      |
                   schoollevel#degree |
                     uppersecondary#1 |  -.0177593   .0060542    -2.93   0.003    -.0296252   -.0058934
                         vocational#1 |  -.0104203   .0074742    -1.39   0.163    -.0250695     .004229
                                      |
              year#schoollevel#degree |
                2019#uppersecondary#1 |   .0086285   .0080925     1.07   0.286    -.0072324    .0244894
                    2019#vocational#1 |   .0196485   .0105918     1.86   0.064     -.001111    .0404081
                2021#uppersecondary#1 |   .0018176   .0078951     0.23   0.818    -.0136565    .0172916
                    2021#vocational#1 |   .0198573   .0103478     1.92   0.055     -.000424    .0401386
                                      |
                               gender |
                                 girl |   .2617988   .0037477    69.86   0.000     .2544534    .2691441
                                      |
                          year#gender |
                            2019#girl |   .0320942   .0052261     6.14   0.000     .0218513    .0423371
                            2021#girl |   .0243162   .0051562     4.72   0.000     .0142103    .0344221
                                      |
                        gender#degree |
                               girl#1 |  -.0196625   .0052734    -3.73   0.000    -.0299982   -.0093268
                                      |
                   year#gender#degree |
                          2019#girl#1 |  -.0148598    .007152    -2.08   0.038    -.0288775   -.0008422
                          2021#girl#1 |  -.0056915   .0069944    -0.81   0.416    -.0194003    .0080172
                                      |
                           urbanrural |
                            semiurban |  -.0117928   .0018732    -6.30   0.000    -.0154642   -.0081215
                                rural |  -.0041275   .0021771    -1.90   0.058    -.0083945    .0001396
                                      |
                            immigrant |
                 one foreign-born parent |   .0466176   .0026628    17.51   0.000     .0413985    .0518366
born in Finland foreign-born parents  |  -.0305271   .0056528    -5.40   0.000    -.0416064   -.0194479
     student and parents born abroad  |   .0643274   .0048603    13.24   0.000     .0548013    .0738535
                                      |
                                _cons |   .6036328   .0035189   171.54   0.000      .596736    .6105296
----------------------------------------------------------------------------------------------------

. testparm year#schoollevel#degree

 ( 1)  [loneliness]2019.year#20.schoollevel#1.degree = 0
 ( 2)  [loneliness]2019.year#30.schoollevel#1.degree = 0
 ( 3)  [loneliness]2021.year#20.schoollevel#1.degree = 0
 ( 4)  [loneliness]2021.year#30.schoollevel#1.degree = 0

           chi2(  4) =    5.65
         Prob > chi2 =    0.2269

. testparm  year#gender#degree

 ( 1)  [loneliness]2019.year#2.gender#1.degree = 0
 ( 2)  [loneliness]2021.year#2.gender#1.degree = 0

           chi2(  2) =    4.49
```

```
        Prob > chi2 =    0.1062

.
. glm loneliness i.year##i.degree i.urbanrural i.immigrant i.schoollevel i.gender, family(gamma) link (log) vce(robust) nolog

Generalized linear models                         Number of obs   =    416,572
Optimization     : ML                             Residual df     =    416,558
                                                  Scale parameter =   .1957449
Deviance         =   84570.5063                   (1/df) Deviance =   .2030222
Pearson          =  81539.11558                   (1/df) Pearson  =   .1957449

Variance function: V(u) = u^2                     [Gamma]
Link function    : g(u) = ln(u)                   [Log]

                                                  AIC             =   3.637579
Log pseudolikelihood = -757642.8059               BIC             =   -5305613

------------------------------------------------------------------------------------------------------
                                      |               Robust
                           loneliness | Coefficient  std. err.      z    P>|z|     [95% conf. interval]
--------------------------------------+---------------------------------------------------------------
                                 year |
                                 2019 |   .0240144   .0025611     9.38   0.000     .0189947    .0290341
                                 2021 |   .1291831    .002524    51.18   0.000     .1242362    .1341301
                                      |
                             1.degree |  -.0078306   .0026336    -2.97   0.003    -.0129923   -.0026689
                                      |
                          year#degree |
                               2019 1 |  -.0002495   .0035217    -0.07   0.944    -.0071519    .0066528
                               2021 1 |   -.001392    .003437    -0.41   0.685    -.0081285    .0053444
                                      |
                           urbanrural |
                            semiurban |  -.0117575   .0018729    -6.28   0.000    -.0154284   -.0080866
                                rural |   -.003901   .0021753    -1.79   0.073    -.0081646    .0003626
                                      |
                            immigrant |
             one foreign-born parent  |   .0467842   .0026629    17.57   0.000      .041565    .0520033
born in Finland foreign-born parents  |  -.0300733    .005652    -5.32   0.000     -.041151   -.0189956
     student and parents born abroad  |   .0642846   .0048562    13.24   0.000     .0547667    .0738025
                                      |
                          schoollevel |
                      uppersecondary  |   .0637999   .0015227    41.90   0.000     .0608155    .0667843
                          vocational  |   .0257653   .0020757    12.41   0.000      .021697    .0298335
                                      |
                               gender |
                                 girl |   .2670809   .0013944   191.54   0.000     .2643479    .2698139
                                _cons |   .6020578   .0022666   265.62   0.000     .5976153    .6065003
------------------------------------------------------------------------------------------------------

. testparm  year#degree

 ( 1)  [loneliness]2019.year#1.degree = 0
 ( 2)  [loneliness]2021.year#1.degree = 0

          chi2(  2) =    0.20
        Prob > chi2 =    0.9050


. glm loneliness i.year##i.schoollevel##i.urbanrural i.year##i.gender##i.urbanrural i.immigrant i.degree, family(gamma) link (log)
vce(robust) nolog

Generalized linear models                         Number of obs   =    416,572
Optimization     : ML                             Residual df     =    416,532
                                                  Scale parameter =    .195708
Deviance         =   84544.5351                   (1/df) Deviance =   .2029725
Pearson          =  81518.64683                   (1/df) Pearson  =    .195708

Variance function: V(u) = u^2                     [Gamma]
Link function    : g(u) = ln(u)                   [Log]

                                                  AIC             =   3.637642
Log pseudolikelihood = -757629.8203               BIC             =   -5305302

------------------------------------------------------------------------------------------------------
                                      |               Robust
                           loneliness | Coefficient  std. err.      z    P>|z|     [95% conf. interval]
--------------------------------------+---------------------------------------------------------------
                                 year |
                                 2019 |    .009737   .0039784     2.45   0.014     .0019395    .0175346
                                 2021 |   .1170966   .0038984    30.04   0.000     .1094559    .1247374
                                      |
                          schoollevel |
                      uppersecondary  |   .0608235   .0035598    17.09   0.000     .0538464    .0678005
                          vocational  |   .0204552   .0041058     4.98   0.000      .012408    .0285024
                                      |
                     year#schoollevel |
                 2019#uppersecondary  |   .0128551   .0046708     2.75   0.006     .0037005    .0220097
                     2019#vocational  |   .0063344   .0058147     1.09   0.276    -.0050622     .017731
                 2021#uppersecondary  |   .0004788   .0045314     0.11   0.916    -.0084026    .0093602
                     2021#vocational  |   .0212485    .005709     3.72   0.000      .010059     .032438
                                      |
                           urbanrural |
                            semiurban |  -.0111742   .0064065    -1.74   0.081    -.0237308    .0013824
                                rural |  -.0101041   .0070189    -1.44   0.150    -.0238609    .0036527
                                      |
                      year#urbanrural |
                       2019#semiurban |  -.0040126   .0086719    -0.46   0.644    -.0210092     .012984
                           2019#rural |   .0000518   .0096232     0.01   0.996    -.0188093    .0189128
                       2021#semiurban |   -.004221   .0085379    -0.49   0.621    -.0209549     .012513
                           2021#rural |   -.003227   .0095115    -0.34   0.734    -.0218691    .0154152
                                      |
               schoollevel#urbanrural |
            uppersecondary#semiurban  |  -.0096632   .0080548    -1.20   0.230    -.0254503    .0061239
                uppersecondary#rural  |   .0159737   .0088851     1.80   0.072    -.0014408    .0333882
                vocational#semiurban  |  -.0045011   .0096445    -0.47   0.641    -.0234039    .0144018
                    vocational#rural  |   .0116119   .0148928     0.78   0.436    -.0175774    .0408013
                                      |
          year#schoollevel#urbanrural |
       2019#uppersecondary#semiurban  |  -.0064801   .0107352    -0.60   0.546    -.0275206    .0145605
           2019#uppersecondary#rural  |  -.0345819   .0121281    -2.85   0.004    -.0583524   -.0108113
           2019#vocational#semiurban  |  -.0168194   .0138401    -1.22   0.224    -.0439454    .0103067
```

```
             2019#vocational#rural  |  -.0272709   .0206563    -1.32   0.187    -.0677565    .0132147
     2021#uppersecondary#semiurban  |   .0085423   .0104156     0.82   0.412     -.011872    .0289566
         2021#uppersecondary#rural  |  -.0158287   .0117441    -1.35   0.178    -.0388467    .0071892
         2021#vocational#semiurban  |  -.0260398   .0134802    -1.93   0.053    -.0524605    .0003808
             2021#vocational#rural  |  -.0423603   .0203059    -2.09   0.037    -.0821592   -.0025614
                                    |
                             gender |
                              girl  |   .2493127   .0031581    78.94   0.000      .243123    .2555025
                                    |
                        year#gender |
                         2019#girl  |   .0203957   .0042408     4.81   0.000     .0120838    .0287076
                         2021#girl  |   .0172371   .0041312     4.17   0.000       .00914    .0253342
                                    |
                  gender#urbanrural |
                    girl#semiurban  |   .0098513   .0070357     1.40   0.161    -.0039384    .0236411
                        girl#rural  |   .0091948   .0080193     1.15   0.252    -.0065227    .0249123
                                    |
             year#gender#urbanrural |
               2019#girl#semiurban  |   .0061517   .0095618     0.64   0.520     -.012589    .0248925
                   2019#girl#rural  |    .011229   .0110189     1.02   0.308    -.0103676    .0328257
               2021#girl#semiurban  |   .0064432   .0093231     0.69   0.490    -.0118297    .0247161
                   2021#girl#rural  |   .0104363   .0107634     0.97   0.332    -.0106595    .0315321
                                    |
                          immigrant |
           one parent foreign-born  |   .0467919   .0026627    17.57   0.000     .0415731    .0520107
born in Finland parents foreign-born  |  -.0296829   .0056507    -5.25   0.000    -.0407581   -.0186077
     student and parents born abroad  |   .0643597   .0048549    13.26   0.000     .0548442    .0738751
                                    |
                           1.degree |  -.0083275   .0014345    -5.81   0.000    -.0111391   -.0055159
                              _cons |   .6124831    .003104   197.32   0.000     .6063994    .6185668
------------------------------------------------------------------------------------------------------

. testparm  year#schoollevel#urbanrural

 ( 1)  [loneliness]2019.year#20.schoollevel#2.urbanrural = 0
 ( 2)  [loneliness]2019.year#20.schoollevel#3.urbanrural = 0
 ( 3)  [loneliness]2019.year#30.schoollevel#2.urbanrural = 0
 ( 4)  [loneliness]2019.year#30.schoollevel#3.urbanrural = 0
 ( 5)  [loneliness]2021.year#20.schoollevel#2.urbanrural = 0
 ( 6)  [loneliness]2021.year#20.schoollevel#3.urbanrural = 0
 ( 7)  [loneliness]2021.year#30.schoollevel#2.urbanrural = 0
 ( 8)  [loneliness]2021.year#30.schoollevel#3.urbanrural = 0

           chi2(  8) =   18.00
         Prob > chi2 =    0.0213

. testparm  year#gender#urbanrural

 ( 1)  [loneliness]2019.year#2.gender#2.urbanrural = 0
 ( 2)  [loneliness]2019.year#2.gender#3.urbanrural = 0
 ( 3)  [loneliness]2021.year#2.gender#2.urbanrural = 0
 ( 4)  [loneliness]2021.year#2.gender#3.urbanrural = 0

           chi2(  4) =    1.61
         Prob > chi2 =    0.8065
.
.
. glm loneliness i.year##i.schoollevel##i.immigrant i.year##i.gender##i.immigrant i.urbanrural i.degree, family(gamma) link (log)
vce(robust) nolo
> g

Generalized linear models                         Number of obs   =    416,572
Optimization     : ML                             Residual df     =    416,521
                                                  Scale parameter =    .195394
Deviance         =  84474.84819                   (1/df) Deviance =   .2028105
Pearson          =   81385.7194                   (1/df) Pearson  =    .195394

Variance function: V(u) = u^2                     [Gamma]
Link function    : g(u) = ln(u)                   [Log]

                                                  AIC             =   3.637527
Log pseudolikelihood = -757594.9769               BIC             =   -5305230

------------------------------------------------------------------------------------------------------------------------
                                                |               Robust
                                     loneliness | Coefficient  std. err.      z    P>|z|     [95% conf. interval]
------------------------------------------------+-----------------------------------------------------------------------
                                           year |
                                          2019  |    .008624   .0034406     2.51   0.012     .0018806    .0153674
                                          2021  |   .1147868   .0033842    33.92   0.000     .1081538    .1214198
                                                |
                                    schoollevel |
                                uppersecondary  |   .0632222   .0031145    20.30   0.000     .0571178    .0693266
                                    vocational  |   .0230182   .0037237     6.18   0.000       .01572    .0303164
                                                |
                               year#schoollevel |
                           2019#uppersecondary  |   .0074287    .004128     1.80   0.072    -.0006621    .0155194
                               2019#vocational  |   .0033305   .0052891     0.63   0.529     -.007036     .013697
                           2021#uppersecondary  |  -.0008187   .0040093    -0.20   0.838    -.0086767    .0070393
                               2021#vocational  |   .0146079   .0051964     2.81   0.005     .0044232    .0247926
                                                |
                                      immigrant |
                         one foreign-born parent  |   .0559476   .0096868     5.78   0.000     .0369619    .0749332
              born in Finland foreign-born parents  |   .0254506   .0205276     1.24   0.215    -.0147828    .0656839
                   student and parents born abroad  |   .1474949   .0163408     9.03   0.000     .1154675    .1795224
                                                |
                                 year#immigrant |
                    2019#one foreign-born parent  |   .0116143   .0128786     0.90   0.367    -.0136273    .0368558
         2019#born in Finland foreign-born parents  |  -.0047475   .0273657    -0.17   0.862    -.0583832    .0488882
              2019#student and parents born abroad  |  -.0069105   .0217058    -0.32   0.750    -.0494532    .0356321
                    2021#one foreign-born parent  |   .0256486   .0127006     2.02   0.043      .000756    .0505413
         2021#born in Finland foreign-born parents  |  -.0236279   .0261001    -0.91   0.365    -.0747831    .0275273
              2021#student and parents born abroad  |  -.0127302   .0208423    -0.61   0.541    -.0535804    .0281201
                                                |
                          schoollevel#immigrant |
          uppersecondary#one foreign-born parent  |  -.0073507   .0119443    -0.62   0.538    -.0307611    .0160597
uppersecondary#born in Finland foreign-born parents  |  -.0536206   .0256608    -2.09   0.037    -.1039148   -.0033264
     uppersecondary#student and parents born abroad  |  -.0208529   .0228628    -0.91   0.362    -.0656631    .0239573
              vocational#one foreign-born parent  |  -.0055797   .0150364    -0.37   0.711    -.0350506    .0238911
    vocational#born in Finland foreign-born parents  |  -.0480966    .032294    -1.49   0.136    -.1113917    .0151985
         vocational#student and parents born abroad  |  -.0434846   .0246114    -1.77   0.077     -.091722    .0047528
                                                |
```

```
                   year#schoollevel#immigrant |
          2019#uppersecondary#one foreign-born parent  |  -.0138697   .0154292    -0.90   0.369    -.0441104     .016371
2019#uppersecondary#born in Finland foreign-born parents  |    .077878   .0329455     2.36   0.018     .0133059    .1424501
     2019#uppersecondary#student and parents born abroad  |   .0064291   .0290465     0.22   0.825     -.050501    .0633592
                  2019#vocational#one foreign-born parent  |  -.0179489   .0208899    -0.86   0.390    -.0588923    .0229946
    2019#vocational#born in Finland foreign-born parents  |   .0099927   .0458588     0.22   0.828    -.0798889    .0998744
         2019#vocational#student and parents born abroad  |  -.0232664   .0352644    -0.66   0.509    -.0923834    .0458506
          2021#uppersecondary#one foreign-born parent  |  -.0019028   .0149081    -0.13   0.898     -.031122    .0273165
2021#uppersecondary#born in Finland foreign-born parents  |   .0639891   .0316357     2.02   0.043     .0019842     .125994
     2021#uppersecondary#student and parents born abroad  |  -.0074897   .0277718    -0.27   0.787    -.0619215     .046942
                  2021#vocational#one foreign-born parent  |   .0139698   .0204376     0.68   0.494    -.0260872    .0540268
    2021#vocational#born in Finland foreign-born parents  |     .05957   .0459718     1.30   0.195    -.0305331    .1496732
         2021#vocational#student and parents born abroad  |  -.0537018   .0333535    -1.61   0.107    -.1190735    .0116698
                                                          |
                                                   gender |
                                                     girl |   .2590971   .0027639    93.74   0.000       .25368    .2645142
                                                          |
                                              year#gender |
                                                2019#girl |   .0235725    .003743     6.30   0.000     .0162364    .0309086
                                                2021#girl |   .0211889   .0036526     5.80   0.000       .01403    .0283478
                                                          |
                                         gender#immigrant |
                             girl#one foreign-born parent |  -.0200535   .0106804    -1.88   0.060    -.0409867    .0008796
               girl#born in Finland foreign-born parents  |  -.0929162   .0229077    -4.06   0.000    -.1378146   -.0480179
                    girl#student and parents born abroad  |  -.1312474   .0187708    -6.99   0.000    -.1680375   -.0944572
                                                          |
                                    year#gender#immigrant |
                        2019#girl#one foreign-born parent |  -.0036208   .0141087    -0.26   0.797    -.0312734    .0240318
          2019#girl#born in Finland foreign-born parents  |  -.0010119    .030262    -0.03   0.973    -.0603243    .0583005
               2019#girl#student and parents born abroad  |  -.0121745   .0249223    -0.49   0.625    -.0610213    .0366724
                        2021#girl#one foreign-born parent |  -.0308772   .0137752    -2.24   0.025    -.0578762   -.0038783
          2021#girl#born in Finland foreign-born parents  |   .0407392   .0289392     1.41   0.159    -.0159805     .097459
               2021#girl#student and parents born abroad  |   .0147499    .023684     0.62   0.533    -.0316698    .0611696
                                                          |
                                               urbanrural |
                                                semiurban |  -.0119205   .0018714    -6.37   0.000    -.0155883   -.0082528
                                                    rural |  -.0040482   .0021739    -1.86   0.063     -.008309    .0002125
                                                          |
                                                 1.degree |  -.0082936   .0014327    -5.79   0.000    -.0111017   -.0054855
                                                    _cons |   .6073571   .0027389   221.76   0.000      .601989    .6127252
---------------------------------------------------------------------------------------------------------------------------

. testparm  year#schoollevel#immigrant

 ( 1)  [loneliness]2019.year#20.schoollevel#2.immigrant = 0
 ( 2)  [loneliness]2019.year#20.schoollevel#3.immigrant = 0
 ( 3)  [loneliness]2019.year#20.schoollevel#4.immigrant = 0
 ( 4)  [loneliness]2019.year#30.schoollevel#2.immigrant = 0
 ( 5)  [loneliness]2019.year#30.schoollevel#3.immigrant = 0
 ( 6)  [loneliness]2019.year#30.schoollevel#4.immigrant = 0
 ( 7)  [loneliness]2021.year#20.schoollevel#2.immigrant = 0
 ( 8)  [loneliness]2021.year#20.schoollevel#3.immigrant = 0
 ( 9)  [loneliness]2021.year#20.schoollevel#4.immigrant = 0
 (10)  [loneliness]2021.year#30.schoollevel#2.immigrant = 0
 (11)  [loneliness]2021.year#30.schoollevel#3.immigrant = 0
 (12)  [loneliness]2021.year#30.schoollevel#4.immigrant = 0

          chi2( 12) =   13.84
        Prob > chi2 =    0.3113

. testparm  year#gender#immigrant

 ( 1)  [loneliness]2019.year#2.gender#2.immigrant = 0
 ( 2)  [loneliness]2019.year#2.gender#3.immigrant = 0
 ( 3)  [loneliness]2019.year#2.gender#4.immigrant = 0
 ( 4)  [loneliness]2021.year#2.gender#2.immigrant = 0
 ( 5)  [loneliness]2021.year#2.gender#3.immigrant = 0
 ( 6)  [loneliness]2021.year#2.gender#4.immigrant = 0

          chi2(  6) =   11.97
        Prob > chi2 =    0.0627

.
. glm loneliness i.year##i.immigrant i.schoollevel i.gender i.urbanrural i.degree, family(gamma) link (log) vce(robust) nolog

Generalized linear models                         Number of obs   =    416,572
Optimization     : ML                             Residual df     =    416,554
                                                  Scale parameter =   .1957365
Deviance         =  84568.38077                   (1/df) Deviance =    .203019
Pearson          =  81534.83012                   (1/df) Pearson  =   .1957365

Variance function: V(u) = u^2                     [Gamma]
Link function    : g(u) = ln(u)                   [Log]

                                                  AIC             =   3.637593
Log pseudolikelihood = -757641.7431               BIC             =   -5305563

-----------------------------------------------------------------------------------------------------------
                                           |               Robust
                                loneliness | Coefficient  std. err.      z    P>|z|     [95% conf. interval]
-------------------------------------------+---------------------------------------------------------------
                                      year |
                                      2019 |   .0239193   .0018485    12.94   0.000     .0202962    .0275423
                                      2021 |   .1279523   .0018034    70.95   0.000     .1244177     .131487
                                           |
                                 immigrant |
                    one foreign-born parent |   .0424904   .0052675     8.07   0.000     .0321663    .0528145
     born in Finland foreign-born parents  |  -.0465592   .0113898    -4.09   0.000    -.0688829   -.0242356
          student and parents born abroad  |   .0786539    .009772     8.05   0.000     .0595011    .0978067
                                           |
                            year#immigrant |
                   2019#one foreign-born parent |   .0028702    .006926     0.41   0.679    -.0107045    .0164449
2019#born in Finland foreign-born parents  |   .0206658   .0150067     1.38   0.168    -.0087467    .0500783
     2019#student and parents born abroad  |   -.019511   .0128738    -1.52   0.130    -.0447432    .0057212
                   2021#one foreign-born parent |   .0088047   .0067423     1.31   0.192      -.00441    .0220194
2021#born in Finland foreign-born parents  |   .0237071    .014296     1.66   0.097    -.0043126    .0517268
     2021#student and parents born abroad  |  -.0200242   .0122654    -1.63   0.103    -.0440639    .0040155
                                           |
                               schoollevel |
                            uppersecondary |   .0638023   .0015227    41.90   0.000     .0608179    .0667867
                                vocational |   .0257569   .0020754    12.41   0.000     .0216892    .0298247
                                           |
```

```
                                  gender |
                                    girl  |   .2670896   .0013944   191.54   0.000     .2643567    .2698226
                                          |
                              urbanrural |
                               semiurban  |  -.0117684   .0018728    -6.28   0.000    -.0154391   -.0080978
                                   rural  |  -.0038914   .0021753    -1.79   0.074    -.0081549    .0003721
                                          |
                                 1.degree |   -.008401   .0014342    -5.86   0.000    -.0112119     -.00559
                                    _cons |    .602503   .0020093   299.86   0.000     .5985649    .6064411
-----------------------------------------------------------------------------------------------------------

. testparm  year#immigrant

 ( 1)  [loneliness]2019.year#2.immigrant = 0
 ( 2)  [loneliness]2019.year#3.immigrant = 0
 ( 3)  [loneliness]2019.year#4.immigrant = 0
 ( 4)  [loneliness]2021.year#2.immigrant = 0
 ( 5)  [loneliness]2021.year#3.immigrant = 0
 ( 6)  [loneliness]2021.year#4.immigrant = 0

          chi2(  6) =    8.11
        Prob > chi2 =    0.2302

.
. ***Final model*****

. glm loneliness i.year##i.schoollevel##i.urbanrural i.gender i.immigrant i.degree, family(gamma) link (log) vce(robust) nolog

Generalized linear models                         Number of obs   =    416,572
Optimization     : ML                             Residual df     =    416,540
                                                  Scale parameter =   .1957291
Deviance         =  84559.40057                   (1/df) Deviance =   .2030043
Pearson          =  81529.00748                   (1/df) Pearson  =   .1957291

Variance function: V(u) = u^2                     [Gamma]
Link function    : g(u) = ln(u)                   [Log]

                                                  AIC             =   3.637639
Log pseudolikelihood =  -757637.253               BIC             =   -5305391

------------------------------------------------------------------------------------------------------
                                      |               Robust
                           loneliness | Coefficient  std. err.      z    P>|z|     [95% conf. interval]
--------------------------------------+---------------------------------------------------------------
                                 year |
                                 2019  |    .020483   .0030143     6.80   0.000     .0145751    .0263909
                                 2021  |   .1261702   .0029306    43.05   0.000     .1204263    .1319141
                                       |
                          schoollevel |
                      uppersecondary  |   .0599358   .0035667    16.80   0.000     .0529452    .0669265
                           vocational  |   .0220498   .0041041     5.37   0.000     .0140058    .0300937
                                       |
                     year#schoollevel |
                 2019#uppersecondary  |   .0138811   .0046735     2.97   0.003     .0047211     .023041
                      2019#vocational  |    .004509   .0058087     0.78   0.438    -.0068759    .0158939
                 2021#uppersecondary  |   .0013359   .0045359     0.29   0.768    -.0075543     .010226
                      2021#vocational  |   .0196997   .0057036     3.45   0.001     .0085209    .0308785
                                       |
                           urbanrural |
                            semiurban  |  -.0059382   .0047384    -1.25   0.210    -.0152253    .0033489
                                rural  |  -.0051518   .0050767    -1.01   0.310    -.0151019    .0047982
                                       |
                      year#urbanrural |
                       2019#semiurban  |   -.000824   .0063853    -0.13   0.897    -.0133389    .0116909
                           2019#rural  |    .005766   .0069198     0.83   0.405    -.0077965    .0193284
                       2021#semiurban  |  -.0007394   .0062264    -0.12   0.905     -.012943    .0114642
                           2021#rural  |   .0021437   .0067554     0.32   0.751    -.0110966    .0153839
                                       |
               schoollevel#urbanrural |
            uppersecondary#semiurban  |  -.0091486   .0080631    -1.13   0.257    -.0249519    .0066548
                uppersecondary#rural  |   .0162943   .0088909     1.83   0.067    -.0011316    .0337201
                vocational#semiurban  |  -.0048904   .0095913    -0.51   0.610    -.0236891    .0139082
                    vocational#rural  |   .0104382   .0148826     0.70   0.483    -.0187312    .0396077
                                       |
          year#schoollevel#urbanrural |
       2019#uppersecondary#semiurban  |  -.0063643   .0107314    -0.59   0.553    -.0273975    .0146688
           2019#uppersecondary#rural  |  -.0334125   .0121012    -2.76   0.006    -.0571304   -.0096946
           2019#vocational#semiurban  |  -.0191848   .0137707    -1.39   0.164    -.0461749    .0078053
               2019#vocational#rural  |  -.0265259   .0206505    -1.28   0.199    -.0670001    .0139482
       2021#uppersecondary#semiurban  |   .0085482   .0104151     0.82   0.412    -.0118651    .0289614
           2021#uppersecondary#rural  |  -.0148649   .0117294    -1.27   0.205    -.0378542    .0081244
           2021#vocational#semiurban  |  -.0279862   .0134133    -2.09   0.037    -.0542758   -.0016967
               2021#vocational#rural  |   -.041372   .0203013    -2.04   0.042    -.0811618   -.0015822
                                       |
                               gender |
                                 girl  |   .2670311   .0013948   191.45   0.000     .2642974    .2697648
                                       |
                            immigrant |
                 one foreign-born parent  |   .0468037   .0026628    17.58   0.000     .0415848    .0520226
born in Finland foreign-born parents  |  -.0298014    .005651    -5.27   0.000    -.0408772   -.0187257
     student and parents born abroad  |   .0644089   .0048566    13.26   0.000     .0548902    .0739276
                                       |
                             1.degree |  -.0083081   .0014346    -5.79   0.000    -.0111198   -.0054965
                                _cons |   .6031427   .0025763   234.11   0.000     .5980933    .6081922
------------------------------------------------------------------------------------------------------

. testparm  year#schoollevel#urbanrural

 ( 1)  [loneliness]2019.year#20.schoollevel#2.urbanrural = 0
 ( 2)  [loneliness]2019.year#20.schoollevel#3.urbanrural = 0
 ( 3)  [loneliness]2019.year#30.schoollevel#2.urbanrural = 0
 ( 4)  [loneliness]2019.year#30.schoollevel#3.urbanrural = 0
 ( 5)  [loneliness]2021.year#20.schoollevel#2.urbanrural = 0
 ( 6)  [loneliness]2021.year#20.schoollevel#3.urbanrural = 0
 ( 7)  [loneliness]2021.year#30.schoollevel#2.urbanrural = 0
 ( 8)  [loneliness]2021.year#30.schoollevel#3.urbanrural = 0

          chi2(  8) =   18.09
        Prob > chi2 =    0.0206

.
. margins  year#schoollevel#urbanrural
```

Predictive margins   Number of obs = 416,572
Model VCE: Robust

Expression: Predicted mean loneliness, predict()

	Margin	Delta-method std. err.	z	P>|z|	[95% conf.	interval]
year#schoollevel#urbanrural						
2017#lowersecondary#urban	2.124311	.0048122	441.45	0.000	2.114879	2.133743
2017#lowersecondary#semiurban	2.111734	.0087759	240.63	0.000	2.094533	2.128934
2017#lowersecondary#rural	2.113395	.00959	220.38	0.000	2.094599	2.132191
2017#uppersecondary#urban	2.255526	.0062059	363.45	0.000	2.243363	2.26769
2017#uppersecondary#semiurban	2.221753	.0131667	168.74	0.000	2.195947	2.247559
2017#uppersecondary#rural	2.280799	.015449	147.63	0.000	2.250519	2.311078
2017#vocational#urban	2.171672	.007412	292.99	0.000	2.157144	2.186199
2017#vocational#semiurban	2.148282	.0163941	131.04	0.000	2.11615	2.180414
2017#vocational#rural	2.183182	.0296461	73.64	0.000	2.125077	2.241288
2019#lowersecondary#urban	2.168272	.004291	505.31	0.000	2.159862	2.176682
2019#lowersecondary#semiurban	2.153659	.0081954	262.79	0.000	2.137596	2.169722
2019#lowersecondary#rural	2.169604	.0092957	233.40	0.000	2.151384	2.187823
2019#uppersecondary#urban	2.334382	.0053663	435.01	0.000	2.323865	2.3449
2019#uppersecondary#semiurban	2.282959	.0117638	194.07	0.000	2.259902	2.306015
2019#uppersecondary#rural	2.296172	.0145164	158.18	0.000	2.26772	2.324623
2019#vocational#urban	2.22663	.0080723	275.84	0.000	2.210809	2.242451
2019#vocational#semiurban	2.159014	.0175873	122.76	0.000	2.124544	2.193485
2019#vocational#rural	2.192441	.0285746	76.73	0.000	2.136436	2.248446
2021#lowersecondary#urban	2.409978	.0044534	541.15	0.000	2.401249	2.418707
2021#lowersecondary#semiurban	2.393939	.00862	277.72	0.000	2.377044	2.410834
2021#lowersecondary#rural	2.402739	.0097814	245.64	0.000	2.383568	2.42191
2021#uppersecondary#urban	2.562259	.0054587	469.39	0.000	2.55156	2.572958
2021#uppersecondary#semiurban	2.543679	.0121047	210.14	0.000	2.519954	2.567404
2021#uppersecondary#rural	2.558217	.0149405	171.23	0.000	2.528934	2.5875
2021#vocational#urban	2.512723	.0088499	283.93	0.000	2.495378	2.530069
2021#vocational#semiurban	2.415274	.0186039	129.83	0.000	2.378811	2.451737
2021#vocational#rural	2.428868	.0305765	79.44	0.000	2.368939	2.488797

3.3. Belonging at school

```
. glm belonging i.year##i.gender##i.schoollevel i.degree  i.immigrant i.urbanrural, family(gaussian) link (identity) vce(robust) nolog

Generalized linear models                         Number of obs   =    415,367
Optimization     : ML                             Residual df     =    415,343
                                                  Scale parameter =   .8953786
Deviance         =  371889.2404                   (1/df) Deviance =   .8953786
Pearson          =  371889.2404                   (1/df) Pearson  =   .8953786

Variance function: V(u) = 1                       [Gaussian]
Link function    : g(u) = u                       [Identity]

                                                  AIC             =   2.727426
Log pseudolikelihood = -566417.4253               BIC             =   -5001369
```

belonging	Coefficient	Robust std. err.	z	P>|z|	[95% conf.	interval]
year						
2019	-.3215634	.00742	-43.34	0.000	-.3361064	-.3070205
2021	-.3356	.0073358	-45.75	0.000	-.3499779	-.3212222
gender						
girl	-.4124564	.0078157	-52.77	0.000	-.427775	-.3971379
year#gender						
2019#girl	.0967504	.0103427	9.35	0.000	.076479	.1170218
2021#girl	.022597	.0102463	2.21	0.027	.0025145	.0426794
schoollevel						
uppersecondary	-.1329128	.0098734	-13.46	0.000	-.1522642	-.1135614
vocational	.1190908	.0097286	12.24	0.000	.100023	.1381586
year#schoollevel						
2019#uppersecondary	.214194	.0130009	16.48	0.000	.1887128	.2396752
2019#vocational	.071104	.0132217	5.38	0.000	.0451899	.097018
2021#uppersecondary	.1254882	.0129317	9.70	0.000	.1001426	.1508339
2021#vocational	.0563896	.0134648	4.19	0.000	.0299991	.08278
gender#schoollevel						
girl#uppersecondary	.104525	.0130863	7.99	0.000	.0788763	.1301738
girl#vocational	.0691527	.0148184	4.67	0.000	.0401092	.0981962
year#gender#schoollevel						
2019#girl#uppersecondary	-.0708448	.0172448	-4.11	0.000	-.1046439	-.0370456
2019#girl#vocational	-.0684957	.0203216	-3.37	0.001	-.1083253	-.028666
2021#girl#uppersecondary	-.0247914	.017167	-1.44	0.149	-.0584382	.0088553
2021#girl#vocational	-.0305142	.0207231	-1.47	0.141	-.0711308	.0101024
1.degree	.0897663	.0030706	29.23	0.000	.083748	.0957846
immigrant						
one foreign-born parent	-.05755	.0059179	-9.72	0.000	-.0691489	-.0459512
born in Finland foreign-born parents	.034785	.0111834	3.11	0.002	.0128659	.0567041
student and parents born abroad	-.0798716	.0100216	-7.97	0.000	-.0995136	-.0602296
urbanrural						
semiurban	.0283806	.0039749	7.14	0.000	.02059	.0361713
rural	.0634933	.0047161	13.46	0.000	.05425	.0727367
_cons	3.861068	.0059992	643.60	0.000	3.84931	3.872826

```
. testparm  year#gender#schoollevel

 ( 1)  [belonging]2019.year#2.gender#20.schoollevel = 0
 ( 2)  [belonging]2019.year#2.gender#30.schoollevel = 0
 ( 3)  [belonging]2021.year#2.gender#20.schoollevel = 0
 ( 4)  [belonging]2021.year#2.gender#30.schoollevel = 0

           chi2(  4) =   23.12
         Prob > chi2 =    0.0001

. margins year#gender#schoollevel

Predictive margins                                     Number of obs = 415,367
Model VCE: Robust

Expression: Predicted mean belonging, predict()

-------------------------------------------------------------------------------------------
                          |            Delta-method
                          |     Margin   std. err.      z    P>|z|     [95% conf. interval]
--------------------------+----------------------------------------------------------------
     year#gender#schoollevel |
 2017#boy#lowersecondary  |   3.916478   .0056108   698.02   0.000     3.905481    3.927475
 2017#boy#uppersecondary  |   3.783565   .0081207   465.91   0.000     3.767649    3.799482
     2017#boy#vocational  |   4.035569   .0079485   507.71   0.000      4.01999    4.051148
2017#girl#lowersecondary  |   3.504022    .005454   642.47   0.000     3.493332    3.514711
2017#girl#uppersecondary  |   3.475634   .0066656   521.43   0.000     3.462569    3.488698
    2017#girl#vocational  |   3.692265   .0098323   375.52   0.000     3.672994    3.711536
 2019#boy#lowersecondary  |   3.594915   .0048549   740.47   0.000     3.585399     3.60443
 2019#boy#uppersecondary  |   3.676196   .0069669   527.67   0.000     3.662541    3.689851
     2019#boy#vocational  |   3.785109    .007591   498.63   0.000     3.770231    3.799987
2019#girl#lowersecondary  |   3.279209   .0047209   694.62   0.000     3.269956    3.288461
2019#girl#uppersecondary  |    3.39417   .0056709   598.52   0.000     3.383055    3.405285
    2019#girl#vocational  |    3.47006    .009542   363.66   0.000     3.451358    3.488762
 2021#boy#lowersecondary  |   3.580878   .0047234   758.12   0.000      3.57162    3.590136
 2021#boy#uppersecondary  |   3.573453   .0069349   515.29   0.000     3.559861    3.587045
     2021#boy#vocational  |   3.756358   .0080828   464.73   0.000     3.740516      3.7722
2021#girl#lowersecondary  |   3.191019   .0046447   687.02   0.000     3.181915    3.200122
2021#girl#uppersecondary  |   3.263328   .0056643   576.13   0.000     3.252226    3.274429
    2021#girl#vocational  |   3.405137   .0100816   337.76   0.000     3.385378    3.424897
-------------------------------------------------------------------------------------------

.
.
. glm belonging i.year##i.gender##i.schoollevel##i.degree i.urbanrural i.immigrant, family(gaussian) link (identity) vce(robust) nolog

Generalized linear models                         Number of obs   =    415,367
Optimization     : ML                             Residual df     =    415,326
                                                  Scale parameter =   .8951363
Deviance         =  371773.3956                   (1/df) Deviance =   .8951363
Pearson          =  371773.3956                   (1/df) Pearson  =   .8951363

Variance function: V(u) = 1                       [Gaussian]
Link function    : g(u) = u                       [Identity]

                                                  AIC             =   2.727197
Log pseudolikelihood =  -566352.721               BIC             =   -5001265

-------------------------------------------------------------------------------------------------------
                                     |               Robust
                           belonging | Coefficient  std. err.      z    P>|z|     [95% conf. interval]
-------------------------------------+-----------------------------------------------------------------
                                year |
                                2019 |  -.3453796   .0106703   -32.37   0.000    -.3662931   -.3244662
                                2021 |  -.3467845   .0106388   -32.60   0.000    -.3676363   -.3259328
                                     |
                              gender |
                                girl |  -.3956018   .0109536   -36.12   0.000    -.4170705   -.3741332
                                     |
                         year#gender |
                           2019#girl |   .0801521   .0148597     5.39   0.000     .0510276    .1092766
                           2021#girl |  -.0053382   .0148412    -0.36   0.719    -.0344265      .02375
                                     |
                         schoollevel |
                      uppersecondary |  -.1223141   .0162316    -7.54   0.000    -.1541275   -.0905006
                          vocational |   .1213819   .0125634     9.66   0.000     .0967581    .1460058
                                     |
                    year#schoollevel |
                 2019#uppersecondary |   .2087424   .0221114     9.44   0.000     .1654048      .25208
                     2019#vocational |   .1042069   .0172823     6.03   0.000     .0703342    .1380796
                 2021#uppersecondary |   .1125752   .0223945     5.03   0.000     .0686827    .1564676
                     2021#vocational |   .0834274   .0176876     4.72   0.000     .0487603    .1180945
                                     |
                  gender#schoollevel |
                 girl#uppersecondary |     .07967   .0208484     3.82   0.000     .0388079    .1205321
                     girl#vocational |   .0684258   .0187346     3.65   0.000     .0317068    .1051449
                                     |
             year#gender#schoollevel |
            2019#girl#uppersecondary |  -.0388703   .0283236    -1.37   0.170    -.0943836     .016643
                2019#girl#vocational |  -.0626146   .0259587    -2.41   0.016    -.1134927   -.0117364
            2021#girl#uppersecondary |  -.0155847   .0287205    -0.54   0.587    -.0718757    .0407064
                2021#girl#vocational |  -.0354539    .026637    -1.33   0.183    -.0876614    .0167536
                                     |
                            1.degree |   .0760741   .0112285     6.78   0.000     .0540667    .0980814
                                     |
                         year#degree |
                              2019 1 |   .0453186   .0148567     3.05   0.002        .0162    .0744371
                              2021 1 |   .0217277   .0147084     1.48   0.140    -.0071003    .0505558
                                     |
                       gender#degree |
                              girl#1 |  -.0354594   .0156347    -2.27   0.023    -.0661028   -.0048161
                                     |
                  year#gender#degree |
                         2019#girl#1 |   .0353667   .0207194     1.71   0.088    -.0052426     .075976
                         2021#girl#1 |   .0554303    .020559     2.70   0.007     .0151355    .0957251
                                     |
                  schoollevel#degree |
                    uppersecondary#1 |  -.0122325   .0206043    -0.59   0.553    -.0526161    .0281511
                        vocational#1 |  -.0121422   .0201158    -0.60   0.546    -.0515684     .027284
                                     |
             year#schoollevel#degree |
               2019#uppersecondary#1 |  -.0026067   .0275715    -0.09   0.925    -.0566459    .0514324
```

```
                  2019#vocational#1  |  -.0670772   .0273054    -2.46   0.014    -.1205947   -.0135597
              2021#uppersecondary#1  |    .013467   .0276836     0.49   0.627     -.040792    .0677259
                  2021#vocational#1  |  -.0601658   .0277482    -2.17   0.030    -.1145512   -.0057804
                                     |
           gender#schoollevel#degree |
              girl#uppersecondary#1  |   .0460609     .02701     1.71   0.088    -.0068778    .0989995
                  girl#vocational#1  |  -.0209802   .0313279    -0.67   0.503    -.0823817    .0404213
                                     |
      year#gender#schoollevel#degree |
         2019#girl#uppersecondary#1  |  -.0555484   .0360109    -1.54   0.123    -.1261285    .0150316
             2019#girl#vocational#1  |  -.0055027   .0427706    -0.13   0.898    -.0893316    .0783261
         2021#girl#uppersecondary#1  |  -.0258054   .0361645    -0.71   0.476    -.0966865    .0450757
             2021#girl#vocational#1  |   .0419291   .0434053     0.97   0.334    -.0431438     .127002
                                     |
                          urbanrural |
                          semiurban  |   .0287199   .0039753     7.22   0.000     .0209285    .0365114
                              rural  |   .0647499   .0047191    13.72   0.000     .0555005    .0739992
                                     |
                           immigrant |
             one foreign-born parent |  -.0572372   .0059178    -9.67   0.000    -.0688358   -.0456386
born in Finland foreign-born parents |   .0361667   .0111861     3.23   0.001     .0142422    .0580911
     student and parents born abroad |  -.0787085    .010025    -7.85   0.000    -.0983572   -.0590599
                                     |
                               _cons |   3.867449   .0080475   480.58   0.000     3.851676    3.883221
------------------------------------------------------------------------------------------------------

. testparm year#schoollevel#gender#degree

 ( 1)  [belonging]2019.year#2.gender#20.schoollevel#1.degree = 0
 ( 2)  [belonging]2019.year#2.gender#30.schoollevel#1.degree = 0
 ( 3)  [belonging]2021.year#2.gender#20.schoollevel#1.degree = 0
 ( 4)  [belonging]2021.year#2.gender#30.schoollevel#1.degree = 0

           chi2(  4) =    4.02
         Prob > chi2 =    0.4027

.
. glm belonging i.year##i.gender##i.schoollevel##i.urbanrural i.degree i.immigrant, family(gaussian) link (identity) vce(robust) nolog

Generalized linear models                         Number of obs   =    415,367
Optimization     : ML                             Residual df     =    415,309
                                                  Scale parameter =    .895038
Deviance         =  371717.3366                   (1/df) Deviance =    .895038
Pearson          =  371717.3366                   (1/df) Pearson  =    .895038

Variance function: V(u) = 1                       [Gaussian]
Link function    : g(u) = u                       [Identity]

                                                  AIC             =   2.727128
Log pseudolikelihood = -566321.4025               BIC             =   -5001101

------------------------------------------------------------------------------------------------------
                                     |               Robust
                           belonging | Coefficient  std. err.      z    P>|z|     [95% conf. interval]
-------------------------------------+----------------------------------------------------------------
                                year |
                               2019  |  -.3033464   .0091435   -33.18   0.000    -.3212672   -.2854255
                               2021  |  -.3260877   .0090543   -36.01   0.000    -.3438338   -.3083415
                                     |
                              gender |
                               girl  |  -.4192744   .0097542   -42.98   0.000    -.4383923   -.4001565
                                     |
                         year#gender |
                          2019#girl  |    .101457   .0127288     7.97   0.000      .076509    .1264049
                          2021#girl  |   .0306446   .0126307     2.43   0.015     .0058888    .0554003
                                     |
                         schoollevel |
                     uppersecondary  |  -.1587734   .0119421   -13.30   0.000    -.1821795   -.1353674
                         vocational  |   .1170207   .0114955    10.18   0.000       .09449    .1395515
                                     |
                    year#schoollevel |
                2019#uppersecondary  |   .2169093   .0155649    13.94   0.000     .1864026    .2474159
                    2019#vocational  |   .0454841   .0155025     2.93   0.003     .0150997    .0758685
                2021#uppersecondary  |   .1371631   .0154649     8.87   0.000     .1068525    .1674737
                    2021#vocational  |    .028786   .0158066     1.82   0.069    -.0021943    .0597663
                                     |
                  gender#schoollevel |
                girl#uppersecondary  |   .1224918    .015818     7.74   0.000     .0914892    .1534945
                    girl#vocational  |   .0900056    .017207     5.23   0.000     .0562805    .1237306
                                     |
             year#gender#schoollevel |
           2019#girl#uppersecondary  |  -.0914425   .0206289    -4.43   0.000    -.1318744   -.0510105
               2019#girl#vocational  |  -.0816159   .0234657    -3.48   0.001    -.1276078    -.035624
           2021#girl#uppersecondary  |  -.0309169   .0205124    -1.51   0.132    -.0711205    .0092866
               2021#girl#vocational  |   -.026285   .0239593    -1.10   0.273    -.0732444    .0206743
                                     |
                          urbanrural |
                          semiurban  |   .0326805   .0145382     2.25   0.025     .0041862    .0611748
                              rural  |   .0697525   .0153944     4.53   0.000       .03958    .0999251
                                     |
                     year#urbanrural |
                     2019#semiurban  |  -.0563833   .0194559    -2.90   0.004     -.094516   -.0182505
                         2019#rural  |  -.0537629    .021043    -2.55   0.011    -.0950064   -.0125193
                     2021#semiurban  |  -.0190434   .0192928    -0.99   0.324    -.0568566    .0187698
                         2021#rural  |  -.0412308   .0206051    -2.00   0.045     -.081616   -.0008456
                                     |
                   gender#urbanrural |
                     girl#semiurban  |   .0138113   .0202126     0.68   0.494    -.0258046    .0534272
                         girl#rural  |   .0268961   .0216829     1.24   0.215    -.0156016    .0693938
                                     |
              year#gender#urbanrural |
                2019#girl#semiurban  |  -.0029065   .0270452    -0.11   0.914     -.055914    .0501011
                    2019#girl#rural  |  -.0276943   .0295729    -0.94   0.349    -.0856561    .0302674
                2021#girl#semiurban  |  -.0160482   .0267815    -0.60   0.549     -.068539    .0364426
                    2021#girl#rural  |  -.0333037   .0291656    -1.14   0.254    -.0904672    .0238598
                                     |
              schoollevel#urbanrural |
           uppersecondary#semiurban  |   .0817824   .0260991     3.13   0.002      .030629    .1329357
               uppersecondary#rural  |   .1128302   .0293657     3.84   0.000     .0552744     .170386
               vocational#semiurban  |   .0379291   .0247586     1.53   0.126    -.0105969    .0864551
                   vocational#rural  |  -.0843967   .0404163    -2.09   0.037    -.1636111   -.0051822
                                     |
```

```
         year#schoolevel#urbanrural |
      2019#uppersecondary#semiurban  |  -.0189384   .0347387    -0.55   0.586     -.087025    .0491481
          2019#uppersecondary#rural  |  -.0140253   .0402042    -0.35   0.727    -.0928242    .0647735
          2019#vocational#semiurban  |   .0521405   .0342547     1.52   0.128    -.0149975    .1192785
              2019#vocational#rural  |   .2219755   .0549244     4.04   0.000     .1143257    .3296253
      2021#uppersecondary#semiurban  |   -.034928   .0346845    -1.01   0.314    -.1029084    .0330524
          2021#uppersecondary#rural  |  -.0621267   .0402157    -1.54   0.122    -.1409481    .0166946
          2021#vocational#semiurban  |   .0597784   .0347072     1.72   0.085    -.0082464    .1278032
              2021#vocational#rural  |   .2550375   .0555599     4.59   0.000      .146142     .363933
                                     |
       gender#schoollevel#urbanrural |
      girl#uppersecondary#semiurban  |  -.0549356   .0347605    -1.58   0.114    -.1230648    .0131937
          girl#uppersecondary#rural  |  -.0652771   .0391254    -1.67   0.095    -.1419615    .0114073
          girl#vocational#semiurban  |  -.1151656   .0402708    -2.86   0.004     -.194095   -.0362363
              girl#vocational#rural  |   .0066217   .0614147     0.11   0.914    -.1137488    .1269922
                                     |
  year#gender#schoollevel#urbanrural |
 2019#girl#uppersecondary#semiurban  |   .0635307   .0462729     1.37   0.170    -.0271626    .1542239
     2019#girl#uppersecondary#rural  |   .0824387   .0532942     1.55   0.122    -.0220161    .1868935
     2019#girl#vocational#semiurban  |   .0636595   .0556205     1.14   0.252    -.0453548    .1726738
         2019#girl#vocational#rural  |   .0003106   .0830577     0.00   0.997    -.1624795    .1631008
 2021#girl#uppersecondary#semiurban  |   -.001846   .0462355    -0.04   0.968    -.0924659    .0887739
     2021#girl#uppersecondary#rural  |   .0295076   .0533726     0.55   0.580    -.0751008    .1341161
     2021#girl#vocational#semiurban  |   .0219062   .0564647     0.39   0.698    -.0887625    .1325749
         2021#girl#vocational#rural  |  -.1754876   .0840646    -2.09   0.037    -.3402512    -.010724
                                     |
                            1.degree |   .0895499   .0030713    29.16   0.000     .0835304    .0955695
                                     |
                           immigrant |
             one foreign-born parent  |  -.0576995   .0059164    -9.75   0.000    -.0692955   -.0461035
 born in Finland foreign-born parents  |    .033746   .0111821     3.02   0.003     .0118294    .0556626
      student and parents born abroad  |  -.0803364   .0100206    -8.02   0.000    -.0999764   -.0606963
                                     |
                               _cons |   3.859372   .0072486   532.43   0.000     3.845165    3.873579
------------------------------------------------------------------------------------------------------

. testparm year#schoollevel#gender#urbanrural

 ( 1)  [belonging]2019.year#2.gender#20.schoollevel#2.urbanrural = 0
 ( 2)  [belonging]2019.year#2.gender#20.schoollevel#3.urbanrural = 0
 ( 3)  [belonging]2019.year#2.gender#30.schoollevel#2.urbanrural = 0
 ( 4)  [belonging]2019.year#2.gender#30.schoollevel#3.urbanrural = 0
 ( 5)  [belonging]2021.year#2.gender#20.schoollevel#2.urbanrural = 0
 ( 6)  [belonging]2021.year#2.gender#20.schoollevel#3.urbanrural = 0
 ( 7)  [belonging]2021.year#2.gender#30.schoollevel#2.urbanrural = 0
 ( 8)  [belonging]2021.year#2.gender#30.schoollevel#3.urbanrural = 0

           chi2(  8) =   11.55
         Prob > chi2 =    0.1725

.
. glm belonging i.year##i.gender##i.schoollevel##i.immigrant i.degree i.urbanrural, family(gaussian) link (identity) vce(robust) nolog

Generalized linear models                         Number of obs   =    415,367
Optimization     : ML                             Residual df     =    415,292
                                                  Scale parameter =   .8949902
Deviance         =  371682.2796                   (1/df) Deviance =   .8949902
Pearson          =  371682.2796                   (1/df) Pearson  =   .8949902

Variance function: V(u) = 1                       [Gaussian]
Link function    : g(u) = u                       [Identity]

                                                  AIC             =   2.727115
Log pseudolikelihood = -566301.8148               BIC             =   -5000916
```

```
--------------------------------------------------------------------------------------------------------------------------
                                                         |               Robust
                                               belonging | Coefficient  std. err.      z    P>|z|     [95% conf. interval]
---------------------------------------------------------+----------------------------------------------------------------
                                                    year |
                                                    2019  |    -.32565   .0077869   -41.82   0.000    -.3409119    -.310388
                                                    2021  |  -.3386439   .0077038   -43.96   0.000     -.353743   -.3235447
                                                         |
                                                  gender |
                                                    girl  |  -.4220037   .0082056   -51.43   0.000    -.4380863   -.4059211
                                                         |
                                             year#gender |
                                               2019#girl  |   .0988114   .0108949     9.07   0.000     .0774577     .120165
                                               2021#girl  |   .0169861   .0108074     1.57   0.116     -.004196    .0381682
                                                         |
                                             schoollevel |
                                          uppersecondary  |  -.1353197    .010337   -13.09   0.000    -.1555799   -.1150596
                                              vocational  |   .1186405   .0100977    11.75   0.000     .0988495    .1384316
                                                         |
                                        year#schoollevel |
                                     2019#uppersecondary  |   .2164492   .0136752    15.83   0.000     .1896463    .2432521
                                         2019#vocational  |   .0697203   .0138034     5.05   0.000     .0426662    .0967744
                                     2021#uppersecondary  |   .1248342   .0136228     9.16   0.000     .0981341    .1515343
                                         2021#vocational  |   .0545846   .0140463     3.89   0.000     .0270543    .0821148
                                                         |
                                      gender#schoollevel |
                                     girl#uppersecondary  |   .1057049   .0137073     7.71   0.000     .0788391    .1325707
                                         girl#vocational  |   .0695777   .0154345     4.51   0.000     .0393266    .0998288
                                                         |
                                 year#gender#schoollevel |
                                2019#girl#uppersecondary  |  -.0717337   .0181507    -3.95   0.000    -.1073083    -.036159
                                    2019#girl#vocational  |  -.0698252   .0212786    -3.28   0.001    -.1115306   -.0281199
                                2021#girl#uppersecondary  |  -.0152314   .0180986    -0.84   0.400     -.050704    .0202413
                                    2021#girl#vocational  |  -.0328787   .0216973    -1.52   0.130    -.0754047    .0096473
                                                         |
                                               immigrant |
                                 one foreign-born parent  |  -.0674393    .022476    -3.00   0.003    -.1114915   -.0233872
                    born in Finland foreign-born parents  |  -.0682133   .0462267    -1.48   0.140    -.1588159    .0223894
                         student and parents born abroad  |  -.2355987   .0396328    -5.94   0.000    -.3132776   -.1579197
                                                         |
                                          year#immigrant |
                            2019#one foreign-born parent  |   .0055824   .0297502     0.19   0.851    -.0527268    .0638917
               2019#born in Finland foreign-born parents  |   .0611661   .0592762     1.03   0.302    -.0550131    .1773452
                    2019#student and parents born abroad  |   .0723476   .0504712     1.43   0.152    -.0265741    .1712693
                            2021#one foreign-born parent  |   -.030677    .029472    -1.04   0.298    -.0884411    .0270871
               2021#born in Finland foreign-born parents  |   .1022341   .0570992     1.79   0.073    -.0096782    .2141465
                    2021#student and parents born abroad  |   .0964185   .0491798     1.96   0.050     .0000279    .1928091
```

```
                                                          |
                                        gender#immigrant  |
                              girl#one foreign-born parent  |   .0281926   .0311427     0.91   0.365    -.0328461    .0892313
                  girl#born in Finland foreign-born parents  |   .0619534   .0638672     0.97   0.332    -.0632239    .1871307
                       girl#student and parents born abroad  |    .181284   .0556965     3.25   0.001      .072121    .2904471
                                                          |
                                   year#gender#immigrant  |
                         2019#girl#one foreign-born parent  |  -.0305075   .0409659    -0.74   0.456    -.1107992    .0497842
             2019#girl#born in Finland foreign-born parents  |   .0742551   .0806134     0.92   0.357    -.0837442    .2322544
                  2019#girl#student and parents born abroad  |  -.0340542   .0718526    -0.47   0.636    -.1748827    .1067743
                         2021#girl#one foreign-born parent  |   .0369165   .0405807     0.91   0.363    -.0426202    .1164532
             2021#girl#born in Finland foreign-born parents  |   .0698707    .078432     0.89   0.373    -.0838531    .2235946
                  2021#girl#student and parents born abroad  |   .0332731   .0694141     0.48   0.632     -.102776    .1693223
                                                          |
                                   schoollevel#immigrant  |
                    uppersecondary#one foreign-born parent  |  -.0191548   .0405161    -0.47   0.636    -.0985648    .0602552
        uppersecondary#born in Finland foreign-born parents  |    .004709   .0806177     0.06   0.953    -.1532988    .1627169
             uppersecondary#student and parents born abroad  |   .0369099   .0783551     0.47   0.638    -.1166633    .1904831
                        vocational#one foreign-born parent  |  -.0091478     .04436    -0.21   0.837    -.0960919    .0777962
            vocational#born in Finland foreign-born parents  |   .0595109   .0920738     0.65   0.518    -.1209503    .2399722
                 vocational#student and parents born abroad  |  -.0564041   .0696384    -0.81   0.418    -.1928928    .0800846
                                                          |
                              year#schoollevel#immigrant  |
               2019#uppersecondary#one foreign-born parent  |   .0204854   .0526795     0.39   0.697    -.0827645    .1237352
   2019#uppersecondary#born in Finland foreign-born parents  |  -.0885628   .1023086    -0.87   0.387     -.289084    .1119584
        2019#uppersecondary#student and parents born abroad  |  -.0050295   .0959498    -0.05   0.958    -.1930876    .1830285
                   2019#vocational#one foreign-born parent  |  -.0295043   .0587437    -0.50   0.615    -.1446397    .0856311
       2019#vocational#born in Finland foreign-born parents  |   .0440617    .114903     0.38   0.701    -.1811441    .2692675
            2019#vocational#student and parents born abroad  |   .1205486   .0907687     1.33   0.184    -.0573548    .2984521
               2021#uppersecondary#one foreign-born parent  |   .0834168    .052006     1.60   0.109    -.0185131    .1853467
   2021#uppersecondary#born in Finland foreign-born parents  |    -.09448   .0996128    -0.95   0.343    -.2897174    .1007574
        2021#uppersecondary#student and parents born abroad  |  -.0443593    .094822    -0.47   0.640     -.230207    .1414884
                   2021#vocational#one foreign-born parent  |   .0188189   .0599555     0.31   0.754    -.0986916    .1363295
       2021#vocational#born in Finland foreign-born parents  |   -.173576   .1233372    -1.41   0.159    -.4153124    .0681604
            2021#vocational#student and parents born abroad  |   .1313127   .0919192     1.43   0.153    -.0488456    .3114711
                                                          |
                            gender#schoollevel#immigrant  |
               girl#uppersecondary#one foreign-born parent  |  -.0009705   .0544833    -0.02   0.986    -.1077559    .1058148
   girl#uppersecondary#born in Finland foreign-born parents  |   .0859497   .1070964     0.80   0.422    -.1239555    .2958548
        girl#uppersecondary#student and parents born abroad  |     .03106   .1026252     0.30   0.762    -.1700817    .2322018
                   girl#vocational#one foreign-born parent  |   .0144712    .066557     0.22   0.828    -.1159781    .1449205
       girl#vocational#born in Finland foreign-born parents  |  -.1045607   .1360643    -0.77   0.442    -.3712419    .1621205
            girl#vocational#student and parents born abroad  |   .0809607   .1050887     0.77   0.441    -.1250093    .2869308
                                                          |
                       year#gender#schoollevel#immigrant  |
          2019#girl#uppersecondary#one foreign-born parent  |   .0405903    .070256     0.58   0.563     -.097109    .1782896
2019#girl#uppersecondary#born in Finland foreign-born parents  |  -.1021332   .1350504    -0.76   0.449    -.3668272    .1625608
   2019#girl#uppersecondary#student and parents born abroad  |  -.0984289   .1268267    -0.78   0.438    -.3470045    .1501468
              2019#girl#vocational#one foreign-born parent  |   .0416519   .0883546     0.47   0.637    -.1315198    .2148237
  2019#girl#vocational#born in Finland foreign-born parents  |  -.2077853   .1741703    -1.19   0.233    -.5491529    .1335822
       2019#girl#vocational#student and parents born abroad  |   .0194502   .1385676     0.14   0.888    -.2521374    .2910378
          2021#girl#uppersecondary#one foreign-born parent  |  -.0914572   .0693439    -1.32   0.187    -.2273688    .0444545
2021#girl#uppersecondary#born in Finland foreign-born parents  |  -.1198847   .1321448    -0.91   0.364    -.3788837    .1391144
   2021#girl#uppersecondary#student and parents born abroad  |  -.0929962   .1250104    -0.74   0.457    -.3380121    .1520197
              2021#girl#vocational#one foreign-born parent  |   .0127552   .0898018     0.14   0.887    -.1632531    .1887636
  2021#girl#vocational#born in Finland foreign-born parents  |   .2170818   .1838516     1.18   0.238    -.1432607    .5774242
       2021#girl#vocational#student and parents born abroad  |  -.0531133   .1400099    -0.38   0.704    -.3275277    .2213011
                                                          |
                                                1.degree  |   .0899057   .0030704    29.28   0.000     .0838878    .0959236
                                                          |
                                              urbanrural  |
                                               semiurban  |   .0286189   .0039747     7.20   0.000     .0208286    .0364091
                                                   rural  |   .0637103    .004716    13.51   0.000     .0544671    .0729535
                                                          |
                                                   _cons  |   3.869229   .0062297   621.09   0.000     3.857019    3.881439
-------------------------------------------------------------------------------------------------------------------------------

. testparm year#schoollevel#gender#immigrant

 ( 1)  [belonging]2019.year#2.gender#20.schoollevel#2.immigrant = 0
 ( 2)  [belonging]2019.year#2.gender#20.schoollevel#3.immigrant = 0
 ( 3)  [belonging]2019.year#2.gender#20.schoollevel#4.immigrant = 0
 ( 4)  [belonging]2019.year#2.gender#30.schoollevel#2.immigrant = 0
 ( 5)  [belonging]2019.year#2.gender#30.schoollevel#3.immigrant = 0
 ( 6)  [belonging]2019.year#2.gender#30.schoollevel#4.immigrant = 0
 ( 7)  [belonging]2021.year#2.gender#20.schoollevel#2.immigrant = 0
 ( 8)  [belonging]2021.year#2.gender#20.schoollevel#3.immigrant = 0
 ( 9)  [belonging]2021.year#2.gender#20.schoollevel#4.immigrant = 0
 (10)  [belonging]2021.year#2.gender#30.schoollevel#2.immigrant = 0
 (11)  [belonging]2021.year#2.gender#30.schoollevel#3.immigrant = 0
 (12)  [belonging]2021.year#2.gender#30.schoollevel#4.immigrant = 0

           chi2( 12) =   14.24
         Prob > chi2 =    0.2856

.
.
. glm belonging i.year##i.schoollevel##i.degree i.year##i.gender##i.degree i.urbanrural i.immigrant, family(gaussian) link (identity)
vce(robust)
> nolog

Generalized linear models                         Number of obs   =    415,367
Optimization     : ML                             Residual df     =    415,338
                                                  Scale parameter =   .8954078
Deviance         =  371896.8945                   (1/df) Deviance =   .8954078
Pearson          =  371896.8945                   (1/df) Pearson  =   .8954078

Variance function: V(u) = 1                       [Gaussian]
Link function    : g(u) = u                       [Identity]

                                                  AIC             =   2.727471
Log pseudolikelihood = -566421.6997               BIC             =   -5001297

------------------------------------------------------------------------------------------------------
                                     |               Robust
                           belonging | Coefficient  std. err.      z    P>|z|     [95% conf. interval]
-------------------------------------+----------------------------------------------------------------
                                year |
                                2019 |  -.3329391   .0093084   -35.77   0.000    -.3511832   -.3146949
                                2021 |  -.3395528   .0093269   -36.41   0.000    -.3578333   -.3212724
                                     |
                         schoollevel |
```

```
                    uppersecondary  |  -.0754066    .010176    -7.41   0.000    -.0953512   -.0554619
                        vocational  |   .1541298   .0093472    16.49   0.000     .1358097    .1724499
                                    |
                   year#schoollevel |
               2019#uppersecondary  |   .1870754    .013797    13.56   0.000     .1600338     .214117
                   2019#vocational  |   .0748327   .0128958     5.80   0.000     .0495574    .1001079
               2021#uppersecondary  |   .1048565   .0140028     7.49   0.000     .0774114    .1323015
                   2021#vocational  |   .0669197    .013231     5.06   0.000     .0409874     .092852
                                    |
                           1.degree |   .0678223   .0097395     6.96   0.000     .0487332    .0869113
                                    |
                        year#degree |
                            2019 1  |   .0536301   .0129174     4.15   0.000     .0283125    .0789477
                            2021 1  |   .0214347   .0128367     1.67   0.095    -.0037247    .0465942
                                    |
                 schoollevel#degree |
                 uppersecondary#1   |   .0095998   .0132909     0.72   0.470    -.0164499    .0356495
                     vocational#1   |  -.0200098   .0153844    -1.30   0.193    -.0501626    .0101431
                                    |
            year#schoollevel#degree |
             2019#uppersecondary#1  |  -.0323377   .0176878    -1.83   0.068    -.0670051    .0023297
                 2019#vocational#1  |  -.0689094   .0209577    -3.29   0.001    -.1099857   -.0278331
             2021#uppersecondary#1  |  -.0006348   .0177697    -0.04   0.972    -.0354629    .0341932
                 2021#vocational#1  |  -.0425066   .0213012    -2.00   0.046    -.0842562    -.000757
                                    |
                             gender |
                              girl  |   -.362645   .0079555   -45.58   0.000    -.3782376   -.3470525
                                    |
                        year#gender |
                         2019#girl  |   .0568199   .0108929     5.22   0.000     .0354702    .0781696
                         2021#girl  |  -.0187543    .011012    -1.70   0.089    -.0403375    .0028289
                                    |
                      gender#degree |
                            girl#1  |  -.0187574   .0112459    -1.67   0.095     -.040799    .0032841
                                    |
                 year#gender#degree |
                       2019#girl#1  |   .0186471   .0150247     1.24   0.215    -.0108008     .048095
                       2021#girl#1  |   .0549769   .0150354     3.66   0.000     .0255079    .0844458
                                    |
                         urbanrural |
                         semiurban  |   .0283212   .0039743     7.13   0.000     .0205317    .0361107
                             rural  |   .0648268   .0047196    13.74   0.000     .0555766    .0740771
                                    |
                          immigrant |
              one foreign-born parent  |  -.0574107    .005918    -9.70   0.000    -.0690097   -.0458117
born in Finland foreign-born parents  |   .0356993   .0111874     3.19   0.001     .0137724    .0576261
     student and parents born abroad  |  -.0777953   .0100253    -7.76   0.000    -.0974446   -.0581461
                                    |
                              _cons |   3.850006   .0070375   547.07   0.000     3.836213    3.863799
-----------------------------------------------------------------------------------------------------

. testparm year#schoollevel#degree

 ( 1)  [belonging]2019.year#20.schoollevel#1.degree = 0
 ( 2)  [belonging]2019.year#30.schoollevel#1.degree = 0
 ( 3)  [belonging]2021.year#20.schoollevel#1.degree = 0
 ( 4)  [belonging]2021.year#30.schoollevel#1.degree = 0

           chi2(  4) =   13.53
         Prob > chi2 =    0.0090

. testparm  year#gender#degree

 ( 1)  [belonging]2019.year#2.gender#1.degree = 0
 ( 2)  [belonging]2021.year#2.gender#1.degree = 0

           chi2(  2) =   14.32
         Prob > chi2 =    0.0008

.
. glm belonging i.year##i.schoollevel##i.urbanrural i.year##i.gender##i.urbanrural i.degree i.immigrant, family(gaussian) link (identity)
vce(robu
> st) nolog

Generalized linear models                         Number of obs   =    415,367
Optimization     : ML                             Residual df     =    415,327
                                                  Scale parameter =   .8953342
Deviance         =  371856.4756                   (1/df) Deviance =   .8953342
Pearson          =  371856.4756                   (1/df) Pearson  =   .8953342

Variance function: V(u) = 1                       [Gaussian]
Link function    : g(u) = u                       [Identity]

                                                  AIC             =   2.727415
Log pseudolikelihood = -566399.1268               BIC             =   -5001195

-----------------------------------------------------------------------------------------------------
                                    |               Robust
                          belonging | Coefficient  std. err.      z    P>|z|     [95% conf. interval]
------------------------------------+----------------------------------------------------------------
                               year |
                              2019  |  -.2805265   .0078541   -35.72   0.000    -.2959203   -.2651328
                              2021  |  -.3175821   .0078013   -40.71   0.000    -.3328723   -.3022919
                                    |
                        schoollevel |
                    uppersecondary  |  -.0897471   .0078409   -11.45   0.000     -.105115   -.0743792
                        vocational  |   .1607667   .0085727    18.75   0.000     .1439645     .177569
                                    |
                   year#schoollevel |
               2019#uppersecondary  |   .1657064   .0102116    16.23   0.000      .145692    .1857209
                   2019#vocational  |   .0062067   .0116277     0.53   0.593    -.0165832    .0289965
               2021#uppersecondary  |   .1205191   .0101581    11.86   0.000     .1006097    .1404285
                   2021#vocational  |    .016052   .0118756     1.35   0.176    -.0072238    .0393279
                                    |
                         urbanrural |
                         semiurban  |    .054441   .0126984     4.29   0.000     .0295526    .0793295
                             rural  |   .0871575   .0138314     6.30   0.000     .0600484    .1142666
                                    |
                    year#urbanrural |
                    2019#semiurban  |  -.0750451   .0170231    -4.41   0.000    -.1084098   -.0416804
                        2019#rural  |  -.0726306   .0189007    -3.84   0.000    -.1096753   -.0355859
                    2021#semiurban  |  -.0238109   .0169193    -1.41   0.159    -.0569721    .0093502
```

```
                   2021#rural  |  -.0423711   .0185928    -2.28   0.023    -.0788124   -.0059298
                               |
           schoollevel#urbanrural |
      uppersecondary#semiurban  |   .0514326   .0172371     2.98   0.003     .0176485    .0852167
          uppersecondary#rural  |   .0761744    .019406     3.93   0.000     .0381393    .1142095
          vocational#semiurban  |  -.0124062   .0194392    -0.64   0.523    -.0505063    .0256939
              vocational#rural  |  -.0836517   .0304689    -2.75   0.006    -.1433696   -.0239339
                               |
      year#schoollevel#urbanrural |
 2019#uppersecondary#semiurban  |   .0164299   .0229453     0.72   0.474     -.028542    .0614018
     2019#uppersecondary#rural  |   .0329164    .026387     1.25   0.212    -.0188013    .0846341
     2019#vocational#semiurban  |   .0835696   .0268779     3.11   0.002     .0308898    .1362494
         2019#vocational#rural  |   .2245199   .0412503     5.44   0.000     .1436707     .305369
 2021#uppersecondary#semiurban  |  -.0370106   .0229328    -1.61   0.107    -.0819581     .007937
     2021#uppersecondary#rural  |  -.0444157   .0264402    -1.68   0.093    -.0962376    .0074062
     2021#vocational#semiurban  |    .068941   .0272883     2.53   0.012     .0154569    .1224252
         2021#vocational#rural  |   .1719572   .0418371     4.11   0.000      .089958    .2539564
                               |
                        gender |
                          girl  |   -.365251   .0067612   -54.02   0.000    -.3785027   -.3519992
                               |
                   year#gender |
                     2019#girl  |   .0583797   .0089271     6.54   0.000     .0408829    .0758766
                     2021#girl  |   .0148221   .0089169     1.66   0.096    -.0026547    .0322989
                               |
             gender#urbanrural |
                girl#semiurban  |  -.0275186   .0148683    -1.85   0.064      -.05666    .0016227
                    girl#rural  |  -.0058127     .017055   -0.34   0.733    -.0392398    .0276144
                               |
        year#gender#urbanrural |
           2019#girl#semiurban  |   .0323421   .0200072     1.62   0.106    -.0068714    .0715555
               2019#girl#rural  |   .0076596   .0232922     0.33   0.742    -.0379923    .0533115
           2021#girl#semiurban  |  -.0073355   .0199531    -0.37   0.713    -.0464428    .0317717
               2021#girl#rural  |  -.0317695   .0231586    -1.37   0.170    -.0771595    .0136206
                               |
                      1.degree |   .0887404   .0030703    28.90   0.000     .0827228    .0947581
                               |
                     immigrant |
          one foreign-born parent  |  -.0579069   .0059173    -9.79   0.000    -.0695045   -.0463093
born in Finland foreign-born parents  |   .0333203    .011183     2.98   0.003      .011402    .0552386
     student and parents born abroad  |  -.0794067   .0100201    -7.92   0.000    -.0990458   -.0597677
                               |
                         _cons |   3.831285   .0062787   610.21   0.000     3.818979    3.843591
------------------------------------------------------------------------------------------------------

. testparm year#schoollevel#urbanrural

 ( 1)  [belonging]2019.year#20.schoollevel#2.urbanrural = 0
 ( 2)  [belonging]2019.year#20.schoollevel#3.urbanrural = 0
 ( 3)  [belonging]2019.year#30.schoollevel#2.urbanrural = 0
 ( 4)  [belonging]2019.year#30.schoollevel#3.urbanrural = 0
 ( 5)  [belonging]2021.year#20.schoollevel#2.urbanrural = 0
 ( 6)  [belonging]2021.year#20.schoollevel#3.urbanrural = 0
 ( 7)  [belonging]2021.year#30.schoollevel#2.urbanrural = 0
 ( 8)  [belonging]2021.year#30.schoollevel#3.urbanrural = 0

           chi2(  8) =   54.15
         Prob > chi2 =    0.0000

. testparm  year#gender#urbanrural

 ( 1)  [belonging]2019.year#2.gender#2.urbanrural = 0
 ( 2)  [belonging]2019.year#2.gender#3.urbanrural = 0
 ( 3)  [belonging]2021.year#2.gender#2.urbanrural = 0
 ( 4)  [belonging]2021.year#2.gender#3.urbanrural = 0

           chi2(  4) =    7.55
         Prob > chi2 =    0.1093

.
. glm belonging i.year##i.schoollevel##i.immigrant i.year##i.gender##i.immigrant i.urbanrural i.degree, family(gaussian) link (identity)
vce(robus
> t) nolog

Generalized linear models                         Number of obs   =    415,367
Optimization     : ML                             Residual df     =    415,316
                                                  Scale parameter =   .8952875
Deviance         =  371827.2302                   (1/df) Deviance =   .8952875
Pearson          =  371827.2302                   (1/df) Pearson  =   .8952875

Variance function: V(u) = 1                       [Gaussian]
Link function    : g(u) = u                       [Identity]

                                                  AIC             =   2.727389
Log pseudolikelihood = -566382.7924               BIC             =   -5001082

------------------------------------------------------------------------------------------------------------------------
                                                       |               Robust
                                             belonging | Coefficient  std. err.      z    P>|z|     [95% conf. interval]
-------------------------------------------------------+----------------------------------------------------------------
                                                  year |
                                                  2019  |  -.3084867   .0067693   -45.57   0.000    -.3217544   -.2952191
                                                  2021  |   -.333167   .0067207   -49.57   0.000    -.3463393   -.3199948
                                                       |
                                           schoollevel |
                                      uppersecondary  |  -.0755307    .006807   -11.10   0.000    -.0888721   -.0621893
                                          vocational  |   .1520353   .0076708    19.82   0.000     .1370008    .1670699
                                                       |
                                      year#schoollevel |
                                 2019#uppersecondary  |   .1762486   .0089887    19.61   0.000     .1586311    .1938661
                                     2019#vocational  |   .0370918   .0104992     3.53   0.000     .0165137    .0576698
                                 2021#uppersecondary  |    .117003   .0089673    13.05   0.000     .0994275    .1345785
                                     2021#vocational  |   .0398497   .0107066     3.72   0.000     .0188652    .0608343
                                                       |
                                             immigrant |
                             one foreign-born parent  |  -.0667299     .019781    -3.37   0.001    -.1054999   -.0279599
                born in Finland foreign-born parents  |  -.0703558   .0404063    -1.74   0.082    -.1495508    .0088392
                     student and parents born abroad  |  -.2375899   .0351885    -6.75   0.000    -.3065581   -.1686218
                                                       |
                                        year#immigrant |
                        2019#one foreign-born parent  |  -.0062417   .0260569    -0.24   0.811    -.0573123    .0448289
           2019#born in Finland foreign-born parents  |   .0837518   .0516334     1.62   0.105    -.0174478    .1849515
```

```
                 2019#student and parents born abroad  |   .0764682   .0447258     1.71   0.087    -.0111929    .1641292
                         2021#one foreign-born parent  |  -.0182491   .0258335    -0.71   0.480    -.0688817    .0323835
            2021#born in Finland foreign-born parents  |   .1051783    .050093     2.10   0.036     .0069979    .2033587
                 2021#student and parents born abroad  |   .1079096   .0436963     2.47   0.014     .0222664    .1935527
                                                       |
                                    schoollevel#immigrant |
               uppersecondary#one foreign-born parent  |  -.0197886   .0271051    -0.73   0.465    -.0729136    .0333364
  uppersecondary#born in Finland foreign-born parents  |    .057533   .0531741     1.08   0.279    -.0466863    .1617523
       uppersecondary#student and parents born abroad  |   .0474217   .0508197     0.93   0.351    -.0521831    .1470265
                   vocational#one foreign-born parent  |  -.0006282   .0331276    -0.02   0.985    -.0655572    .0643008
      vocational#born in Finland foreign-born parents  |   .0134209   .0678656     0.20   0.843    -.1195931     .146435
           vocational#student and parents born abroad  |  -.0286843   .0523148    -0.55   0.583    -.1312195    .0738509
                                                       |
                               year#schoollevel#immigrant |
          2019#uppersecondary#one foreign-born parent  |   .0443475   .0348589     1.27   0.203    -.0239748    .1126698
2019#uppersecondary#born in Finland foreign-born parents  |  -.1490825   .0668615    -2.23   0.026    -.2801286   -.0180364
     2019#uppersecondary#student and parents born abroad  |  -.0556909   .0628718    -0.89   0.376    -.1789174    .0675355
              2019#vocational#one foreign-born parent  |  -.0111778   .0438996    -0.25   0.799    -.0972194    .0748639
    2019#vocational#born in Finland foreign-born parents  |  -.0340349   .0864922    -0.39   0.694    -.2035564    .1354866
         2019#vocational#student and parents born abroad  |   .1319404   .0686963     1.92   0.055    -.0027019    .2665826
          2021#uppersecondary#one foreign-born parent  |   .0305147   .0344089     0.89   0.375    -.0369254    .0979549
2021#uppersecondary#born in Finland foreign-born parents  |  -.1672899   .0655375    -2.55   0.011    -.2957411   -.0388387
     2021#uppersecondary#student and parents born abroad  |  -.0921793   .0619528    -1.49   0.137    -.2136046     .029246
              2021#vocational#one foreign-born parent  |   .0217088   .0446933     0.49   0.627    -.0658885    .1093061
    2021#vocational#born in Finland foreign-born parents  |  -.0804646   .0915384    -0.88   0.379    -.2598766    .0989474
         2021#vocational#student and parents born abroad  |   .1127407   .0694435     1.62   0.104    -.0233661    .2488474
                                                       |
                                                gender |
                                                  girl  |  -.3795702   .0058797   -64.56   0.000    -.3910942   -.3680463
                                                       |
                                           year#gender |
                                             2019#girl  |   .0663846   .0078678     8.44   0.000      .050964    .0818053
                                             2021#girl  |   .0067657   .0078616     0.86   0.389    -.0086428    .0221741
                                                       |
                                      gender#immigrant |
                          girl#one foreign-born parent  |   .0258925   .0234488     1.10   0.269    -.0200662    .0718513
             girl#born in Finland foreign-born parents  |   .0640532   .0473563     1.35   0.176    -.0287633    .1568698
                  girl#student and parents born abroad  |   .1946337   .0417174     4.67   0.000     .1128691    .2763982
                                                       |
                                 year#gender#immigrant |
                     2019#girl#one foreign-born parent  |  -.0088892   .0306305    -0.29   0.772    -.0689238    .0511454
        2019#girl#born in Finland foreign-born parents  |   .0341286   .0599578     0.57   0.569    -.0833866    .1516439
             2019#girl#student and parents born abroad  |  -.0507017    .053217    -0.95   0.341    -.1550051    .0536016
                     2021#girl#one foreign-born parent  |   .0138981   .0304077     0.46   0.648    -.0456998    .0734961
        2021#girl#born in Finland foreign-born parents  |   .0648374   .0589625     1.10   0.271     -.050727    .1804019
             2021#girl#student and parents born abroad  |   .0038373   .0521615     0.07   0.941    -.0983973    .1060719
                                                       |
                                            urbanrural |
                                             semiurban  |   .0281592   .0039738     7.09   0.000     .0203706    .0359477
                                                 rural  |   .0637896   .0047166    13.52   0.000     .0545453    .0730339
                                                       |
                                              1.degree |   .0890047   .0030693    29.00   0.000      .082989    .0950204
                                                 _cons |   3.847404   .0055161   697.49   0.000     3.836592    3.858215
--------------------------------------------------------------------------------------------------------------------------

. testparm year#schoollevel#immigrant

 ( 1)  [belonging]2019.year#20.schoollevel#2.immigrant = 0
 ( 2)  [belonging]2019.year#20.schoollevel#3.immigrant = 0
 ( 3)  [belonging]2019.year#20.schoollevel#4.immigrant = 0
 ( 4)  [belonging]2019.year#30.schoollevel#2.immigrant = 0
 ( 5)  [belonging]2019.year#30.schoollevel#3.immigrant = 0
 ( 6)  [belonging]2019.year#30.schoollevel#4.immigrant = 0
 ( 7)  [belonging]2021.year#20.schoollevel#2.immigrant = 0
 ( 8)  [belonging]2021.year#20.schoollevel#3.immigrant = 0
 ( 9)  [belonging]2021.year#20.schoollevel#4.immigrant = 0
 (10)  [belonging]2021.year#30.schoollevel#2.immigrant = 0
 (11)  [belonging]2021.year#30.schoollevel#3.immigrant = 0
 (12)  [belonging]2021.year#30.schoollevel#4.immigrant = 0

           chi2( 12) =   18.53
         Prob > chi2 =    0.1004

. testparm  year#gender#immigrant

 ( 1)  [belonging]2019.year#2.gender#2.immigrant = 0
 ( 2)  [belonging]2019.year#2.gender#3.immigrant = 0
 ( 3)  [belonging]2019.year#2.gender#4.immigrant = 0
 ( 4)  [belonging]2021.year#2.gender#2.immigrant = 0
 ( 5)  [belonging]2021.year#2.gender#3.immigrant = 0
 ( 6)  [belonging]2021.year#2.gender#4.immigrant = 0

           chi2(  6) =    3.44
         Prob > chi2 =    0.7517

.
. glm belonging i.year##i.immigrant i.schoollevel i.gender i.urbanrural i.degree, family(gaussian) link (identity) vce(robust) nolog

Generalized linear models                         Number of obs   =    415,367
Optimization     : ML                             Residual df     =    415,349
                                                  Scale parameter =   .8967718
Deviance         =  372473.2584                   (1/df) Deviance =   .8967718
Pearson          =  372473.2584                   (1/df) Pearson  =   .8967718

Variance function: V(u) = 1                       [Gaussian]
Link function    : g(u) = u                       [Identity]

                                                  AIC             =   2.728967
Log pseudolikelihood = -566743.3174               BIC             =   -5000863

------------------------------------------------------------------------------------------------------------
                                           |               Robust
                                 belonging | Coefficient  std. err.      z    P>|z|     [95% conf. interval]
-------------------------------------------+----------------------------------------------------------------
                                      year |
                                      2019  |  -.2158267   .0039053   -55.26   0.000     -.223481   -.2081724
                                      2021  |  -.2897577   .0039093   -74.12   0.000    -.2974198   -.2820956
                                           |
                                 immigrant |
                   one foreign-born parent  |  -.0556032   .0116971    -4.75   0.000     -.078529   -.0326773
      born in Finland foreign-born parents  |  -.0165054   .0235576    -0.70   0.484    -.0626774    .0296665
           student and parents born abroad  |  -.1349596   .0209101    -6.45   0.000    -.1759426   -.0939767
```

```
                                          |
                           year#immigrant |
              2019#one foreign-born parent  |  -.0013229   .0152414    -0.09   0.931    -.0311955    .0285496
  2019#born in Finland foreign-born parents |   .0531329    .029715     1.79   0.074    -.0051073    .1113732
       2019#student and parents born abroad |   .0515706   .0265947     1.94   0.052    -.0005542    .1036953
              2021#one foreign-born parent  |  -.0021507   .0151232    -0.14   0.887    -.0317916    .0274901
  2021#born in Finland foreign-born parents |    .082569   .0293105     2.82   0.005     .0251216    .1400165
       2021#student and parents born abroad |   .1017167   .0260998     3.90   0.000      .050562    .1528713
                                          |
                              schoollevel |
                         uppersecondary  |   .0294431   .0033846     8.70   0.000     .0228094    .0360769
                             vocational  |   .1818955   .0041804    43.51   0.000     .1737021     .190089
                                          |
                                   gender |
                                    girl  |  -.3444851   .0029671  -116.10   0.000    -.3503006   -.3386696
                                          |
                               urbanrural |
                               semiurban  |   .0279187   .0039766     7.02   0.000     .0201246    .0357127
                                   rural  |   .0638291   .0047189    13.53   0.000     .0545802     .073078
                                          |
                                 1.degree |   .0888917   .0030713    28.94   0.000     .0828721    .0949112
                                    _cons |   3.794052   .0041753   908.69   0.000     3.785869    3.802236
--------------------------------------------------------------------------------------------------------------

. testparm year#immigrant

 ( 1)  [belonging]2019.year#2.immigrant = 0
 ( 2)  [belonging]2019.year#3.immigrant = 0
 ( 3)  [belonging]2019.year#4.immigrant = 0
 ( 4)  [belonging]2021.year#2.immigrant = 0
 ( 5)  [belonging]2021.year#3.immigrant = 0
 ( 6)  [belonging]2021.year#4.immigrant = 0

           chi2(  6) =   23.28
         Prob > chi2 =    0.0007

.
.

. glm belonging i.year##i.schoollevel##i.degree i.year##i.gender##i.degree i.year##i.immigrant i.year##i.schoollevel##i.urbanrural,
family(gaussia
> n) link (identity) vce(robust) nolog

Generalized linear models                         Number of obs   =    415,367
Optimization     : ML                             Residual df     =    415,316
                                                  Scale parameter =   .8950893
Deviance         =  371744.9054                   (1/df) Deviance =   .8950893
Pearson          =  371744.9054                   (1/df) Pearson  =   .8950893

Variance function: V(u) = 1                       [Gaussian]
Link function    : g(u) = u                       [Identity]

                                                  AIC             =   2.727168
Log pseudolikelihood =  -566336.805               BIC             =   -5001164

--------------------------------------------------------------------------------------------------------------
                                          |               Robust
                                belonging | Coefficient  std. err.      z    P>|z|     [95% conf. interval]
------------------------------------------+-------------------------------------------------------------------
                                     year |
                                    2019  |  -.3147252   .0105399   -29.86   0.000     -.335383   -.2940673
                                    2021  |  -.3329136    .010534   -31.60   0.000    -.3535599   -.3122673
                                          |
                              schoollevel |
                         uppersecondary  |  -.0986425   .0116083    -8.50   0.000    -.1213944   -.0758905
                             vocational  |   .1607999   .0105755    15.20   0.000     .1400723    .1815274
                                          |
                         year#schoollevel |
                    2019#uppersecondary  |   .1785996   .0155759    11.47   0.000     .1480713    .2091279
                        2019#vocational  |   .0418754   .0145754     2.87   0.004     .0133081    .0704426
                    2021#uppersecondary  |   .1191582   .0157541     7.56   0.000     .0882808    .1500356
                        2021#vocational  |   .0414709   .0149537     2.77   0.006     .0121622    .0707796
                                          |
                                 1.degree |   .0685403    .009817     6.98   0.000     .0492994    .0877812
                                          |
                              year#degree |
                                  2019 1  |   .0463362   .0130489     3.55   0.000     .0207608    .0719117
                                  2021 1  |   .0179855   .0129533     1.39   0.165    -.0074025    .0433736
                                          |
                       schoollevel#degree |
                       uppersecondary#1  |   .0181007   .0134409     1.35   0.178     -.008243    .0444444
                           vocational#1  |  -.0215174   .0154448    -1.39   0.164    -.0517886    .0087538
                                          |
                  year#schoollevel#degree |
                  2019#uppersecondary#1  |  -.0282765   .0178886    -1.58   0.114    -.0633375    .0067845
                      2019#vocational#1  |  -.0581343   .0210631    -2.76   0.006    -.0994172   -.0168514
                  2021#uppersecondary#1  |  -.0052516     .01797    -0.29   0.770    -.0404721     .029969
                      2021#vocational#1  |   -.035034   .0214075    -1.64   0.102     -.076992     .006924
                                          |
                                   gender |
                                    girl  |  -.3627912   .0079527   -45.62   0.000    -.3783782   -.3472042
                                          |
                              year#gender |
                               2019#girl  |   .0567969   .0108905     5.22   0.000     .0354519    .0781419
                               2021#girl  |  -.0180498   .0110094    -1.64   0.101    -.0396278    .0035281
                                          |
                            gender#degree |
                                 girl#1  |   -.018658   .0112423    -1.66   0.097    -.0406924    .0033764
                                          |
                       year#gender#degree |
                            2019#girl#1  |   .0188125   .0150212     1.25   0.210    -.0106285    .0482534
                            2021#girl#1  |   .0545961    .015032     3.63   0.000      .025134    .0840583
                                          |
                                immigrant |
                   one foreign-born parent  |   -.054491   .0116935    -4.66   0.000    -.0774099   -.0315721
       born in Finland foreign-born parents |  -.0127943   .0236051    -0.54   0.588    -.0590595    .0334709
            student and parents born abroad |   -.143966   .0209525    -6.87   0.000     -.185032   -.1028999
                                          |
                           year#immigrant |
              2019#one foreign-born parent  |  -.0045399   .0152426    -0.30   0.766    -.0344149    .0253351
  2019#born in Finland foreign-born parents |   .0471225   .0297962     1.58   0.114    -.0112769    .1055219
       2019#student and parents born abroad |   .0655432   .0266328     2.46   0.014     .0133439    .1177424
```

```
            2021#one foreign-born parent  |  -.0036396   .0151305    -0.24   0.810    -.0332949    .0260157
2021#born in Finland foreign-born parents  |   .0794799   .0294112     2.70   0.007     .0218349    .1371248
     2021#student and parents born abroad  |   .1108265   .0261666     4.24   0.000     .0595409    .1621122
                                          |
                              urbanrural |
                               semiurban  |   .0339197   .0101939     3.33   0.001     .0139401    .0538993
                                   rural  |   .0764766   .0109802     6.96   0.000     .0549557    .0979975
                                          |
                         year#urbanrural |
                          2019#semiurban  |  -.0483379   .0136382    -3.54   0.000    -.0750682   -.0216076
                              2019#rural  |  -.0555536   .0149771    -3.71   0.000    -.0849082   -.0261989
                          2021#semiurban  |  -.0178052    .013506    -1.32   0.187    -.0442765    .0086662
                              2021#rural  |  -.0462629   .0147679    -3.13   0.002    -.0752075   -.0173183
                                          |
                  schoollevel#urbanrural |
              uppersecondary#semiurban  |   .0523826   .0172952     3.03   0.002     .0184847    .0862805
                  uppersecondary#rural  |   .0793463   .0195118     4.07   0.000     .0411039    .1175886
                  vocational#semiurban  |  -.0061017    .019296    -0.32   0.752    -.0439212    .0317178
                      vocational#rural  |  -.0806281   .0304829    -2.65   0.008    -.1403734   -.0208827
                                          |
             year#schoollevel#urbanrural |
         2019#uppersecondary#semiurban  |    .014311   .0230224     0.62   0.534    -.0308121    .0594341
             2019#uppersecondary#rural  |   .0275945    .026523     1.04   0.298    -.0243896    .0795786
             2019#vocational#semiurban  |   .0678237   .0267221     2.54   0.011     .0154494     .120198
                 2019#vocational#rural  |   .2101786   .0412891     5.09   0.000     .1292534    .2911038
         2021#uppersecondary#semiurban  |  -.0384344   .0230191    -1.67   0.095    -.0835509    .0066822
             2021#uppersecondary#rural  |  -.0483811   .0265482    -1.82   0.068    -.1004146    .0036524
             2021#vocational#semiurban  |   .0591903   .0271445     2.18   0.029     .0059881    .1123925
                 2021#vocational#rural  |   .1620922   .0418838     3.87   0.000     .0800015    .2441829
                                          |
                                   _cons |   3.849526   .0078445   490.73   0.000     3.834151    3.864901
------------------------------------------------------------------------------------------------------------

. testparm year#schoollevel#degree

 ( 1)  [belonging]2019.year#20.schoollevel#1.degree = 0
 ( 2)  [belonging]2019.year#30.schoollevel#1.degree = 0
 ( 3)  [belonging]2021.year#20.schoollevel#1.degree = 0
 ( 4)  [belonging]2021.year#30.schoollevel#1.degree = 0

          chi2(  4) =    8.99
        Prob > chi2 =    0.0614

. testparm  year#gender#degree

 ( 1)  [belonging]2019.year#2.gender#1.degree = 0
 ( 2)  [belonging]2021.year#2.gender#1.degree = 0

          chi2(  2) =   14.08
        Prob > chi2 =    0.0009

. testparm year#schoollevel#urbanrural

 ( 1)  [belonging]2019.year#20.schoollevel#2.urbanrural = 0
 ( 2)  [belonging]2019.year#20.schoollevel#3.urbanrural = 0
 ( 3)  [belonging]2019.year#30.schoollevel#2.urbanrural = 0
 ( 4)  [belonging]2019.year#30.schoollevel#3.urbanrural = 0
 ( 5)  [belonging]2021.year#20.schoollevel#2.urbanrural = 0
 ( 6)  [belonging]2021.year#20.schoollevel#3.urbanrural = 0
 ( 7)  [belonging]2021.year#30.schoollevel#2.urbanrural = 0
 ( 8)  [belonging]2021.year#30.schoollevel#3.urbanrural = 0

          chi2(  8) =   47.87
        Prob > chi2 =    0.0000

. testparm year#immigrant

 ( 1)  [belonging]2019.year#2.immigrant = 0
 ( 2)  [belonging]2019.year#3.immigrant = 0
 ( 3)  [belonging]2019.year#4.immigrant = 0
 ( 4)  [belonging]2021.year#2.immigrant = 0
 ( 5)  [belonging]2021.year#3.immigrant = 0
 ( 6)  [belonging]2021.year#4.immigrant = 0

          chi2(  6) =   25.08
        Prob > chi2 =    0.0003



***Final model****

. glm belonging i.year##i.gender##i.degree i.year##i.immigrant i.year##i.schoollevel##i.urbanrural, family(gaussian) link (identity)
vce(robust) n
> olog

Generalized linear models                         Number of obs   =    415,367
Optimization     : ML                             Residual df     =    415,322
                                                  Scale parameter =   .8951855
Deviance         =  371790.2197                   (1/df) Deviance =   .8951855
Pearson          =  371790.2197                   (1/df) Pearson  =   .8951855

Variance function: V(u) = 1                       [Gaussian]
Link function    : g(u) = u                       [Identity]

                                                  AIC             =   2.727261
Log pseudolikelihood = -566362.1193               BIC             =   -5001196

------------------------------------------------------------------------------------------------------------
                                         |               Robust
                               belonging | Coefficient  std. err.      z    P>|z|     [95% conf. interval]
-----------------------------------------+------------------------------------------------------------------
                                    year |
                                    2019  |  -.3053881   .0097538   -31.31   0.000    -.3245051    -.286271
                                    2021  |  -.3292969   .0097716   -33.70   0.000    -.3484488   -.3101449
                                          |
                                  gender |
                                    girl  |  -.3643941   .0079091   -46.07   0.000    -.3798957   -.3488925
                                          |
                             year#gender |
                               2019#girl  |   .0564429   .0108246     5.21   0.000     .0352271    .0776587
                               2021#girl  |   -.018939   .0109433    -1.73   0.084    -.0403876    .0025095
                                          |
```

```
                               1.degree |   .0679128   .0081379     8.35   0.000     .0519628    .0838629
                                        |
                            year#degree |
                                 2019 1 |   .0301079   .0109088     2.76   0.006     .0087269    .0514888
                                 2021 1 |   .0120336   .0109111     1.10   0.270    -.0093519     .033419
                                        |
                          gender#degree |
                                 girl#1 |  -.0156364   .0111319    -1.40   0.160    -.0374547    .0061818
                                        |
                     year#gender#degree |
                            2019#girl#1 |   .0202086   .0148708     1.36   0.174    -.0089376    .0493548
                            2021#girl#1 |    .055896   .0148865     3.75   0.000      .026719    .0850729
                                        |
                              immigrant |
                  one foreign-born parent |  -.0547131   .0116937    -4.68   0.000    -.0776323   -.0317939
    born in Finland foreign-born parents |  -.0135615   .0236046    -0.57   0.566    -.0598257    .0327028
         student and parents born abroad |  -.1444653   .0209523    -6.89   0.000     -.185531   -.1033995
                                        |
                         year#immigrant |
             2019#one foreign-born parent |  -.0046127   .0152427    -0.30   0.762    -.0344877    .0252624
2019#born in Finland foreign-born parents |   .0471466   .0297932     1.58   0.114    -.0112471    .1055403
     2019#student and parents born abroad |    .065116     .02663     2.45   0.014     .0129222    .1173098
             2021#one foreign-born parent |  -.0035395   .0151309    -0.23   0.815    -.0331954    .0261165
2021#born in Finland foreign-born parents |    .079337   .0294104     2.70   0.007     .0216936    .1369804
     2021#student and parents born abroad |   .1102693   .0261643     4.21   0.000     .0589883    .1615503
                                        |
                            schoollevel |
                         uppersecondary |  -.0866097   .0078607   -11.02   0.000    -.1020164   -.0712031
                             vocational |   .1536111   .0086338    17.79   0.000     .1366892     .170533
                                        |
                       year#schoollevel |
                    2019#uppersecondary |   .1610388   .0102469    15.72   0.000     .1409551    .1811224
                        2019#vocational |   .0159963   .0117541     1.36   0.174    -.0070413    .0390339
                    2021#uppersecondary |   .1172002   .0101991    11.49   0.000     .0972103    .1371902
                        2021#vocational |   .0256947   .0119996     2.14   0.032     .0021758    .0492136
                                        |
                             urbanrural |
                              semiurban |    .034015   .0101616     3.35   0.001     .0140987    .0539313
                                  rural |   .0766209   .0109245     7.01   0.000     .0552093    .0980325
                                        |
                        year#urbanrural |
                         2019#semiurban |  -.0505095   .0135966    -3.71   0.000    -.0771583   -.0238607
                             2019#rural |  -.0587615   .0149012    -3.94   0.000    -.0879674   -.0295556
                         2021#semiurban |   -.018507   .0134671    -1.37   0.169     -.044902     .007888
                             2021#rural |  -.0473066   .0147009    -3.22   0.001    -.0761199   -.0184932
                                        |
                 schoollevel#urbanrural |
               uppersecondary#semiurban |   .0500718   .0172023     2.91   0.004      .016356    .0837876
                   uppersecondary#rural |   .0758928    .019351     3.92   0.000     .0379656      .11382
                   vocational#semiurban |  -.0048197   .0192594    -0.25   0.802    -.0425674     .032928
                       vocational#rural |  -.0791309   .0304419    -2.60   0.009    -.1387959    -.019466
                                        |
            year#schoollevel#urbanrural |
          2019#uppersecondary#semiurban |   .0181522   .0229022     0.79   0.428    -.0267353    .0630397
              2019#uppersecondary#rural |     .03329   .0263051     1.27   0.206    -.0182672    .0848471
              2019#vocational#semiurban |   .0746026   .0266555     2.80   0.005     .0223588    .1268464
                  2019#vocational#rural |   .2187153   .0412249     5.31   0.000      .137916    .2995146
          2021#uppersecondary#semiurban |  -.0375194   .0228944    -1.64   0.101    -.0823915    .0073527
              2021#uppersecondary#rural |   -.047373   .0263393    -1.80   0.072     -.098997     .004251
              2021#vocational#semiurban |   .0636591   .0270738     2.35   0.019     .0105953    .1167228
                  2021#vocational#rural |   .1665557    .041827     3.98   0.000     .0845762    .2485352
                                        |
                                  _cons |   3.849917   .0072617   530.17   0.000     3.835684    3.864149
------------------------------------------------------------------------------------------------------------

. testparm  year#gender#degree

 ( 1)  [belonging]2019.year#2.gender#1.degree = 0
 ( 2)  [belonging]2021.year#2.gender#1.degree = 0

           chi2(  2) =   14.90
         Prob > chi2 =    0.0006

. testparm year#schoollevel#urbanrural

 ( 1)  [belonging]2019.year#20.schoollevel#2.urbanrural = 0
 ( 2)  [belonging]2019.year#20.schoollevel#3.urbanrural = 0
 ( 3)  [belonging]2019.year#30.schoollevel#2.urbanrural = 0
 ( 4)  [belonging]2019.year#30.schoollevel#3.urbanrural = 0
 ( 5)  [belonging]2021.year#20.schoollevel#2.urbanrural = 0
 ( 6)  [belonging]2021.year#20.schoollevel#3.urbanrural = 0
 ( 7)  [belonging]2021.year#30.schoollevel#2.urbanrural = 0
 ( 8)  [belonging]2021.year#30.schoollevel#3.urbanrural = 0

           chi2(  8) =   52.42
         Prob > chi2 =    0.0000

. testparm year#immigrant

 ( 1)  [belonging]2019.year#2.immigrant = 0
 ( 2)  [belonging]2019.year#3.immigrant = 0
 ( 3)  [belonging]2019.year#4.immigrant = 0
 ( 4)  [belonging]2021.year#2.immigrant = 0
 ( 5)  [belonging]2021.year#3.immigrant = 0
 ( 6)  [belonging]2021.year#4.immigrant = 0

           chi2(  6) =   24.88
         Prob > chi2 =    0.0004

.
. margins  year#gender#degree

Predictive margins                                     Number of obs = 415,367
Model VCE: Robust

Expression: Predicted mean belonging, predict()

--------------------------------------------------------------------------------
                |            Delta-method
                |     Margin   std. err.      z    P>|z|     [95% conf. interval]
----------------+---------------------------------------------------------------
year#gender#degree |
```

```
    2017#boy#0  |   3.858758   .0058255   662.39   0.000      3.84734    3.870176
    2017#boy#1  |   3.926671   .0056591   693.87   0.000     3.915579    3.937762
   2017#girl#0  |   3.494364   .0055222   632.78   0.000     3.483541    3.505187
   2017#girl#1  |    3.54664   .0055677   637.00   0.000     3.535728    3.557553
    2019#boy#0  |   3.595945   .0053983   666.12   0.000     3.585365    3.606526
    2019#boy#1  |   3.693966   .0047751   773.59   0.000     3.684607    3.703325
   2019#girl#0  |   3.287994   .0051156   642.74   0.000     3.277968    3.298021
   2019#girl#1  |   3.390587   .0046299   732.32   0.000     3.381513    3.399662
    2021#boy#0  |   3.563798   .0054966   648.36   0.000     3.553025    3.574571
    2021#boy#1  |   3.643744   .0046573   782.38   0.000     3.634616    3.652872
   2021#girl#0  |   3.180465   .0052554   605.18   0.000     3.170164    3.190765
   2021#girl#1  |   3.300671   .0045214   730.00   0.000     3.291809    3.309532
--------------------------------------------------------------------------------

. margins year#schoollevel#urbanrural

Predictive margins                                     Number of obs = 415,367
Model VCE: Robust

Expression: Predicted mean belonging, predict()

------------------------------------------------------------------------------------------------
                               |            Delta-method
                               |     Margin   std. err.      z    P>|z|     [95% conf. interval]
-------------------------------+----------------------------------------------------------------
   year#schoollevel#urbanrural |
    2017#lowersecondary#urban  |   3.681103   .0048682   756.16   0.000     3.671562    3.690645
2017#lowersecondary#semiurban  |   3.715118   .0089084   417.03   0.000     3.697658    3.732578
    2017#lowersecondary#rural  |   3.757724   .0097697   384.63   0.000     3.738576    3.776872
    2017#uppersecondary#urban  |   3.594493   .0061718   582.41   0.000     3.582397     3.60659
2017#uppersecondary#semiurban  |    3.67858   .0125402   293.34   0.000     3.654002    3.703158
    2017#uppersecondary#rural  |   3.747007   .0148401   252.49   0.000     3.717921    3.776093
        2017#vocational#urban  |   3.834714   .0071177   538.76   0.000     3.820764    3.848665
    2017#vocational#semiurban  |    3.86391   .0149659   258.18   0.000     3.834577    3.893242
        2017#vocational#rural  |   3.832204   .0276185   138.75   0.000     3.778073    3.886336
    2019#lowersecondary#urban  |   3.430269   .0040971   837.25   0.000     3.422239    3.438299
2019#lowersecondary#semiurban  |   3.413775   .0080238   425.46   0.000     3.398048    3.429501
    2019#lowersecondary#rural  |   3.448129   .0092353   373.36   0.000     3.430028     3.46623
    2019#uppersecondary#urban  |   3.504698   .0051889   675.43   0.000     3.494528    3.514868
2019#uppersecondary#semiurban  |   3.556428   .0110602   321.55   0.000      3.53475    3.578105
    2019#uppersecondary#rural  |   3.631741   .0138107   262.97   0.000     3.604672    3.658809
        2019#vocational#urban  |   3.599877   .0068052   528.99   0.000     3.586539    3.613215
    2019#vocational#semiurban  |   3.653165   .0147224   248.14   0.000      3.62431    3.682021
        2019#vocational#rural  |    3.75732   .0250773   149.83   0.000      3.70817    3.806471
    2021#lowersecondary#urban  |   3.368952   .0040322   835.51   0.000     3.361049    3.376855
2021#lowersecondary#semiurban  |    3.38446   .0078312   432.18   0.000     3.369111    3.399808
    2021#lowersecondary#rural  |   3.398266   .0089365   380.27   0.000     3.380751    3.415781
    2021#uppersecondary#urban  |   3.399542   .0051682   657.78   0.000     3.389413    3.409672
2021#uppersecondary#semiurban  |   3.427603   .0112248   305.36   0.000     3.405602    3.449603
    2021#uppersecondary#rural  |   3.457376   .0140978   245.24   0.000     3.429745    3.485008
        2021#vocational#urban  |   3.548258    .007252   489.28   0.000     3.534044    3.562471
    2021#vocational#semiurban  |   3.622605   .0153614   235.83   0.000     3.592497    3.652713
        2021#vocational#rural  |   3.664997   .0260307   140.80   0.000     3.613977    3.716016
------------------------------------------------------------------------------------------------

. margins year#immigrant

Predictive margins                                     Number of obs = 415,367
Model VCE: Robust

Expression: Predicted mean belonging, predict()

------------------------------------------------------------------------------------------------------------
                                           |            Delta-method
                                           |     Margin   std. err.      z    P>|z|     [95% conf. interval]
-------------------------------------------+----------------------------------------------------------------
                            year#immigrant |
 2017#student and parents born in Finland  |   3.706979   .0029426  1259.74   0.000     3.701211    3.712746
              2017#one foreign-born parent |   3.652266   .0113078   322.99   0.000     3.630103    3.674429
2017#born in Finland foreign-born parents  |   3.693417   .0234125   157.75   0.000      3.64753    3.739305
     2017#student and parents born abroad  |   3.562514   .0207475   171.71   0.000     3.521849    3.603178
 2019#student and parents born in Finland  |   3.493595   .0025881  1349.89   0.000     3.488522    3.498667
              2019#one foreign-born parent |   3.434269    .009426   364.34   0.000     3.415794    3.452744
2019#born in Finland foreign-born parents  |    3.52718   .0179877   196.09   0.000     3.491925    3.562435
     2019#student and parents born abroad  |   3.414245   .0162274   210.40   0.000      3.38244    3.446051
 2021#student and parents born in Finland  |   3.419895    .002592  1319.38   0.000     3.414814    3.424975
              2021#one foreign-born parent |   3.361642   .0092492   363.45   0.000     3.343514     3.37977
2021#born in Finland foreign-born parents  |    3.48567   .0173438   200.98   0.000     3.451677    3.519664
     2021#student and parents born abroad  |   3.385699    .015449   219.15   0.000     3.355419    3.415978
------------------------------------------------------------------------------------------------------------
```

4. Multivariable regressions with marginal effects

4.1. Close friends with Odds Ratios and marginal effects

```
. ologit closefriend i.year i.gender i.year i.schoollevel i.degree i.urbanrural i.immigrant, vce(robust) nolog or

Ordered logistic regression                          Number of obs = 415,932
                                                     Wald chi2(11) = 4036.20
                                                     Prob > chi2   =  0.0000
Log pseudolikelihood = -486744.17                    Pseudo R2     =  0.0042

---------------------------------------------------------------------------------------------------------
                                      |               Robust
                          closefriend | Odds ratio   std. err.      z    P>|z|     [95% conf. interval]
--------------------------------------+------------------------------------------------------------------
                                 year |
                                 2019  |    .943022   .0070849    -7.81   0.000     .9292375     .957011
                                 2021  |   .7986718   .0058325   -30.78   0.000     .7873216    .8101855
                                      |
                               gender |
                                 girl  |   .8121809   .0049266   -34.30   0.000     .8025822    .8218944
                                      |
                          schoollevel |
                       uppersecondary  |   1.041182   .0070578     5.95   0.000      1.02744    1.055107
                           vocational  |    1.04131   .0089572     4.71   0.000     1.023902    1.059015
                                      |
                             1.degree |   1.148069   .0070108    22.61   0.000      1.13441    1.161892
                                      |
                           urbanrural |
                            semiurban  |   .9681785   .0077134    -4.06   0.000     .9531779    .9834151
                                rural  |   .9146484    .008471    -9.63   0.000     .8981953    .9314029
                                      |
                            immigrant |
              one parent foreign-born  |   .8639798   .0097997   -12.89   0.000     .8449846    .8834019
born in Finland parents foreign-born  |   .7847356   .0167496   -11.36   0.000     .7525841    .8182607
     student and parents born abroad  |   .5979846   .0108514   -28.34   0.000       .57709    .6196357
--------------------------------------+------------------------------------------------------------------
                                /cut1 |  -2.622885   .0099498                     -2.642386   -2.603384
                                /cut2 |  -1.206868   .0086493                      -1.22382   -1.189916
                                /cut3 |  -.2915825   .0083454                     -.3079392   -.2752259
---------------------------------------------------------------------------------------------------------
Note: Estimates are transformed only in the first equation to odds ratios.

. margins, dydx(year) vsquish

Average marginal effects                             Number of obs = 415,932
Model VCE: Robust

dy/dx wrt: 2019.year 2021.year

1._predict: Pr(closefriend==1), predict(pr outcome(1))
2._predict: Pr(closefriend==2), predict(pr outcome(2))
3._predict: Pr(closefriend==3), predict(pr outcome(3))
4._predict: Pr(closefriend==4), predict(pr outcome(4))

------------------------------------------------------------------------------
             |            Delta-method
             |      dy/dx   std. err.      z    P>|z|     [95% conf. interval]
-------------+----------------------------------------------------------------
2017.year    |  (base outcome)
-------------+----------------------------------------------------------------
2019.year    |
    _predict |
          1  |   .0040425   .0005173     7.81   0.000     .0030286    .0050563
          2  |   .0068524    .000877     7.81   0.000     .0051335    .0085714
          3  |    .003532   .0004537     7.79   0.000     .0026428    .0044212
          4  |  -.0144269   .0018469    -7.81   0.000    -.0180468   -.0108071
-------------+----------------------------------------------------------------
2021.year    |
    _predict |
          1  |   .0166297   .0005384    30.89   0.000     .0155745    .0176849
          2  |   .0268124   .0008703    30.81   0.000     .0251067    .0285182
          3  |   .0121444   .0004085    29.73   0.000     .0113437    .0129451
          4  |  -.0555866   .0018004   -30.88   0.000    -.0591152   -.0520579
------------------------------------------------------------------------------
Note: dy/dx for factor levels is the discrete change from the base level.

. margins, dydx(gender) vsquish

Average marginal effects                             Number of obs = 415,932
Model VCE: Robust

dy/dx wrt: 2.gender

1._predict: Pr(closefriend==1), predict(pr outcome(1))
2._predict: Pr(closefriend==2), predict(pr outcome(2))
3._predict: Pr(closefriend==3), predict(pr outcome(3))
4._predict: Pr(closefriend==4), predict(pr outcome(4))

------------------------------------------------------------------------------
             |            Delta-method
             |      dy/dx   std. err.      z    P>|z|     [95% conf. interval]
-------------+----------------------------------------------------------------
1.gender        |  (base outcome)
-------------+----------------------------------------------------------------
2.gender        |
    _predict |
          1  |   .0151648   .0004312    35.17   0.000     .0143196    .0160101
          2  |   .0247263   .0007247    34.12   0.000     .0233059    .0261468
          3  |   .0115267   .0003536    32.60   0.000     .0108337    .0122197
          4  |  -.0514178    .001495   -34.39   0.000    -.0543479   -.0484877
------------------------------------------------------------------------------
Note: dy/dx for factor levels is the discrete change from the base level.

. margins, dydx(schoollevel) vsquish
```

```
Average marginal effects                                 Number of obs = 415,932
Model VCE: Robust

dy/dx wrt: 20.schoollevel 30.schoollevel

1._predict: Pr(closefriend==1), predict(pr outcome(1))
2._predict: Pr(closefriend==2), predict(pr outcome(2))
3._predict: Pr(closefriend==3), predict(pr outcome(3))
4._predict: Pr(closefriend==4), predict(pr outcome(4))

------------------------------------------------------------------------------
             |            Delta-method
             |      dy/dx   std. err.      z    P>|z|     [95% conf. interval]
-------------+----------------------------------------------------------------
10.schooll~l |  (base outcome)
-------------+----------------------------------------------------------------
20.schooll~l |
    _predict |
          1  |  -.0029574   .0004943    -5.98   0.000    -.0039262   -.0019885
          2  |  -.0047925   .0008039    -5.96   0.000    -.0063681   -.0032169
          3  |  -.0022115   .0003748    -5.90   0.000    -.0029461   -.0014769
          4  |   .0099613   .0016725     5.96   0.000     .0066833    .0132394
-------------+----------------------------------------------------------------
30.schooll~l |
    _predict |
          1  |  -.0029662   .0006248    -4.75   0.000    -.0041907   -.0017418
          2  |  -.0048071   .0010186    -4.72   0.000    -.0068034   -.0028107
          3  |  -.0022184   .0004788    -4.63   0.000    -.0031569     -.00128
          4  |   .0099918   .0021217     4.71   0.000     .0058333    .0141502
------------------------------------------------------------------------------
Note: dy/dx for factor levels is the discrete change from the base level.

. margins, dydx(degree) vsquish

Average marginal effects                                 Number of obs = 415,932
Model VCE: Robust

dy/dx wrt: 1.degree

1._predict: Pr(closefriend==1), predict(pr outcome(1))
2._predict: Pr(closefriend==2), predict(pr outcome(2))
3._predict: Pr(closefriend==3), predict(pr outcome(3))
4._predict: Pr(closefriend==4), predict(pr outcome(4))

------------------------------------------------------------------------------
             |            Delta-method
             |      dy/dx   std. err.      z    P>|z|     [95% conf. interval]
-------------+----------------------------------------------------------------
0.degree     |  (base outcome)
-------------+----------------------------------------------------------------
1.degree     |
    _predict |
          1  |  -.0101771   .0004532   -22.46   0.000    -.0110653   -.0092889
          2  |  -.0164425   .0007298   -22.53   0.000    -.0178727   -.0150122
          3  |  -.0075019    .000332   -22.60   0.000    -.0081526   -.0068512
          4  |   .0341214   .0015084    22.62   0.000     .0311651    .0370778
------------------------------------------------------------------------------
Note: dy/dx for factor levels is the discrete change from the base level.

. margins, dydx(urbanrural) vsquish

Average marginal effects                                 Number of obs = 415,932
Model VCE: Robust

dy/dx wrt: 2.urbanrural 3.urbanrural

1._predict: Pr(closefriend==1), predict(pr outcome(1))
2._predict: Pr(closefriend==2), predict(pr outcome(2))
3._predict: Pr(closefriend==3), predict(pr outcome(3))
4._predict: Pr(closefriend==4), predict(pr outcome(4))

------------------------------------------------------------------------------
             |            Delta-method
             |      dy/dx   std. err.      z    P>|z|     [95% conf. interval]
-------------+----------------------------------------------------------------
1.urbanrural |  (base outcome)
-------------+----------------------------------------------------------------
2.urbanrural |
    _predict |
          1  |   .0023732   .0005895     4.03   0.000     .0012178    .0035286
          2  |   .0038424   .0009489     4.05   0.000     .0019827    .0057021
          3  |   .0017683   .0004299     4.11   0.000     .0009257     .002611
          4  |   -.007984    .001968    -4.06   0.000    -.0118412   -.0041268
-------------+----------------------------------------------------------------
3.urbanrural |
    _predict |
          1  |   .0067052   .0007148     9.38   0.000     .0053043    .0081061
          2  |   .0106689   .0011155     9.56   0.000     .0084825    .0128553
          3  |   .0046834   .0004643    10.09   0.000     .0037733    .0055934
          4  |  -.0220575   .0022926    -9.62   0.000    -.0265509    -.017564
------------------------------------------------------------------------------
Note: dy/dx for factor levels is the discrete change from the base level.

. margins, dydx(immigrant) vsquish

Average marginal effects                                 Number of obs = 415,932
Model VCE: Robust

dy/dx wrt: 2.immigrant 3.immigrant 4.immigrant

1._predict: Pr(closefriend==1), predict(pr outcome(1))
2._predict: Pr(closefriend==2), predict(pr outcome(2))
3._predict: Pr(closefriend==3), predict(pr outcome(3))
4._predict: Pr(closefriend==4), predict(pr outcome(4))

------------------------------------------------------------------------------
             |            Delta-method
             |      dy/dx   std. err.      z    P>|z|     [95% conf. interval]
-------------+----------------------------------------------------------------
1.immigrant  |  (base outcome)
-------------+----------------------------------------------------------------
```

```
2.immigrant  |
    _predict |
           1  |   .0110931   .0009072    12.23   0.000      .009315    .0128712
           2  |   .0175911   .0013822    12.73   0.000      .014882    .0203002
           3  |   .0075525   .0005308    14.23   0.000     .0065123    .0085928
           4  |  -.0362368   .0028159   -12.87   0.000    -.0417559   -.0307176
-------------+----------------------------------------------------------------
3.immigrant  |
    _predict |
           1  |    .019154   .0018542    10.33   0.000     .0155198    .0227881
           2  |   .0294169   .0026369    11.16   0.000     .0242486    .0345853
           3  |   .0115483   .0008077    14.30   0.000     .0099652    .0131313
           4  |  -.0601192   .0052917   -11.36   0.000    -.0704908   -.0497476
-------------+----------------------------------------------------------------
4.immigrant  |
    _predict |
           1  |   .0455418   .0019456    23.41   0.000     .0417285     .049355
           2  |   .0630857   .0022296    28.29   0.000     .0587158    .0674557
           3  |   .0180966    .000271    66.78   0.000     .0175655    .0186277
           4  |  -.1267241   .0043727   -28.98   0.000    -.1352944   -.1181538
------------------------------------------------------------------------------
Note: dy/dx for factor levels is the discrete change from the base level.


*Interactions year*gender*school level

. ologit closefriend i.year##i.gender i.year##i.schoollevel i.degree i.urbanrural i.immigrant, vce(robust) nolog or

Ordered logistic regression                          Number of obs = 415,932
                                                     Wald chi2(17) = 4427.81
                                                     Prob > chi2   =  0.0000
Log pseudolikelihood = -486632.63                    Pseudo R2     =  0.0045

----------------------------------------------------------------------------------------------------
                                  |               Robust
                      closefriend | Odds ratio   std. err.      z    P>|z|     [95% conf. interval]
----------------------------------+-----------------------------------------------------------------
                             year |
                             2019  |   .9720055   .0133768    -2.06   0.039     .9461378    .9985803
                             2021  |    .901538   .0121112    -7.72   0.000     .8781103    .9255907
                                  |
                           gender |
                             girl  |   .8717079   .0097955   -12.22   0.000      .852719    .8911198
                                  |
                      year#gender |
                         2019#girl  |   .9911431   .0151972    -0.58   0.562     .9618003    1.021381
                         2021#girl  |   .8339654   .0124507   -12.16   0.000     .8099159    .8587289
                                  |
                      schoollevel |
                   uppersecondary  |   1.063769   .0138151     4.76   0.000     1.037033    1.091193
                       vocational  |   1.100368   .0162102     6.49   0.000      1.06905    1.132602
                                  |
                 year#schoollevel |
              2019#uppersecondary  |   .9590125    .016649    -2.41   0.016     .9269299    .9922055
                  2019#vocational  |   .9258753   .0193285    -3.69   0.000     .8887567    .9645441
              2021#uppersecondary  |   .9828072   .0165017    -1.03   0.302     .9509908    1.015688
                  2021#vocational  |   .9190064   .0189204    -4.10   0.000     .8826612    .9568481
                                  |
                         1.degree |   1.148281   .0070125    22.64   0.000     1.134619    1.162108
                                  |
                       urbanrural |
                        semiurban  |   .9686666   .0077177    -4.00   0.000     .9536576    .9839117
                            rural  |   .9151797   .0084793    -9.57   0.000     .8987106    .9319505
                                  |
                        immigrant |
          one parent foreign-born  |   .8645557   .0098099   -12.83   0.000     .8455409    .8839982
born in Finland parents foreign-born  |   .7843242   .0167452   -11.38   0.000     .7521815    .8178406
     student and parents born abroad  |   .5981597   .0108564   -28.31   0.000     .5772555    .6198209
----------------------------------+-----------------------------------------------------------------
                            /cut1 |  -2.567881   .0122264                         -2.591845   -2.543918
                            /cut2 |  -1.151461   .0112175                         -1.173447   -1.129475
                            /cut3 |  -.2356877   .0109943                         -.2572361   -.2141392
----------------------------------------------------------------------------------------------------
Note: Estimates are transformed only in the first equation to odds ratios.

. margins, dydx(year) at(gender=(1 2 )) vsquish

Average marginal effects                             Number of obs = 415,932
Model VCE: Robust

dy/dx wrt: 2019.year 2021.year

1._predict: Pr(closefriend==1), predict(pr outcome(1))
2._predict: Pr(closefriend==2), predict(pr outcome(2))
3._predict: Pr(closefriend==3), predict(pr outcome(3))
4._predict: Pr(closefriend==4), predict(pr outcome(4))
1._at: gender = 1
2._at: gender = 2

------------------------------------------------------------------------------
             |            Delta-method
             |      dy/dx   std. err.      z    P>|z|     [95% conf. interval]
-------------+----------------------------------------------------------------
2017.year    |  (base outcome)
-------------+----------------------------------------------------------------
2019.year    |
_predict#_at |
         1 1  |   .0033712   .0007675     4.39   0.000     .0018669    .0048756
         1 2  |   .0044513   .0007013     6.35   0.000     .0030767    .0058258
         2 1  |   .0060249   .0013615     4.43   0.000     .0033564    .0086935
         2 2  |   .0073544   .0011502     6.39   0.000     .0051002    .0096087
         3 1  |   .0035102   .0007813     4.49   0.000     .0019789    .0050415
         3 2  |    .003501   .0005391     6.49   0.000     .0024444    .0045577
         4 1  |  -.0129063   .0029092    -4.44   0.000    -.0186082   -.0072045
         4 2  |  -.0153067   .0023887    -6.41   0.000    -.0199886   -.0106249
-------------+----------------------------------------------------------------
2021.year    |
_predict#_at |
         1 1  |   .0080946    .000775    10.44   0.000     .0065756    .0096135
         1 2  |   .0244565   .0007451    32.82   0.000     .0229962    .0259168
         2 1  |   .0141142   .0013501    10.45   0.000      .011468    .0167604
         2 2  |    .037083   .0011307    32.80   0.000     .0348669     .039299
```

```
        3 1  |    .007746   .0007458    10.39   0.000     .0062843    .0092076
        3 2  |   .0139112   .0004566    30.47   0.000     .0130163    .0148061
        4 1  |  -.0299548   .0028659   -10.45   0.000    -.0355718   -.0243377
        4 2  |  -.0754506   .0022967   -32.85   0.000    -.0799521   -.0709491
------------------------------------------------------------------------------
Note: dy/dx for factor levels is the discrete change from the base level.

. margins, dydx(year) at(schoollevel=(10 20 30)) vsquish

Average marginal effects                                 Number of obs = 415,932
Model VCE: Robust

dy/dx wrt: 2019.year 2021.year

1._predict: Pr(closefriend==1), predict(pr outcome(1))
2._predict: Pr(closefriend==2), predict(pr outcome(2))
3._predict: Pr(closefriend==3), predict(pr outcome(3))
4._predict: Pr(closefriend==4), predict(pr outcome(4))
1._at: schoollevel = 10
2._at: schoollevel = 20
3._at: schoollevel = 30

------------------------------------------------------------------------------
             |            Delta-method
             |      dy/dx   std. err.      z    P>|z|     [95% conf. interval]
-------------+----------------------------------------------------------------
2017.year    |  (base outcome)
-------------+----------------------------------------------------------------
2019.year    |
_predict#_at |
        1 1  |   .0023391   .0007151     3.27   0.001     .0009376    .0037406
        1 2  |   .0050945   .0009514     5.35   0.000     .0032297    .0069592
        1 3  |   .0073748   .0012175     6.06   0.000     .0049886     .009761
        2 1  |   .0039101    .001205     3.24   0.001     .0015484    .0062719
        2 2  |   .0087355   .0016415     5.32   0.000     .0055182    .0119528
        2 3  |   .0127626   .0021006     6.08   0.000     .0086455    .0168798
        3 1  |    .001928   .0006102     3.16   0.002     .0007321     .003124
        3 2  |   .0046051   .0008836     5.21   0.000     .0028733    .0063369
        3 3  |   .0068916   .0011346     6.07   0.000     .0046678    .0091154
        4 1  |  -.0081772   .0025279    -3.23   0.001    -.0131319   -.0032226
        4 2  |  -.0184351   .0034735    -5.31   0.000    -.0252429   -.0116272
        4 3  |   -.027029   .0044473    -6.08   0.000    -.0357456   -.0183125
-------------+----------------------------------------------------------------
2021.year    |
_predict#_at |
        1 1  |   .0157053   .0007384    21.27   0.000     .0142581    .0171526
        1 2  |   .0162843   .0009729    16.74   0.000     .0143775    .0181912
        1 3  |   .0211419   .0013229    15.98   0.000     .0185491    .0237346
        2 1  |     .02421   .0011824    20.48   0.000     .0218925    .0265275
        2 2  |   .0259946   .0016106    16.14   0.000     .0228379    .0291512
        2 3  |   .0339009   .0021236    15.96   0.000     .0297387    .0380631
        3 1  |   .0096126   .0005472    17.57   0.000     .0085401    .0106851
        3 2  |   .0114314   .0008094    14.12   0.000      .009845    .0130179
        3 3  |   .0151703   .0010142    14.96   0.000     .0131825    .0171581
        4 1  |  -.0495279    .002448   -20.23   0.000    -.0543259     -.04473
        4 2  |  -.0537103   .0033725   -15.93   0.000    -.0603203   -.0471004
        4 3  |  -.0702131   .0044209   -15.88   0.000    -.0788779   -.0615483
------------------------------------------------------------------------------
Note: dy/dx for factor levels is the discrete change from the base level.

. margins, dydx(degree)

Average marginal effects                                 Number of obs = 415,932
Model VCE: Robust

dy/dx wrt: 1.degree

1._predict: Pr(closefriend==1), predict(pr outcome(1))
2._predict: Pr(closefriend==2), predict(pr outcome(2))
3._predict: Pr(closefriend==3), predict(pr outcome(3))
4._predict: Pr(closefriend==4), predict(pr outcome(4))

------------------------------------------------------------------------------
             |            Delta-method
             |      dy/dx   std. err.      z    P>|z|     [95% conf. interval]
-------------+----------------------------------------------------------------
0.degree     |  (base outcome)
-------------+----------------------------------------------------------------
1.degree     |
    _predict |
          1  |  -.0101874   .0004531   -22.48   0.000    -.0110754   -.0092994
          2  |  -.0164531   .0007293   -22.56   0.000    -.0178825   -.0150237
          3  |   -.007515   .0003322   -22.62   0.000     -.008166    -.006864
          4  |   .0341555    .001508    22.65   0.000        .0312    .0371111
------------------------------------------------------------------------------
Note: dy/dx for factor levels is the discrete change from the base level.

. margins, dydx(urbanrural)

Average marginal effects                                 Number of obs = 415,932
Model VCE: Robust

dy/dx wrt: 2.urbanrural 3.urbanrural

1._predict: Pr(closefriend==1), predict(pr outcome(1))
2._predict: Pr(closefriend==2), predict(pr outcome(2))
3._predict: Pr(closefriend==3), predict(pr outcome(3))
4._predict: Pr(closefriend==4), predict(pr outcome(4))

-------------------------------------------------------------------------------
              |            Delta-method
              |      dy/dx   std. err.      z    P>|z|     [95% conf. interval]
--------------+----------------------------------------------------------------
1.urbanrural  |  (base outcome)
--------------+----------------------------------------------------------------
2.urbanrural  |
     _predict |
           1  |   .0023353   .0005892     3.96   0.000     .0011805    .0034901
           2  |   .0037798   .0009482     3.99   0.000     .0019214    .0056383
           3  |   .0017417   .0004303     4.05   0.000     .0008984    .0025851
           4  |  -.0078568   .0019674    -3.99   0.000    -.0117129   -.0040007
--------------+----------------------------------------------------------------
```

```
3.urbanrural  |
     _predict |
           1  |   .0066585   .0007146     9.32   0.000     .0052579    .0080591
           2  |   .0105918   .0011151     9.50   0.000     .0084062    .0127774
           3  |   .0046562    .000465    10.01   0.000     .0037447    .0055676
           4  |  -.0219065   .0022928    -9.55   0.000    -.0264003   -.0174128
------------------------------------------------------------------------------
Note: dy/dx for factor levels is the discrete change from the base level.

. margins, dydx(immigrant)

Average marginal effects                              Number of obs = 415,932
Model VCE: Robust

dy/dx wrt: 2.immigrant 3.immigrant 4.immigrant

1._predict: Pr(closefriend==1), predict(pr outcome(1))
2._predict: Pr(closefriend==2), predict(pr outcome(2))
3._predict: Pr(closefriend==3), predict(pr outcome(3))
4._predict: Pr(closefriend==4), predict(pr outcome(4))

-----------------------------------------------------------------------------
             |            Delta-method
             |      dy/dx   std. err.      z    P>|z|     [95% conf. interval]
-------------+---------------------------------------------------------------
1.immigrant  |  (base outcome)
-------------+---------------------------------------------------------------
2.immigrant  |
    _predict |
          1  |   .0110361   .0009068    12.17   0.000     .0092588    .0128134
          2  |   .0174977   .0013817    12.66   0.000     .0147897    .0202057
          3  |   .0075256   .0005319    14.15   0.000     .0064831    .0085682
          4  |  -.0360594   .0028161   -12.80   0.000    -.0415789   -.0305399
-------------+---------------------------------------------------------------
3.immigrant  |
    _predict |
          1  |   .0191935   .0018548    10.35   0.000     .0155582    .0228287
          2  |   .0294614    .002636    11.18   0.000     .0242951    .0346278
          3  |   .0115755   .0008079    14.33   0.000      .009992    .0131591
          4  |  -.0602304   .0052915   -11.38   0.000    -.0706016   -.0498592
-------------+---------------------------------------------------------------
4.immigrant  |
    _predict |
          1  |   .0454929   .0019446    23.39   0.000     .0416816    .0493042
          2  |   .0630078   .0022288    28.27   0.000     .0586395    .0673762
          3  |   .0181196   .0002725    66.49   0.000     .0175855    .0186537
          4  |  -.1266204   .0043729   -28.96   0.000    -.1351911   -.1180496
-----------------------------------------------------------------------------
Note: dy/dx for factor levels is the discrete change from the base level.


*Interactions year*gender*school level with parental education, urban-rural location of the school and immigration status of the student

. ologit closefriend i.year##i.schoollevel##i.degree i.year##i.gender##i.degree i.year##i.urbanrural i.immigrant, vce(robust) nolog or

Ordered logistic regression                           Number of obs = 415,932
                                                      Wald chi2(32) = 4641.80
                                                      Prob > chi2   =  0.0000
Log pseudolikelihood = -486559.68                     Pseudo R2     =  0.0046

-----------------------------------------------------------------------------------------------------
                                     |               Robust
                         closefriend | Odds ratio   std. err.      z    P>|z|     [95% conf. interval]
-------------------------------------+---------------------------------------------------------------
                                year |
                                2019 |   .9924609   .0208547    -0.36   0.719     .9524167    1.034189
                                2021 |   .9081297   .0187186    -4.68   0.000     .8721731    .9455687
                                     |
                         schoollevel |
                     uppersecondary  |   1.028791   .0203821     1.43   0.152     .9896086    1.069525
                         vocational  |   1.068332   .0197563     3.57   0.000     1.030303    1.107764
                                     |
                    year#schoollevel |
                2019#uppersecondary  |   .9700578   .0264168    -1.12   0.264     .9196394     1.02324
                    2019#vocational  |   .9582618   .0255008    -1.60   0.109     .9095621    1.009569
                2021#uppersecondary  |   .9556722   .0255197    -1.70   0.090     .9069409    1.007022
                    2021#vocational  |   .9382204   .0247936    -2.41   0.016     .8908628    .9880955
                                     |
                            1.degree |   1.086576   .0221284     4.08   0.000     1.044059    1.130824
                                     |
                         year#degree |
                              2019 1 |   .9616864   .0266555    -1.41   0.159     .9108364    1.015375
                              2021 1 |   .9650511   .0261283    -1.31   0.189     .9151755    1.017645
                                     |
                  schoollevel#degree |
                   uppersecondary#1  |      1.055   .0278282     2.03   0.042     1.001843    1.110977
                       vocational#1  |   1.076474   .0336124     2.36   0.018      1.01257     1.14441
                                     |
             year#schoollevel#degree |
             2019#uppersecondary#1   |   .9823991   .0349679    -0.50   0.618     .9161992    1.053382
                 2019#vocational#1   |   .9056981   .0399387    -2.25   0.025     .8307071    .9874588
             2021#uppersecondary#1   |   1.049262   .0363278     1.39   0.165     .9804226    1.122934
                 2021#vocational#1   |   .9375032   .0405501    -1.49   0.136     .8613021    1.020446
                                     |
                              gender |
                               girl  |   .8374055   .0130688   -11.37   0.000     .8121788    .8634157
                                     |
                         year#gender |
                          2019#girl  |   .9491619   .0208424    -2.38   0.017     .9091781    .9909042
                          2021#girl  |   .8284498    .017917    -8.70   0.000      .794067    .8643215
                                     |
                       gender#degree |
                             girl#1  |   1.089069   .0245131     3.79   0.000     1.042068    1.138189
                                     |
                  year#gender#degree |
                        2019#girl#1  |   1.070761   .0329505     2.22   0.026     1.008088     1.13733
                        2021#girl#1  |   .9986191   .0299886    -0.05   0.963     .9415388     1.05916
                                     |
                          urbanrural |
                          semiurban  |   .9571703   .0141106    -2.97   0.003     .9299097    .9852299
                              rural  |     .88462   .0148872    -7.28   0.000     .8559176    .9142848
                                     |
```

```
             year#urbanrural |
              2019#semiurban  |   .9985336   .0201384    -0.07   0.942     .9598329    1.038795
                  2019#rural  |   1.026009   .0238969     1.10   0.270     .9802246    1.073931
              2021#semiurban  |   1.032968   .0202358     1.66   0.098     .9940586    1.073401
                  2021#rural  |   1.070911   .0242837     3.02   0.003     1.024358     1.11958
                              |
                    immigrant |
        one parent foreign-born  |   .8649865    .009816   -12.78   0.000     .8459599    .8844412
born in Finland parents foreign-born  |   .7856848   .0167855   -11.29   0.000     .7534652    .8192822
     student and parents born abroad  |    .598117   .0108614   -28.30   0.000     .5772035    .6197883
-------------------------------------+----------------------------------------------------------------
                          /cut1 |  -2.602482   .0161065                     -2.63405   -2.570914
                          /cut2 |  -1.185894   .0153389                    -1.215958   -1.155831
                          /cut3 |  -.2698157    .015169                    -.2995464    -.240085
------------------------------------------------------------------------------------------------------
Note: Estimates are transformed only in the first equation to odds ratios.

. margins schoollevel, dydx(year) at(degree=(0 1)) vsquish

Average marginal effects                             Number of obs = 415,932
Model VCE: Robust

dy/dx wrt: 2019.year 2021.year

1._predict: Pr(closefriend==1), predict(pr outcome(1))
2._predict: Pr(closefriend==2), predict(pr outcome(2))
3._predict: Pr(closefriend==3), predict(pr outcome(3))
4._predict: Pr(closefriend==4), predict(pr outcome(4))
1._at: degree = 0
2._at: degree = 1

------------------------------------------------------------------------------------------
                         |            Delta-method
                         |      dy/dx   std. err.      z    P>|z|     [95% conf. interval]
-------------------------+----------------------------------------------------------------
2017.year                |  (base outcome)
-------------------------+----------------------------------------------------------------
2019.year                |
_predict#_at#schoollevel |
     1#1#lowersecondary  |    .002546   .0010753     2.37   0.018     .0004385    .0046534
     1#1#uppersecondary  |   .0047497    .001704     2.79   0.005     .0014099    .0080894
         1#1#vocational  |   .0055007   .0015821     3.48   0.001     .0023999    .0086015
     1#2#lowersecondary  |   .0023122   .0009675     2.39   0.017     .0004159    .0042084
     1#2#uppersecondary  |    .005248   .0011223     4.68   0.000     .0030483    .0074477
         1#2#vocational  |   .0110677   .0020261     5.46   0.000     .0070966    .0150388
     2#1#lowersecondary  |   .0039487   .0017472     2.26   0.024     .0005242    .0073732
     2#1#uppersecondary  |   .0075715   .0027891     2.71   0.007     .0021049     .013038
         2#1#vocational  |   .0089663   .0026133     3.43   0.001     .0038444    .0140883
     2#2#lowersecondary  |   .0040681   .0016908     2.41   0.016     .0007542     .007382
     2#2#uppersecondary  |   .0094645    .002023     4.68   0.000     .0054995    .0134294
         2#2#vocational  |    .020014   .0036366     5.50   0.000     .0128864    .0271415
     3#1#lowersecondary  |   .0015613   .0008073     1.93   0.053    -.0000209    .0031436
     3#1#uppersecondary  |   .0032653    .001308     2.50   0.013     .0007017     .005829
         3#1#vocational  |   .0041202   .0012585     3.27   0.001     .0016536    .0065867
     3#2#lowersecondary  |   .0022711   .0009302     2.44   0.015      .000448    .0040942
     3#2#uppersecondary  |   .0056198   .0012042     4.67   0.000     .0032597      .00798
         3#2#vocational  |   .0119805   .0021649     5.53   0.000     .0077373    .0162236
     4#1#lowersecondary  |   -.008056   .0036222    -2.22   0.026    -.0151554   -.0009566
     4#1#uppersecondary  |  -.0155865   .0057948    -2.69   0.007     -.026944   -.0042289
         4#1#vocational  |  -.0185872   .0054466    -3.41   0.001    -.0292624   -.0079119
     4#2#lowersecondary  |  -.0086513    .003587    -2.41   0.016    -.0156817    -.001621
     4#2#uppersecondary  |  -.0203323   .0043465    -4.68   0.000    -.0288514   -.0118132
         4#2#vocational  |  -.0430621   .0078126    -5.51   0.000    -.0583744   -.0277497
-------------------------+----------------------------------------------------------------
2021.year                |
_predict#_at#schoollevel |
     1#1#lowersecondary  |   .0152121   .0011123    13.68   0.000     .0130321    .0173922
     1#1#uppersecondary  |   .0187237   .0017964    10.42   0.000     .0152027    .0222446
         1#1#vocational  |   .0197167   .0017324    11.38   0.000     .0163212    .0231122
     1#2#lowersecondary  |   .0162527   .0009946    16.34   0.000     .0143034    .0182021
     1#2#uppersecondary  |   .0149685   .0011312    13.23   0.000     .0127514    .0171856
         1#2#vocational  |   .0239765   .0021505    11.15   0.000     .0197616    .0281913
     2#1#lowersecondary  |   .0223755   .0017155    13.04   0.000     .0190133    .0257378
     2#1#uppersecondary  |   .0278346   .0027748    10.03   0.000     .0223962    .0332731
         2#1#vocational  |   .0299224   .0026561    11.27   0.000     .0247165    .0351283
     2#2#lowersecondary  |   .0261914    .001654    15.84   0.000     .0229497    .0294332
     2#2#uppersecondary  |   .0253099    .001972    12.83   0.000     .0214448    .0291749
         2#2#vocational  |    .040523   .0036152    11.21   0.000     .0334373    .0476086
     3#1#lowersecondary  |    .007627   .0007223    10.56   0.000     .0062113    .0090428
     3#1#uppersecondary  |   .0098768   .0011717     8.43   0.000     .0075804    .0121733
         3#1#vocational  |   .0113485   .0011132    10.19   0.000     .0091666    .0135304
     3#2#lowersecondary  |   .0117117    .000835    14.03   0.000      .010075    .0133483
     3#2#uppersecondary  |   .0128803   .0011131    11.57   0.000     .0106986     .015062
         3#2#vocational  |   .0207512   .0019514    10.63   0.000     .0169265    .0245759
     4#1#lowersecondary  |  -.0452147   .0035191   -12.85   0.000    -.0521121   -.0383173
     4#1#uppersecondary  |  -.0564351   .0057023    -9.90   0.000    -.0676113   -.0452589
         4#1#vocational  |  -.0609876   .0054546   -11.18   0.000    -.0716783   -.0502968
     4#2#lowersecondary  |  -.0541558   .0034616   -15.64   0.000    -.0609404   -.0473713
     4#2#uppersecondary  |  -.0531587   .0041973   -12.66   0.000    -.0613852   -.0449321
         4#2#vocational  |  -.0852507   .0076459   -11.15   0.000    -.1002363    -.070265
------------------------------------------------------------------------------------------
Note: dy/dx for factor levels is the discrete change from the base level.

. margins gender, dydx(year) at(degree=(0 1)) vsquish

Average marginal effects                             Number of obs = 415,932
Model VCE: Robust

dy/dx wrt: 2019.year 2021.year

1._predict: Pr(closefriend==1), predict(pr outcome(1))
2._predict: Pr(closefriend==2), predict(pr outcome(2))
3._predict: Pr(closefriend==3), predict(pr outcome(3))
4._predict: Pr(closefriend==4), predict(pr outcome(4))
1._at: degree = 0
2._at: degree = 1

----------------------------------------------------------------------------------
                 |            Delta-method
                 |      dy/dx   std. err.      z    P>|z|     [95% conf. interval]
-----------------+----------------------------------------------------------------
2017.year        |  (base outcome)
```

```
-----------------+----------------------------------------------------------------
2019.year        |
_predict#_at#gender |
        1#1#boy  |   .0013331   .0011856     1.12   0.261    -.0009906    .0036569
       1#1#girl  |    .005727   .0010813     5.30   0.000     .0036077    .0078462
        1#2#boy  |   .0049199    .001028     4.79   0.000      .002905    .0069348
       1#2#girl  |   .0042012   .0009465     4.44   0.000     .0023462    .0060563
        2#1#boy  |   .0023562   .0020605     1.14   0.253    -.0016824    .0063947
       2#1#girl  |   .0088774    .001667     5.33   0.000     .0056101    .0121447
        2#2#boy  |   .0090138   .0018607     4.84   0.000     .0053669    .0126607
       2#2#girl  |   .0073999   .0016413     4.51   0.000     .0041831    .0106167
        3#1#boy  |   .0013272   .0011146     1.19   0.234    -.0008573    .0035118
       3#1#girl  |   .0034959   .0006499     5.38   0.000     .0022221    .0047697
        3#2#boy  |   .0055604   .0011197     4.97   0.000     .0033657     .007755
       3#2#girl  |   .0041418   .0008875     4.67   0.000     .0024022    .0058813
        4#1#boy  |  -.0050165   .0043602    -1.15   0.250    -.0135623    .0035293
       4#1#girl  |  -.0181003   .0033951    -5.33   0.000    -.0247545   -.0114461
        4#2#boy  |   -.019494   .0040056    -4.87   0.000    -.0273448   -.0116432
       4#2#girl  |  -.0157429   .0034725    -4.53   0.000    -.0225489   -.0089369
-----------------+----------------------------------------------------------------
2021.year        |
_predict#_at#gender |
        1#1#boy  |   .0073084   .0012205     5.99   0.000     .0049162    .0097006
       1#1#girl  |   .0255507   .0011518    22.18   0.000     .0232932    .0278082
        1#2#boy  |   .0087351   .0010236     8.53   0.000     .0067288    .0107414
       1#2#girl  |   .0245228   .0010013    24.49   0.000     .0225604    .0264853
        2#1#boy  |   .0124641   .0020693     6.02   0.000     .0084084    .0165197
       2#1#girl  |   .0364848   .0016341    22.33   0.000     .0332819    .0396876
        2#2#boy  |   .0156594   .0018281     8.57   0.000     .0120764    .0192424
       2#2#girl  |   .0393223   .0016078    24.46   0.000     .0361712    .0424734
        3#1#boy  |   .0064219   .0010563     6.08   0.000     .0043516    .0084923
       3#1#girl  |   .0110724   .0005374    20.60   0.000     .0100191    .0121256
        3#2#boy  |   .0091776   .0010714     8.57   0.000     .0070777    .0112775
       3#2#girl  |   .0173214    .000762    22.73   0.000     .0158278     .018815
        4#1#boy  |  -.0261944   .0043413    -6.03   0.000    -.0347033   -.0176855
       4#1#girl  |  -.0731079    .003275   -22.32   0.000    -.0795268   -.0666889
        4#2#boy  |  -.0335721   .0039162    -8.57   0.000    -.0412476   -.0258965
       4#2#girl  |  -.0811665    .003329   -24.38   0.000    -.0876912   -.0746419
----------------------------------------------------------------------------------
Note: dy/dx for factor levels is the discrete change from the base level.

. margins, dydx(year) at(urbanrural=(1 2 3)) vsquish

Average marginal effects                                Number of obs = 415,932
Model VCE: Robust

dy/dx wrt: 2019.year 2021.year

1._predict: Pr(closefriend==1), predict(pr outcome(1))
2._predict: Pr(closefriend==2), predict(pr outcome(2))
3._predict: Pr(closefriend==3), predict(pr outcome(3))
4._predict: Pr(closefriend==4), predict(pr outcome(4))
1._at: urbanrural = 1
2._at: urbanrural = 2
3._at: urbanrural = 3

------------------------------------------------------------------------------
             |            Delta-method
             |      dy/dx   std. err.      z    P>|z|     [95% conf. interval]
-------------+----------------------------------------------------------------
2017.year    |  (base outcome)
-------------+----------------------------------------------------------------
2019.year    |
_predict#_at |
        1 1  |   .0041319   .0006129     6.74   0.000     .0029307    .0053332
        1 2  |    .004394   .0012595     3.49   0.000     .0019254    .0068625
        1 3  |   .0026279   .0015797     1.66   0.096    -.0004683    .0057242
        2 1  |   .0071233   .0010546     6.75   0.000     .0050564    .0091902
        2 2  |   .0073908   .0021185     3.49   0.000     .0032386    .0115429
        2 3  |   .0042663   .0025597     1.67   0.096    -.0007506    .0092831
        3 1  |   .0038238   .0005694     6.72   0.000     .0027078    .0049399
        3 2  |   .0037238   .0010697     3.48   0.000     .0016272    .0058204
        3 3  |   .0019599    .001166     1.68   0.093    -.0003254    .0042452
        4 1  |   -.015079   .0022309    -6.76   0.000    -.0194515   -.0107066
        4 2  |  -.0155085   .0044434    -3.49   0.000    -.0242174   -.0067996
        4 3  |  -.0088541   .0053021    -1.67   0.095     -.019246    .0015378
-------------+----------------------------------------------------------------
2021.year    |
_predict#_at |
        1 1  |   .0175599   .0006375    27.54   0.000     .0163103    .0188094
        1 2  |    .015538   .0012974    11.98   0.000     .0129952    .0180809
        1 3  |    .013497   .0016264     8.30   0.000     .0103093    .0166848
        2 1  |   .0279072   .0010418    26.79   0.000     .0258653     .029949
        2 2  |   .0242079   .0020823    11.63   0.000     .0201266    .0282892
        2 3  |    .020115   .0025158     8.00   0.000     .0151841    .0250459
        3 1  |   .0121679   .0005174    23.52   0.000     .0111538     .013182
        3 2  |   .0099495   .0009674    10.28   0.000     .0080535    .0118455
        3 3  |   .0072009    .001044     6.90   0.000     .0051547    .0092472
        4 1  |  -.0576349   .0021698   -26.56   0.000    -.0618876   -.0533822
        4 2  |  -.0496954   .0043234   -11.49   0.000    -.0581691   -.0412217
        4 3  |   -.040813   .0051646    -7.90   0.000    -.0509354   -.0306905
------------------------------------------------------------------------------
Note: dy/dx for factor levels is the discrete change from the base level.

. margins, dydx(immigrant) vsquish

Average marginal effects                                Number of obs = 415,932
Model VCE: Robust

dy/dx wrt: 2.immigrant 3.immigrant 4.immigrant

1._predict: Pr(closefriend==1), predict(pr outcome(1))
2._predict: Pr(closefriend==2), predict(pr outcome(2))
3._predict: Pr(closefriend==3), predict(pr outcome(3))
4._predict: Pr(closefriend==4), predict(pr outcome(4))

------------------------------------------------------------------------------
             |            Delta-method
             |      dy/dx   std. err.      z    P>|z|     [95% conf. interval]
-------------+----------------------------------------------------------------
1.immigrant  |  (base outcome)
-------------+----------------------------------------------------------------
```

```
2.immigrant  |
    _predict |
          1  |   .0109945   .0009064    12.13   0.000      .009218    .0127711
          2  |   .0174297   .0013811    12.62   0.000     .0147227    .0201367
          3  |   .0075021   .0005324    14.09   0.000     .0064586    .0085456
          4  |  -.0359264   .0028158   -12.76   0.000    -.0414451   -.0304076
-------------+----------------------------------------------------------------
3.immigrant  |
    _predict |
          1  |   .0190399   .0018531    10.27   0.000     .0154078     .022672
          2  |    .029235   .0026361    11.09   0.000     .0240683    .0344018
          3  |   .0115106   .0008119    14.18   0.000     .0099193    .0131018
          4  |  -.0597855   .0052942   -11.29   0.000    -.0701619   -.0494091
-------------+----------------------------------------------------------------
4.immigrant  |
    _predict |
          1  |   .0454928   .0019453    23.39   0.000     .0416801    .0493055
          2  |   .0629894    .002229    28.26   0.000     .0586207    .0673581
          3  |   .0181253   .0002729    66.41   0.000     .0175904    .0186603
          4  |  -.1266075   .0043743   -28.94   0.000     -.135181    -.118034
------------------------------------------------------------------------------
Note: dy/dx for factor levels is the discrete change from the base level.
```

4.2. Loneliness with exponentiated Beta and marginal effects

```
*Main effects only

. glm loneliness i.year i.gender i.schoollevel i.degree  i.immigrant i.urbanrural, family(gamma) link (log) vce(robust) eform nolog

Generalized linear models                         Number of obs   =    416,572
Optimization     : ML                             Residual df     =    416,560
                                                  Scale parameter =   .1957444
Deviance         =  84570.54471                   (1/df) Deviance =   .2030213
Pearson          =  81539.28191                   (1/df) Pearson  =   .1957444

Variance function: V(u) = u^2                     [Gamma]
Link function    : g(u) = ln(u)                   [Log]

                                                  AIC             =    3.63757
Log pseudolikelihood = -757642.8251               BIC             =   -5305639

----------------------------------------------------------------------------------------------------
                                   |               Robust
                        loneliness |     exp(b)   std. err.      z    P>|z|     [95% conf. interval]
-----------------------------------+----------------------------------------------------------------
                              year |
                              2019 |   1.024199    .001802    13.59   0.000     1.020673    1.027737
                              2021 |   1.137042     .00195    74.89   0.000     1.133227    1.140871
                                   |
                            gender |
                              girl |   1.306143   .0018213   191.53   0.000     1.302578    1.309718
                                   |
                       schoollevel |
                   uppersecondary  |   1.065874   .0016229    41.90   0.000     1.062698     1.06906
                        vocational |   1.026093   .0021297    12.41   0.000     1.021927    1.030275
                                   |
                          1.degree |   .9916205   .0014221    -5.87   0.000     .9888371    .9944118
                                   |
                         immigrant |
            one parent foreign-born |     1.0479   .0027904    17.57   0.000     1.042446    1.053384
born in Finland parents foreign-born |     .97039   .0054844    -5.32   0.000     .9597001    .9811989
     student and parents born abroad |   1.066403   .0051786    13.24   0.000     1.056301    1.076601
                                   |
                        urbanrural |
                         semiurban |   .9883112    .001851    -6.28   0.000     .9846899    .9919458
                             rural |   .9961087   .0021668    -1.79   0.073     .9918707    1.000365
                                   |
                             _cons |   1.826403   .0036111   304.66   0.000      1.81934    1.833495
----------------------------------------------------------------------------------------------------

. margins, dydx(year) vsquish

Average marginal effects                               Number of obs = 416,572
Model VCE: Robust

Expression: Predicted mean loneliness, predict()
dy/dx wrt:  2019.year 2021.year

------------------------------------------------------------------------------
             |            Delta-method
             |      dy/dx   std. err.      z    P>|z|     [95% conf. interval]
-------------+----------------------------------------------------------------
        year |
       2019  |   .0524541   .0038555    13.60   0.000     .0448974    .0600108
       2021  |   .2970566   .0039379    75.44   0.000     .2893384    .3047747
------------------------------------------------------------------------------
Note: dy/dx for factor levels is the discrete change from the base level.

. margins, dydx(gender) vsquish

Average marginal effects                               Number of obs = 416,572
Model VCE: Robust

Expression: Predicted mean loneliness, predict()
dy/dx wrt:  2.gender

------------------------------------------------------------------------------
             |            Delta-method
             |      dy/dx   std. err.      z    P>|z|     [95% conf. interval]
-------------+----------------------------------------------------------------
      gender |
       girl  |   .6041162   .0030913   195.43   0.000     .5980574     .610175
------------------------------------------------------------------------------
```

```
Note: dy/dx for factor levels is the discrete change from the base level.

. margins, dydx(schoollevel) vsquish

Average marginal effects                                Number of obs = 416,572
Model VCE: Robust

Expression: Predicted mean loneliness, predict()
dy/dx wrt:  20.schoollevel 30.schoollevel

--------------------------------------------------------------------------------
                |            Delta-method
                |      dy/dx   std. err.      z    P>|z|     [95% conf. interval]
----------------+---------------------------------------------------------------
    schoollevel |
uppersecondary  |    .1475635   .0035497    41.57   0.000     .1406062    .1545208
    vocational  |    .0584505   .0047448    12.32   0.000     .0491507    .0677502
--------------------------------------------------------------------------------
Note: dy/dx for factor levels is the discrete change from the base level.

. margins, dydx(degree) vsquish

Average marginal effects                                Number of obs = 416,572
Model VCE: Robust

Expression: Predicted mean loneliness, predict()
dy/dx wrt:  1.degree

-----------------------------------------------------------------------------
             |            Delta-method
             |      dy/dx   std. err.      z    P>|z|     [95% conf. interval]
-------------+---------------------------------------------------------------
    1.degree |  -.0193054   .0032917    -5.86   0.000     -.025757   -.0128537
-----------------------------------------------------------------------------
Note: dy/dx for factor levels is the discrete change from the base level.

. margins, dydx(urbanrural) vsquish

Average marginal effects                                Number of obs = 416,572
Model VCE: Robust

Expression: Predicted mean loneliness, predict()
dy/dx wrt:  2.urbanrural 3.urbanrural

-----------------------------------------------------------------------------
             |            Delta-method
             |      dy/dx   std. err.      z    P>|z|     [95% conf. interval]
-------------+---------------------------------------------------------------
  urbanrural |
  semiurban  |  -.0268723   .0042653    -6.30   0.000    -.0352322   -.0185125
      rural  |  -.0089461   .0049844    -1.79   0.073    -.0187154    .0008231
-----------------------------------------------------------------------------
Note: dy/dx for factor levels is the discrete change from the base level.

. margins, dydx(immigrant) vsquish

Average marginal effects                                Number of obs = 416,572
Model VCE: Robust

Expression: Predicted mean loneliness, predict()
dy/dx wrt:  2.immigrant 3.immigrant 4.immigrant

------------------------------------------------------------------------------------------------------
                                      |            Delta-method
                                      |      dy/dx   std. err.      z    P>|z|     [95% conf. interval]
--------------------------------------+---------------------------------------------------------------
                            immigrant |
             one parent foreign-born  |    .1093234   .0063474    17.22   0.000     .0968827    .1217641
born in Finland parents foreign-born  |   -.0675792   .0125235    -5.40   0.000    -.0921249   -.0430335
     student and parents born abroad  |    .1515514   .0118023    12.84   0.000     .1284194    .1746834
------------------------------------------------------------------------------------------------------
Note: dy/dx for factor levels is the discrete change from the base level.



*Interactions year*gender*school level

. glm loneliness i.year##i.gender##i.schoollevel i.degree  i.immigrant i.urbanrural, family(gamma) link (log) vce(robust) eform nolog

Generalized linear models                          Number of obs   =    416,572
Optimization     : ML                              Residual df     =    416,548
                                                   Scale parameter =   .1955379
Deviance         =  84402.40005                    (1/df) Deviance =   .2026235
Pearson          =  81450.92023                    (1/df) Pearson  =   .1955379

Variance function: V(u) = u^2                      [Gamma]
Link function    : g(u) = ln(u)                    [Log]

                                                   AIC             =   3.637224
Log pseudolikelihood = -757558.7528                BIC             =   -5305651

------------------------------------------------------------------------------------------------------
                                      |               Robust
                           loneliness |     exp(b)   std. err.      z    P>|z|     [95% conf. interval]
--------------------------------------+---------------------------------------------------------------
                                 year |
                                 2019 |   1.003193   .0038824     0.82   0.410     .9956124    1.010831
                                 2021 |   1.113805   .0042443    28.28   0.000     1.105517    1.122154
                                      |
                               gender |
                                 girl |   1.294396   .0047609    70.16   0.000     1.285099    1.303761
                                      |
                          year#gender |
                            2019#girl |   1.034819   .0051067     6.94   0.000     1.024858    1.044877
                            2021#girl |   1.035844   .0049843     7.32   0.000     1.026121    1.045659
                                      |
                          schoollevel |
                       uppersecondary |   1.096353   .0053766    18.76   0.000     1.085866    1.106942
                           vocational |   1.001766   .0052101     0.34   0.734     .9916066     1.01203
                                      |
                     year#schoollevel |
```

```
                2019#uppersecondary  |   1.022614   .0066431     3.44   0.001     1.009677    1.035718
                    2019#vocational  |   1.004672   .0074383     0.63   0.529     .9901989    1.019357
                2021#uppersecondary  |   1.015644   .0064677     2.44   0.015     1.003046    1.028399
                    2021#vocational  |   1.026928   .0075477     3.62   0.000     1.012241    1.041828
                                     |
                  gender#schoollevel |
                girl#uppersecondary  |   .9478999    .005772    -8.79   0.000     .9366542    .9592805
                    girl#vocational  |   1.045984   .0073054     6.44   0.000     1.031763    1.060401
                                     |
             year#gender#schoollevel |
           2019#girl#uppersecondary  |   .9748703   .0078631    -3.16   0.002     .9595802     .990404
               2019#girl#vocational  |   .9957354   .0099065    -0.43   0.668     .9765071    1.015342
           2021#girl#uppersecondary  |    .972989   .0076382    -3.49   0.000      .958133    .9880753
               2021#girl#vocational  |   .9750066   .0095023    -2.60   0.009     .9565592    .9938099
                                     |
                            1.degree |    .991061   .0014213    -6.26   0.000     .9882792    .9938505
                                     |
                           immigrant |
             one parent foreign-born  |   1.047592   .0027901    17.46   0.000     1.042138    1.053075
born in Finland parents foreign-born  |   .9700218   .0054785    -5.39   0.000     .9593433    .9808191
     student and parents born abroad  |   1.067454   .0051929    13.42   0.000     1.057324     1.07768
                                     |
                          urbanrural |
                          semiurban  |   .9884453   .0018512    -6.21   0.000     .9848236    .9920802
                              rural  |   .9959326   .0021647    -1.88   0.061     .9916989    1.000184
                                     |
                               _cons |   1.838759   .0056083   199.70   0.000       1.8278    1.849784
-----------------------------------------------------------------------------------------------------

. margins gender, dydx(year) at(schoollevel=(10 20 30)) vsquish

Average marginal effects                                Number of obs = 416,572
Model VCE: Robust

Expression: Predicted mean loneliness, predict()
dy/dx wrt:  2019.year 2021.year
1._at: schoollevel = 10
2._at: schoollevel = 20
3._at: schoollevel = 30

------------------------------------------------------------------------------
             |            Delta-method
             |      dy/dx   std. err.      z    P>|z|     [95% conf. interval]
-------------+----------------------------------------------------------------
2017.year    |  (base outcome)
-------------+----------------------------------------------------------------
2019.year    |
   _at#gender |
     1#boy  |   .0058566   .0071084     0.82   0.410    -.0080757    .0197889
     1#girl  |   .0905101   .0073988    12.23   0.000     .0760088    .1050115
      2#boy  |   .0520413   .0106112     4.90   0.000     .0312438    .0728388
     2#girl  |   .0861629   .0091919     9.37   0.000     .0681472    .1041786
      3#boy  |   .0144795   .0116467     1.24   0.214    -.0083476    .0373066
     3#girl  |    .095841   .0149702     6.40   0.000        .0665     .125182
-------------+----------------------------------------------------------------
2021.year    |
   _at#gender |
     1#boy  |   .2087367   .0073393    28.44   0.000      .194352    .2231214
     1#girl  |   .3649705   .0074302    49.12   0.000     .3504075    .3795335
      2#boy  |   .2638867   .0108236    24.38   0.000     .2426728    .2851006
     2#girl  |   .3457283   .0091712    37.70   0.000     .3277531    .3637034
      3#boy  |   .2642131   .0124555    21.21   0.000     .2398008    .2886254
     3#girl  |   .3860475   .0152482    25.32   0.000     .3561615    .4159335
------------------------------------------------------------------------------
Note: dy/dx for factor levels is the discrete change from the base level.

. margins, dydx(degree) vsquish

Average marginal effects                                Number of obs = 416,572
Model VCE: Robust

Expression: Predicted mean loneliness, predict()
dy/dx wrt:  1.degree

------------------------------------------------------------------------------
             |            Delta-method
             |      dy/dx   std. err.      z    P>|z|     [95% conf. interval]
-------------+----------------------------------------------------------------
    1.degree |     -.0206   .0032916    -6.26   0.000    -.0270514   -.0141487
------------------------------------------------------------------------------
Note: dy/dx for factor levels is the discrete change from the base level.

. margins, dydx(urbanrural) vsquish

Average marginal effects                                Number of obs = 416,572
Model VCE: Robust

Expression: Predicted mean loneliness, predict()
dy/dx wrt:  2.urbanrural 3.urbanrural

------------------------------------------------------------------------------
             |            Delta-method
             |      dy/dx   std. err.      z    P>|z|     [95% conf. interval]
-------------+----------------------------------------------------------------
  urbanrural |
  semiurban  |  -.0265631   .0042654    -6.23   0.000    -.0349232    -.018203
      rural  |  -.0093505   .0049794    -1.88   0.060    -.0191099    .0004089
------------------------------------------------------------------------------
Note: dy/dx for factor levels is the discrete change from the base level.

. margins, dydx(immigrant) vsquish

Average marginal effects                                Number of obs = 416,572
Model VCE: Robust

Expression: Predicted mean loneliness, predict()
dy/dx wrt:  2.immigrant 3.immigrant 4.immigrant

-----------------------------------------------------------------------------------------------------
                                     |            Delta-method
                                     |      dy/dx   std. err.      z    P>|z|     [95% conf. interval]
-------------------------------------+---------------------------------------------------------------
```

```
                      immigrant |
          one parent foreign-born  |   .1086152   .0063465    17.11   0.000     .0961762    .1210542
born in Finland parents foreign-born  |  -.0684167   .0125098    -5.47   0.000    -.0929354    -.043898
     student and parents born abroad  |   .1539434   .0118342    13.01   0.000     .1307488    .1771381
-----------------------------------------------------------------------------------------------------
Note: dy/dx for factor levels is the discrete change from the base level.


*Interactions year*gender*school level with parental education, urban-rural location of the school and immigration status of the student


. glm loneliness i.year##i.schoollevel##i.urbanrural i.gender i.immigrant i.degree, family(gamma) link (log) vce(robust) eform nolog

Generalized linear models                         Number of obs   =    416,572
Optimization     : ML                             Residual df     =    416,540
                                                  Scale parameter =   .1957291
Deviance         =  84559.40057                   (1/df) Deviance =   .2030043
Pearson          =  81529.00748                   (1/df) Pearson  =   .1957291

Variance function: V(u) = u^2                     [Gamma]
Link function    : g(u) = ln(u)                   [Log]

                                                  AIC             =   3.637639
Log pseudolikelihood =  -757637.253               BIC             =   -5305391

-----------------------------------------------------------------------------------------------------
                                     |               Robust
                          loneliness |     exp(b)   std. err.      z    P>|z|     [95% conf. interval]
-------------------------------------+---------------------------------------------------------------
                                year |
                                2019 |   1.020694   .0030767     6.80   0.000     1.014682    1.026742
                                2021 |   1.134475   .0033247    43.05   0.000     1.127978     1.14101
                                     |
                         schoollevel |
                     uppersecondary  |   1.061768    .003787    16.80   0.000     1.054372    1.069217
                         vocational  |   1.022295   .0041956     5.37   0.000     1.014104    1.030551
                                     |
                    year#schoollevel |
                2019#uppersecondary  |   1.013978   .0047389     2.97   0.003     1.004732    1.023309
                    2019#vocational  |   1.004519    .005835     0.78   0.438     .9931477    1.016021
                2021#uppersecondary  |   1.001337   .0045419     0.29   0.768     .9924742    1.010279
                    2021#vocational  |   1.019895   .0058171     3.45   0.001     1.008557     1.03136
                                     |
                          urbanrural |
                          semiurban  |   .9940794   .0047104    -1.25   0.210       .98489    1.003355
                              rural  |   .9948614   .0050506    -1.01   0.310     .9850116     1.00481
                                     |
                     year#urbanrural |
                     2019#semiurban  |   .9991764     .00638    -0.13   0.897     .9867497     1.01176
                         2019#rural  |   1.005783   .0069598     0.83   0.405     .9922338    1.019516
                     2021#semiurban  |   .9992609   .0062218    -0.12   0.905     .9871404     1.01153
                         2021#rural  |   1.002146   .0067699     0.32   0.751     .9889648    1.015503
                                     |
              schoollevel#urbanrural |
           uppersecondary#semiurban  |   .9908932   .0079897    -1.13   0.257     .9753568    1.006677
               uppersecondary#rural  |   1.016428    .009037     1.83   0.067     .9988691    1.034295
               vocational#semiurban  |   .9951215   .0095445    -0.51   0.610     .9765893    1.014005
                   vocational#rural  |   1.010493   .0150388     0.70   0.483     .9814432    1.040403
                                     |
         year#schoollevel#urbanrural |
      2019#uppersecondary#semiurban  |   .9936559   .0106633    -0.59   0.553     .9729744    1.014777
          2019#uppersecondary#rural  |   .9671395   .0117035    -2.76   0.006     .9444709    .9903522
          2019#vocational#semiurban  |   .9809981    .013509    -1.39   0.164      .954875    1.007836
              2019#vocational#rural  |   .9738228   .0201099    -1.28   0.199     .9351951    1.014046
      2021#uppersecondary#semiurban  |   1.008585   .0105045     0.82   0.412      .988205    1.029385
          2021#uppersecondary#rural  |   .9852451   .0115564    -1.27   0.205     .9628534    1.008158
          2021#vocational#semiurban  |   .9724017   .0130431    -2.09   0.037     .9471708    .9983048
              2021#vocational#rural  |   .9594721   .0194785    -2.04   0.042     .9220445     .998419
                                     |
                              gender |
                               girl  |   1.306081   .0018217   191.45   0.000     1.302516    1.309656
                                     |
                           immigrant |
             one parent foreign-born |   1.047916   .0027903    17.58   0.000     1.042462      1.0534
born in Finland parents foreign-born |   .9706383   .0054851    -5.27   0.000      .959947    .9814486
     student and parents born abroad |   1.066528   .0051796    13.26   0.000     1.056425    1.076729
                                     |
                            1.degree |   .9917263   .0014227    -5.79   0.000     .9889418    .9945186
                               _cons |   1.827854   .0047091   234.11   0.000     1.818648    1.837107
-----------------------------------------------------------------------------------------------------

. margins schoollevel, dydx(year) at(urbanrural=(1 2 3)) vsquish

Average marginal effects                               Number of obs = 416,572
Model VCE: Robust

Expression: Predicted mean loneliness, predict()
dy/dx wrt:  2019.year 2021.year
1._at: urbanrural = 1
2._at: urbanrural = 2
3._at: urbanrural = 3

--------------------------------------------------------------------------------
                  |            Delta-method
                  |      dy/dx   std. err.      z    P>|z|     [95% conf. interval]
------------------+-------------------------------------------------------------
2017.year         |  (base outcome)
------------------+-------------------------------------------------------------
2019.year         |
  _at#schoollevel |
1#lowersecondary  |     .043961    .006461     6.80   0.000     .0312976    .0566244
1#uppersecondary  |    .0788563   .0081758     9.65   0.000      .062832    .0948806
    1#vocational  |    .0549584   .0109304     5.03   0.000     .0335351    .0763816
2#lowersecondary  |    .0419254    .011997     3.49   0.000     .0184117    .0654391
2#uppersecondary  |    .0612057   .0176508     3.47   0.001     .0266108    .0958007
    2#vocational  |    .0107321   .0240058     0.45   0.655    -.0363183    .0577826
3#lowersecondary  |    .0562089   .0133311     4.22   0.000     .0300804    .0823374
3#uppersecondary  |    .0153729   .0211929     0.73   0.468    -.0261645    .0569103
    3#vocational  |    .0092589   .0411542     0.22   0.822    -.0714019    .0899198
------------------+-------------------------------------------------------------
2021.year         |
  _at#schoollevel |
```

```
1#lowersecondary  |   .2856672   .0065732    43.46   0.000      .272784    .2985503
1#uppersecondary  |    .306733   .0082302    37.27   0.000     .2906022    .3228638
    1#vocational  |   .3410515   .0115214    29.60   0.000     .3184699    .3636331
2#lowersecondary  |   .2822052   .0122945    22.95   0.000     .2581083     .306302
2#uppersecondary  |   .3219258   .0178793    18.01   0.000     .2868831    .3569686
    2#vocational  |   .2669919   .0247578    10.78   0.000     .2184675    .3155163
3#lowersecondary  |   .2893444   .0136814    21.15   0.000     .2625294    .3161594
3#uppersecondary  |   .2774184   .0214875    12.91   0.000     .2353037    .3195331
    3#vocational  |   .2456852   .0425693     5.77   0.000     .1622509    .3291196
-----------------------------------------------------------------------------------
Note: dy/dx for factor levels is the discrete change from the base level.

. margins, dydx(gender) vsquish

Average marginal effects                             Number of obs = 416,572
Model VCE: Robust

Expression: Predicted mean loneliness, predict()
dy/dx wrt:  2.gender

------------------------------------------------------------------------------
             |            Delta-method
             |      dy/dx   std. err.      z    P>|z|     [95% conf. interval]
-------------+----------------------------------------------------------------
      gender |
        girl  |   .6040088    .003092   195.34   0.000     .5979486     .610069
------------------------------------------------------------------------------
Note: dy/dx for factor levels is the discrete change from the base level.

. margins, dydx(degree) vsquish

Average marginal effects                             Number of obs = 416,572
Model VCE: Robust

Expression: Predicted mean loneliness, predict()
dy/dx wrt:  1.degree

------------------------------------------------------------------------------
             |            Delta-method
             |      dy/dx   std. err.      z    P>|z|     [95% conf. interval]
-------------+----------------------------------------------------------------
    1.degree |  -.0190606   .0032926    -5.79   0.000    -.0255139   -.0126072
------------------------------------------------------------------------------
Note: dy/dx for factor levels is the discrete change from the base level.

. margins, dydx(immigrant) vsquish

Average marginal effects                             Number of obs = 416,572
Model VCE: Robust

Expression: Predicted mean loneliness, predict()
dy/dx wrt:  2.immigrant 3.immigrant 4.immigrant

-----------------------------------------------------------------------------------------------------
                                     |            Delta-method
                                     |      dy/dx   std. err.      z    P>|z|     [95% conf. interval]
-------------------------------------+---------------------------------------------------------------
                           immigrant |
             one parent foreign-born  |   .1093584   .0063472    17.23   0.000     .0969181    .1217987
born in Finland parents foreign-born  |  -.0670118    .012525    -5.35   0.000    -.0915603   -.0424633
     student and parents born abroad  |   .1518367   .0118046    12.86   0.000     .1287001    .1749732
-----------------------------------------------------------------------------------------------------
Note: dy/dx for factor levels is the discrete change from the base level.
```

4.3. Belonging at school with exponentiated Beta and marginal effects

```
*Main effects only

. glm belonging i.year i.gender i.schoollevel i.degree  i.immigrant i.urbanrural, family(gaussian) link (identity) vce(robust) eform nolog

Generalized linear models                         Number of obs   =    415,367
Optimization     : ML                             Residual df     =    415,355
                                                  Scale parameter =   .8968263
Deviance         =  372501.3057                   (1/df) Deviance =   .8968263
Pearson          =  372501.3057                   (1/df) Pearson  =   .8968263

Variance function: V(u) = 1                       [Gaussian]
Link function    : g(u) = u                       [Identity]

                                                  AIC             =   2.729013
Log pseudolikelihood = -566758.9554               BIC             =   -5000912

-----------------------------------------------------------------------------------------------------
                                     |               Robust
                           belonging |     exp(b)   std. err.      z    P>|z|     [95% conf. interval]
-------------------------------------+---------------------------------------------------------------
                                year |
                                2019  |     .80768    .003002   -57.46   0.000     .8018175    .8135854
                                2021  |   .7517688   .0027941   -76.77   0.000     .7463124    .7572652
                                     |
                              gender |
                                girl  |     .70859   .0021026  -116.09   0.000     .7044809     .712723
                                     |
                         schoollevel |
                      uppersecondary  |   1.029975   .0034859     8.73   0.000     1.023165     1.03683
                          vocational  |   1.199418   .0050143    43.50   0.000     1.189631    1.209287
                                     |
                            1.degree |   1.092848   .0033566    28.91   0.000     1.086289    1.099447
                                     |
                           immigrant |
             one parent foreign-born  |   .9446422   .0055947    -9.62   0.000     .9337401    .9556716
born in Finland parents foreign-born  |   1.035142   .0115749     3.09   0.002     1.012702    1.058079
     student and parents born abroad  |   .9252678   .0092725    -7.75   0.000     .9072713    .9436213
                                     |
                          urbanrural |
                          semiurban  |   1.028316   .0040893     7.02   0.000     1.020332    1.036362
```

```
                             rural  |   1.065972   .0050306    13.54   0.000     1.056158    1.075878
                                    |
                             _cons  |   44.33223   .1820223   923.49   0.000     43.97691    44.69043
---------------------------------------------------------------------------------------------------------

. margins, dydx(year) vsquish

Average marginal effects                              Number of obs = 415,367
Model VCE: Robust

Expression: Predicted mean belonging, predict()
dy/dx wrt:  2019.year 2021.year

------------------------------------------------------------------------------
             |            Delta-method
             |      dy/dx   std. err.      z    P>|z|     [95% conf. interval]
-------------+----------------------------------------------------------------
        year |
       2019  |  -.2135893   .0037169   -57.46   0.000    -.2208742   -.2063044
       2021  |  -.2853264   .0037167   -76.77   0.000     -.292611   -.2780418
------------------------------------------------------------------------------
Note: dy/dx for factor levels is the discrete change from the base level.

. margins, dydx(gender) vsquish

Average marginal effects                              Number of obs = 415,367
Model VCE: Robust

Expression: Predicted mean belonging, predict()
dy/dx wrt:  2.gender

------------------------------------------------------------------------------
             |            Delta-method
             |      dy/dx   std. err.      z    P>|z|     [95% conf. interval]
-------------+----------------------------------------------------------------
      gender |
       girl  |  -.3444783   .0029673  -116.09   0.000    -.3502941   -.3386624
------------------------------------------------------------------------------
Note: dy/dx for factor levels is the discrete change from the base level.

. margins, dydx(schoollevel) vsquish

Average marginal effects                              Number of obs = 415,367
Model VCE: Robust

Expression: Predicted mean belonging, predict()
dy/dx wrt:  20.schoollevel 30.schoollevel

---------------------------------------------------------------------------------
                |            Delta-method
                |      dy/dx   std. err.      z    P>|z|     [95% conf. interval]
----------------+----------------------------------------------------------------
    schoollevel |
uppersecondary  |   .0295346   .0033845     8.73   0.000     .0229012    .0361681
    vocational  |   .1818368   .0041806    43.50   0.000      .173643    .1900306
---------------------------------------------------------------------------------
Note: dy/dx for factor levels is the discrete change from the base level.

. margins, dydx(degree) vsquish

Average marginal effects                              Number of obs = 415,367
Model VCE: Robust

Expression: Predicted mean belonging, predict()
dy/dx wrt:  1.degree

------------------------------------------------------------------------------
             |            Delta-method
             |      dy/dx   std. err.      z    P>|z|     [95% conf. interval]
-------------+----------------------------------------------------------------
    1.degree |   .0887873   .0030714    28.91   0.000     .0827675    .0948071
------------------------------------------------------------------------------
Note: dy/dx for factor levels is the discrete change from the base level.

. margins, dydx(urbanrural) vsquish

Average marginal effects                              Number of obs = 415,367
Model VCE: Robust

Expression: Predicted mean belonging, predict()
dy/dx wrt:  2.urbanrural 3.urbanrural

------------------------------------------------------------------------------
             |            Delta-method
             |      dy/dx   std. err.      z    P>|z|     [95% conf. interval]
-------------+----------------------------------------------------------------
  urbanrural |
  semiurban  |   .0279224   .0039767     7.02   0.000     .0201283    .0357166
      rural  |   .0638874   .0047192    13.54   0.000     .0546378    .0731369
------------------------------------------------------------------------------
Note: dy/dx for factor levels is the discrete change from the base level.

. margins, dydx(immigrant) vsquish

Average marginal effects                              Number of obs = 415,367
Model VCE: Robust

Expression: Predicted mean belonging, predict()
dy/dx wrt:  2.immigrant 3.immigrant 4.immigrant

---------------------------------------------------------------------------------------------------------
                                    |            Delta-method
                                    |      dy/dx   std. err.      z    P>|z|     [95% conf. interval]
------------------------------------+--------------------------------------------------------------------
                          immigrant |
             one parent foreign-born  |  -.0569491   .0059226    -9.62   0.000    -.0685571    -.045341
born in Finland parents foreign-born  |   .0345385    .011182     3.09   0.002     .0126222    .0564548
     student and parents born abroad  |  -.0776721   .0100215    -7.75   0.000    -.0973138   -.0580304
---------------------------------------------------------------------------------------------------------
Note: dy/dx for factor levels is the discrete change from the base level.
```

*Interactions year*gender*school level

```
. glm belonging i.year##i.gender##i.schoollevel i.degree  i.immigrant i.urbanrural, family(gaussian) link (identity) vce(robust) eform
nolog

Generalized linear models                         Number of obs   =    415,367
Optimization     : ML                             Residual df     =    415,343
                                                  Scale parameter =   .8953786
Deviance         =  371889.2404                   (1/df) Deviance =   .8953786
Pearson          =  371889.2404                   (1/df) Pearson  =   .8953786

Variance function: V(u) = 1                       [Gaussian]
Link function    : g(u) = u                       [Identity]

                                                  AIC             =   2.727426
Log pseudolikelihood = -566417.4253               BIC             =   -5001369

-----------------------------------------------------------------------------------------------------
                                   |               Robust
                         belonging |     exp(b)   std. err.      z    P>|z|     [95% conf. interval]
-----------------------------------+-----------------------------------------------------------------
                              year |
                             2019  |   .7250146   .0053796   -43.34   0.000     .7145471    .7356355
                             2021  |    .714909   .0052444   -45.75   0.000     .7047037    .7252621
                                   |
                            gender |
                             girl  |    .662022   .0051742   -52.77   0.000     .6519581    .6722413
                                   |
                       year#gender |
                        2019#girl  |   1.101585   .0113934     9.35   0.000      1.07948    1.124144
                        2021#girl  |   1.022854   .0104805     2.21   0.027     1.002518    1.043603
                                   |
                       schoollevel |
                   uppersecondary  |   .8755414   .0086445   -13.46   0.000     .8587613    .8926494
                       vocational  |   1.126472    .010959    12.24   0.000     1.105196    1.148158
                                   |
                  year#schoollevel |
              2019#uppersecondary  |   1.238863   .0161063    16.48   0.000     1.207694    1.270836
                  2019#vocational  |   1.073693    .014196     5.38   0.000     1.046227     1.10188
              2021#uppersecondary  |   1.133702   .0146607     9.70   0.000     1.105329    1.162803
                  2021#vocational  |    1.05801   .0142458     4.19   0.000     1.030454    1.086303
                                   |
                gender#schoollevel |
              girl#uppersecondary  |   1.110183   .0145282     7.99   0.000      1.08207    1.139026
                  girl#vocational  |     1.0716   .0158794     4.67   0.000     1.040924    1.103179
                                   |
           year#gender#schoollevel |
         2019#girl#uppersecondary  |   .9316065   .0160653    -4.11   0.000     .9006452    .9636322
             2019#girl#vocational  |   .9337975   .0189763    -3.37   0.001     .8973356     .971741
         2021#girl#uppersecondary  |   .9755133   .0167467    -1.44   0.149     .9432365    1.008895
             2021#girl#vocational  |   .9699466   .0201003    -1.47   0.141       .93134    1.010154
                                   |
                          1.degree |   1.093919    .003359    29.23   0.000     1.087355    1.100522
                                   |
                         immigrant |
          one parent foreign-born  |   .9440747   .0055869    -9.72   0.000     .9331878    .9550886
born in Finland parents foreign-born  |   1.035397   .0115793     3.11   0.002     1.012949    1.058343
     student and parents born abroad  |   .9232348   .0092523    -7.97   0.000     .9052776    .9415483
                                   |
                        urbanrural |
                        semiurban  |   1.028787   .0040893     7.14   0.000     1.020803    1.036833
                            rural  |   1.065552   .0050252    13.46   0.000     1.055749    1.075447
                                   |
                             _cons |   47.51607   .2850581   643.60   0.000     46.96064    48.07807
-----------------------------------------------------------------------------------------------------

. margins gender, dydx(year) at(schoollevel=(10 20 30)) vsquish

Average marginal effects                                Number of obs = 415,367
Model VCE: Robust

Expression: Predicted mean belonging, predict()
dy/dx wrt:  2019.year 2021.year
1._at: schoollevel = 10
2._at: schoollevel = 20
3._at: schoollevel = 30

------------------------------------------------------------------------------
             |            Delta-method
             |      dy/dx   std. err.      z    P>|z|     [95% conf. interval]
-------------+----------------------------------------------------------------
2017.year    |  (base outcome)
-------------+----------------------------------------------------------------
2019.year    |
     _at#gender |
      1#boy  |  -.3215634     .00742   -43.34   0.000    -.3361064   -.3070205
     1#girl  |   -.224813   .0072114   -31.17   0.000    -.2389471    -.210679
      2#boy  |  -.1073694   .0106794   -10.05   0.000    -.1283006   -.0864383
     2#girl  |  -.0814638    .008743    -9.32   0.000    -.0985997   -.0643279
      3#boy  |  -.2504595   .0109446   -22.88   0.000    -.2719106   -.2290084
     3#girl  |  -.2222047   .0136467   -16.28   0.000    -.2489518   -.1954577
-------------+----------------------------------------------------------------
2021.year    |
     _at#gender |
      1#boy  |     -.3356   .0073358   -45.75   0.000    -.3499779   -.3212222
     1#girl  |   -.313003   .0071632   -43.70   0.000    -.3270427   -.2989634
      2#boy  |  -.2101118   .0106564   -19.72   0.000    -.2309979   -.1892257
     2#girl  |  -.2123063   .0087367   -24.30   0.000    -.2294299   -.1951826
      3#boy  |  -.2792104   .0112934   -24.72   0.000    -.3013452   -.2570757
     3#girl  |  -.2871277   .0140348   -20.46   0.000    -.3146355   -.2596199
------------------------------------------------------------------------------
Note: dy/dx for factor levels is the discrete change from the base level.

. margins, dydx(degree)

Average marginal effects                                Number of obs = 415,367
Model VCE: Robust

Expression: Predicted mean belonging, predict()
dy/dx wrt:  1.degree

------------------------------------------------------------------------------
```

```
             |            Delta-method
             |      dy/dx   std. err.      z    P>|z|     [95% conf. interval]
-------------+----------------------------------------------------------------
    1.degree |   .0897663   .0030706    29.23   0.000      .083748    .0957846
------------------------------------------------------------------------------
Note: dy/dx for factor levels is the discrete change from the base level.

. margins, dydx(urbanrural)

Average marginal effects                               Number of obs = 415,367
Model VCE: Robust

Expression: Predicted mean belonging, predict()
dy/dx wrt:  2.urbanrural 3.urbanrural

------------------------------------------------------------------------------
             |            Delta-method
             |      dy/dx   std. err.      z    P>|z|     [95% conf. interval]
-------------+----------------------------------------------------------------
  urbanrural |
  semiurban  |   .0283806   .0039749     7.14   0.000       .02059    .0361713
      rural  |   .0634933   .0047161    13.46   0.000       .05425    .0727367
------------------------------------------------------------------------------
Note: dy/dx for factor levels is the discrete change from the base level.

. margins, dydx(immigrant)

Average marginal effects                               Number of obs = 415,367
Model VCE: Robust

Expression: Predicted mean belonging, predict()
dy/dx wrt:  2.immigrant 3.immigrant 4.immigrant

-----------------------------------------------------------------------------------------------------
                                      |            Delta-method
                                      |      dy/dx   std. err.      z    P>|z|     [95% conf. interval]
--------------------------------------+--------------------------------------------------------------
                            immigrant |
              one parent foreign-born  |    -.05755   .0059179    -9.72   0.000    -.0691489   -.0459512
born in Finland parents foreign-born  |    .034785   .0111834     3.11   0.002     .0128659    .0567041
     student and parents born abroad  |  -.0798716   .0100216    -7.97   0.000    -.0995136   -.0602296
-----------------------------------------------------------------------------------------------------
Note: dy/dx for factor levels is the discrete change from the base level.


*Interactions year*gender*school level with parental education, urban-rural location of the school and immigration status of the student


. glm belonging i.year##i.gender##i.degree i.year##i.immigrant i.year##i.schoollevel##i.urbanrural, family(gaussian) link (identity)
vce(robust) efo
> rm nolog

Generalized linear models                         Number of obs   =    415,367
Optimization     : ML                             Residual df     =    415,322
                                                  Scale parameter =   .8951855
Deviance         =  371790.2197                   (1/df) Deviance =   .8951855
Pearson          =  371790.2197                   (1/df) Pearson  =   .8951855

Variance function: V(u) = 1                       [Gaussian]
Link function    : g(u) = u                       [Identity]

                                                  AIC             =   2.727261
Log pseudolikelihood = -566362.1193               BIC             =   -5001196

----------------------------------------------------------------------------------------------------------
                                          |               Robust
                                belonging |     exp(b)   std. err.      z    P>|z|     [95% conf. interval]
------------------------------------------+---------------------------------------------------------------
                                     year |
                                     2019 |   .7368374    .007187   -31.31   0.000      .722885     .751059
                                     2021 |   .7194294     .00703   -33.70   0.000      .705782    .7333407
                                          |
                                   gender |
                                     girl |   .6946174   .0054938   -46.07   0.000     .6839328     .705469
                                          |
                              year#gender |
                                2019#girl |   1.058066   .0114531     5.21   0.000     1.035855    1.080754
                                2021#girl |   .9812392    .010738    -1.73   0.084     .9604171    1.002513
                                          |
                                 1.degree |   1.070272   .0087098     8.35   0.000     1.053337     1.08748
                                          |
                              year#degree |
                                   2019 1 |   1.030566   .0112423     2.76   0.006     1.008765    1.052837
                                   2021 1 |   1.012106   .0110432     1.10   0.270     .9906917    1.033984
                                          |
                            gender#degree |
                                   girl#1 |   .9844852   .0109592    -1.40   0.160     .9632381    1.006201
                                          |
                       year#gender#degree |
                              2019#girl#1 |   1.020414   .0151744     1.36   0.174     .9911022    1.050593
                              2021#girl#1 |   1.057488   .0157423     3.75   0.000     1.027079    1.088796
                                          |
                                immigrant |
                  one parent foreign-born |   .9467567   .0110711    -4.68   0.000     .9253046    .9687062
     born in Finland parents foreign-born |   .9865301   .0232867    -0.57   0.566     .9419287    1.033243
          student and parents born abroad |    .865485   .0181339    -6.89   0.000     .8306631    .9017666
                                          |
                           year#immigrant |
             2019#one parent foreign-born |    .995398   .0151725    -0.30   0.762     .9661002    1.025584
2019#born in Finland parents foreign-born |   1.048276   .0312315     1.58   0.114     .9888159    1.111311
     2019#student and parents born abroad |   1.067283   .0284217     2.45   0.014     1.013006    1.124468
             2021#one parent foreign-born |   .9964668   .0150774    -0.23   0.815     .9673495    1.026461
2021#born in Finland parents foreign-born |   1.082569   .0318388     2.70   0.007     1.021931    1.146806
     2021#student and parents born abroad |   1.116579   .0292144     4.21   0.000     1.060763    1.175332
                                          |
                              schoollevel |
                          uppersecondary  |   .9170349   .0072085   -11.02   0.000     .9030147    .9312727
                               vocational |   1.166037   .0100673    17.79   0.000     1.146472    1.185937
                                          |
                         year#schoollevel |
                      2019#uppersecondary |   1.174731   .0120374    15.72   0.000     1.151373    1.198562
                          2019#vocational |   1.016125   .0119436     1.36   0.174     .9929834    1.039806
```

```
       2021#uppersecondary  |   1.124345   .0114673    11.49   0.000     1.102092    1.147046
           2021#vocational  |   1.026028    .012312     2.14   0.032     1.002178    1.050445
                            |
                 urbanrural |
                  semiurban |     1.0346   .0105132     3.35   0.001     1.014199    1.055412
                      rural |   1.079633   .0117944     7.01   0.000     1.056762    1.102999
                            |
            year#urbanrural |
             2019#semiurban |   .9507449   .0129269    -3.71   0.000     .9257433    .9764217
                 2019#rural |   .9429317   .0140508    -3.94   0.000     .9157908    .9708769
             2021#semiurban |   .9816632   .0132202    -1.37   0.169     .9560911    1.007919
                 2021#rural |    .953795   .0140217    -3.22   0.001     .9267051    .9816767
                            |
     schoollevel#urbanrural |
  uppersecondary#semiurban  |   1.051347   .0180855     2.91   0.004      1.01649    1.087398
      uppersecondary#rural  |   1.078847   .0208767     3.92   0.000     1.038695     1.12055
      vocational#semiurban  |   .9951919   .0191668    -0.25   0.802     .9583259    1.033476
          vocational#rural  |   .9239189   .0281258    -2.60   0.009     .8704057    .9807223
                            |
year#schoollevel#urbanrural |
2019#uppersecondary#semiurban |   1.018318   .0233217     0.79   0.428     .9736189    1.065069
    2019#uppersecondary#rural |    1.03385   .0271956     1.27   0.206     .9818987    1.088551
    2019#vocational#semiurban |   1.077456   .0287201     2.80   0.005     1.022611    1.135243
        2019#vocational#rural |   1.244477   .0513034     5.31   0.000     1.147879    1.349204
2021#uppersecondary#semiurban |   .9631757   .0220513    -1.64   0.101     .9209113     1.00738
    2021#uppersecondary#rural |   .9537316   .0251206    -1.80   0.072     .9057455     1.00426
    2021#vocational#semiurban |   1.065729   .0288534     2.35   0.019     1.010652    1.123808
        2021#vocational#rural |   1.181229   .0494073     3.98   0.000     1.088256    1.282146
                            |
                      _cons |   46.98914   .3412204   530.17   0.000      46.3251     47.6627
------------------------------------------------------------------------------------------

. margins gender, dydx(year) at(degree=(0 1)) vsquish

Average marginal effects                            Number of obs = 415,367
Model VCE: Robust

Expression: Predicted mean belonging, predict()
dy/dx wrt:  2019.year 2021.year
1._at: degree = 0
2._at: degree = 1

------------------------------------------------------------------------------
             |            Delta-method
             |      dy/dx   std. err.      z    P>|z|     [95% conf. interval]
-------------+----------------------------------------------------------------
2017.year    |  (base outcome)
-------------+----------------------------------------------------------------
2019.year    |
     _at#gender |
      1#boy  |  -.2628125   .0079422   -33.09   0.000     -.278379   -.2472461
     1#girl  |  -.2063697   .0075275   -27.42   0.000    -.2211234    -.191616
      2#boy  |  -.2327047   .0074045   -31.43   0.000    -.2472173    -.218192
     2#girl  |  -.1560532   .0072412   -21.55   0.000    -.1702458   -.1418606
-------------+----------------------------------------------------------------
2021.year    |
     _at#gender |
      1#boy  |  -.2949602   .0080093   -36.83   0.000    -.3106582   -.2792622
     1#girl  |  -.3138992   .0076233   -41.18   0.000    -.3288406   -.2989579
      2#boy  |  -.2829266   .0073291   -38.60   0.000    -.2972914   -.2685619
     2#girl  |  -.2459697   .0071724   -34.29   0.000    -.2600273   -.2319121
------------------------------------------------------------------------------
Note: dy/dx for factor levels is the discrete change from the base level.

. margins, dydx(year) at(immigrant=(1 2 3 4)) vsquish

Average marginal effects                            Number of obs = 415,367
Model VCE: Robust

Expression: Predicted mean belonging, predict()
dy/dx wrt:  2019.year 2021.year
1._at: immigrant = 1
2._at: immigrant = 2
3._at: immigrant = 3
4._at: immigrant = 4

------------------------------------------------------------------------------
             |            Delta-method
             |      dy/dx   std. err.      z    P>|z|     [95% conf. interval]
-------------+----------------------------------------------------------------
2017.year    |  (base outcome)
-------------+----------------------------------------------------------------
2019.year    |
         _at |
          1  |  -.2133841   .0039188   -54.45   0.000    -.2210649   -.2057034
          2  |  -.2179968   .0147213   -14.81   0.000    -.2468499   -.1891437
          3  |  -.1662375   .0295247    -5.63   0.000    -.2241048   -.1083702
          4  |  -.1482682   .0263399    -5.63   0.000    -.1998933    -.096643
-------------+----------------------------------------------------------------
2021.year    |
         _at |
          1  |  -.2870841   .0039215   -73.21   0.000      -.29477   -.2793982
          2  |  -.2906236   .0146087   -19.89   0.000    -.3192561    -.261991
          3  |  -.2077471   .0291368    -7.13   0.000    -.2648542   -.1506399
          4  |  -.1768148   .0258676    -6.84   0.000    -.2275143   -.1261153
------------------------------------------------------------------------------
Note: dy/dx for factor levels is the discrete change from the base level.

. margins schoollevel, dydx(year) at(urbanrural=(1 2 3)) vsquish

Average marginal effects                            Number of obs = 415,367
Model VCE: Robust

Expression: Predicted mean belonging, predict()
dy/dx wrt:  2019.year 2021.year
1._at: urbanrural = 1
2._at: urbanrural = 2
3._at: urbanrural = 3

-----------------------------------------------------------------------------------
                  |            Delta-method
                  |      dy/dx   std. err.      z    P>|z|     [95% conf. interval]
```

2017.year	(base outcome)					
2019.year						
_at#schoollevel						
1#lowersecondary	-.2508338	.0063628	-39.42	0.000	-.2633047	-.238363
1#uppersecondary	-.089795	.0080632	-11.14	0.000	-.1055986	-.0739915
1#vocational	-.2348375	.0098475	-23.85	0.000	-.2541382	-.2155368
2#lowersecondary	-.3013433	.0119892	-25.13	0.000	-.3248418	-.2778449
2#uppersecondary	-.1221524	.0167207	-7.31	0.000	-.1549244	-.0893803
2#vocational	-.2107444	.0209936	-10.04	0.000	-.2518911	-.1695978
3#lowersecondary	-.3095953	.0134439	-23.03	0.000	-.3359449	-.2832457
3#uppersecondary	-.1152666	.0202723	-5.69	0.000	-.1549995	-.0755336
3#vocational	-.0748837	.0373049	-2.01	0.045	-.1479999	-.0017675
2021.year						
_at#schoollevel						
1#lowersecondary	-.3121514	.0063212	-49.38	0.000	-.3245408	-.299762
1#uppersecondary	-.1949512	.0080499	-24.22	0.000	-.2107288	-.1791736
1#vocational	-.2864567	.0101614	-28.19	0.000	-.3063727	-.2665408
2#lowersecondary	-.3306584	.0118612	-27.88	0.000	-.3539059	-.307411
2#uppersecondary	-.2509776	.0168301	-14.91	0.000	-.283964	-.2179912
2#vocational	-.2413047	.0214465	-11.25	0.000	-.283339	-.1992704
3#lowersecondary	-.359458	.0132404	-27.15	0.000	-.3854087	-.3335073
3#uppersecondary	-.2896307	.020469	-14.15	0.000	-.3297491	-.2495123
3#vocational	-.1672076	.0379523	-4.41	0.000	-.2415928	-.0928223

Note: dy/dx for factor levels is the discrete change from the base level.
