# Supplementary material for: The cohort trends of social connectedness in secondary school students in Finland between 2017 and 2021
Source: PLoS One. 2024 Oct 28;19(10):e0312579. doi: 10.1371/journal.pone.0312579 (PMC11516007; doi:10.1371/journal.pone.0312579)
Supplement: S2 File — (PDF) [file pone.0312579.s002.pdf]

S2 Online resource.
Supplementary figures

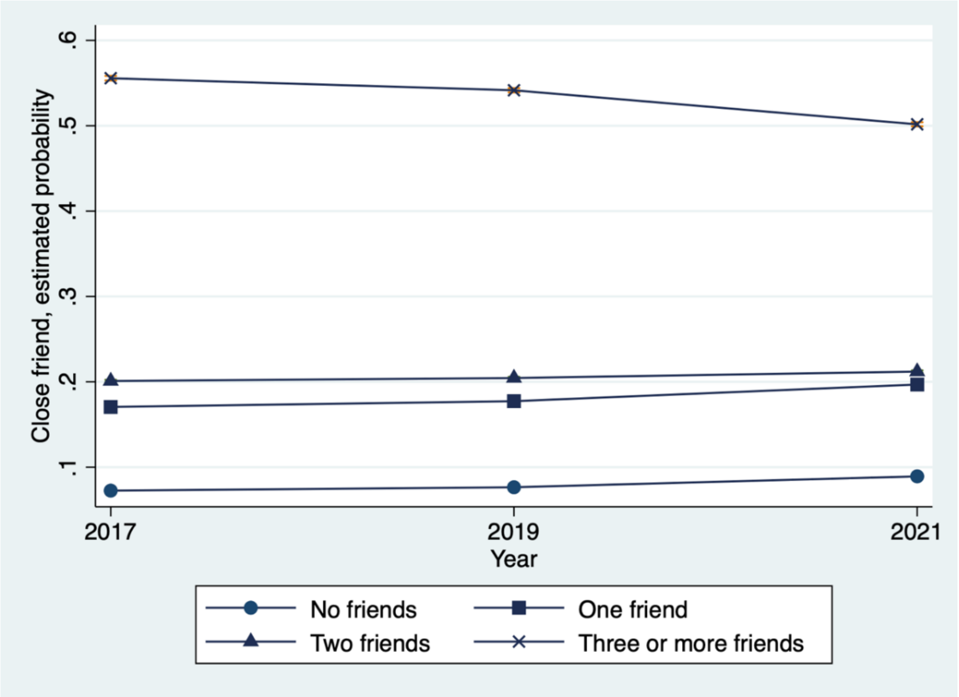

Supplementary Figure 1. Number of close friends in secondary (lower, upper and vocational) school students in Finland between 2017 and 2021. The estimated probabilities are shown from a model adjusted for school level, gender, parental education, immigration status of the student and urban/rural location of the school. Spikes indicate the 95% confidence interval.

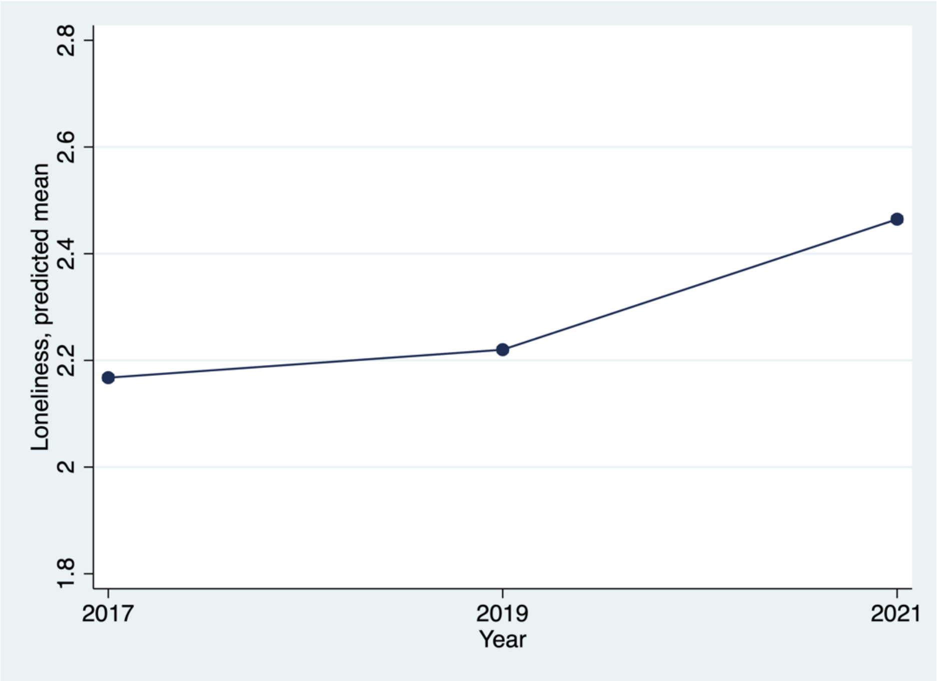

Supplementary Figure 2. Loneliness mean in secondary (lower, upper and vocational) school students in Finland between 2017 and 2021. The estimated probabilities are shown from a model adjusted for school level, gender, parental education, immigration status of the student and urban/rural location of the school. Spikes indicate the 95% confidence interval.

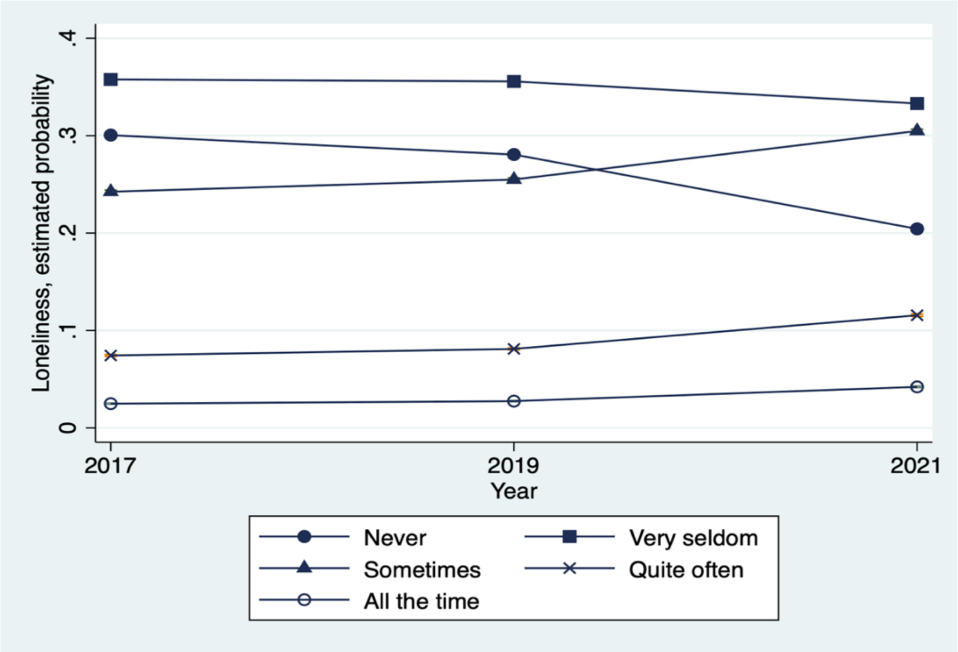

Supplementary Figure 3. Loneliness categories in secondary (lower, upper and vocational) school students in Finland between 2017 and 2021. The estimated probabilities are shown from a model adjusted for school level, gender, parental education, immigration status of the student and urban/rural location of the school. Spikes indicate the 95% confidence interval.

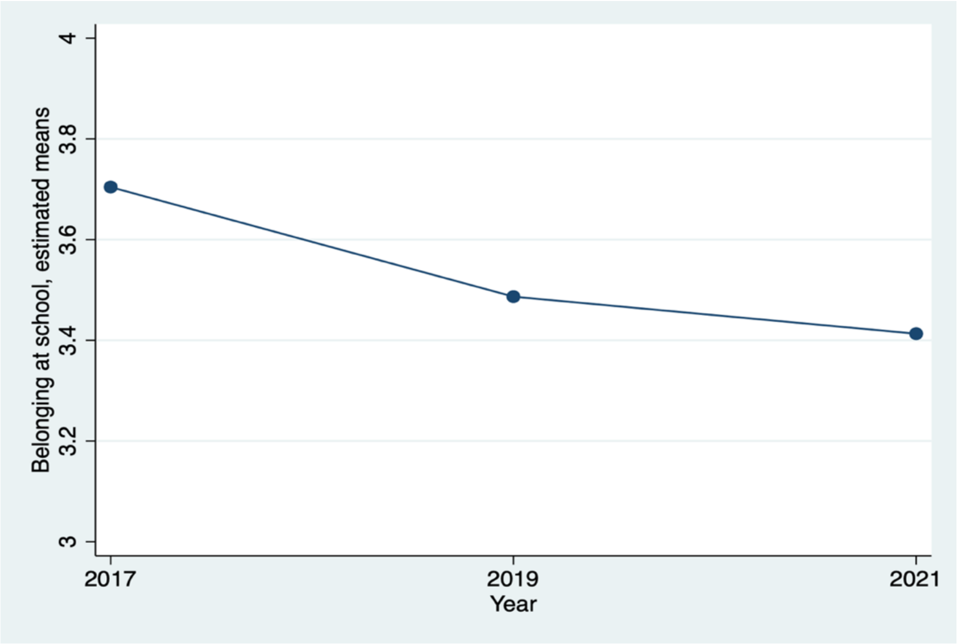

Supplementary Figure 4. Belonging at school in secondary (lower, upper and vocational) school students in Finland between 2017 and 2021. The estimated probabilities are shown from a model adjusted for school level, gender, parental education, immigration status of the student and urban/rural location of the school. Spikes indicate the 95% confidence interval.

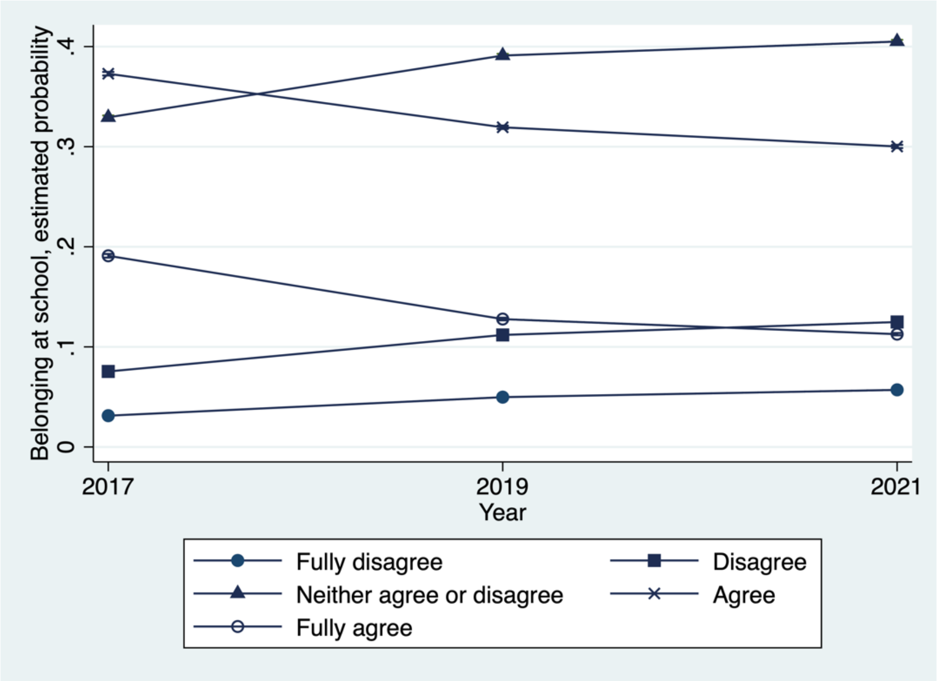

Supplementary Figure 5. Belonging at school categories in secondary (lower, upper and vocational) school students in Finland between 2017 and 2021. The estimated mean is shown from a model adjusted for school level, gender, parental education, immigration status of the student and urban/rural location of the school. Spikes indicate the 95% confidence interval.

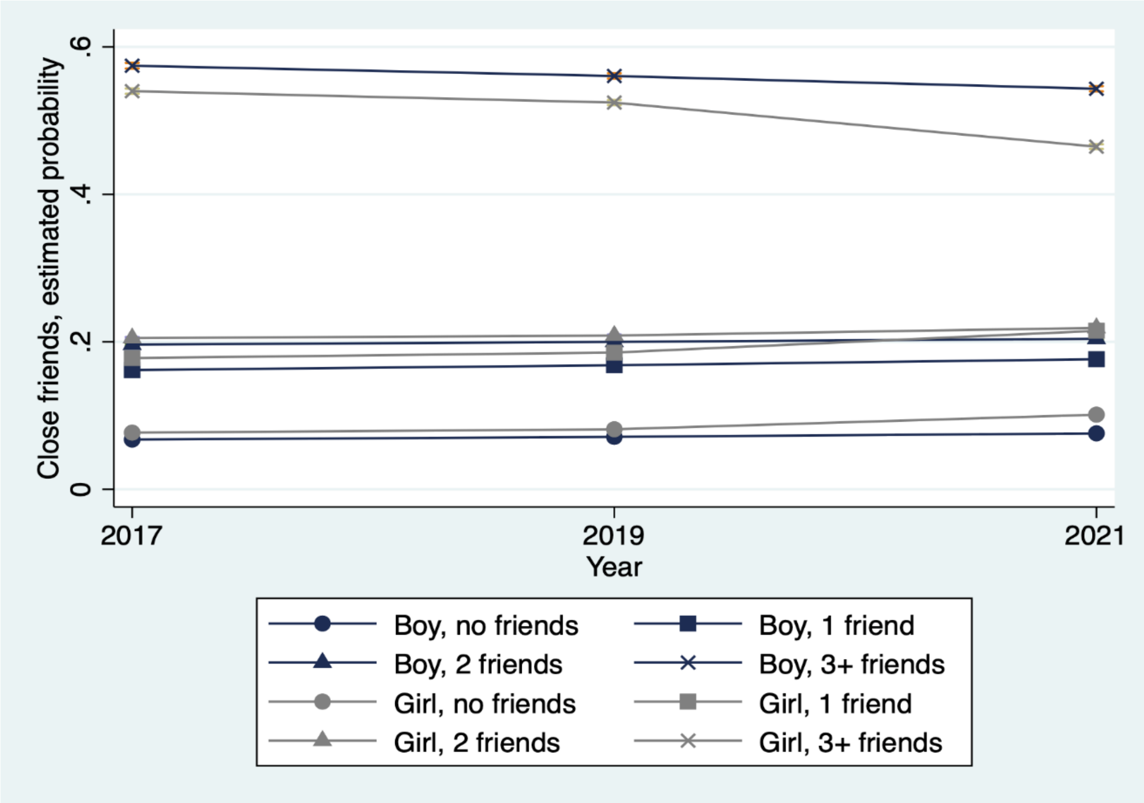

Supplementary Figure 6. Having close friends by gender in secondary (lower, upper and vocational) school students in Finland between 2017 and 2021. The estimated probabilities are shown from a model including an interaction year*gender and the main effects of school level, parental education, immigration status of the student and urban/rural location of the school. Spikes indicate the 95% confidence interval.

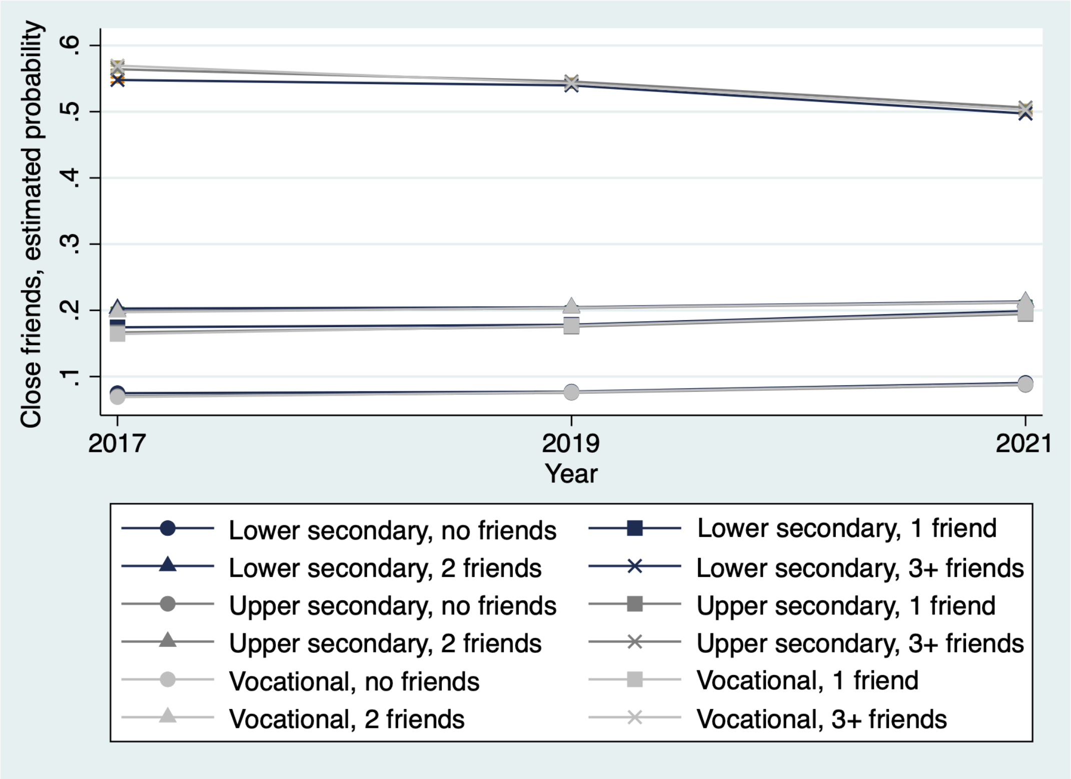

Supplementary Figure 7. Number of close friends by school level in secondary (lower, upper and vocational) school students in Finland between 2017 and 2021. The estimated probabilities are shown from a model including an interaction year*school level and the main effects of gender, parental education, immigration status of the student and urban/rural location of the school. Spikes indicate the 95% confidence interval.

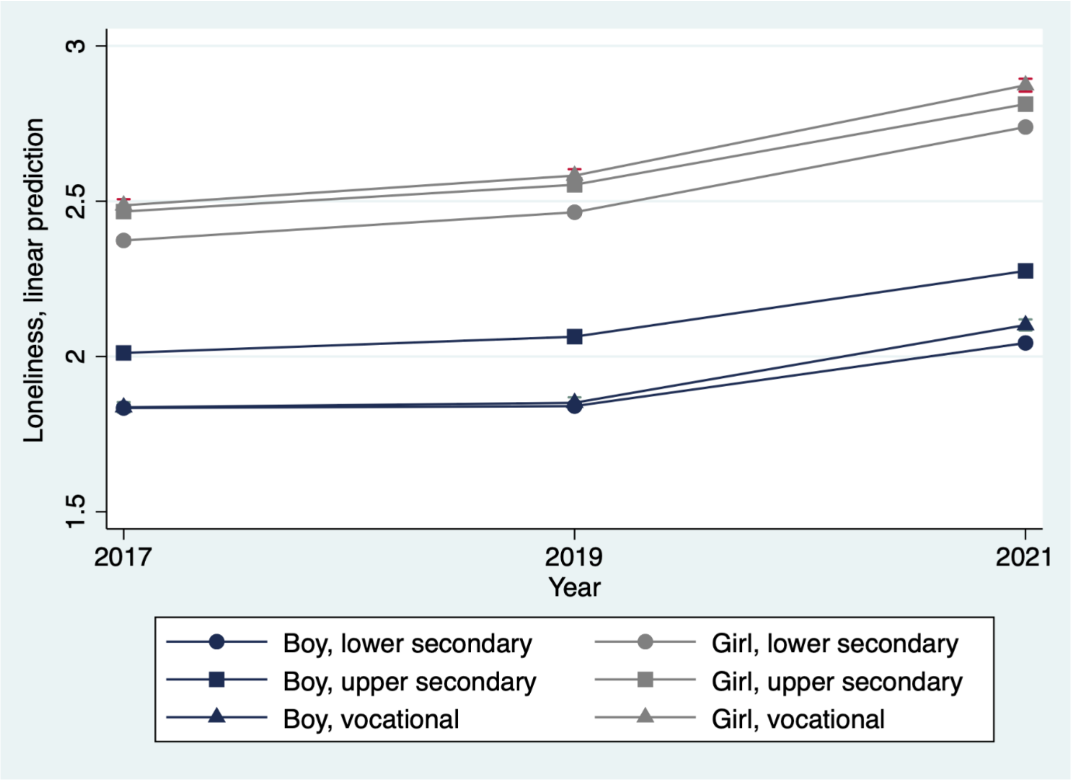

Supplementary Figure 8. Loneliness by gender and school level in secondary (lower, upper and vocational) school students in Finland between 2017 and 2021. The estimated linear predictions are shown from a model including an interaction year*gender*school level and the main effects of parental education, immigration status of the student and urban/rural location of the school. Spikes indicate the 95% confidence interval.

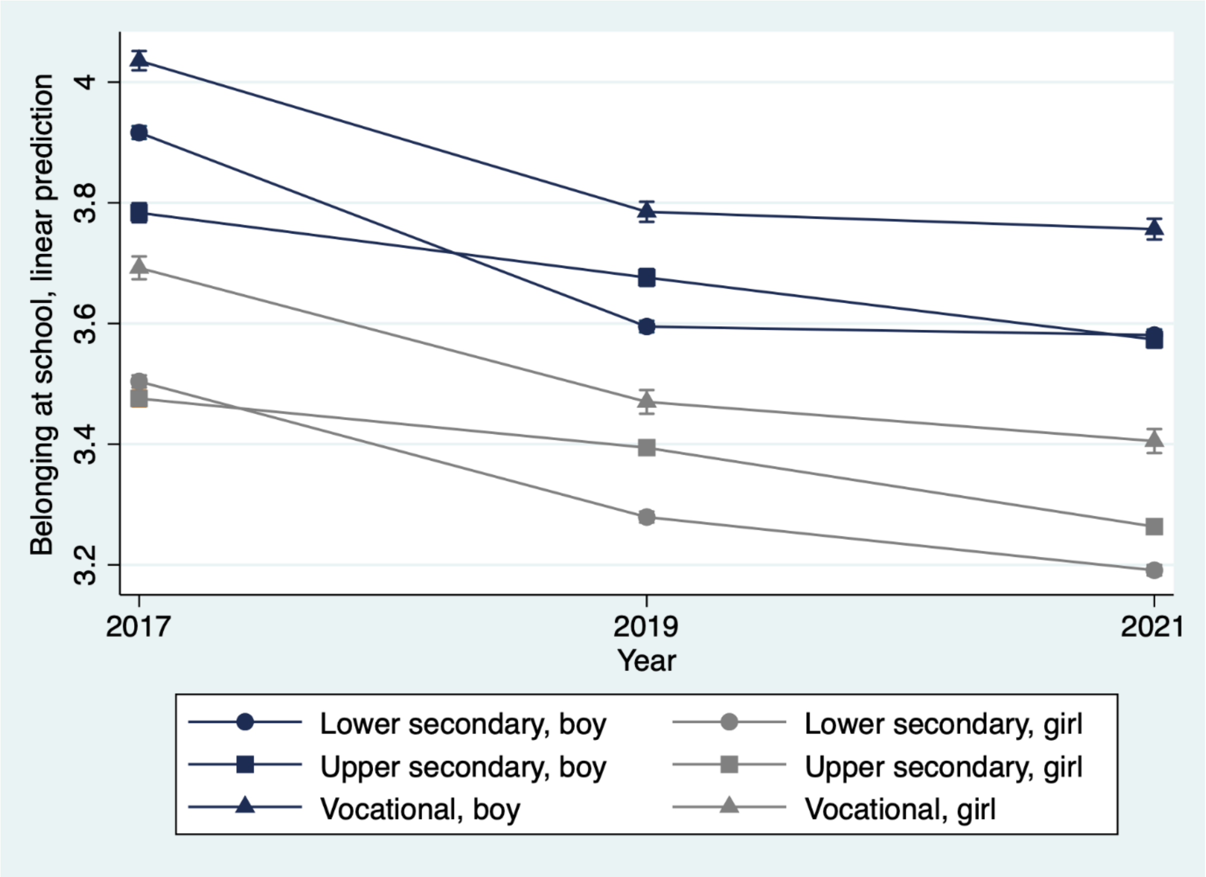

Supplementary Figure 9. Belonging at school by gender and school level in secondary (lower, upper and vocational) school students in Finland between 2017 and 2021. The linear predictions are shown from a model including an interaction year*gender*school level and the main effects of parental education, immigration status of the student and urban/rural location of the school. Spikes indicate the 95% confidence interval.

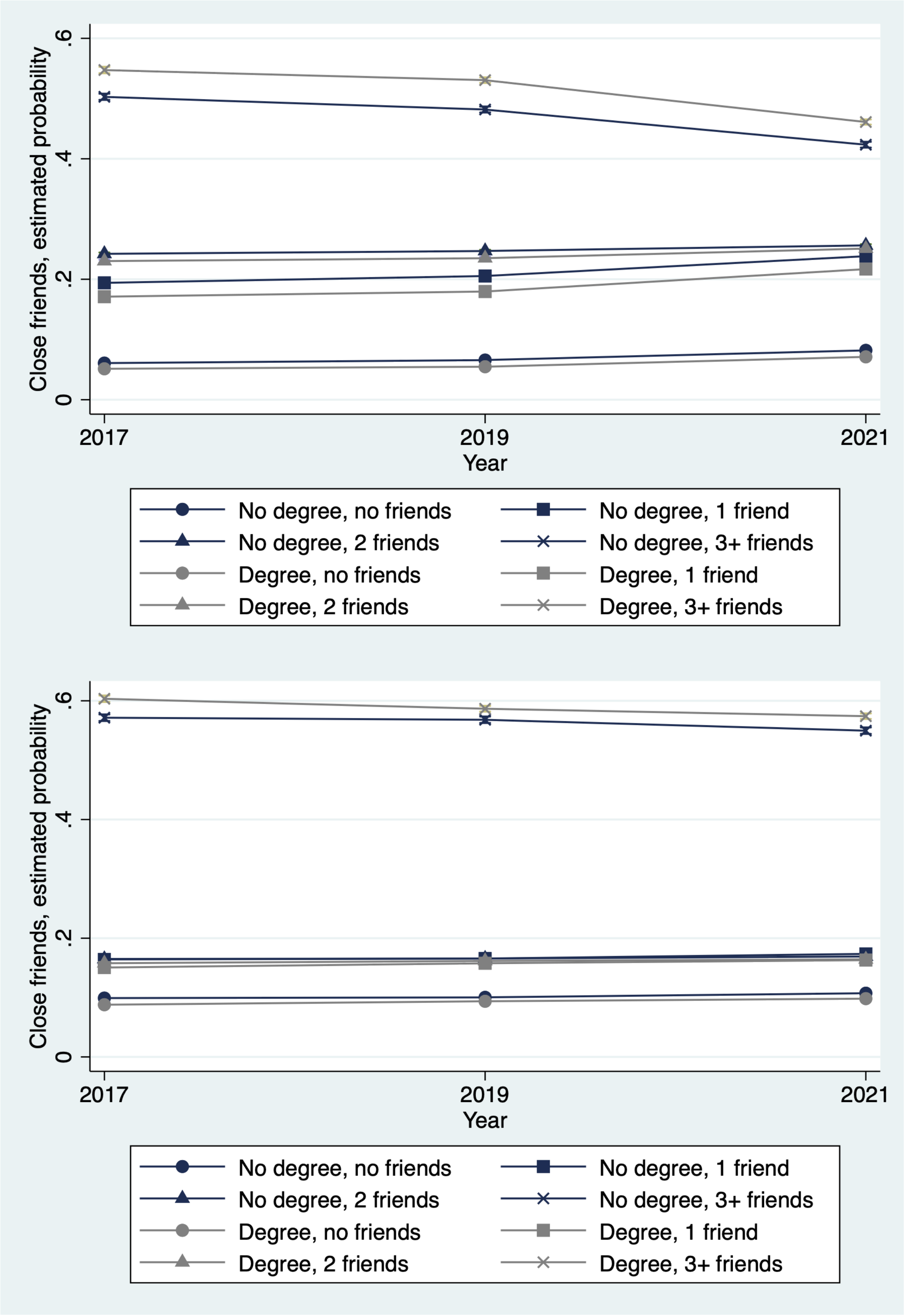

Supplementary Figure 10. Number of close friends by parental degree in girls (top figure) and boys (bottom figure) in secondary (lower, upper and vocational) school students in Finland between 2017 and 2021. The estimated probabilities are shown from a model including an interaction year*gender*parental degree and the main effects of school level, immigration status of the student and urban/rural location of the school. Spikes indicate the 95% confidence interval.

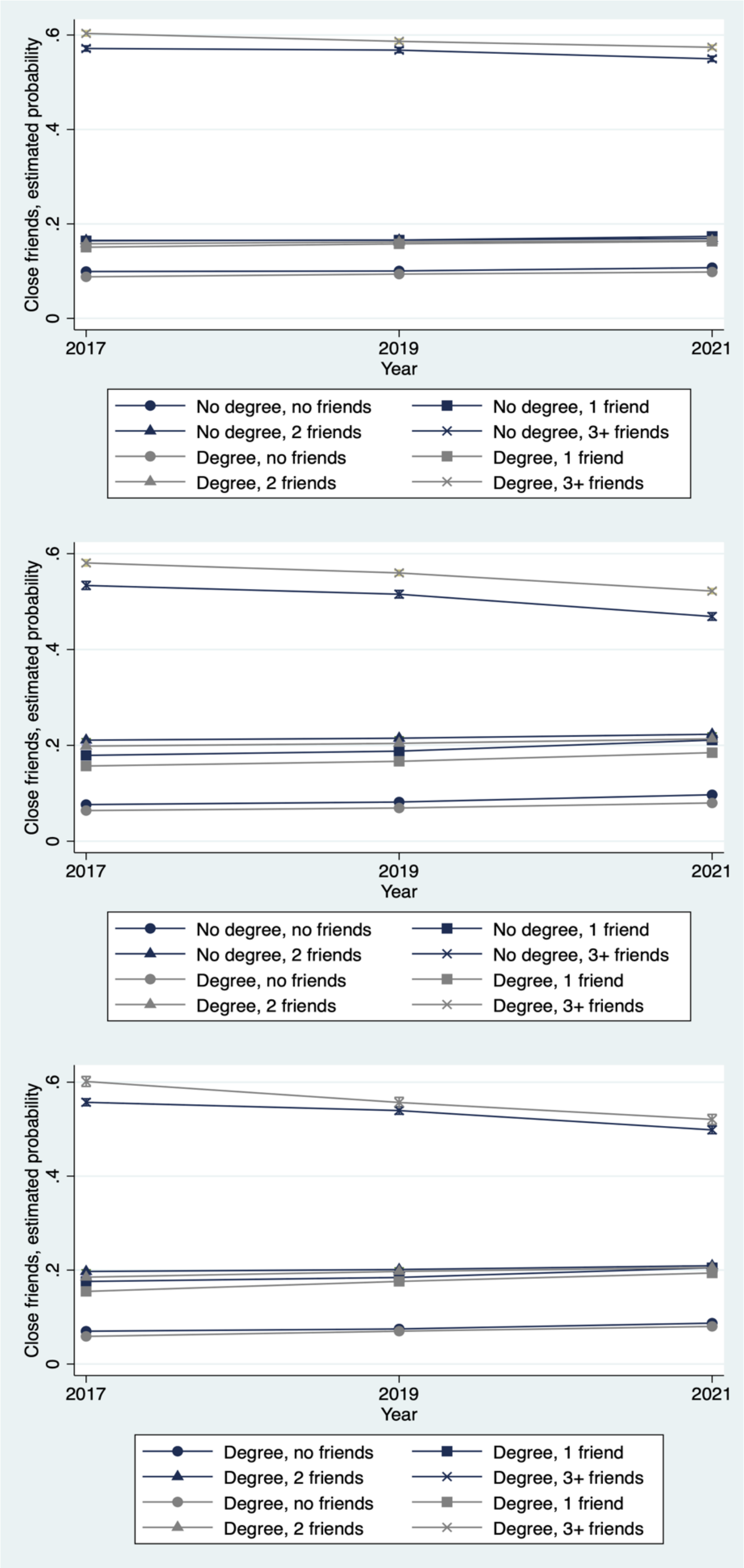

Supplementary Figure 11. Number of close friends by parental degree in lower secondary (top figure), upper secondary (middle figure) and vocational school (bottom figure) in Finland between 2017 and 2021. The estimated probabilities are shown from a model including an interaction year*school level*parental degree and the main effects of gender, immigration status of the student and urban/rural location of the school. Spikes indicate the 95% confidence interval.

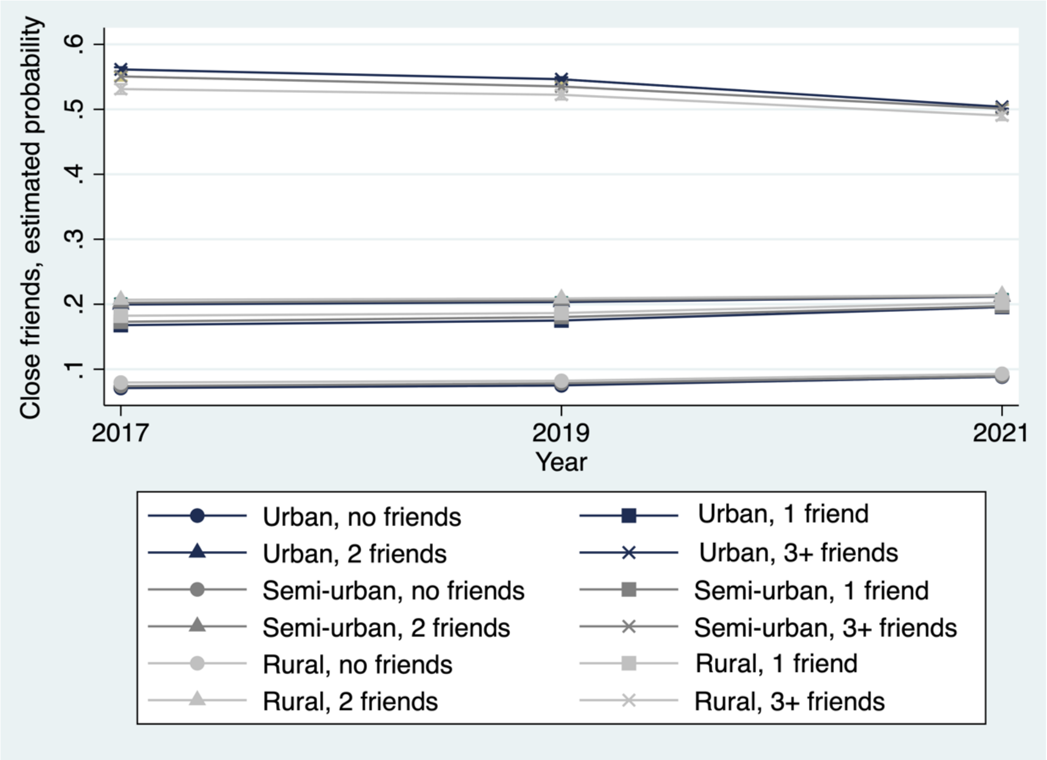

Supplementary Figure 12. Number of close friends by urban/rural location of the school in Finland between 2017 and 2021. The estimated probabilities are shown from a model including an interaction year*urban/rural location of the school and the main effects of gender, school level, parental degree and immigration status of the student. Spikes indicate the 95% confidence interval.

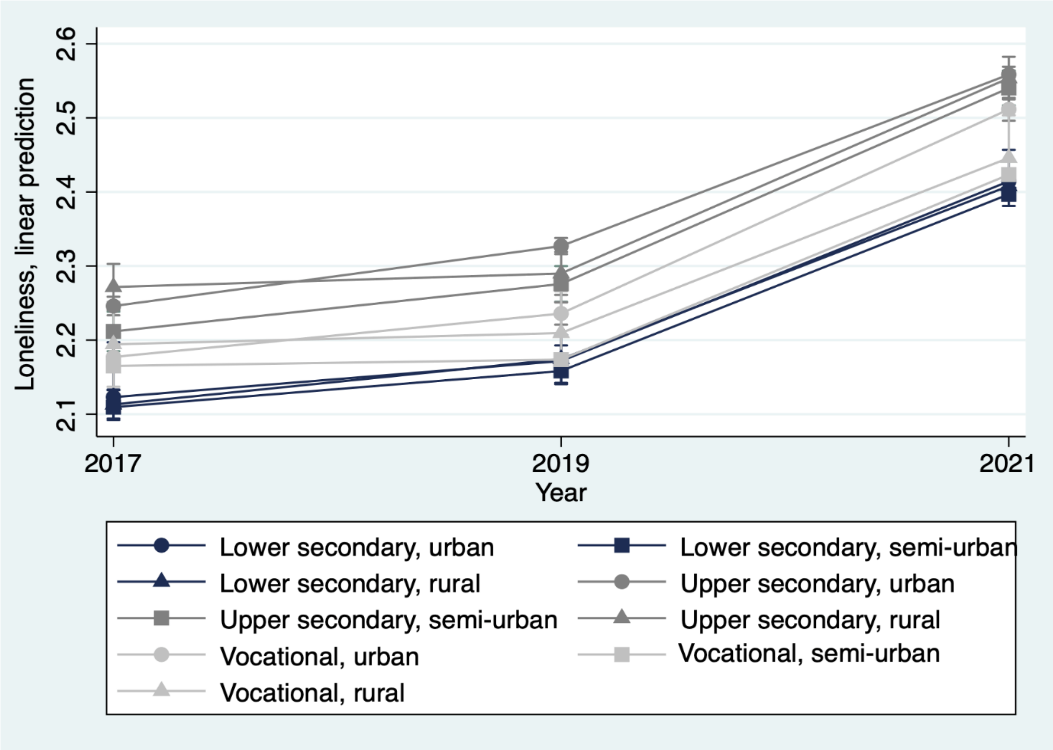

Supplementary Figure 13. Loneliness by school level and urban/rural location of the school in secondary school students in Finland between 2017 and 2021. The linear predictions are shown from a model including an interaction year*school level*urban/rural location of the school and the main effects of gender, parental education and immigration status of the student. Spikes indicate the 95% confidence interval.

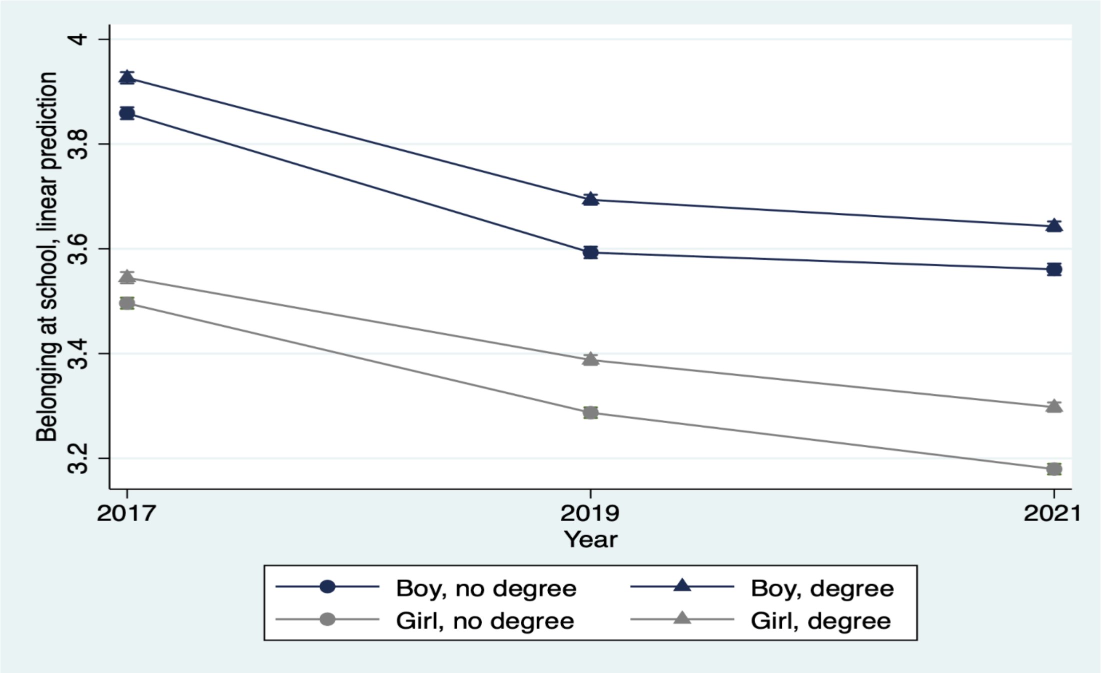

Supplementary Figure 14. Belonging at school by gender and parental degree in secondary schools in Finland between 2017 and 2021. The linear predictions are shown from a model including an interaction year*gender*parental degree and the main effects of school level, immigration status of the student and urban/rural location of the school. Spikes indicate the 95% confidence interval.

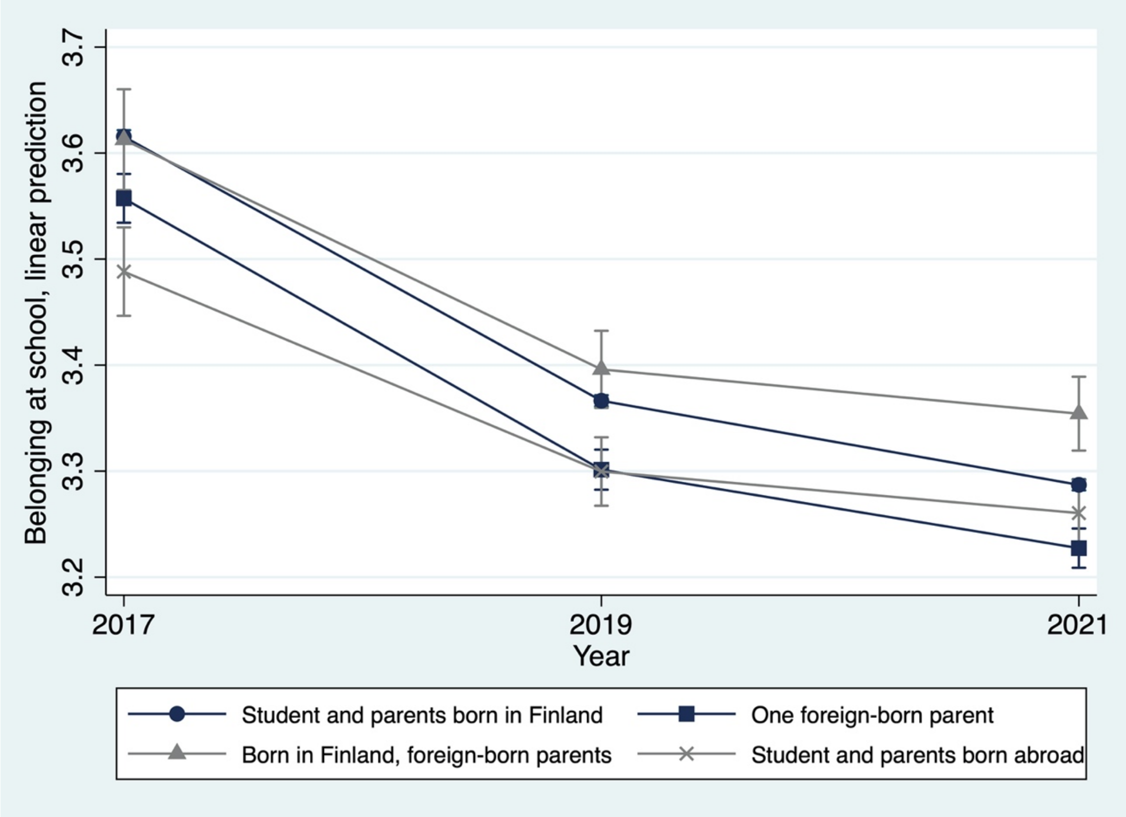

Supplementary Figure 15. Belonging at school by immigrations status of the student in Finland between 2017 and 2021. The linear predictions are shown from a model including an interaction year*immigration status of the student and the main effects of school level, gender, parental education and urban/rural location of the school. Spikes indicate the 95% confidence interval.

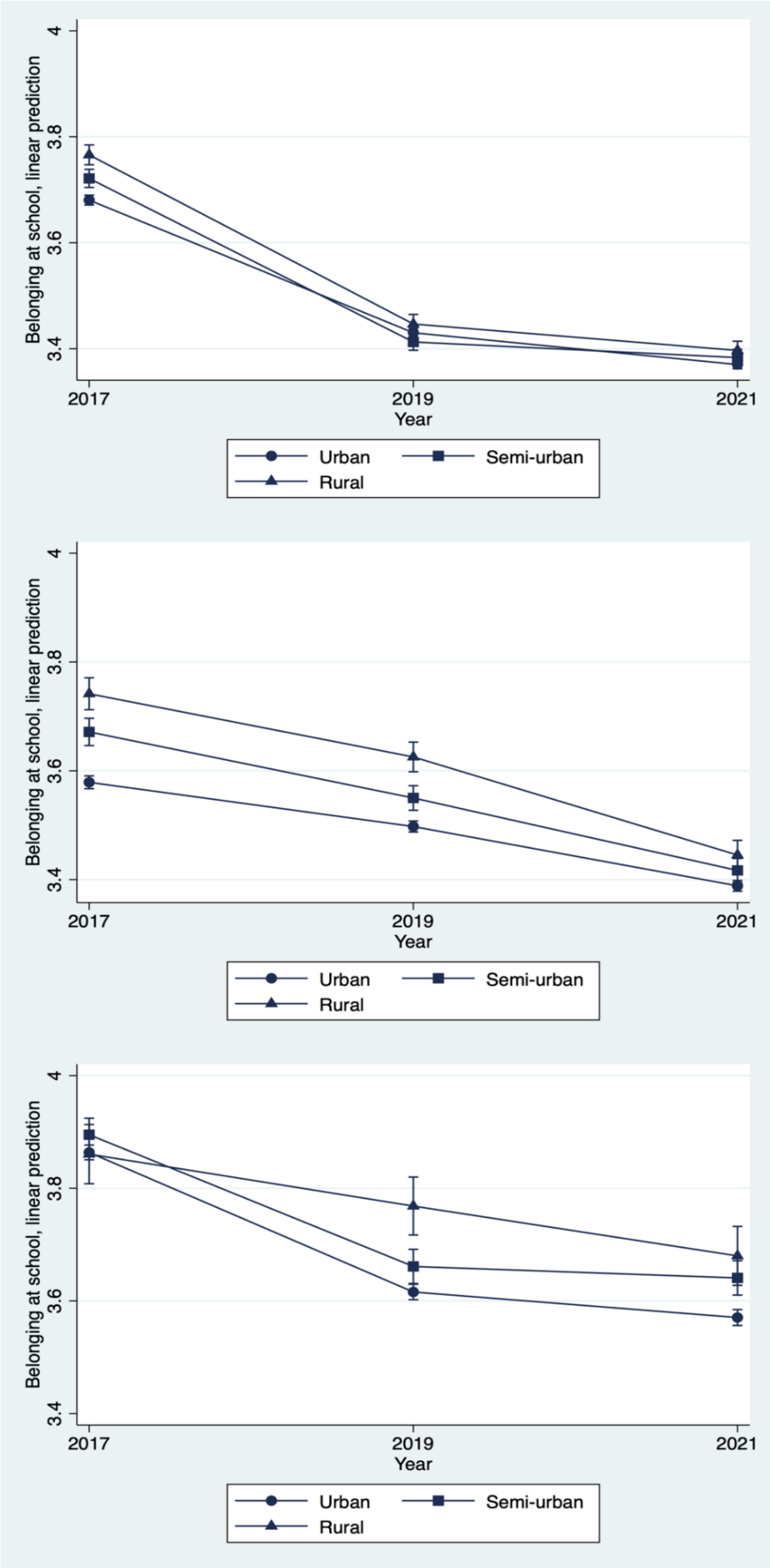

Supplementary Figure 16. Belonging at school by school level and urban/rural location of the school in lower secondary (top figure), upper secondary (middle figure) and vocational school (bottom figure) in Finland between 2017 and 2021. The linear predictions are shown from a model including an interaction year*school level*urban/rural location of the school and the main effects of gender, parental education and immigration status of the student. Spikes indicate the 95% confidence interval.
