# Supplementary material for: The cohort trends of social connectedness in secondary school students in Finland between 2017 and 2021
Source: PLoS One. 2024 Oct 28;19(10):e0312579. doi: 10.1371/journal.pone.0312579 (PMC11516007; doi:10.1371/journal.pone.0312579)
Supplement: S3 File — (PDF) [file pone.0312579.s003.pdf]

# S3 Online resource.

## Postestimation diagnostics and missing data patterns

## Table of Contents

***1 Parallel lines of ordered logistic regression*** ........................................ ***2***

***2 Residuals*** ........................................ ***5***

**Belonging at school** ........................................ **5**

**Loneliness** ........................................ **7**

***3 Multicollinarity*** ........................................ ***9***

***4 Missingness patterns*** ........................................ ***9***

**Having close friends** ........................................ **10**

**Loneliness** ........................................ **11**

**Belonging at school** ........................................ **12**

Note: Assumptions related to residuals are applicable to the model for belonging at school as an outcome (gaussian distribution). Ordered logistic and generalised linear models with different distributions have fewer assumptions. There are also limitations on using postestimation diagnostics with large sample sizes. We display the postestimation results relevant for each outcome. Using robust standard errors (VCE) includes the robustness to heteroskedasticity in the model.

# 1 Parallel lines of ordered logistic regression

```
. ologit closefriend i.year##i.schoollevel##i.degree i.year##i.gender##i.degree i.year##i.urbanrural i.immigrant,
> vce(robust) nolog

Ordered logistic regression                         Number of obs = 415,932
                                                    Wald chi2(32) = 4641.80
                                                    Prob > chi2   =  0.0000
Log pseudolikelihood = -486559.68                   Pseudo R2     =  0.0046

---------------------------------------------------------------------------------------------------------
                                      |               Robust
                          closefriend | Coefficient  std. err.      z    P>|z|     [95% conf. interval]
--------------------------------------+------------------------------------------------------------------
                                 year |
                                 2019 |  -.0075677   .0210131    -0.36   0.719    -.0487526    .0336172
                                 2021 |   -.096368   .0206123    -4.68   0.000    -.1367674   -.0559687
                                      |
                          schoollevel |
                      uppersecondary  |   .0283845   .0198117     1.43   0.152    -.0104457    .0672147
                          vocational  |   .0660981   .0184927     3.57   0.000     .0298531    .1023431
                                      |
                     year#schoollevel |
                  2019#uppersecondary |  -.0303997   .0272321    -1.12   0.264    -.0837737    .0229744
                      2019#vocational |  -.0426343   .0266115    -1.60   0.109     -.094792    .0095234
                  2021#uppersecondary |  -.0453403   .0267034    -1.70   0.090     -.097678    .0069973
                      2021#vocational |  -.0637704   .0264262    -2.41   0.016    -.1155649    -.011976
                                      |
                             1.degree |   .0830313   .0203653     4.08   0.000     .0431161    .1229465
                                      |
                          year#degree |
                               2019 1 |  -.0390669   .0277174    -1.41   0.159     -.093392    .0152583
                               2021 1 |  -.0355742   .0270746    -1.31   0.189    -.0886394    .0174909
                                      |
                   schoollevel#degree |
                    uppersecondary#1  |   .0535405   .0263775     2.03   0.042     .0018415    .1052394
                        vocational#1  |   .0736907   .0312245     2.36   0.018     .0124918    .1348896
                                      |
              year#schoollevel#degree |
                2019#uppersecondary#1 |  -.0177577   .0355944    -0.50   0.618    -.0875215    .0520061
                    2019#vocational#1 |  -.0990492   .0440971    -2.25   0.025     -.185478   -.0126205
                2021#uppersecondary#1 |   .0480867   .0346222     1.39   0.165    -.0197716    .1159451
                    2021#vocational#1 |  -.0645351   .0432533    -1.49   0.136      -.14931    .0202397
                                      |
                               gender |
                                 girl |  -.1774469   .0156063   -11.37   0.000    -.2080347    -.146859
                                      |
                          year#gender |
                            2019#girl |  -.0521759   .0219588    -2.38   0.017    -.0952143   -.0091374
                            2021#girl |   -.188199   .0216272    -8.70   0.000    -.2305875   -.1458105
                                      |
                        gender#degree |
                               girl#1 |    .085323   .0225083     3.79   0.000     .0412075    .1294385
                                      |
                   year#gender#degree |
                          2019#girl#1 |   .0683691    .030773     2.22   0.026     .0080551    .1286831
                          2021#girl#1 |  -.0013818   .0300301    -0.05   0.963    -.0602397    .0574761
                                      |
                           urbanrural |
                           semiurban  |   -.043774     .014742   -2.97   0.003    -.0726678   -.0148802
                               rural  |  -.1225971   .0168289    -7.28   0.000    -.1555811   -.0896131
                                      |
                      year#urbanrural |
                       2019#semiurban |  -.0014675    .020168    -0.07   0.942    -.0409961    .0380611
                           2019#rural |   .0256763   .0232911     1.10   0.270    -.0199735    .0713261
                       2021#semiurban |   .0324364   .0195899     1.66   0.098    -.0059591    .0708319
                           2021#rural |   .0685099   .0226757     3.02   0.003     .0240663    .1129535
                                      |
                            immigrant |
              one parent foreign-born |  -.1450413   .0113482   -12.78   0.000    -.1672834   -.1227993
 born in Finland parents foreign-born |  -.2411996   .0213641   -11.29   0.000    -.2830725   -.1993267
      student and parents born abroad |  -.5139689   .0181593   -28.30   0.000    -.5495604   -.4783774
--------------------------------------+------------------------------------------------------------------
                                /cut1 |  -2.602482   .0161065                      -2.63405   -2.570914
                                /cut2 |  -1.185894   .0153389                     -1.215958   -1.155831
                                /cut3 |  -.2698157    .015169                     -.2995464    -.240085
---------------------------------------------------------------------------------------------------------

. brant, detail

Estimated coefficients from binary logits

------------------------------------------------
    Variable |  y_gt_1      y_gt_2      y_gt_3
-------------+----------------------------------
        year |
        2019 |   -0.057      -0.037       0.012
             |    -1.59       -1.58        0.58
        2021 |   -0.050      -0.086      -0.094
             |    -1.40       -3.71       -4.53
             |
```

| | | | |
|---|---|---|---|
| schoollevel | | | |
| upperseco~y | -0.031 | 0.007 | 0.044 |
| | -0.78 | 0.28 | 2.05 |
| vocational | 0.209 | 0.064 | 0.053 |
| | 5.42 | 2.85 | 2.71 |
| | | | |
| year# | | | |
| schoollevel | | | |
| 2019 # | | | |
| upperseco~y | 0.041 | 0.018 | -0.060 |
| | 0.75 | 0.55 | -2.05 |
| 2019 # | | | |
| vocational | -0.062 | -0.010 | -0.059 |
| | -1.16 | -0.32 | -2.07 |
| | | | |
| year# | | | |
| schoollevel | | | |
| 2021 # | | | |
| upperseco~y | 0.041 | -0.008 | -0.077 |
| | 0.76 | -0.23 | -2.60 |
| 2021 # | | | |
| vocational | -0.124 | -0.094 | -0.048 |
| | -2.32 | -2.95 | -1.68 |
| | | | |
| degree | | | |
| 1 | 0.050 | 0.082 | 0.077 |
| | 1.41 | 3.56 | 3.79 |
| | | | |
| year#degree | | | |
| 2019 1 | -0.045 | -0.019 | -0.042 |
| | -0.96 | -0.63 | -1.54 |
| | | | |
| year#degree | | | |
| 2021 1 | -0.023 | -0.029 | -0.039 |
| | -0.50 | -0.97 | -1.45 |
| | | | |
| schoollevel# | | | |
| degree | | | |
| upperseco~y # | | | |
| 1 | 0.117 | 0.095 | 0.030 |
| | 2.21 | 2.94 | 1.08 |
| | | | |
| schoollevel# | | | |
| degree | | | |
| vocational # | | | |
| 1 | 0.098 | 0.084 | 0.067 |
| | 1.51 | 2.20 | 2.06 |
| | | | |
| year# | | | |
| schoollevel# | | | |
| degree | | | |
| 2019 # | | | |
| upperseco~y # | | | |
| 1 | -0.090 | -0.093 | 0.021 |
| | -1.29 | -2.16 | 0.56 |
| | | | |
| year# | | | |
| schoollevel# | | | |
| degree | | | |
| 2019 # | | | |
| vocational # | | | |
| 1 | -0.136 | -0.141 | -0.075 |
| | -1.55 | -2.66 | -1.64 |
| | | | |
| year# | | | |
| schoollevel# | | | |
| degree | | | |
| 2021 # | | | |
| upperseco~y # | | | |
| 1 | -0.023 | -0.008 | 0.087 |
| | -0.34 | -0.20 | 2.33 |
| | | | |
| year# | | | |
| schoollevel# | | | |
| degree | | | |
| 2021 # | | | |
| vocational # | | | |
| 1 | -0.081 | -0.033 | -0.077 |
| | -0.92 | -0.62 | -1.68 |
| | | | |
| gender | | | |
| girl | 0.572 | 0.031 | -0.336 |
| | 18.11 | 1.66 | -20.51 |
| | | | |
| year#gender | | | |
| 2019#girl | -0.143 | -0.088 | -0.038 |
| | -3.34 | -3.40 | -1.65 |
| | | | |
| year#gender | | | |
| 2021#girl | -0.207 | -0.228 | -0.225 |
| | -4.88 | -8.91 | -9.76 |
| | | | |
| gender#degree | | | |
| girl#1 | -0.075 | 0.057 | 0.137 |

```
             |    -1.66       2.12       5.84
             |
 year#gender#|
      degree |
2019#girl#1  |    0.187      0.093      0.042
             |     3.12       2.56       1.32
             |
 year#gender#|
      degree |
2021#girl#1  |    0.063      0.017     -0.001
             |     1.06       0.48      -0.04
             |
  urbanrural |
  semiurban  |   -0.006     -0.054     -0.043
             |    -0.22      -3.02      -2.74
      rural  |   -0.092     -0.128     -0.126
             |    -2.76      -6.27      -7.00
             |
       year#|
  urbanrural |
      2019 #|
  semiurban  |   -0.002      0.012     -0.007
             |    -0.04       0.48      -0.31
 2019#rural  |    0.066      0.026      0.024
             |     1.45       0.95       0.99
             |
       year#|
  urbanrural |
      2021 #|
  semiurban  |   -0.027      0.044      0.032
             |    -0.70       1.85       1.54
 2021#rural  |    0.020      0.049      0.085
             |     0.46       1.82       3.49
             |
   immigrant |
one paren..  |   -0.207     -0.143     -0.141
             |    -9.75     -10.64     -11.55
born in F..  |   -0.433     -0.249     -0.209
             |   -12.10     -10.25      -9.28
student a..  |   -0.850     -0.502     -0.428
             |   -34.00     -26.47     -23.27
             |
       _cons |    2.282      1.088      0.339
             |    85.81      63.15      22.28
----------------------------------------------
                                  Legend: b/t

Brant test of parallel regression assumption

                                   |       chi2     p>chi2      df
 ----------------------------------+------------------------------
                               All |    6582.78      0.000      64
 ----------------------------------+------------------------------
                         2019.year |       8.22      0.016       2
                         2021.year |       1.72      0.423       2
                    20.schoollevel |       5.01      0.082       2
                    30.schoollevel |      18.66      0.000       2
          2019.year#20.schoollevel |       8.86      0.012       2
          2019.year#30.schoollevel |       4.37      0.112       2
          2021.year#20.schoollevel |       7.96      0.019       2
          2021.year#30.schoollevel |       3.71      0.156       2
                          1.degree |       1.22      0.542       2
                2019.year#1.degree |       1.25      0.536       2
                2021.year#1.degree |       0.21      0.902       2
           20.schoollevel#1.degree |       6.51      0.039       2
           30.schoollevel#1.degree |       0.38      0.825       2
 2019.year#20.schoollevel#1.degree |      10.67      0.005       2
 2019.year#30.schoollevel#1.degree |       2.46      0.292       2
 2021.year#20.schoollevel#1.degree |       7.76      0.021       2
 2021.year#30.schoollevel#1.degree |       1.40      0.498       2
                          2.gender |    1038.10      0.000       2
                2019.year#2.gender |       8.50      0.014       2
                2021.year#2.gender |       0.31      0.857       2
                 2.gender#1.degree |      26.27      0.000       2
       2019.year#2.gender#1.degree |       6.76      0.034       2
       2021.year#2.gender#1.degree |       1.26      0.532       2
                      2.urbanrural |       3.72      0.155       2
                      3.urbanrural |       1.47      0.479       2
            2019.year#2.urbanrural |       0.98      0.614       2
            2019.year#3.urbanrural |       1.00      0.607       2
            2021.year#2.urbanrural |       4.48      0.107       2
            2021.year#3.urbanrural |       3.39      0.183       2
                       2.immigrant |      12.06      0.002       2
                       3.immigrant |      42.44      0.000       2
                       4.immigrant |     322.62      0.000       2

A significant test statistic provides evidence that the parallel
regression assumption has been violated.
```

Note that with large n, as in this model (*n*= 415,932), violation of the parallel lines according to Brant test is common [1,2].

1. Williams RA, Quiroz C. Ordinal Regression Models. SAGE Research Methods Foundations. 2020. doi:10.4135/9781526421036885901

2. Williams R. Ordered Logit Models-Basic & Intermediate Topics. 2022. doi:10.4135/9781526421036885901

# 2 Residuals

## Belonging at school

Note that loneliness had nine categories and was modelled using gamma distribution (=linear regression)

```
. reg belonging i.year##i.gender##i.degree i.year##i.immigrant i.year##i.schoollevel##i.urbanrural,  vce(robust)

Linear regression                               Number of obs     =    415,367
                                                F(44, 415322)     =     562.11
                                                Prob > F          =     0.0000
                                                R-squared         =     0.0562
                                                Root MSE          =     .94614

------------------------------------------------------------------------------------------------------------
                                            |               Robust
                                  belonging | Coefficient  std. err.      t    P>|t|     [95% conf. interval]
--------------------------------------------+---------------------------------------------------------------
                                       year |
                                      2019  |  -.3053881   .0097543   -31.31   0.000    -.3245062     -.28627
                                      2021  |  -.3292969   .0097721   -33.70   0.000    -.3484499    -.3101438
                                            |
                                     gender |
                                      girl  |  -.3643941   .0079095   -46.07   0.000    -.3798965    -.3488916
                                            |
                                year#gender |
                                 2019#girl  |   .0564429   .0108252     5.21   0.000     .0352259     .0776599
                                 2021#girl  |   -.018939   .0109439    -1.73   0.084    -.0403888     .0025107
                                            |
                                   1.degree |   .0679128   .0081384     8.34   0.000     .0519619     .0838638
                                            |
                                year#degree |
                                    2019 1  |   .0301079   .0109094     2.76   0.006     .0087258      .05149
                                    2021 1  |   .0120336   .0109117     1.10   0.270    -.0093531     .0334202
                                            |
                              gender#degree |
                                    girl#1  |  -.0156364   .0111325    -1.40   0.160    -.0374559     .006183
                                            |
                         year#gender#degree |
                               2019#girl#1  |   .0202086   .0148716     1.36   0.174    -.0089393     .0493564
                               2021#girl#1  |    .055896   .0148873     3.75   0.000     .0267174     .0850746
                                            |
                                  immigrant |
                   one parent foreign-born  |  -.0547131   .0116943    -4.68   0.000    -.0776336    -.0317926
      born in Finland parents foreign-born  |  -.0135615   .0236059    -0.57   0.566    -.0598283     .0327054
           student and parents born abroad  |  -.1444653   .0209534    -6.89   0.000    -.1855333    -.1033972
                                            |
                             year#immigrant |
              2019#one parent foreign-born  |  -.0046127   .0152435    -0.30   0.762    -.0344894     .0252641
 2019#born in Finland parents foreign-born  |   .0471466   .0297948     1.58   0.114    -.0112503     .1055436
      2019#student and parents born abroad  |    .065116   .0266314     2.45   0.014     .0129192     .1173127
              2021#one parent foreign-born  |  -.0035395   .0151317    -0.23   0.815    -.0331971     .0261181
 2021#born in Finland parents foreign-born  |    .079337    .029412     2.70   0.007     .0216904     .1369837
      2021#student and parents born abroad  |   .1102693   .0261656     4.21   0.000     .0589854     .1615532
                                            |
                                schoollevel |
                           uppersecondary   |  -.0866097   .0078611   -11.02   0.000    -.1020173    -.0712022
                               vocational   |   .1536111   .0086342    17.79   0.000     .1366883     .1705339
                                            |
                           year#schoollevel |
                      2019#uppersecondary   |   .1610388   .0102475    15.71   0.000      .140954     .1811235
                          2019#vocational   |   .0159963   .0117547     1.36   0.174    -.0070426     .0390352
                      2021#uppersecondary   |   .1172002   .0101997    11.49   0.000     .0972092     .1371913
                          2021#vocational   |   .0256947   .0120003     2.14   0.032     .0021745     .0492149
                                            |
                                 urbanrural |
                                semiurban   |    .034015   .0101621     3.35   0.001     .0140975     .0539324
                                    rural   |   .0766209    .010925     7.01   0.000     .0552081     .0980337
                                            |
                            year#urbanrural |
                           2019#semiurban   |  -.0505095   .0135973    -3.71   0.000    -.0771598    -.0238592
                               2019#rural   |  -.0587615    .014902    -3.94   0.000     -.087969     -.029554
                           2021#semiurban   |   -.018507   .0134678    -1.37   0.169    -.0449035     .0078895
```

```
                     2021#rural  |  -.0473066   .0147017    -3.22   0.001    -.0761215   -.0184916
                                 |
          schoollevel#urbanrural |
   uppersecondary#semiurban  |   .0500718   .0172032     2.91   0.004     .0163541    .0837895
      uppersecondary#rural  |   .0758928    .019352     3.92   0.000     .0379634    .1138221
      vocational#semiurban  |  -.0048197   .0192604    -0.25   0.802    -.0425695    .0329301
          vocational#rural  |  -.0791309   .0304435    -2.60   0.009    -.1387992   -.0194626
                                 |
     year#schoollevel#urbanrural |
2019#uppersecondary#semiurban  |   .0181522   .0229034     0.79   0.428    -.0267378    .0630422
   2019#uppersecondary#rural  |     .03329   .0263065     1.27   0.206      -.01827      .08485
   2019#vocational#semiurban  |   .0746026   .0266569     2.80   0.005     .0223559    .1268493
       2019#vocational#rural  |   .2187153   .0412271     5.31   0.000     .1379115    .2995191
2021#uppersecondary#semiurban  |  -.0375194   .0228956    -1.64   0.101    -.0823941    .0073552
   2021#uppersecondary#rural  |   -.047373   .0263407    -1.80   0.072    -.0989998    .0042539
   2021#vocational#semiurban  |   .0636591   .0270753     2.35   0.019     .0105924    .1167258
       2021#vocational#rural  |   .1665557   .0418292     3.98   0.000     .0845717    .2485398
                                 |
                           _cons |   3.849917   .0072621   530.14   0.000     3.835683     3.86415
------------------------------------------------------------------------------------------------------------

. predict r, resid
(35,497 missing values generated)

. pnorm r
```

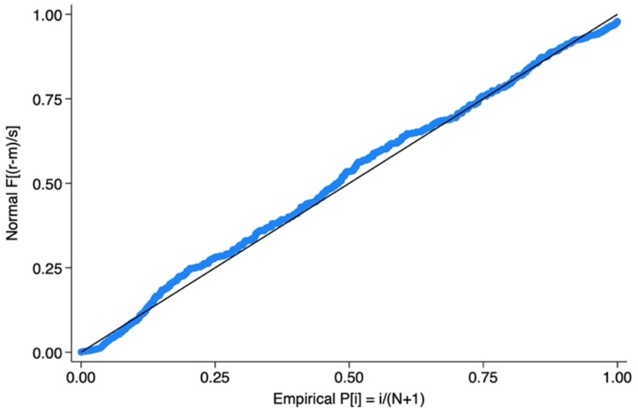


```
. qnorm r
```

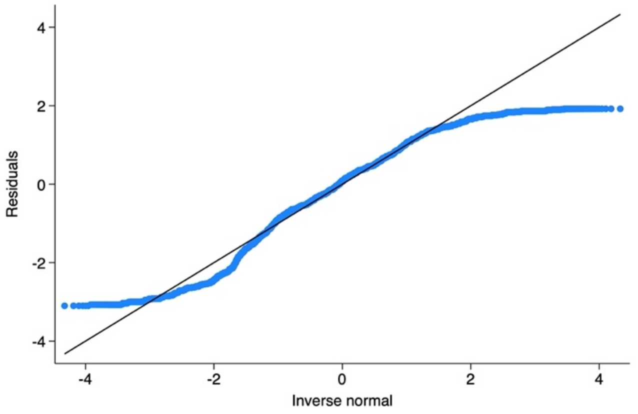


The plots above suggest relatively good estimation in the middle of the scale (pnorm), but some deviation at the tails of the scale (qnorm).

```
. iqr r

   mean=  6.0e-10         std.dev.=  .9461          (n= 415367)
 median=  .0822    pseudo std.dev.=  .8985        (IQR=  1.212)
10 trim=  .0527
                                               low         high
                                               -------------------
                                inner fences   -2.369       2.479
                              # mild outliers  8151         0
                              % mild outliers  1.96%        0.00%

                                outer fences   -4.187       4.297
                            # severe outliers  0            0
                            % severe outliers  0.00%        0.00%

hist r, normal bins(15)
```

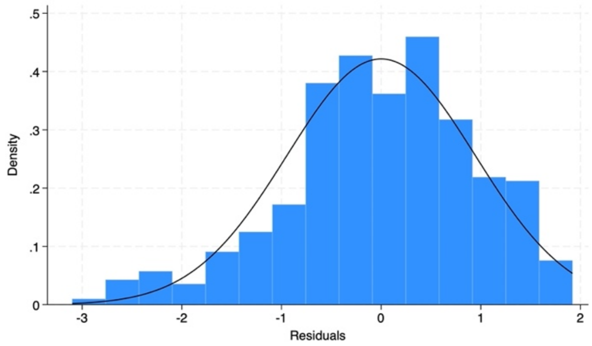


Small number of mild outliers, none severe. Residuals approximately normally distributed.

## Loneliness

### Note that loneliness had five categories and was modelled using gamma distribution

```
glm loneliness i.year##i.schoollevel##i.urbanrural i.gender i.immigrant i.degree, family(gamma) link (log)
vce(robust) nolog
```

```
Generalized linear models                         Number of obs     =    416,572
Optimization     : ML                             Residual df       =    416,540
                                                  Scale parameter =   .1957291
Deviance         =  84559.40057                   (1/df) Deviance =   .2030043
Pearson          =  81529.00748                   (1/df) Pearson  =   .1957291

Variance function: V(u) = u^2                     [Gamma]
Link function    : g(u) = ln(u)                   [Log]

                                                  AIC               =   3.637639
Log pseudolikelihood =  -757637.253               BIC               =   -5305391
```

| loneliness | Coefficient | Robust std. err. | z | P>\|z\| | [95% conf. | interval] |
|---|---|---|---|---|---|---|
| **year** | | | | | | |
| 2019 | .020483 | .0030143 | 6.80 | 0.000 | .0145751 | .0263909 |
| 2021 | .1261702 | .0029306 | 43.05 | 0.000 | .1204263 | .1319141 |
| **schoollevel** | | | | | | |
| uppersecondary | .0599358 | .0035667 | 16.80 | 0.000 | .0529452 | .0669265 |
| vocational | .0220498 | .0041041 | 5.37 | 0.000 | .0140058 | .0300937 |
| **year#schoollevel** | | | | | | |
| 2019#uppersecondary | .0138811 | .0046735 | 2.97 | 0.003 | .0047211 | .023041 |
| 2019#vocational | .004509 | .0058087 | 0.78 | 0.438 | -.0068759 | .0158939 |
| 2021#uppersecondary | .0013359 | .0045359 | 0.29 | 0.768 | -.0075543 | .010226 |
| 2021#vocational | .0196997 | .0057036 | 3.45 | 0.001 | .0085209 | .0308785 |
| **urbanrural** | | | | | | |
| semiurban | -.0059382 | .0047384 | -1.25 | 0.210 | -.0152253 | .0033489 |
| rural | -.0051518 | .0050767 | -1.01 | 0.310 | -.0151019 | .0047982 |
| **year#urbanrural** | | | | | | |
| 2019#semiurban | -.000824 | .0063853 | -0.13 | 0.897 | -.0133389 | .0116909 |
| 2019#rural | .005766 | .0069198 | 0.83 | 0.405 | -.0077965 | .0193284 |
| 2021#semiurban | -.0007394 | .0062264 | -0.12 | 0.905 | -.012943 | .0114642 |
| 2021#rural | .0021437 | .0067554 | 0.32 | 0.751 | -.0110966 | .0153839 |
| **schoollevel#urbanrural** | | | | | | |
| uppersecondary#semiurban | -.0091486 | .0080631 | -1.13 | 0.257 | -.0249519 | .0066548 |
| uppersecondary#rural | .0162943 | .0088909 | 1.83 | 0.067 | -.0011316 | .0337201 |
| vocational#semiurban | -.0048904 | .0095913 | -0.51 | 0.610 | -.0236891 | .0139082 |
| vocational#rural | .0104382 | .0148826 | 0.70 | 0.483 | -.0187312 | .0396077 |
| **year#schoollevel#urbanrural** | | | | | | |
| 2019#uppersecondary#semiurban | -.0063643 | .0107314 | -0.59 | 0.553 | -.0273975 | .0146688 |
| 2019#uppersecondary#rural | -.0334125 | .0121012 | -2.76 | 0.006 | -.0571304 | -.0096946 |
| 2019#vocational#semiurban | -.0191848 | .0137707 | -1.39 | 0.164 | -.0461749 | .0078053 |
| 2019#vocational#rural | -.0265259 | .0206505 | -1.28 | 0.199 | -.0670001 | .0139482 |
| 2021#uppersecondary#semiurban | .0085482 | .0104151 | 0.82 | 0.412 | -.0118651 | .0289614 |
| 2021#uppersecondary#rural | -.0148649 | .0117294 | -1.27 | 0.205 | -.0378542 | .0081244 |
| 2021#vocational#semiurban | -.0279862 | .0134133 | -2.09 | 0.037 | -.0542758 | -.0016967 |
| 2021#vocational#rural | -.041372 | .0203013 | -2.04 | 0.042 | -.0811618 | -.0015822 |
| **gender** | | | | | | |
| girl | .2670311 | .0013948 | 191.45 | 0.000 | .2642974 | .2697648 |

```
                            immigrant |
            one parent foreign-born   |   .0468037   .0026628    17.58   0.000     .0415848    .0520226
born in Finland parents foreign-born  |  -.0298014    .005651    -5.27   0.000    -.0408772   -.0187257
     student and parents born abroad  |   .0644089   .0048566    13.26   0.000     .0548902    .0739276
                                      |
                             1.degree |  -.0083081   .0014346    -5.79   0.000    -.0111198   -.0054965
                                _cons |   .6031427   .0025763   234.11   0.000     .5980933    .6081922
```

```
pnorm dr_logitlone
```

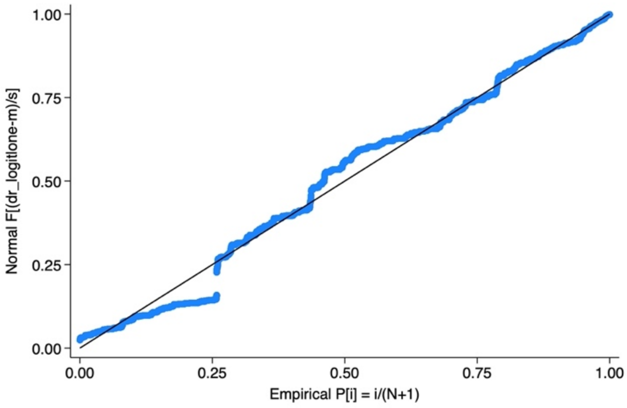


```
qnorm dr_logitlone
```

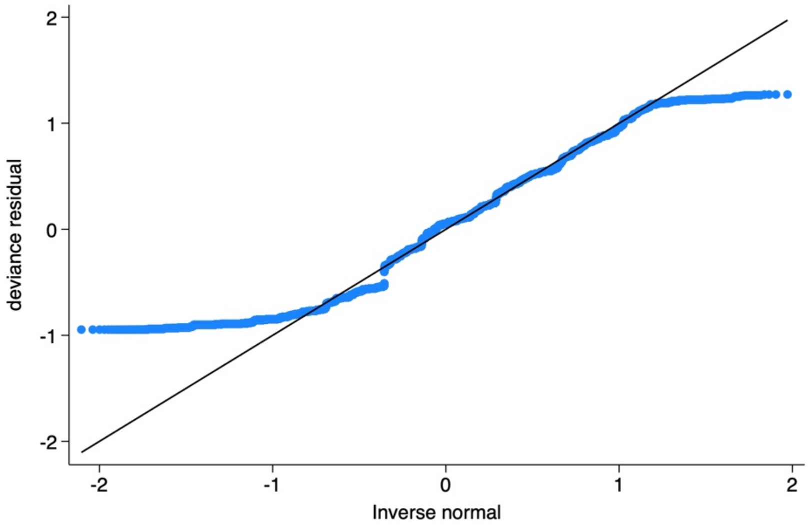


```
iqr dr_logitlone

   mean= -.0663          std.dev.=  .4456          (n= 416572)
 median= -.0042   pseudo std.dev.=  .5675         (IQR=  .7656)
10 trim= -.0713
                                                low          high
                                                -------------------
                              inner fences     -1.69         1.373
                            # mild outliers     0            0
                            % mild outliers     0.00%        0.00%

                              outer fences     -2.838        2.521
                          # severe outliers     0            0
                          % severe outliers     0.00%        0.00%

hist dr_logitlone, normal bins(8)
```

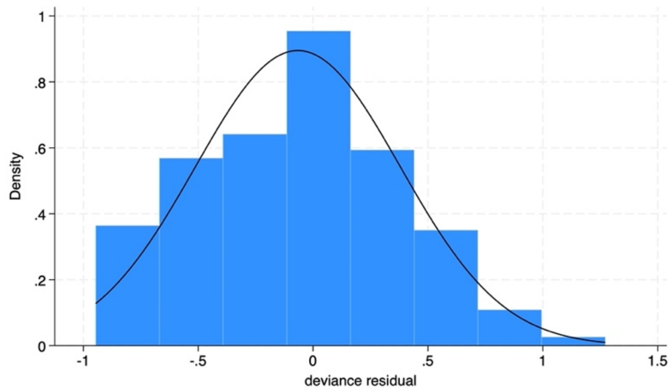


# 3 Multicollinearity

```
. collin year gender schoollevel degree urbanrural immigrant
(obs=418,225)

  Collinearity Diagnostics

                        SQRT                   R-
  Variable      VIF     VIF    Tolerance    Squared
----------------------------------------------------
      year      1.01    1.00    0.9936      0.0064
    gender      1.00    1.00    0.9981      0.0019
schoollevel     1.02    1.01    0.9763      0.0237
    degree      1.03    1.01    0.9715      0.0285
urbanrural      1.04    1.02    0.9603      0.0397
 immigrant      1.01    1.00    0.9919      0.0081
----------------------------------------------------
  Mean VIF      1.02

                           Cond
        Eigenval           Index
---------------------------------
    1     5.9769          1.0000
    2     0.4448          3.6655
    3     0.2180          5.2357
    4     0.1966          5.5144
    5     0.1191          7.0831
    6     0.0446         11.5789
    7     0.0000       4345.5817
---------------------------------
 Condition Number      4345.5817
 Eigenvalues & Cond Index computed from scaled raw sscp (w/ intercept)
 Det(correlation matrix)     0.9481
```

Multicollinearity not evident based on VIF (variance inflation factor) and tolerance values.

Multicollinearity should be inspected when:

- VIF values are large, individual VIF greater than 10 or average VIF greater than 6
- tolerance values are small, close to zero, tolerance less than .1

Source: http://www.philender.com/courses/categorical/notes2/collin.html

# 4 Missingness patterns

```
. mcartest belonging loneliness closefriend year gender schoollevel degree  immigrant urbanrural

Little's MCAR test

Number of obs        = 450864
Chi-square distance = 26009.6800
Degrees of freedom  = 321
Prob > chi-square   = 0.0000
```

MCAR test: not missing completely at random

Proportions of missingness in each outcome by background factors
Note: 1=missing, 0=not missing

## Having close friends

```
. tab  year closefriendmi, row
```

| year | closefriendmi 0 | 1 | Total |
|---|---|---|---|
| 2017 | 132,542 | 2,360 | 134,902 |
| | 98.25 | 1.75 | 100.00 |
| 2019 | 153,263 | 1,903 | 155,166 |
| | 98.77 | 1.23 | 100.00 |
| 2021 | 159,117 | 1,679 | 160,796 |
| | 98.96 | 1.04 | 100.00 |
| Total | 444,922 | 5,942 | 450,864 |
| | 98.68 | 1.32 | 100.00 |

```
. tab  gender closefriendmi , row
```

| gender | closefriendmi 0 | 1 | Total |
|---|---|---|---|
| boy | 213,926 | 4,111 | 218,037 |
| | 98.11 | 1.89 | 100.00 |
| girl | 229,670 | 1,531 | 231,201 |
| | 99.34 | 0.66 | 100.00 |
| Total | 443,596 | 5,642 | 449,238 |
| | 98.74 | 1.26 | 100.00 |

```
. tab  schoollevel closefriendmi , row
```

| schoollevel | closefriendmi 0 | 1 | Total |
|---|---|---|---|
| lowersecondary | 248,372 | 4,081 | 252,453 |
| | 98.38 | 1.62 | 100.00 |
| uppersecondary | 125,717 | 811 | 126,528 |
| | 99.36 | 0.64 | 100.00 |
| vocational | 70,833 | 1,050 | 71,883 |
| | 98.54 | 1.46 | 100.00 |
| Total | 444,922 | 5,942 | 450,864 |
| | 98.68 | 1.32 | 100.00 |

```
. tab  degree closefriendmi , row
```

| degree | closefriendmi 0 | 1 | Total |
|---|---|---|---|
| 0 | 193,184 | 1,164 | 194,348 |
| | 99.40 | 0.60 | 100.00 |
| 1 | 227,874 | 1,316 | 229,190 |
| | 99.43 | 0.57 | 100.00 |
| Total | 421,058 | 2,480 | 423,538 |
| | 99.41 | 0.59 | 100.00 |

```
. tab immigrant  closefriendmi , row
```

| immigrant | closefriendmi 0 | 1 | Total |
|---|---|---|---|
| student and parents b | 375,869 | 2,248 | 378,117 |
| | 99.41 | 0.59 | 100.00 |
| one parent foreign-bo | 30,722 | 194 | 30,916 |

```
                      |     99.37       0.63 |    100.00 
----------------------+----------------------+----------
born in Finland paren |     8,714         98 |     8,812 
                      |     98.89       1.11 |    100.00 
----------------------+----------------------+----------
student and parents b |    13,732        185 |    13,917 
                      |     98.67       1.33 |    100.00 
----------------------+----------------------+----------
                Total |   429,037      2,725 |   431,762 
                      |     99.37       0.63 |    100.00 
```

```
. tab urbanrural closefriendmi , row
```

```
           |     closefriendmi
urbanrural |         0          1 |     Total
-----------+----------------------+----------
     urban |   315,622      4,410 |   320,032 
           |     98.62       1.38 |    100.00 
-----------+----------------------+----------
 semiurban |    76,352        973 |    77,325 
           |     98.74       1.26 |    100.00 
-----------+----------------------+----------
     rural |    52,948        559 |    53,507 
           |     98.96       1.04 |    100.00 
-----------+----------------------+----------
     Total |   444,922      5,942 |   450,864 
           |     98.68       1.32 |    100.00 
```

Loneliness

```
. tab  year lonelinessmi , row
```

```
           |     lonelinessmi
      year |         0          1 |     Total
-----------+----------------------+----------
      2017 |   132,986      1,916 |   134,902 
           |     98.58       1.42 |    100.00 
-----------+----------------------+----------
      2019 |   153,638      1,528 |   155,166 
           |     99.02       0.98 |    100.00 
-----------+----------------------+----------
      2021 |   159,118      1,678 |   160,796 
           |     98.96       1.04 |    100.00 
-----------+----------------------+----------
     Total |   445,742      5,122 |   450,864 
           |     98.86       1.14 |    100.00 
```

```
. tab  gender lonelinessmi , row
```

```
           |     lonelinessmi
    gender |         0          1 |     Total
-----------+----------------------+----------
       boy |   214,465      3,572 |   218,037 
           |     98.36       1.64 |    100.00 
-----------+----------------------+----------
      girl |   229,949      1,252 |   231,201 
           |     99.46       0.54 |    100.00 
-----------+----------------------+----------
     Total |   444,414      4,824 |   449,238 
           |     98.93       1.07 |    100.00 
```

```
. tab  schoollevel lonelinessmi , row
```

```
               |     lonelinessmi
   schoollevel |         0          1 |     Total
---------------+----------------------+----------
lowersecondary |   248,940      3,513 |   252,453 
               |     98.61       1.39 |    100.00 
---------------+----------------------+----------
uppersecondary |   125,867        661 |   126,528 
               |     99.48       0.52 |    100.00 
---------------+----------------------+----------
    vocational |    70,935        948 |    71,883 
               |     98.68       1.32 |    100.00 
---------------+----------------------+----------
         Total |   445,742      5,122 |   450,864 
               |     98.86       1.14 |    100.00 
```

```
. tab  degree lonelinessmi , row
```

```
           |     lonelinessmi
```

| degree | 0 | 1 | Total |
|---|---|---|---|
| 0 | 193,439 | 909 | 194,348 |
| | 99.53 | 0.47 | 100.00 |
| 1 | 228,262 | 928 | 229,190 |
| | 99.60 | 0.40 | 100.00 |
| Total | 421,701 | 1,837 | 423,538 |
| | 99.57 | 0.43 | 100.00 |

. tab immigrant  lonelinessmi , row

| | lonelinessmi | | |
|---|---|---|---|
| immigrant | 0 | 1 | Total |
| student and parents b | 376,529 | 1,588 | 378,117 |
| | 99.58 | 0.42 | 100.00 |
| one parent foreign-bo | 30,764 | 152 | 30,916 |
| | 99.51 | 0.49 | 100.00 |
| born in Finland paren | 8,741 | 71 | 8,812 |
| | 99.19 | 0.81 | 100.00 |
| student and parents b | 13,746 | 171 | 13,917 |
| | 98.77 | 1.23 | 100.00 |
| Total | 429,780 | 1,982 | 431,762 |
| | 99.54 | 0.46 | 100.00 |

. tab urbanrural lonelinessmi , row

| | lonelinessmi | | |
|---|---|---|---|
| urbanrural | 0 | 1 | Total |
| urban | 316,194 | 3,838 | 320,032 |
| | 98.80 | 1.20 | 100.00 |
| semiurban | 76,493 | 832 | 77,325 |
| | 98.92 | 1.08 | 100.00 |
| rural | 53,055 | 452 | 53,507 |
| | 99.16 | 0.84 | 100.00 |
| Total | 445,742 | 5,122 | 450,864 |
| | 98.86 | 1.14 | 100.00 |

### Belonging at school

. tab  year belongingmi , row

| | belongingmi | | |
|---|---|---|---|
| year | 0 | 1 | Total |
| 2017 | 127,235 | 7,667 | 134,902 |
| | 94.32 | 5.68 | 100.00 |
| 2019 | 154,101 | 1,065 | 155,166 |
| | 99.31 | 0.69 | 100.00 |
| 2021 | 159,859 | 937 | 160,796 |
| | 99.42 | 0.58 | 100.00 |
| Total | 441,195 | 9,669 | 450,864 |
| | 97.86 | 2.14 | 100.00 |

. tab  gender belongingmi , row

| | belongingmi | | |
|---|---|---|---|
| gender | 0 | 1 | Total |
| boy | 211,579 | 6,458 | 218,037 |
| | 97.04 | 2.96 | 100.00 |
| girl | 228,340 | 2,861 | 231,201 |
| | 98.76 | 1.24 | 100.00 |
| Total | 439,919 | 9,319 | 449,238 |
| | 97.93 | 2.07 | 100.00 |

. tab  schoollevel belongingmi , row

| | belongingmi |
|---|---|

| schoollevel | 0 | 1 | Total |
|---|---|---|---|
| lowersecondary | 245,932 | 6,521 | 252,453 |
| | 97.42 | 2.58 | 100.00 |
| uppersecondary | 125,475 | 1,053 | 126,528 |
| | 99.17 | 0.83 | 100.00 |
| vocational | 69,788 | 2,095 | 71,883 |
| | 97.09 | 2.91 | 100.00 |
| Total | 441,195 | 9,669 | 450,864 |
| | 97.86 | 2.14 | 100.00 |

. tab  degree belongingmi , row

| | belongingmi | | |
|---|---|---|---|
| degree | 0 | 1 | Total |
| 0 | 192,429 | 1,919 | 194,348 |
| | 99.01 | 0.99 | 100.00 |
| 1 | 227,785 | 1,405 | 229,190 |
| | 99.39 | 0.61 | 100.00 |
| Total | 420,214 | 3,324 | 423,538 |
| | 99.22 | 0.78 | 100.00 |

. tab immigrant  belongingmi , row

| | belongingmi | | |
|---|---|---|---|
| immigrant | 0 | 1 | Total |
| student and parents b | 375,297 | 2,820 | 378,117 |
| | 99.25 | 0.75 | 100.00 |
| one parent foreign-bo | 30,646 | 270 | 30,916 |
| | 99.13 | 0.87 | 100.00 |
| born in Finland paren | 8,680 | 132 | 8,812 |
| | 98.50 | 1.50 | 100.00 |
| student and parents b | 13,531 | 386 | 13,917 |
| | 97.23 | 2.77 | 100.00 |
| Total | 428,154 | 3,608 | 431,762 |
| | 99.16 | 0.84 | 100.00 |

. tab urbanrural belongingmi , row

| | belongingmi | | |
|---|---|---|---|
| urbanrural | 0 | 1 | Total |
| urban | 313,033 | 6,999 | 320,032 |
| | 97.81 | 2.19 | 100.00 |
| semiurban | 75,665 | 1,660 | 77,325 |
| | 97.85 | 2.15 | 100.00 |
| rural | 52,497 | 1,010 | 53,507 |
| | 98.11 | 1.89 | 100.00 |
| Total | 441,195 | 9,669 | 450,864 |
| | 97.86 | 2.14 | 100.00 |
